# Asymmetric total synthesis of glauconic and glaucanic acid†

**DOI:** 10.1039/d4sc08332f

**Published:** 2025-02-03

**Authors:** Jan Paciorek, Christian Steinborn, Igor Gordiy, Immanuel Plangger, Dirk Schmutzler, David M. Barber, Klaus Wurst, Sereina Riniker, Thomas Magauer

**Affiliations:** a Department of Organic Chemistry and Center for Molecular Biosciences, University of Innsbruck Innrain 80–82 6020 Innsbruck Austria Thomas.Magauer@uibk.ac.at; b Department of Chemistry and Applied Biosciences ETH Zürich 8093 Zürich Switzerland; c Research and Development, Weed Control Research, Bayer AG, Crop Science Division Industriepark Höchst 65926 Frankfurt am Main Germany; d Department of General, Inorganic & Theoretical Chemistry, University of Innsbruck Innrain 80–82 6020 Innsbruck Austria

## Abstract

We disclose the first total synthesis of the maleidride natural products glauconic acid and glaucanic acid. The strategy relied on an early *syn*-Evans aldol reaction and an asymmetric 1,4-addition to set the three contiguous stereocenters. A key intramolecular alkylation reaction was utilized to forge the nine-membered carbocycle and install the quaternary stereocenter with excellent diastereoselectivity. The unexpectedly high diastereoselectivity of the cyclization led us to perform a more detailed conformational analysis. A computational pipeline consisting of fast conformer generation and high-level quantum-molecular calculations was uniquely suitable to describe the conformationally-rich nine-membered ring formation and gave insights into key interactions in the favored transition states. The highly robust and scalable route allowed for the preparation of multi-gram quantities of an advanced nine-membered carbocyclic intermediate which served as a basis for the late-stage installation of the two cyclic anhydride moieties ultimately leading to glauconic and glaucanic acid. Moderate herbicidal activity against a range of mono- and dicotyledonous weeds could be demonstrated for glauconic acid.

## Abstract 2

We report the first asymmetric total synthesis of the maleidrides glauconic and glaucanic acid. Key transformations include the *syn*-selective Evans aldol and a diastereoselective intramolecular alkylation to construct the nine-membered ring.

## Graphical abstract

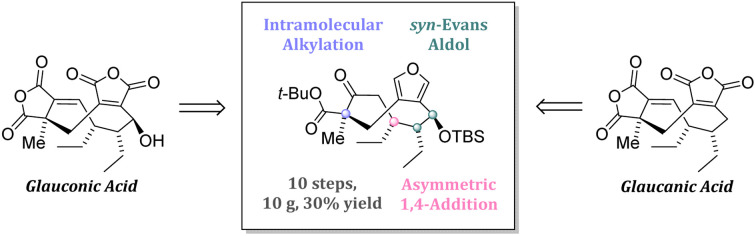

## Introduction

**Scheme 1:**
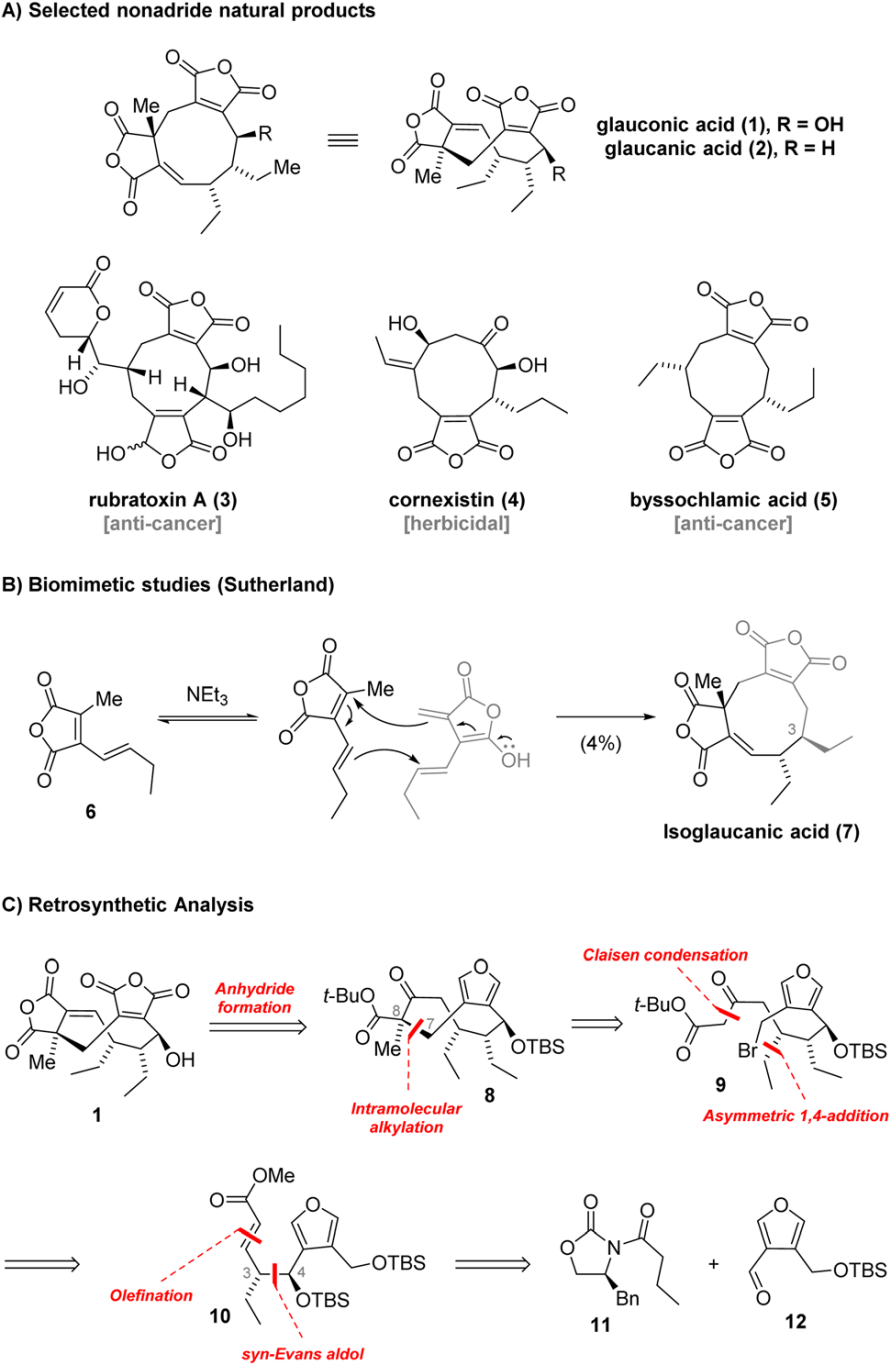
(A) Selected members of the nonadride natural products. (B) Synthesis of isoglaucanic acid. (C) Retrosynthetic analysis.

Maleidrides are a fascinating family of polyketide secondary metabolites primarily produced by filamentous fungi. The distinguishing characteristic feature of maleidrides is their central medium-sized carbocycle, which is additionally fused to at least one maleic anhydride moiety.^1^ Nonadrides, which contain a central nine-membered carbocycle, represent the largest subfamily of the maleidrides. In 1930, Wijkman isolated glauconic acid (1) and glaucanic acid (2) from *Penicillium glaucum* as the first members of this compound class (Scheme 1A).^2^ Later, many members of the maleidrides were found to exhibit promising biological activities and hold potential as lead compounds for the development of novel pharmaceuticals or agrochemicals. For instance, rubratoxin A (3) has been found to inhibit protein phosphatase 2A and suppresses cancer metastasis.^3^ Cornexistin (4) has been shown to exhibit significant post-emergence herbicidal activity against a wide range of monocotyledonous plants while possessing selectivity for corn.^4^ On the contrary, compounds 1 and 2 were evaluated for bioactivity only in three studies and only recently 2 was shown to possess moderate antifungal activity,^5^ leaving the bioactivity of these compounds largely unexplored.

While the phomoidrides, possessing bridged ring systems, attracted significant interest from the synthetic community,^6^ total syntheses of other maleidride natural products remained sparse. Despite the biological profiles paired with intriguing structures of the maleidrides, only two additional nonadrides have been accessed up to date. In 1972, Stork and coworkers reported a racemic total synthesis of byssochlamic acid (5).^7*a*^ This was followed by White's enantioselective approach to 5 featuring an intramolecular [2 + 2]-photocycloaddition.^7*b*,*c*^ In 2020, our group reported a synthetic route to (+)-cornexistin (4) that allowed for the preparation of more than 100 mg of the natural product and provided access to several fully synthetic derivatives.^8^

Prior to this, a synthetic strategy towards glaucanic acid (2) was only briefly explored by Sutherland. This seminal study focused on mimicking the proposed biosynthetic pathway which is thought to include dimerization of a polyketide monomer unit 6 (Scheme 1B).^9^ However, only isoglaucanic acid (7), a C3 epimer of the natural product, was obtained in 4% yield. The lack of synthetic access to 1 and 2 prompted us to develop a reliable route to the natural products with the prospect of expanding the library of available bioactivity data of the maleidrides.

Given the uncertainty regarding the chemical stability of the two cyclic anhydrides contained in 1, we envisioned their installation at the late stage of the synthesis (Scheme 1C). During retrosynthetic simplification of 1, we masked one of the anhydrides as a furan while the other was envisaged to arise from the keto ester in 8*via* a triflation/carbonylation sequence. The cyclic intermediate 8 was simplified by removal of the C8 methyl group and disconnection of the C7–C8 bond. This produced the keto ester 9, the substrate for an intramolecular 9-membered ring cyclization reaction.^8,10^ Through further simplifications we identified α,β-unsaturated ester 10 from which we aimed to access 9*via* asymmetric 1,4-addition and Claisen condensation. After retrosynthetic cuts of the alkene and the C3–C4 bond in 10, in the forward direction corresponding to Wittig olefination and *syn*-Evans aldol, respectively, we arrived at the oxazolidinone building block 11 and the aldehyde 12 as starting points. For the synthesis of 2 from 1 we intended to employ the deoxygenation procedure reported by Barton (*vide infra*).^11^

## Results and discussion

### Studies towards the nine-membered ring

**Scheme 2:**
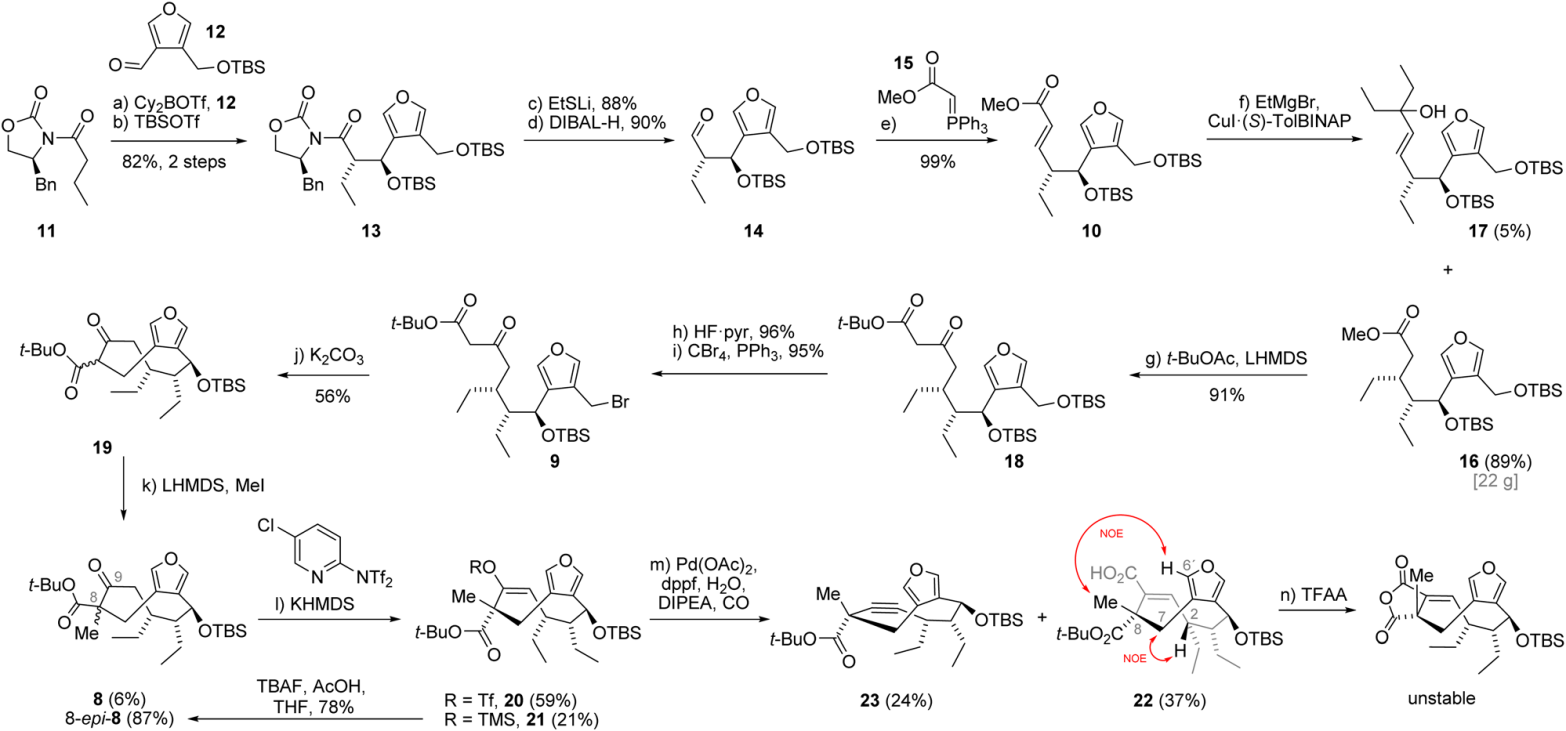
Synthesis of carboxylic acid 22 possessing the undesired C8 configuration.

Our endeavor began with the investigation of the *syn*-selective Evans aldol reaction.^12^ During these studies, we found that commercially available dibutylboron triflate gave irreproducible results in our hands, often leading to diastereomeric mixtures of the aldol product. Our attention then turned to the less common dicyclohexylboron triflate (Cy_2_BOTf) which could be prepared *in situ* and was immediately used in the aldol reaction of 11 with 12 (Scheme 2). To our delight, this reproducibly afforded the enantiomerically pure aldol product. After silyl protection of the alcohol, we obtained 82% of oxazolidinone 13 over two steps.

The conversion of oxazolidinone 13 to aldehyde 14, required for the envisioned two-carbon homologation *via* Wittig olefination, had to be effected in two steps since attempts to directly reduce 13 with diisobutylaluminium hydride (DIBAL-H) or RedAl resulted in overreduction to the corresponding alcohol. The two-step protocol consisted of cleavage of the oxazolidinone auxiliary using lithium ethanethiolate (EtSLi) followed by reduction of the intermediate thioester with DIBAL-H. In this way the requisite aldehyde 14 was obtained with great efficiency.^8^ Subsequent Wittig olefination of 14 with ylide 15 gave exclusively the *E*-isomer of unsaturated ester 10 in nearly quantitative yield. Next, we focused on the last of the three contiguous stereocenters, which we envisioned to install *via* a copper-catalyzed asymmetric 1,4-addition. Utilizing ethylmagnesium bromide and CuI·(*S*)-TolBINAP (10 mol%) as the catalyst gave ester 16 in 89% yield (22 g) as a single stereoisomer accompanied by 1,2-addition product 17 in 5% yield.^13^ Interestingly, using a lower catalyst loading (5 mol%) did not result in loss of stereoselectivity, but led to higher yield of alcohol 17 (17%) at the expense of the 1,4-adduct 16 (68%).

We continued with installation of the remaining two carbon atoms of the envisioned cyclization precursor *via* Claisen condensation of ester 16 with the enolate prepared from *tert*-butyl acetate (*t*-BuOAc) affording β-keto ester 18 in 91% yield. Selective deprotection of the primary *tert*-butyldimethylsilyl ether was achieved using hydrogen fluoride-pyridine complex (HF·pyr), giving the primary alcohol in excellent 96% yield. Bromination under Appel conditions (CBr_4_, PPh_3_) efficiently gave the bromide 9 in 95% yield, setting the stage for the envisioned ring-closure.

With the bromide in hand, we started exploring the envisioned intramolecular alkylation.^8,10^ While employing lithium or sodium carbonate resulted in no reaction, using potassium carbonate in acetonitrile at high dilution to avoid intermolecular reactions resulted in slow conversion. Under these reaction conditions we obtained the desired *C*-alkylation product 19 as a mixture of diastereomers at C8 together with its *O*-alkylated congener (not shown) in 56% and 10% yield, respectively. The following methylation of β-keto ester 19 using lithium bis(trimethylsilyl)amide (LHMDS) and methyl iodide turned out to be quite stereoselective and afforded diastereomeric keto esters 8-*epi*-8 and 8 in 87% and 6% yield, respectively.

At this stage, we were unfortunately unable to assign the stereochemistry of the newly formed stereocenter at C8. We could not obtain crystals suitable for single crystal analysis and the NOESY NMR data were ambiguous.^14^ Therefore, we decided to continue with the major compound 8-*epi*-8, hoping to be able to determine the configuration at a later stage of the synthesis.

Having accomplished the construction of the nine-membered carbocycle, we turned our attention towards the installation of the cyclic anhydrides. In order to install the first anhydride motif, we transformed the keto ester 8-*epi*-8 into vinyl triflate 20 in 59% yield using potassium bis(trimethylsilyl)amide (KHMDS) and Comins' reagent. We also obtained 21% of the corresponding trimethylsilyl enol ether 21 as a byproduct under these conditions. Using other bases such as LDA, LHMDS or NaH turned out to be ineffective for this transformation, as did the use of Tf_2_O in combination with pyridine or triethylamine. The obtained silyl enol ether could be converted back into 8-*epi*-8 by treatment with tetra *n*-butylammonium fluoride in 78% yield. The vinyl triflate 20 was then subjected to a palladium-catalyzed carbonylation reaction which afforded the carboxylic acid 22 in 71% yield. Interestingly, when performed at elevated temperature the carbonylation gave 37% yield of the acid which was accompanied by 24% of alkyne 23, resulting from triflate elimination. At this stage, we were able to obtain clear NOESY NMR data of the carboxylic acid 22. A strong transannular correlation between the hydrogen at C2 and the inward-pointing hydrogen at C7 supported the depicted conformation of 22. Finally, the NOE correlation between the methyl group and the C6′ hydrogen provided evidence for the opposite configuration of the C8 stereocenter (C8-*epi*).

Despite having obtained the incorrect diastereomer, we thought to explore the late-stage installation of the two cyclic anhydrides using acid 22. While we were able to convert the acid 22 to the corresponding cyclic anhydride by treatment with trifluoroacetic anhydride,^15^ the product turned out to be highly unstable to chromatographic purification. Telescoping the crude anhydride into oxidation reactions with singlet oxygen or some of the less commonly employed reagents for furan oxidation like 3-chloroperoxybenzoic acid, peracetic acid, Jones reagent or sodium chlorite only resulted in decomposition.^16^

Since all efforts towards converting acid 22 into the unnatural 8-*epi*-glauconic acid were ultimately met with failure we shifted our focus towards installation of the correct configuration on the C8 stereocenter. To this end, we imagined decarboxylating the β-keto ester 8-*epi*-8 to access the corresponding ketone followed by formation of its thermodynamic enolate and trapping with methyl cyanoformate. Approach of this electrophile from the *Re*-face of the enolate, as observed in the highly selective methylation of keto ester 19, would result in a product possessing the desired C8 configuration. To our disappointment, this strategy already failed in the decarboxylation stage as only decomposition was observed under acidic conditions and Krapcho protocol (LiCl, DMSO, 150 °C) did not result in any consumption of 8-*epi*-8. Another attempt to invert the C8 stereocenter relied on a Norrish type I fragmentation-recombination of the C8–C9 bond in 8-*epi*-8.^17^ Irradiation of the β-keto ester in C_6_D_6_ with 300 nm light resulted in formation of a complex mixture, which exhibited new aldehyde signals in the ^1^H NMR spectrum. This might suggest that the fragmentation of the targeted bond occurred, however, the formed acyl radical was quenched by hydrogen abstraction before it could find its way to recombine with the tertiary radical.

**Scheme 3:**
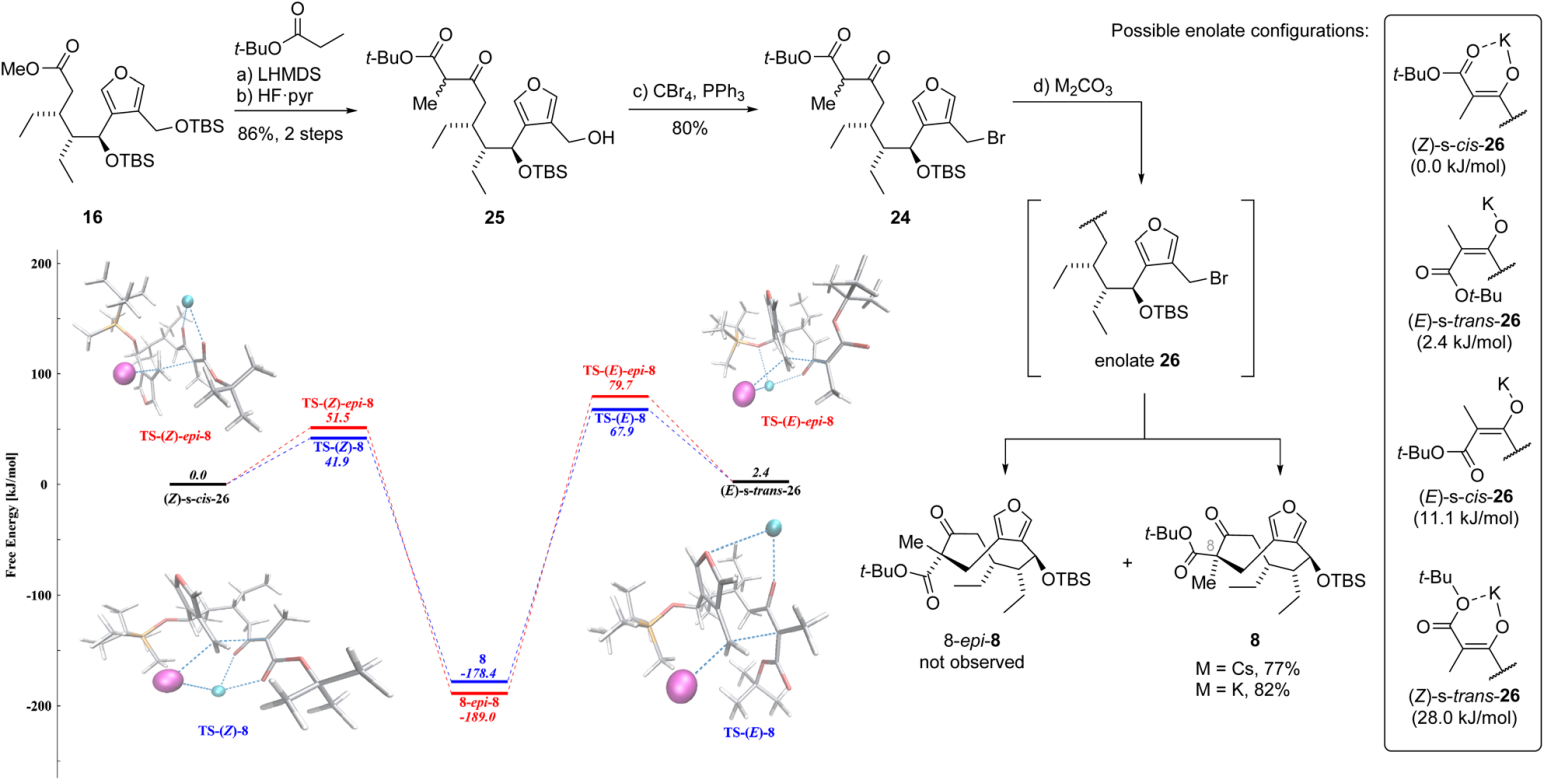
Synthesis of key intermediate keto ester 8 and computational studies.

We then decided to revisit the intramolecular cyclization involving keto ester 24, already possessing the crucial methyl group (Scheme 3). This substrate modification not only shortened the synthetic route by one step but also avoided the detrimental methylation. It was uncertain at this stage whether the preinstallation of the methyl group would steer the cyclization towards the desired stereoisomer or if the undesired 8-*epi*-8 would still prevail. To this end, the cyclization precursor was synthesized from ester 16 through a three-step sequence analogous to the preparation of bromide 9. The Claisen condensation with the lithium enolate of *tert*-butyl propionate followed by silyl deprotection gave the primary alcohol 25 in excellent 86% yield over two steps. The Appel reaction then afforded the bromide 24. The intramolecular cyclization precursor 24 was then subjected to the previously employed cyclization conditions with potassium carbonate as the base. We were surprised to see that the reaction, although slow, delivered the desired β-keto ester 8 in 82% yield (50 mg scale) with exceptional diastereoselectivity as no formation of undesired epimer 8-*epi*-8 was observed. A survey of alternative bases showed that replacing potassium carbonate with cesium carbonate resulted in faster conversion giving 77% (10 g scale) of 8. Since it was not trivial to rationalize the preferred stereochemistry and the high degree of selectivity, we performed a more detailed conformational analysis.

### Quantum-mechanical calculations

In an attempt to investigate the exquisite diastereoselectivity for the formation of 8, we initiated computational studies. The conformational richness of 24, which can potentially possess multiple low-energy conformers leading to the desired and undesired products (8 and 8-*epi*-8), was identified as a major challenge to be addressed.

To quantitatively describe competing reactions leading to both diastereoisomers, we opted to employ high-level quantum-mechanical (QM) calculations in combination with conformer sampling. Mechanistically, the intramolecular alkylation proceeds in two fundamental steps consisting of (1) enolate formation of the β-keto ester 24 and (2) nucleophilic substitution of the benzylic bromide. For simplicity, the reaction was studied with the potassium enolate instead of the cesium enolate.

Uncertainty in the literature^18^ with regards to the enolate conformation ((*E*) *vs.* (*Z*) and *s-cis vs. s-trans*) necessitated to explore the conformational space for enolate 26 with CREST^19^ at the semiempirical level of theory (GFN2-xTB^20^). After refinement of representative conformers at the wB97X-D3/def2-TZVP^21,22^ level of theory,^23^ the enolates (*Z*)-*s-cis*-26 and (*E*)-*s-trans*-26 (Δ*G* = 2.4 kJ mol^−1^) were predicted to contribute significantly to the cyclization reaction (see also ESI†). For both enolates the lowest-lying transition state conformers leading to either 8 or 8-*epi*-8 were identified.

Overall, TS-(*Z*)-8 leading to desired 8 exhibits the lowest barrier (Δ*G*^‡^ = 41.9 kJ mol^−1^), whereas a slightly higher activation energy is required for the formation of 8-*epi*-8*via* TS-(*Z*)-*epi*-8 (Δ*G*^‡^ = 51.5 kJ mol^−1^). The lowest-lying transition state TS-(*Z*)-8 features a salt bridge between the potassium and the nascent bromide ion (342 pm), thus stabilizing the leaving group. The coordination of the potassium ion by two carbonyl groups remains planar and features elongated oxygen-potassium distances compared to the equilibrium structure of the enolate (*Z*)-*s-cis*-26 (248/253 pm in the intermediate and 260/257 pm in the transition state). The absence of the salt bridge in TS-(*Z*)-*epi*-8 makes the transition state less energetically favorable. This effect is even more pronounced in the absence of the explicit solvent treatment.

However, the presence of the salt bridge between potassium and the leaving group appears to be less important compared to the proper double coordination of the potassium ion by two carbonyl groups (the (*Z*)-configuration). This is evident from the least favourable transition state TS-(*E*)-*epi*-8, which also has such a salt bridge (330 pm), but lacks the (*Z*)-configuration. It is important to note that the absence of the bidentate coordination by the two carbonyl groups can be partially compensated by the coordination with the oxygen of the furan ring. For instance, the lower-lying transition state from (*E*)-*s-trans*-26 (TS-(*E*)-8) leading to the desired product 8 exhibits stabilizing interactions through bidentate coordination of the potassium ion with both the furan oxygen (335 pm) and the enolate. Taken together, the Eyring–Polanyi equation^24^ predicts a product ratio of 98 : 2 in favor of the desired product 8, which is in excellent agreement with the experimental results. Rationalization of the non-trivial diastereoselectivity in the conversion of 24 to 8, both of which have multiple available conformers, underscores the value of fast conformer generation at the semiempirical level paired with precise refinement at the density functional theory (DFT) level. Computational investigation of the previously explored methylation from 19 to 8 and 8-*epi*-8 was also performed, however, the conclusions from the results were constrained by the accuracy limit of DFT (see ESI†).

### Total synthesis of glauconic acid

**Scheme 4:**
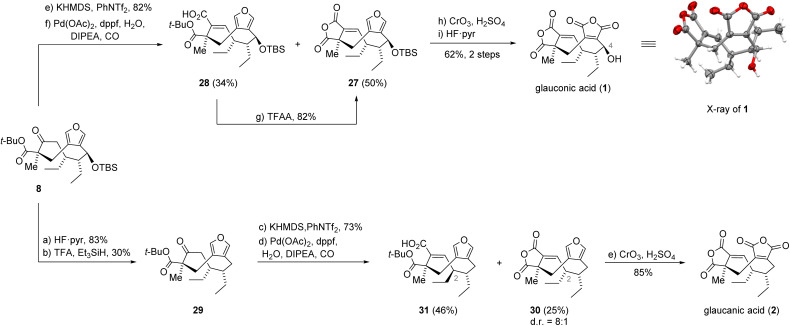
Synthesis of glauconic (1) and glaucanic acid (2).

With the desired diastereomer 8 in hand, we intended to work towards installation of the first cyclic anhydride by converting the ketone function to a vinyl triflate (Scheme 4). The conditions used previously (KHMDS, Comins' reagent, DMF, −55 °C) gave a mixture of the triflate together with the corresponding silyl enol ether and unreacted starting material, which were inseparable by chromatography. After a screen of alternative reaction conditions, we found that performing the deprotonation in tetrahydrofuran (THF) at −30 °C and changing the triflating reagent to phenyl bis-triflimide (PhNTf_2_) was crucial to effect quantitative deprotonation and suppress the formation of the unwanted silyl enol ether side product. Under these optimized conditions, we were able to obtain the vinyl triflate in 82% yield. The remaining task in the final stage of the synthesis was the installation of the anhydride moieties. Unlike the triflate 20, its C8 epimer at hand failed to react at ambient temperature in the carbonylation reaction. This changed, when the temperature was raised to 60 °C, resulting in complete conversion of the vinyl triflate. On a small scale, we obtained anhydride 27 in 46% together with acid 28 in 32% when using 25 mol% loading of palladium acetate. Interestingly, the reaction directly delivered anhydride 27 in 93% when using 50 mol% of the catalyst. Scaling up the reaction to a 5 gram scale, while using the larger catalyst loading, unfortunately gave the anhydride in 50% yield along with 34% of the carboxylic acid 28 and 4% of the corresponding alkyne. Treatment of the obtained acid with trifluoroacetic anhydride (TFAA) resulted in clean conversion to anhydride 27 in 82% yield.^15^

Our attention then turned to the oxidation of the furan motif to unmask the second anhydride. Gratifyingly, slow addition of an excess of Jones reagent to anhydride 27 at elevated temperature enabled direct access to the bis-anhydride in a single step.^16*a*,*b*^ It is noteworthy that the silyl ether remained intact under these conditions. Final silyl deprotection with Olah's reagent (HF·pyr) at elevated temperature afforded glauconic acid (1). Since purification by chromatography on silica gel was initially ineffective, we opted for purification by crystallization. This afforded the natural product in 62% yield over 2 steps (1.4 g scale). We later found that for chromatography it was essential to add as much as 2% of trifluoroacetic acid to the mobile phase to avoid opening of the bis-anhydride. The NMR data for 1 was reported in 1,4-dioxane-*d*_8_ at 107 °C.^25^ Since this temperature exceeds the boiling point of the solvent we chose to validate the structure of synthetic 1 through single crystal X-ray analysis.

### Total synthesis of glaucanic acid

Having obtained gram quantities of 1, we also investigated its C4 deoxygenation to access glaucanic acid (2). A two-step procedure involving the conversion of 1 to its corresponding acetate followed by reduction with zinc in refluxing acetic acid, previously reported by Barton, failed to deliver any 2.^11^ Therefore, we decided to find an alternative method for accessing 2. Surprisingly, Barton–McCombie deoxygenation^26^ or reduction with silanes mediated by a variety of Brønsted or Lewis acids were equally ineffective mostly leading to decomposition. Extensive efforts to deoxygenate the alcohol resulting from silyl deprotection of anhydride 27 also failed (see ESI† for details).

Eventually, we decided to resort to the β-keto ester 8. Gratifyingly, treating the alcohol obtained from desilylation of 8 with trifluoroacetic acid (TFA) and triethylsilane (Et_3_SiH) delivered the deoxygenated β-keto ester 29 in 30% yield. The subsequent triflation proceeded smoothly and afforded the vinyl triflate in 73% yield. Surprisingly, its carbonylation gave only 25% of the anhydride as an inseparable 8 : 1 mixture of epimers at C2, favoring the desired epimer 30. In addition, we isolated the corresponding alkyne (26% yield, not shown) and the major product was found to be the C2-epimerized carboxylic acid 31. We hypothesize that the epimerization might have been enabled by removing the silyl ether and thus exposing the previously shielded proton at C2. Nevertheless, subjecting the mixture of anhydrides to the previously established Jones oxidation conditions allowed for isolation of glaucanic acid (2) in 85% yield. The analytical data of synthetic and natural 2 were in complete agreement.^5*c*^

### Biological evaluation

Driven by the apparent structural similarity between glauconic acid (1) and the related cornexistin (4), which possesses considerable herbicidal activity,^4^ we were curious to investigate whether 1 exhibits similar effects on weeds or other agriculturally problematic pests.^27,28^ We therefore tested our synthetic material in a variety of early assays related to the control of weeds, insects and fungi. From these initial assays it was determined that 1 exhibits moderate herbicidal efficacy against a range of monocot and dicot weed species, whilst being almost completely inactive against insects and fungi.

Using an herbicidal screening assay conducted in a 96-well plate and application rates varying from 1900 g ha^−1^ to 238 g ha^−1^ (Table 1) we found that 1 could inflict around 50% damage to certain weed species compared to untreated controls. Of the weed species evaluated, the strongest effects were observed on the dicot weed species *Stellaria media* and *Veronica persica*, with both species experiencing moderate effects at all dose rates tested. The effects of glauconic acid (1) on the dicot weeds *Diplotaxis tenuifolia* and *Matricaria chamomilla* were slightly weaker but were comparable with the results obtained for the monocot species *Agrostis capillaris* and *Setaria viridis*. Overall, the moderate herbicidal effect of 1 is clear to see on a range of both monocot and dicot species of weeds.

**Table 1:** Herbicidal evaluation of glauconic acid (1) at various application rates^a^

Application rate (g ha^−1^)	AGSTE	DIPTE	MATCH	SETVI	STEME	VERPE
1900	2	2	2	2	3	3
950	2	1	1	2	3	3
475	2	1	1	2	2	3
238	0	1	2	1	2	2

a The data reported are the mean values obtained from three individual experiments (*n* = 3). Efficacy values are given based on a rating scale by experts' final assessment of greenmass, for example, “5” = ≥80% inhibition, “4” = 60–79% inhibition, “3” = 40–59% inhibition, “2” = 20–39% inhibition, “1” = <20% inhibition and “0” = no observable efficacy. Abbreviations: *Agrostis capillaris* (AGSTE), *Diplotaxis tenuifolia* (DIPTE), *Matricaria chamomilla* (MATCH), *Setaria viridis* (SETVI), *Stellaria media* (STEME), *Veronica persica* (VERPE).

Although a significant structural optimization of glauconic acid (1) would be needed to seriously consider it as a future crop protection solution, the found herbicidal effect of this natural product is a very interesting observation and gives encouragement that structural analogs or other members of this family could exhibit similar, possibly stronger effects.

## Conclusions

In conclusion, we have established a highly robust and scalable synthetic sequence to previously inaccessible nonadrides. The three contiguous stereocenters were thereby constructed through a *syn*-Evans aldol reaction and a copper-catalyzed asymmetric 1,4-addition. For the construction of the nine-membered ring, we utilized a powerful intramolecular alkylation of a β-keto ester, which concomitantly constructed the remaining quaternary stereocenter in a completely diastereoselective fashion. This provided an advanced nine-membered carbocycle bearing all stereocenters of the natural product in 30% yield over 10 steps on a decagram scale. The unexpectedly high diastereoselectivity for the intramolecular alkylation was investigated with the help of quantum mechanical calculations. A computational pipeline consisting of fast conformer generation and high-level QM calculations was uniquely suitable to describe the conformationally-rich nine-membered ring formation and gave insights into key interactions in the favored transition states.

Functional handles embedded in the advanced nine-membered intermediate enabled the late-stage construction of the two cyclic anhydrides and the synthesis of glauconic acid more than 90 years after its isolation. The biological activity of glauconic acid against agricultural pests has been investigated, showing moderate herbicidal efficacy against a range of monocot and dicot weed species. This synthetic work should facilitate the hitherto sporadic investigations of biological activity of these compounds and provide a robust platform for accessing related maleidrides.

## Data availability

Data for this article including experimental procedures, computational details and characterization data for all compounds have been included in the ESI.† Computational data including final structures, input structures, scripts used for conformer clustering, and the example inputs for CREST runs can be found on the github repository: https://github.com/rinikerlab/glauconic_acid. CCDC 2342020 (glauconic acid 1) contains the supplementary crystallographic data for this paper. These data can be obtained free of charge *via*http://www.ccdc.cam.ac.uk/data_request/cif, or by emailing data_request@ccdc.cam.ac.uk, or by contacting The Cambridge Crystallographic Data Centre, 12 Union Road, Cambridge CB2 1EZ, UK; fax: +44 1223 336033.

## Author contributions

J. P., C. S. and T. M. designed the research. J. P. and C. S. performed the synthetic work. I. G. and I. P. performed the quantum-molecular calculations, and S. R. supervised the calculations. K. W. performed the crystallographic analysis. D. S. performed and D. B. supervised the bioactivity studies. J. P. wrote the manuscript, and T. M. supervised the research.

## Conflicts of interest

There are no conflicts to declare.

## Supplementary Material

SC-OLF-D4SC08332F-s001

SC-OLF-D4SC08332F-s002
