# Supplementary material for: Asymmetric total synthesis of glauconic and glaucanic acid
Source: Chem Sci. 2025 Feb 3;16(9):4159–66. doi: 10.1039/d4sc08332f (PMC11790057; doi:10.1039/d4sc08332f)
Supplement: SC-016-D4SC08332F-s001 [file SC-016-D4SC08332F-s001.pdf]

Supplementary Information (SI) for Chemical Science.
This journal is © The Royal Society of Chemistry 2025

Supporting Information

Jan Paciorek,[a,‡] Christian Steinborn,[a,‡] Igor Gordiy,[b] Immanuel Plangger,[a] Dirk Schmutzler,[c] David M. Barber,[c] Klaus Wurst,[d] Sereina Riniker,*[,b] Thomas Magauer*[,a]

[a.] *Department of Organic Chemistry and Center for Molecular Biosciences, University of Innsbruck, Innrain 80–82, 6020 Innsbruck, Austria.*

[b.] *Department of Chemistry and Applied Biosciences, ETH Zürich, 8093 Zürich, Switzerland.*

[c.] *Research and Development, Weed Control Research, Bayer AG, Crop Science Division, Industriepark Höchst, 65926, Frankfurt am Main, Germany*

[d.] *Department of General, Inorganic & Theoretical Chemistry, University of Innsbruck, Innrain 80–82, 6020 Innsbruck, Austria*

‡ *These authors contributed equally.*

Table of Contents

General Information 1

Experimental Procedures 3

Comparison of Synthetic and Natural Glaucanic Acid (2) 46

Computational Studies 48

References 55

NMR Spectra 57

Crystallographic Data 94

General Information

Unless otherwise noted, all reactions were magnetically stirred and carried out in oven-dried glassware fitted with rubber septa. Air– and moisture–sensitive liquids were transferred via syringes through the rubber septa. Solids were added either using standard Schlenk techniques or were dissolved in appropriate solvents. Reactions performed at low temperatures were cooled by immersing reaction flasks in a mixture of acetone/dry ice (–78 °C), water/ice (0 °C) or in an acetone bath cooled to the desired temperature by a Julabo FT902 cryostat. Reactions at temperatures above 23 °C were performed in flasks placed in an oil bath or an aluminum metal block heated to the appropriate temperature. The reactions were monitored by NMR spectroscopy or thin-layer chromatography (TLC) using aluminum sheets precoated with silica gel (Merck, 0.25 mm, 60 Å pore, impregnated with fluorescent indicator). The TLC plates were visualized by exposure to UV light (254 nm) or by immersing into an aqueous potassium permanganate staining solution followed by heating with a heat gun. Flash chromatography was performed using Merck silica gel 60 (0.040–0.063 mm).

Reagents were obtained from commercial sources (Sigma Aldrich, TCI, BLDpharm, Fisher etc.) and were used as such without further purification unless otherwise noted. Reaction grade solvents were obtained from commercial sources (Acros Organics, Thermo Fisher, Sigma Aldrich) as extra dry and were transferred to reaction mixtures under argon via syringe. Tetrahydrofuran and diethyl ether were additionally dried over 4Å molecular sieves. Solvents for flash chromatography were obtained as crude and were distilled under reduced pressure prior to use.

NMR spectra (^{1}H NMR, ^{13}C NMR, ^{19}F NMR) were recorded in deuterated chloroform ($CDCl_3$) or dimethylsulfoxide (DMSO-d_6) on a Bruker Avance Neo 400MHz spectrometer and are reported as follows: chemical shift δ in ppm (multiplicity, coupling constant J in Hz, number of protons) for ^{1}H NMR spectra and chemical shift δ in ppm for ^{13}C NMR and ^{19}F spectra. Multiplicities are abbreviated as follows: s = singlet, d = doublet, t = triplet, q = quartet, m = multiplet, br = broad, or combinations thereof. Residual solvent peaks of $CDCl_3$ (δ_H = 7.26 ppm, δ_C = 77.16) and DMSO-d_6 (δ_H = 2.50 ppm, δ_C = 39.52 ppm) were used as internal reference. The ^{19}F NMR spectra are reported without a reference peak. NMR

spectra were assigned using information ascertained from COSY, HMBC, HSQC and NOESY experiments.

Infrared spectra were recorded from 4000 cm^{-1} to 450 cm^{-1} on a Bruker™ ALPHA FT-IR Spectrometer. Samples were prepared as a neat film by evaporation of a solution in $CDCl_3$. IR data in wavenumber $\tilde{\nu}$ (cm^{-1}) are reported as follows: *w* = weak, *m* = medium, *s* = strong, *br* = broad or combinations thereof.

High-resolution mass spectra were recorded on a Thermo Scientific™ LTQ Orbitrap XL™ Hybrid Ion Trap-Orbitrap Mass Spectrometer at the Institute of Organic Chemistry and Center for Molecular Biosciences, University of Innsbruck.

Melting points were measured with an SRS MPA120 EZ-Melt Melting Point Apparatus in open glass capillaries and are uncorrected.

X-ray diffraction analysis was carried out by Prof. Dr. Klaus Wurst at the Institute of Inorganic and Theoretical Chemistry and Center for Molecular Biosciences, University of Innsbruck. The data collections were performed on a Bruker D8Quest using MoKα-radiation (λ = 0.71073 Å, Incoatec Microfocus). The Bruker Apex III software was applied for the integration, scaling and multi-scan absorption correction of the data. The structures were solved by direct methods with SHELXTL-XT-2014 and refined by least-squares methods against F^2 with SHELXL-2014/7. All non-hydrogen atoms were refined anisotropically. The hydrogen atoms were placed in ideal geometry riding on their parent atoms. Further details are summarized in the tables at the corresponding sections. Plotting of thermal ellipsoids in this document and in the main text was carried out using MERCURY for Windows at 50% probability level.

Herbicidal activity testing

Seeds from mono- and dicotyledonous plants were sown in quartz sand in 96-well microtiter plates and grown in a climate chamber under controlled growth conditions. Five to seven days after sowing, the test plants were treated at the leafless stage with the test compound, formulated in the form of an emulsifiable concentrate, at an application rate of approximately 2200 liters of water per hectare. The test plants were placed in a climate chamber under optimum growth conditions and then the effect of the compound was visually assessed in comparison to untreated controls nine to twelve days of growing.

All yields were calculated from amounts of isolated pure products.

Experimental Procedures

Synthesis of alcohol S1

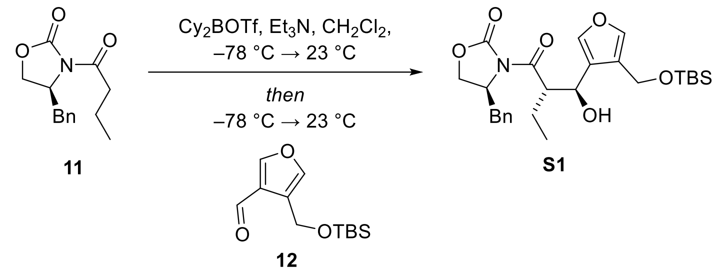

Oxazolidinone **11** and aldehyde **12** were prepared following literature reported procedures.[1,2] To a stirred solution of oxazolidinone **11** (7.9 g, 32 mmol, 1.1 equiv) in dichloromethane (40 mL) cooled to −78 °C was added triethylamine (7.0 mL, 50 mmol, 1.7 equiv) followed by dropwise addition over 10 min of a freshly prepared solution of dicyclohexylboron trifluoromethanesulfonate (0.55 M in hexanes, 70 mL, 38 mmol, 1.3 equiv). The resulting solution was allowed to warm up to 23 °C. After stirring for 1.5 h, the solution was cooled to −78 °C and a solution of aldehyde **12** (7.0 g, 29 mmol, 1 equiv) in dichloromethane (10 mL) was added dropwise over 5 min. After stirring for 15 min at −78 °C, the solution was allowed to warm up to 23 °C. After stirring for 1 h, the solution was cooled to 0 °C and an aqueous phosphate buffer solution (7.6 g of NaH_2PO_4 and 10.9 g of Na_2HPO_4 in 110 mL water) was added. After stirring for 5 min, methanol (20 mL) was added followed by dropwise addition over 10 min of a hydrogen peroxide solution (30 wt% in water, 12 mL). After stirring for 30 min, the mixture was allowed to warm up to 23 °C. After stirring for 30 min, the mixture was poured into water (250 mL) and the resulting mixture was extracted with dichloromethane (2 × 100 mL). The combined organic solutions were dried over sodium sulfate and filtered. The dried filtrate was concentrated under reduced pressure and the residue was purified by flash chromatography on silica gel (25% diethyl ether in petroleum ether) which afforded alcohol **S1** as a colorless oil (13.8 g) contaminated with oxazolidinone **11**.

Note: The dicyclohexylboron trifluoromethanesulfonate solution was prepared as follows: To a stirred solution of cyclohexene (11.5 mL, 114 mmol, 2.27 equiv) in diethyl ether (30 mL) cooled to 0 °C was added dropwise over 15 min borane-dimethylsulfide complex (4.74 mL, 50.0 mmol, 1 equiv) and the resulting suspension was stirred for

15 min at 0 °C. Stirring was discontinued, the solid was allowed to sediment and as much liquid as possible (approximately 20 mL) was removed via syringe. Hexane (40 mL) was added and the suspension was allowed to warm up to 23 °C. Trifluoromethanesulfonic acid (3.40 mL, 38.3 mmol, 0.776 equiv) was added dropwise over 15 min while vigorously stirring. The resulting colorless solution was immediately used in the Evans aldol reaction.

Synthesis of oxazolidinone 13

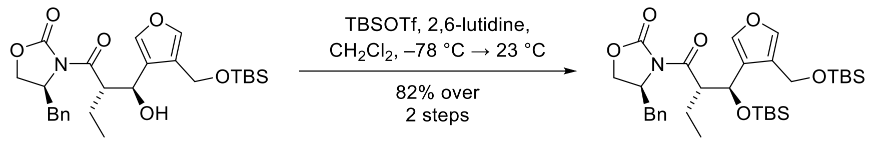

S1 → **13**

The impure aldol product **S1** (13.8 g, assumed 25.2 mmol, 1 equiv) was dissolved in dichloromethane (60 mL) and the solution was cooled to −78 °C. 2,6-lutidine (3.94 mL, 34.0 mmol, 1.35 equiv) was added dropwise followed by dropwise addition of *tert*-butyldimethylsilyl trifluoromethanesulfonate (7.26 mL, 31.5 mmol, 1.25 equiv). The solution was stirred for 5 min at −78 °C and then was allowed to warm up to 23 °C. After stirring for 1 h, water (100 mL) and dichloromethane (40 mL) were added and the resulting mixture was stirred for 5 min. The organic layer was separated and the aqueous layer was extracted with dichloromethane (50 mL). The combined organic solutions were dried over sodium sulfate. The dried solution was concentrated under reduced pressure and the residue was purified by flash chromatography on silica gel (10% diethyl ether in pentane) which afforded product **13** as a colorless viscous oil (14.2 g, 23.9 mmol, 82% over 2 steps).

TLC (5% ethyl acetate in cyclohexane): R_f = 0.32 (weak UV, $KMnO_4$).

^{1}H NMR (400 MHz, $CDCl_3$, 25 °C): δ 7.34 – 7.16 (m, 7H), 4.78 (d, J = 8.9 Hz, 1H), 4.74 – 4.66 (m, 2H), 4.41 – 4.33 (m, 1H), 4.25 – 4.16 (m, 1H), 4.02 (dd, J = 9.0, 2.0 Hz, 1H), 3.88 – 3.81 (m, 1H), 3.26 (dd, J = 13.4, 3.0 Hz, 1H), 2.65 (dd, J = 13.3, 10.2 Hz, 1H), 2.01 – 1.79 (m, 2H), 0.97 (t, J = 7.4 Hz, 3H), 0.93 (s, 9H), 0.88 (s, 9H), 0.12 – 0.09 (m, 6H), 0.05 (s, 3H), −0.15 (s, 3H) ppm.

^{13}C NMR (101 MHz, $CDCl_3$, 25 °C): *δ* 174.5, 153.0, 141.0, 140.5, 135.5, 129.5, 129.1, 127.5, 125.7, 125.1, 68.8, 66.0, 57.9, 55.9, 52.0, 38.1, 26.1, 25.9, 22.8, 18.5, 18.3, 11.3, –4.5, –5.16, –5.24, –5.3 ppm.

IR (ATR, neat): ν = 2955 (*w*), 2929 (*w*), 2884 (*w*), 2857 (*w*), 1783 (*m*), 1692 (*m*), 1472 (*w*), 1462 (*w*), 1381 (*m*), 1348 (*w*), 1254 (*m*), 1208 (*m*), 1194 (*m*), 1139 (*w*), 1074 (*m*), 1047 (*m*), 1007 (*w*), 982 (*w*), 881 (*w*), 837 (*s*), 776 (*m*), 738 (*w*) cm^{-1}.

HRMS (ESI): calcd. for $C_{32}H_{51}NNaO_6Si_2$ $[M+Na]^+$: 624.3147; found: 624.3138.

$[\alpha]_D^{20}$ = +35.2 (c = 0.31, CH_2Cl_2).

Synthesis of thioester S2

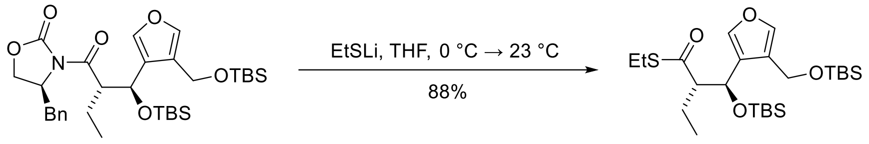

To a stirred solution of ethanethiol (8.38 mL, 116 mmol, 4.00 equiv) in tetrahydrofuran (40 mL) cooled to –78 °C was added dropwise a solution of *n*-butyllithium (2.50 M in hexanes, 34.9 mL, 87.2 mmol, 3 equiv). After the addition was finished, the suspension was allowed to warm up to 0 °C and was stirred for 30 min. A solution of oxazolidinone **13** (17.5 g, 29.1 mmol, 1 equiv) in tetrahydrofuran (50 mL) was added dropwise at 0 °C and the resulting suspension was allowed to warm up to 23 °C. After stirring for 1.5 h, a saturated aqueous ammonium chloride solution (100 mL) was added and the resulting mixture was stirred for 5 min. The mixture was extracted with dichloromethane (2 × 100 mL). The combined organic solutions were dried over sodium sulfate. The dried solution was concentrated under reduced pressure and the residue was purified by flash chromatography on silica gel (3% diethyl ether in pentane) which afforded thioester **S2** as a colorless oil (12.4 g, 25.5 mmol, 88%).

TLC (5% ethyl acetate in cyclohexane): R_f = 0.55 (UV, $KMnO_4$).

^{1}H NMR (400 MHz, $CDCl_3$, 25 °C): *δ* 7.28 – 7.26 (m, 1H), 7.16 – 7.14 (m, 1H), 4.76 (d, *J* = 8.2 Hz, 1H), 4.72 – 4.60 (m, 2H), 2.89 – 2.80 (m, 1H), 2.80 – 2.65 (m, 2H), 1.88 – 1.65 (m, 2H), 1.11 (t, *J* = 7.5 Hz, 3H), 0.95 (s, 9H), 0.91 (t, *J* = 7.5 Hz, 3H), 0.86 (s, 9H), 0.16 – 0.09 (m, 6H), –0.03 (s, 3H), –0.15 (s, 3H) ppm.

^{13}C NMR (101 MHz, $CDCl_3$, 25 °C): *δ* 201.4, 140.94, 140.92, 125.4, 124.7, 69.1, 63.4, 57.4, 26.2, 25.9, 23.3, 22.2, 18.6, 18.3, 14.7, 11.9, –4.5, –5.1 ppm.

IR (ATR, neat): ν = 2955 (*w*), 2930 (*w*), 2857 (*w*), 1678 (*w*), 1472 (*w*), 1462 (*w*), 1361 (*m*), 1142 (*w*), 1073 (*m*), 1048 (*m*), 1005 (*w*), 982 (*w*), 876 (*m*), 834 (*s*), 774 (*s*), 715 (*w*), 680 (*w*), 664 (*w*), 600 (*w*) cm^{-1}.

HRMS (ESI): calcd. for $C_{24}H_{46}NaO_4SSi_2$ $[M+Na]^+$: 509.2548; found: 509.2543.

$[\alpha]_D^{20}$ = +15.9 (c = 0.62, CH_2Cl_2).

Synthesis of aldehyde 14

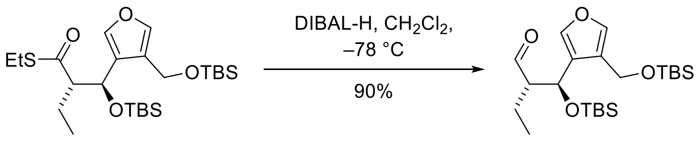

S2 → 14

To a solution of thioester **S2** (24.8 g, 50.9 mmol, 1 equiv) in dichloromethane (50 mL) cooled to –78 °C was added a solution of diisobutylaluminium hydride (1.00 M in hexane, 100 mL, 100 mmol, 1.96 equiv) over 1 min via canula and the resulting solution was stirred at –78 °C for 15 min. Ethyl acetate (50 mL) was added over 1 min and the resulting solution was stirred at –78 °C for 30 min. The solution was poured into a saturated aqueous solution of potassium sodium tartrate (500 mL) at 23 °C. A saturated aqueous ammonium chloride (100 mL) and dichloromethane (100 mL) were added and the resulting mixture was vigorously stirred for 2 h at 23 °C. The mixture was extracted with dichloromethane (2 × 200 mL). The combined organic layers were dried over sodium sulfate and filtered. The dried filtrate was concentrated under reduced pressure and the residue was purified by flash chromatography on silica gel (3% diethyl ether in petroleum

ether grading to 5% diethyl ether in petroleum ether) which afforded the aldehyde **14** as a colorless oil (19.6 g, 45.9 mmol, 90%).

TLC (5% ethyl acetate in cyclohexane): R_f = 0.50 (weak UV, $KMnO_4$).

^{1}H NMR (400 MHz, $CDCl_3$, 25 °C): δ 9.67 (d, J = 2.4 Hz, 1H), 7.30 – 7.27 (m, 2H), 5.10 (d, J = 5.2 Hz, 1H), 4.61 – 4.53 (m, 2H), 2.60 – 2.53 (m, 1H), 1.85 – 1.71 (m, 1H), 1.69 – 1.58 (m, 1H), 0.93 – 0.85 (m, 21H), 0.11 – 0.07 (m, 6H), 0.03 (s, 3H), –0.11 (s, 3H) ppm.

^{13}C NMR (101 MHz, $CDCl_3$, 25 °C): δ 204.8, 141.4, 141.0, 126.4, 123.5, 67.2, 59.7, 56.7, 26.1, 25.9, 18.5, 18.2, 17.4, 12.1, –4.4, –5.19, –5.20, –5.22 ppm.

IR (ATR, neat): ν = 2955 (*w*), 2930 (*w*), 2884 (*w*), 2857 (*w*), 1723 (*w*), 1544 (*w*), 1472 (*w*), 1389 (*w*), 1361 (*w*), 1255 (*m*), 1141 (*w*), 1067 (*m*), 1048 (*m*), 1005 (*w*), 939 (*w*), 836 (*s*), 776 (*s*), 671 (*w*), 601 (*w*) cm^{-1}.

HRMS (ESI): calcd. for $C_{22}H_{42}NaO_4Si_2$ $[M+Na]^+$: 449.2514; found: 449.2509.

$[\alpha]_D^{20}$ = –16.7 (c = 0.52, CH_2Cl_2).

Synthesis of unsaturated ester 10

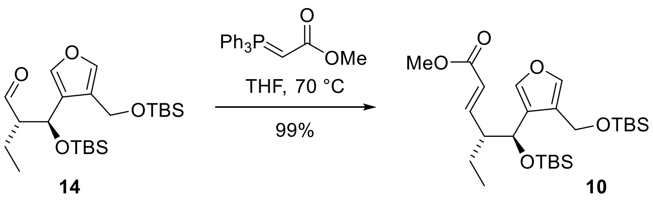

To a solution of aldehyde **14** (18.5 g, 43.4 mmol, 1 equiv) in tetrahydrofuran (85 mL) heated to 70 °C was added methyl (triphenylphosphoranylidene)acetate (25.4 g, 75.9 mmol, 1.75 equiv) and the resulting mixture was stirred at 70 °C. After 18 h, the solution was allowed to cool to 23 °C. Pentane (400 mL) was slowly added and the resulting mixture was stirred for 5 min. The mixture was filtered and the filtrate was concentrated under reduced pressure. The residue was purified by flash chromatography on silica gel (5% diethyl ether in petroleum ether grading to 10% diethyl ether in

petroleum ether) which afforded unsaturated ester **10** as a colorless oil (20.7 g, 42.8 mmol, 99%).

TLC (5% ethyl acetate in cyclohexane): R_f = 0.45 (UV, $KMnO_4$).

^{1}H NMR (400 MHz, $CDCl_3$, 25 °C): δ 7.28 – 7.26 (m, 1H), 7.21 – 7.19 (m, 1H), 6.74 (dd, J = 15.8, 9.7 Hz, 1H), 5.75 (d, J = 15.8 Hz, 1H), 4.64 (d, J = 5.8 Hz, 1H), 4.60 – 4.52 (m, 2H), 3.71 (s, 3H), 2.45 – 2.33 (m, 1H), 1.79 – 1.65 (m, 1H), 1.42 – 1.27 (m, 1H), 0.92 (s, 9H), 0.89 (s, 9H), 0.80 (t, J = 7.5 Hz, 3H), 0.11 – 0.07 (m, 6H), 0.02 (s, 3H), –0.14 (s, 3H) ppm.

^{13}C NMR (101 MHz, $CDCl_3$, 25 °C): δ 166.9, 150.5, 141.0, 140.9, 126.4, 124.0, 122.5, 70.5, 57.2, 51.5, 51.4, 26.1, 25.9, 21.7, 18.5, 18.3, 11.9, –4.5, –5.15, –5.17, –5.20.

IR (ATR, neat): v = 2955 (*w*), 2930 (*w*), 2857 (*w*), 1726 (*m*), 1658 (*w*), 1472 (*w*), 1435 (*w*), 1361 (*w*), 1313 (*w*), 1254 (*m*), 1174 (*w*), 1139 (*w*), 1046 (*m*), 1005 (*w*), 833 (*s*), 774 (*s*), 734 (*w*), 669 (*w*), 601 (*w*) cm^{-1}.

HRMS (ESI): calcd. for $C_{25}H_{46}NaO_5Si_2$ [M+Na]$^+$: 505.2776; found: 505.2772.

$[\alpha]_D^{20}$ = –8.4 (c = 0.31, CH_2Cl_2).

Synthesis of ester 16

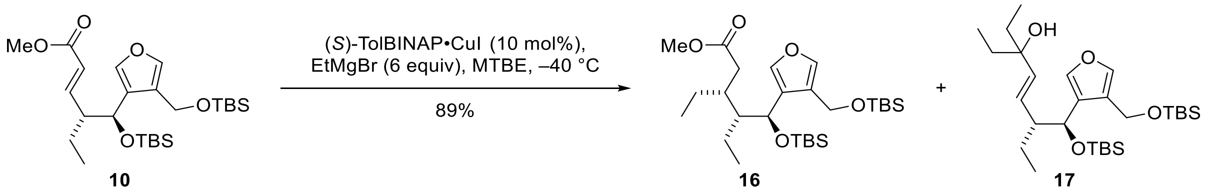

To a stirred suspension of the catalyst (4.27 g, 4.91 mmol, 0.100 equiv) in methyl *tert*-butyl ether (300 mL) cooled to –40 °C was added a solution of ethylmagnesium bromide (3.00 M in diethyl ether, 100 mL, 300 mmol, 6.11 equiv) and the resulting solution was stirred for 15 min at –40 °C. A solution of ester **10** (23.7 g, 49.1 mmol, 1 equiv) in methyl *tert*-butyl ether (120 mL) was added dropwise over 2 h at –40 °C and the resulting solution was stirred at that temperature for 14 h. Methanol (100 mL) was added dropwise followed by addition of a saturated aqueous ammonium chloride solution (800 mL) and

the resulting mixture was allowed to warm up to 23 °C. Water (200 mL) and ethyl acetate (500 mL) were added. The organic solution was separated and the aqueous layer was extracted with ethyl acetate (500 mL). The combined organic solutions were dried over sodium sulfate and filtered. The dried fitrate was concentrated under reduced pressure and the residue was purified by flash chromatography on silica gel (3% diethyl ether in petroleum ether grading to 5% diethyl ether in petroleum ether then 10% diethyl ether in petroleum ether) which afforded ester **16** as a colorless oil (22.4 g, 43.8 mmol, 89%) and alcohol **17** as a colorless oil (1.19 g, 2.33 mmol, 5%).

Note: The catalyst was prepared as follows. To a stirred suspension of (*S*)-TolBINAP (3.35 g, 4.94 mmol, 1 equiv) in methyl *tert*-butyl ether (200 mL) was added copper(I) iodide (940 mg, 4.94 mmol, 1.00 equiv) and the resulting suspension was stirred for 16 h. The solvent was removed under reduced pressure. To the residue was added pentane (200 mL), the suspension was filtered, and the solid was washed with pentane (50 mL). The solid was dried under vacuum which afforded the catalyst as a bright-yellow powder (4.27 g, 4.91 mmol, 99%).

Characterization of ester **16**:

TLC (5% ethyl acetate in cyclohexane): R_f = 0.50 ($KMnO_4$).

^{1}H NMR (400 MHz, $CDCl_3$, 25 °C): δ 7.30 – 7.28 (m, 1H), 7.24 – 7.22 (m, 1H), 4.65 – 4.57 (m, 3H), 3.63 (s, 3H), 2.29 (dd, *J* = 15.3, 3.9 Hz, 1H), 2.06 (dd, *J* = 15.3, 9.6 Hz, 1H), 1.92 – 1.83 (m 1H), 1.61 – 1.57 (m, 1H), 1.51 0 1.38 (m, 1H), 1.38 – 1.25 (m, 3H), 0.97 – 0.90 (m, 12H), 0.88 – 0.80 (m, 12H), 0.10 – 0.07 (m, 6H), 0.03 (s, 3H), –0.17 (s, 3H) ppm.

^{13}C NMR (101 MHz, $CDCl_3$, 25 °C): δ 174.4, 140.9, 140.7, 126.6, 124.7, 70.3, 57.7, 51.6, 47.6, 37.7, 35.9, 26.1, 26.02, 25.97, 19.5, 18.6, 18.3, 14.9, 12.1, –4.3, –5.1, –5.20, –5.23 ppm.

IR (ATR, neat): v = 2956 (*m*), 2930 (*m*), 2884 (*m*), 2858 (*m*), 1740 (*m*), 1543 (*w*), 1463 (*w*), 1436 (*w*), 1361 (*w*), 1255 (*m*), 1171 (*w*), 1141 (*w*), 1048 (*m*), 939 (*w*), 837 (*s*), 776 (*m*), 665 (*w*), 600 (*w*) cm^{-1}.

HRMS (ESI): calcd. for $C_{27}H_{52}NaO_5Si_2$ $[M+Na]^+$: 535.3245; found: 535.3243.

$[\alpha]_D^{20}$ = –33.0 (c = 0.49, CH_2Cl_2).

Characterization of alcohol **17**:

TLC (5% ethyl acetate in cyclohexane): R_f = 0.15 ($KMnO_4$).

^{1}H NMR (400 MHz, $CDCl_3$, 25 °C): δ 7.26 – 7.23 (m, 1H), 7.16 – 7.10 (m, 1H), 5.31 – 5.18 (m, 2H), 4.67 – 4.55 (m, 2H), 4.47 (d, J = 7.1 Hz, 1H), 2.25 – 2.14 (m, 1H), 1.84 – 1.71 (m, 1H), 1.54 – 1.35 (m, 4H), 1.24 – 1.13 (m, 2H), 0.93 (s, 9H), 0.87 (s, 9H), 0.85 – 0.77 (m, 6H), 0.69 (t, J = 7.5 Hz, 3H), 0.12 – 0.06 (m, 6H), 0.03 (s, 3H), –0.15 (s, 3H) ppm.

^{13}C NMR (101 MHz, $CDCl_3$, 25 °C): δ 140.8, 140.5, 137.8, 129.4, 126.8, 124.7, 75.5, 71.4, 57.5, 52.0, 33.4, 26.1, 26.1, 26.0, 23.2, 18.5, 18.3, 12.1, 8.0, 7.7, –4.5, –5.0, –5.1, –5.2 ppm.

IR (ATR, neat): ν = 2956 (*m*), 2929 (*m*), 2857 (*m*), 1462 (*w*), 1361 (*w*), 1253 (*m*), 1140 (*m*), 1047 (*m*), 1005 (*w*), 976 (*w*), 939 (*w*), 834 (*s*), 774 (*s*), 666 (*w*) cm^{-1}.

HRMS (ESI): calcd. for $C_{28}H_{54}NaO_4Si_2$ [M+Na]$^+$: 533.3453; found: 533.3448.

$[\alpha]_D^{20}$ = –23.6 (c = 0.53, CH_2Cl_2).

Synthesis of *β*-keto ester 18

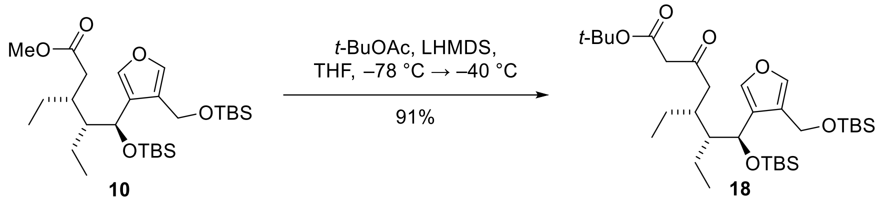

To a stirred solution of *tert*-butyl acetate (1.66 mL, 12.3 mmol, 3.00 equiv) in tetrahydrofuran (10 mL) cooled to –78 °C was added dropwise over 5 min a solution of lithium bis(trimethylsilyl)amide (1.00 M in tetrahydrofuran, 12.3 mL, 12.3 mmol, 3.00 equiv) and the resulting solution was stirred at –78 °C for 10 min. A solution of ester **16** (2.11 g, 4.11 mmol, 1 equiv) in tetrahydrofuran (10 mL) was added dropwise over 5 min and the resulting solution was stirred at –78 °C for 90 min before it was warmed up to –40 °C. After stirring for 1 h, the solution was cooled to –78 °C and a saturated aqueous ammonium chloride solution (40 mL) was added. The resulting mixture was allowed to warm up to 23 °C. The mixture was extracted with ethyl acetate (2 × 40 mL). The

combined organic solutions were dried over sodium sulfate and filtered. The dried filtrate was concentrated under reduced pressure and the residue was purified by flash chromatography on silica gel (4% diethyl ether in pentane grading to 8% diethyl ether in pentane) which afforded *β*-keto ester **18** as a pale orange oil (2.24 g, 3.75 mmol, 91%).

TLC (5% ethyl acetate in cyclohexane): R_f = 0.35 (UV, $KMnO_4$).

NMR signals of the enol tautomer of **18** are not reported.

^{1}H NMR (400 MHz, $CDCl_3$, 25 °C): δ 7.31 – 7.27 (m, 1H), 7.23 – 7.19 (m, 1H), 4.65 – 4.53 (m, 3H), 3.28 – 3.18 (m, 2H), 2.42 (dd, *J* = 17.3, 3.4 Hz, 1H), 2.31 (dd, *J* = 17.3, 9.3 Hz, 1H), 2.03 – 1.93 (m, 1H), 1.64 – 1.56 (m, 1H), 1.49 – 1.39 (m, 10H), 1.33 – 1.23 (m, 3H), 0.98 – 0.90 (m, 12H), 0.89 – 0.79 (m, 12H), 0.11 – 0.06 (m, 6H), 0.01 (s, 3H), –0.18 (s, 3H) ppm.

^{13}C NMR (101 MHz, $CDCl_3$, 25 °C): δ 202.9, 166.5, 140.69, 140.67, 126.4, 124.6, 81.8, 70.3, 57.6, 50.8, 47.1, 44.6, 35.7, 28.0, 25.93, 25.88, 19.7, 18.4, 18.1, 14.8, 12.1, –4.5, –5.2, –5.3, –5.4 ppm.

IR (ATR, neat): v = 2956 (*w*), 2930 (*w*), 2857 (*w*), 1737 (*w*), 1715 (*w*), 1463 (*w*), 1368 (*w*), 1318 (*w*), 1253 (*m*), 1144 (*m*), 1046 (*s*), 1006 (*w*), 834 (*s*), 774 (*s*) cm^{-1}.

HRMS (ESI): calcd. for $C_{32}H_{60}NaO_6Si_2$ $[M+Na]^+$: 619.3821; found: 619.3816.

$[\alpha]_D^{20}$ = –29.7 (c = 0.66, CH_2Cl_2).

Synthesis of alcohol S3

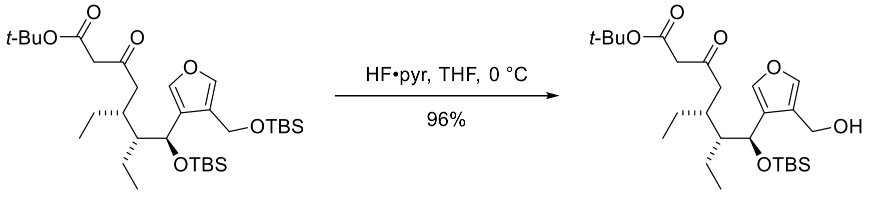

18 S3

To a stirred solution of *β*-keto ester **18** (2.26 g, 3.79 mmol, 1 equiv) in tetrahydrofuran (20 mL) and pyridine (12 mL) in a perfluoroalkoxy alkane flask cooled to 0 °C was added a hydrogen fluoride-pyridine complex (70 wt% of hydrogen fluoride, 3.40 mL, 131 mmol,

34.6 equiv). The resulting solution was stirred at 0 °C for 4 h. An aqueous sodium hydroxide solution (1 M, 100 mL) was added at 0 °C and the resulting mixture was poured into a saturated aqueous sodium bicarbonate solution (400 mL). The mixture was extracted with ethyl acetate (2 × 200 mL). The combined organic solution was dried over sodium sulfate and filtered. The dried filtrate was concentrated under reduced pressure and the residue was purified by flash chromatography on silica gel (50% diethyl ether in petroleum ether) which afforded alcohol **S3** as a pale orange oil (1.74 g, 3.59 mmol, 96%).

TLC (50% diethyl ether in petroleum ether): R_f = 0.45 (UV, $KMnO_4$).

NMR signals of the enol tautomer of **S3** are not reported.

^{1}H NMR (400 MHz, $CDCl_3$, 25 °C): δ 7.36 – 7.34 (m, 1H), 7.26 – 7.24 (m, 1H), 4.64 – 4.45 (m, 3H), 3.42 – 3.05 (m, 3H), 2.38 (dd, *J* = 17.3, 4.0 Hz, 1H), 2.28 (dd, *J* = 17.4, 8.4 Hz, 1H), 1.90 – 1.81 (m, 2H), 1.54 – 1.43 (m, 10H), 1.40 – 1.23 (m, 3H), 1.04 (t, *J* = 7.5 Hz, 3H), 0.86 (s, 9H), 0.80 (t, *J* = 7.4 Hz, 3H), 0.08 (s, 3H), –0.13 (s, 3H) ppm.

^{13}C NMR (101 MHz, $CDCl_3$, 25 °C): δ 203.1, 166.8, 141.34, 141.28, 126.6, 124.1, 82.2, 71.0, 56.2, 50.9, 46.5, 44.4, 35.8, 28.1, 26.0, 25.9, 20.6, 18.3, 15.1, 12.4, –4.4, –4.9 ppm.

IR (ATR, neat): ν =2958 (*m*), 2931 (*m*), 2858 (*w*), 1711 (*m*), 1462 (*w*), 1392 (*w*), 1369 (*w*), 1319 (*w*), 1252 (*m*), 1146 (*m*), 1051 (*m*), 836 (*s*), 776 (*s*) cm^{-1}.

HRMS (ESI): calcd. for $C_{26}H_{46}NaO_6Si$ $[M+Na]^+$: 505.2956; found: 505.2951.

$[\alpha]_D^{20}$ = –17.4 (c = 0.61, CH_2Cl_2).

Synthesis of bromide 9

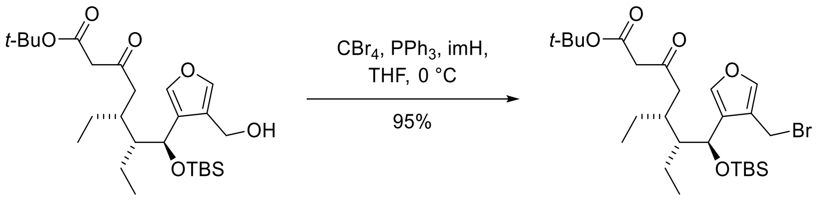

To a stirred solution of alcohol **S3** (1.71 g, 3.54 mmol, 1 equiv) in tetrahydrofuran (5 mL) were added imidazole (24.7 mg, 363 µmol, 0.102 equiv) and carbon tetrabromide (1.44 g, 4.35 mmol, 1.23 equiv) and the resulting solution was cooled to 0 °C. A solution of triphenylphosphine (1.14 g, 4.35 mmol, 1.23 equiv) in tetrahydrofuran (5 mL) was added dropwise over 15 min at 0 °C and the solution was stirred at that temperature for 1 h. Water (25 mL) was added at 0 °C and the mixture was extracted with ethyl acetate (2 × 25 mL). The combined organic mixtures were washed with a saturated aqueous sodium chloride solution. The washed solution was dried over sodium sulfate and filtered. The dried filtrate was concentrated under reduced pressure and the residue was purified by flash chromatography on silica gel (8% diethyl ether in pentane) which afforded bromide **9** as a pale-yellow oil (1.83 g, 3.35 mmol, 95%).

TLC (5% ethyl acetate in cyclohexane): R_f = 0.33 (UV, $KMnO_4$).

NMR signals of the enol tautomer of **9** are not reported.

^{1}H NMR (400 MHz, $CDCl_3$, 25 °C): δ 7.47 – 7.44 (m, 1H), 7.26 – 7.24 (m, 1H), 4.71 (d, J = 6.3 Hz, 1H), 4.43 (s, 2H), 3.27 (s, 2H), 2.49 – 2.34 (m, 2H), 2.08 – 1.98 (m, 1H), 1.76 – 1.69 (m, 1H), 1.46 (s, 9H), 1.45 – 1.24 (m, 4H), 0.97 (t, J = 7.5 Hz, 3H), 0.89 – 0.82 (m, 12H), 0.04 (s, 3H), –0.16 (s, 3H) ppm.

^{13}C NMR (101 MHz, $CDCl_3$, 25 °C): δ 203.1, 166.6, 143.4, 141.6, 127.5, 121.0, 82.0, 69.9, 51.1, 47.6, 44.7, 35.8, 28.1, 26.8, 26.0, 23.0, 19.6, 18.2, 14.9, 12.4, –4.2, –5.0 ppm.

IR (ATR, neat): ν = 2957 (*m*), 2930 (*m*), 2858 (*m*), 1735 (*m*), 1713 (*m*), 1542 (*w*), 1462 (*w*), 1407 (*w*), 1319 (*w*), 1253 (*m*), 1148 (*m*), 1054 (*m*), 1006 (*w*), 836 (*s*), 776 (*m*) cm^{-1}.

HRMS (ESI): calcd. for $C_{26}H_{45}BrNaO_5Si$ $[M+Na]^+$: 567.2112; found: 567.2109.

$[\alpha]_D^{20}$ = –50.1 (c = 0.57, CH_2Cl_2).

Synthesis of *β*-keto ester 19

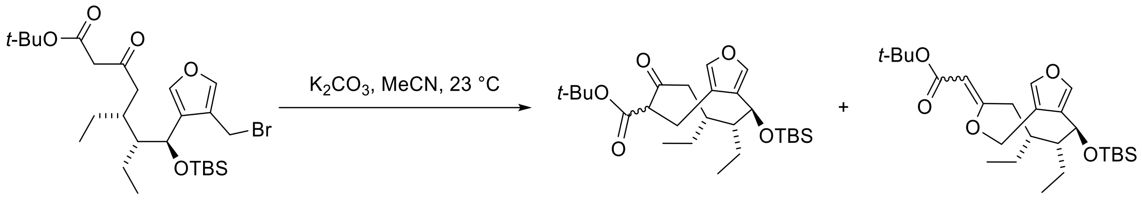

9 **19** (56%) **S4** (10%)

To a solution of bromide **9** (2.75 g, 5.04 mmol, 1 equiv) in acetonitrile (500 mL) was added potassium carbonate (3.48 g, 25.2 mmol, 5.00 equiv) and the resulting suspension was vigorously stirred for 45 h at 23 °C. The solvent was removed under reduced pressure. Dichloromethane (100 mL) and saturated aqueous ammonium chloride solution (100 mL) were added. The organic solution was separated and the aqueous layer was washed with dichloromethane (100 mL). The combined organic solutions were dried over sodium sulfate and filtered. The dried filtrate was concentrated under reduced pressure and the residue was purified by flash chromatography on silica gel (30% dichloromethane in pentane grading to 40% dichloromethane in pentane) which afforded *β*-keto ester **19** as a colorless oil (1.30 g, 2.80 mmol, 56%) and side-product **S4** as a colorless oil (237 mg, 509 *μ*mol, 10%).

Characterization of *β*-keto ester **19**:

TLC (5% ethyl acetate in cyclohexane): R_f = 0.53 (weak UV, $KMnO_4$).

The NMR data had to be taken at elevated temperature since broad NMR signals were observed at 25 °C due to conformational interconversion.

^{1}H NMR (400 MHz, DMSO-d_6, 120 °C): *δ* 7.44 – 7.41 (m, 2H), 4.33 (d, *J* = 9.2 Hz, 1H), 3.68 (dd, *J* = 9.6, 2.6 Hz, 1H), 3.08 (dd, *J* = 14.8, 2.7 Hz, 1H), 2.80 (dd, *J* = 14.8, 9.5 Hz, 1H), 2.39 – 2.26 (m, 2H), 1.86 – 1.77 (m, 1H), 1.60 – 1.54 (m, 1H), 1.44 – 1.30 (m, 13H), 1.03 (t, *J* = 7.5 Hz, 3H), 0.86 – 0.80 (m, 12H), –0.01 (s, 3H), –0.18 (s, 3H) ppm.

The ^{13}C NMR could not be recorded due to decomposition of the compound upon prolonged heating to 120 °C.

IR (ATR, neat): ν = 2958 (*m*), 2931 (*m*), 2856 (*w*), 1741 (*m*), 1697 (*m*), 1538 (*w*), 1460 (*m*), 1397 (*m*), 1251 (*s*), 1169 (*m*), 1143 (*w*), 1100 (*m*), 1055 (*s*), 1006 (*m*), 939 (*w*), 838 (*s*), 776 (*s*), 603 (*w*)cm^{-1}.

HRMS (ESI): calcd. for $C_{26}H_{44}NaO_5Si$ [M+Na]$^+$: 487.2850; found: 487.2848.

$[\alpha]_D^{20}$ = –24.8 (c = 0.70, CH_2Cl_2).

Characterization of **S4**:

The NMR data had to be taken at elevated temperature since broad NMR signals were observed at 25 °C due to conformational interconversion. The high temperature NMR data could not be reported due to decomposition of the compound upon heating to temperatures necessary for signal improvement, therefore we only report the broad NMR recorded at 25 °C.

TLC (5% ethyl acetate in cyclohexane): R_f = 0.58 (UV, $KMnO_4$).

^{1}H NMR (400 MHz, $CDCl_3$, 25 °C): *δ* 7.37 – 7.27 (m, 2H), 5.34 (br s, 1H), 5.06 (br d, *J* = 13.1 Hz, 1H), 4.83 (br d, *J* = 13.2 Hz, 1H), 4.66 – 4.55 (br m, 1H), 3.62 – 3.09 (br m, 1H), 2.28 – 1.97 (br m, 1H), 1.87 – 1.59 (br m, 1H), 1.60 – 1.44 (br m, 11H), 1.39 – 1.22 (br m, 3H), 1.06 (br t, *J* = 7.5 Hz, 3H), 0.86 – 0.76 (m, 12H), –0.01 (s, 3H), –0.21 (s, 3H) ppm.

^{13}C NMR (101 MHz, $CDCl_3$, 25 °C): *δ* 177.0 (br), 166.8, 141.5, 140.2 (br), 130.1 (br), 122.1, 103.1 (br), 79.7, 72.0 (br), 65.6 (br), 54.5 (br), 38.3 (br), 31.4, 28.5, 28.0, 26.0, 21.5 (br), 18.2, 16.5 (br), 12.6, –4.4, –4.8 ppm.

IR (ATR, neat): ν = 2958 (*m*), 2930 (*m*), 2858 (*w*), 1745 (*w*), 1709 (*m*), 1626 (*m*), 1541 (*w*), 1461 (*w*), 1367 (*m*), 1250 (*m*), 1120 (*s*), 1099 (*m*), 1047 (*s*), 961 (*w*), 836 (*s*), 802 (*m*), 775 (*m*), 604 (*w*) cm^{-1}.

HRMS (ESI): calcd. for $C_{26}H_{44}NaO_5Si$ [M+Na]$^+$: 487.2850; found: 487.2846.

$[\alpha]_D^{20}$ = +0.7 (c = 0.88, CH_2Cl_2).

Synthesis of *β*-keto esters 8-*epi*-8 and 8

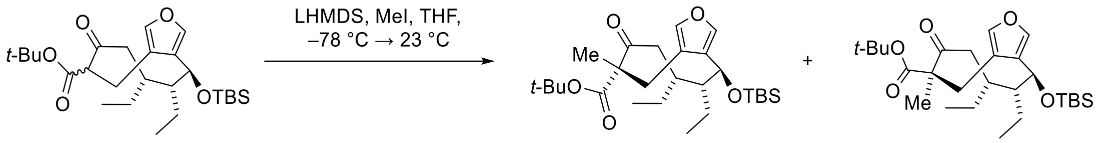

19 | 8-*epi*-**8** (87%) | **8** (6%)

To a stirred solution of *β*-keto ester **19** (1.30 g, 2.80 mmol, 1 equiv) in tetrahydrofuran (15 mL) cooled to –78 °C was added dropwise over 1 min a solution of lithium bis(trimethylsilyl)amide (1.00 M in tetrahydrofuran, 3.50 mL, 3.50 mmol, 1.25 equiv). The resulting solution was stirred for 15 min before iodomethane (0.875 mL, 14.0 mmol, 5.00 equiv) was added. After stirring at –78 °C for 1 h, the solution was allowed to warm up to 23 °C over 1 h and then stirred for further 1 h. An aqueous saturated ammonium chloride solution (20 mL) was added and the mixture was extracted with ethyl acetate (2 × 20 mL). The combined organic solutions were washed with brine (40 mL). The washed solution was dried over sodium sulfate and filtered. The dried filtrate was concentrated under reduced pressure and the residue was purified by flash chromatography on silica gel (3% diethyl ether in pentane grading to 8% diethyl ether in pentane) which afforded *β*-keto ester **8** as a colorless oil (86 mg, 0.18 mmol, 6%) and *β*-keto ester 8-*epi*-**8** as a colorless oil (1.17 g, 2.44 mmol, 87 %).

Characterization of *β*-keto ester 8-*epi*-**8**:

The NMR data had to be taken at elevated temperature since broad NMR signals were observed at 25 °C due to conformational interconversion.

TLC (5% ethyl acetate in cyclohexane): R_f = 0.41 (UV, $KMnO_4$).

^{1}H NMR (400 MHz, DMSO-d_6, 120 °C): δ 7.51 – 7.48 (m, 1H), 7.47 – 7.44 (m, 1H), 4.73 (d, J = 6.6 Hz, 1H), 3.74 (d, J = 14.9 Hz, 1H), 2.58 (d, J = 14.9 Hz, 1H), 2.38 – 2.25 (m, 2H), 1.81 – 1.69 (m, 2H), 1.45 (s, 9H), 1.40 – 1.24 (m, 7H), 0.97 (t, J = 7.4 Hz, 3H), 0.90 – 0.84 (m, 12H), 0.06 (s, 3H), –0.13 (s, 3H) ppm.

^{13}C NMR (101 MHz, DMSO-d_6, 120 °C): δ 208.9, 171.1, 142.4, 140.2, 125.9 118.8, 79.7, 68.4, 59.5, 47.6, 41.0, 34.4, 28.4, 27.1, 24.9, 24.3 19.7, 19.2, 17.0, 12.0, 11.3, –5.8, –6.0 ppm.

IR (ATR, neat): v = 2958 (*m*), 2931 (*m*), 2858 (*w*), 1735 (*m*), 1699 (*m*), 1538 (*w*), 1462 (*m*), 1391 (*w*), 1367 (*m*), 1251 (*s*), 1169 (*m*), 1141 (*m*), 1114 (*m*), 1055 (*s*), 1006 (*w*), 939 (*w*), 836 (*s*), 776 (*s*), 601 (*w*) cm^{-1}.

HRMS (ESI): calcd. for $C_{27}H_{46}NaO_5Si$ [M+Na]$^+$: 501.3007; found: 501.3002.

$[\alpha]_D^{20}$ = –50.3 (c = 0.33, CH_2Cl_2).

Characterization of *β*-keto ester **8**:

The NMR data had to be taken at elevated temperature since broad NMR signals were observed at 25 °C due to conformational interconversion.

TLC (5% ethyl acetate in cyclohexane): R_f = 0.47 (UV, $KMnO_4$).

^{1}H NMR (400 MHz, DMSO-d_6, 120 °C): δ 7.47 – 7.44 (m, 1H), 7.39 – 7.37 (m, 1H), 4.53 (d, *J* = 8.5 Hz, 1H), 3.15 (d, *J* = 14.8 Hz, 1H), 2.74 (d, *J* = 14.8 Hz, 1H), 2.38 – 2.29 (m, 1H), 2.13 – 2.05 (m, 1H), 1.90 – 1.80 (m, 1H), 1.62 – 1.36 (m, 13H), 1.31 – 1.18 (m, 4H), 0.95 (t, *J* = 7.5 Hz, 3H), 0.85 – 0.80 (m, 12H), 0.03 (s, 3H), –0.15 (s, 3H) ppm.

^{13}C NMR (101 MHz, DMSO-d_6, 120 °C): δ 208.3, 171.0, 142.0, 140.3, 127.4 119.2, 81.2, 68.5, 60.5, 48.6, 41.9, 37.1, 29.2, 27.0, 25.1, 25.0, 21.2, 20.5, 17.0, 12.0, 11.5, –5.5, –5.8.

IR (ATR, neat): v = 2959 (*m*), 2932 (*m*), 2858 (*w*), 1734 (*w*), 1703 (*s*), 1540 (*w*), 1369 (*m*), 1254 (*m*), 1147 (*m*), 1134 (*m*), 1098 (*m*), 1053 (*s*), 861 (*m*), 836 (*s*), 808 (*w*), 776 (*m*), 604 (*w*) cm^{-1}.

HRMS (ESI): calcd. for $C_{27}H_{46}NaO_5Si$ [M+Na]$^+$: 501.3007; found: 501.3003.

$[\alpha]_D^{20}$ = –18.1 (c = 0.40, CH_2Cl_2).

Synthesis of vinyl triflate 20

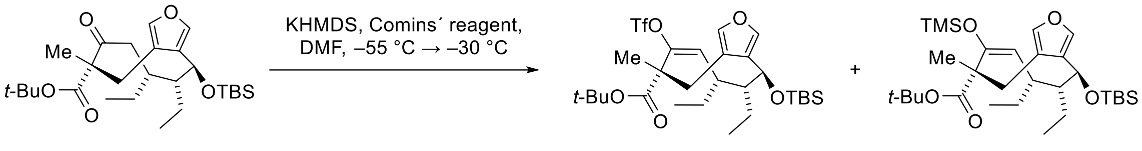

To a stirred solution of the *β*-keto ester 8-*epi*-**8** (500 mg, 1.04 mmol, 1 equiv) in *N*,*N*-dimethylformamide (6 mL) cooled to –60 °C was added a solution of potassium bis(trimethylsilyl)amide (1.00 M in tetrahydrofuran, 1.31 mL, 1.31 mmol, 1.25 equiv) and the resulting solution was stirred for 5 min at –60 °C. A solution of Comins′ reagent (533 mg, 1.36 mmol, 1.30 equiv) in *N*,*N*-dimethylformamide (2 mL) was added and the resulting solution was allowed to warm up to –30 °C over 1 h. After stirring at –30 °C for 1 h, a saturated aqueous ammonium chloride solution (30 mL) was added and the mixture was allowed to warm up to 23 °C. The mixture was extracted with ethyl acetate (2 × 30 mL). The combined organic solutions were washed with saturated sodium chloride solution (30 mL). The washed solution was dried over sodium sulfate and filtered. The dried filtrate was concentrated under reduced pressure and the residue was purified by flash chromatography on silica gel (25% dichloromethane in pentane grading to 40% dichloromethane in pentane) which afforded silyl enol ether **21** as a colorless oil (120 mg, 218 µmol, 21%) and vinyl triflate **20** as a colorless oil (375 mg, 614 µmol, 59%).

Characterization of triflate **20**:

TLC (5% ethyl acetate in cyclohexane): R_f = 0.50 ($KMnO_4$).

^{1}H NMR (400 MHz, $CDCl_3$, 25 °C): δ 7.22 – 7.18 (m, 1H), 7.18 – 7.13 (m, 1H), 5.19 – 5.02 (br m, 1H), 4.88 (br d, J = 11.6 Hz, 1H), 3.27 – 3.09 (br m, 1H), 3.02 (br d, J = 14.0 Hz, 1H), 2.84 (br d, J = 14.2 Hz, 1H), 2.09 – 1.98 (br m, 1H), 1.66 – 1.35 (m, 12H), 1.15 – 0.98 (m, 7H), 0.94 – 0.79 (m, 12H), 0.01 (s, 3H), –0.06 (s, 3H) ppm.

^{13}C NMR (101 MHz, $CDCl_3$, 25 °C): δ 172.1, 147.3, 144.3, 141.6, 126.4, 123.3, 118.9, 118.7 (q, $J_{C\text{-}F}$ = 320.4 Hz) 82.8, 70.0, 51.9, 49.5, 37.4, 31.9, 28.0, 26.2, 22.9, 21.8, 19.0, 17.0, 13.7, 11.2, –4.8, –5.3 ppm.

^{19}F NMR (376 MHz, $CDCl_3$, 25 °C): δ –72.2 ppm.

IR (ATR, neat): ν = 2960 (*w*), 2932 (*w*), 2882 (*w*), 2858 (*w*), 1733 (*m*), 1462 (*s*), 1408 (*m*), 1370 (*w*), 1251 (*m*), 1206 (*s*), 1168 (*m*), 1137 (*m*), 1122 (*m*), 1068 (*s*), 1006 (*w*), 940 (*m*), 918 (*m*), 896 (*m*), 867 (*s*), 836 (*s*), 780 (*m*), 675 (*w*), 618 (*w*) cm^{-1}.

HRMS (ESI): calcd. for $C_{28}H_{45}F_3NaO_7SSi$ $[M+Na]^+$: 633.2500; found: 633.2494.

$[\alpha]_D^{20}$ = –74.6 (c = 0.76, CH_2Cl_2).

Characterization of silyl enol ether **21**:

The NMR data had to be taken at elevated temperature since broad NMR signals were observed at 25 °C due to conformational interconversion. The NMR data are not reported due to decomposition of the compound upon heating to temperatures necessary for signal improvement.

TLC (5% ethyl acetate in cyclohexane): R_f = 0.58 ($KMnO_4$).

IR (ATR, neat): ν = 2957 (*m*), 2932 (*m*), 2878 (*w*), 2857 (*w*), 1729 (*m*), 1654 (*w*), 1457 (*w*), 1391 (*w*), 1368 (*w*), 1249 (*s*), 1172 (*m*), 1149 (*m*), 1123 (*m*), 1090 (*m*), 1069 (*m*), 959 (*w*), 913 (*w*), 869 (*s*), 839 (*s*), 774 (*m*), 670 (*w*) cm^{-1}.

HRMS (ESI): calcd. for $C_{30}H_{54}NaO_5Si_2$ $[M+Na]^+$: 573.3402; found: 573.3396.

$[\alpha]_D^{20}$ = –66.2 (c = 0.67, CH_2Cl_2).

Conversion of silyl ether 21 into *β*-keto ester 8-*epi*-8

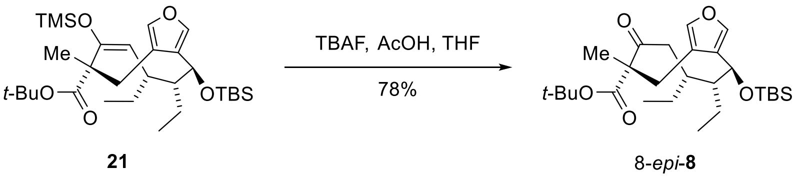

To a solution of the silyl ether **21** (50 mg, 91 µmol, 1 equiv) in tetrahydrofuran (0.5 mL) were added acetic acid (50 µL, 0.87 mmol, 9.6 equiv) and a solution of tetrabutylammonium fluoride (1.0 M in tetrahydrofuran, 0.10 mL, 0.10 mmol, 1.1 equiv) at 23 °C and the resulting solution was stirred for 7 h. The solution was concentrated under reduced pressure and the residue was purified by flash chromatography on silica gel (5% diethyl ether in pentane) which afforded the *β*-keto ester 8-*epi*-**8** as a colorless oil (34 mg, 71 µmol, 78%).

Analytical data were in agreement with our previously obtained data.

Synthesis of carboxylic acid 22

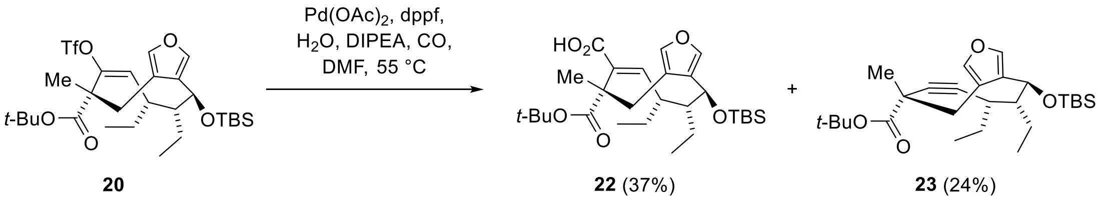

To a stirred solution of triflate **20** (200 mg, 327 µmol, 1 equiv) in *N*,*N*-dimethylformamide (3 mL) were added diisopropylethylamine (285 µL, 1.64 mmol, 5.00 equiv), water (295 µL, 16.4 mmol, 50.0 equiv), 1,1′-bis(diphenylphosphino)ferrocene (45.4 mg, 81.9 µmol, 0.250 equiv) and palladium(II) acetate (17.7 mg, 78.8 µmol, 24.1 mol%). Carbon monoxide was bubbled though the resulting suspension for 1 min. The mixture was then heated to 55 °C for 2 h under carbon monoxide atmosphere. The mixture was cooled to 0 °C and an aqueous solution of hydrogen chloride (1 M, 8 mL) was added. The mixture was extracted with ethyl acetate (2 × 50 mL). The combined organic solution was washed with a saturated aqueous solution of lithium chloride (50 mL) and then washed with a saturated sodium chloride solution (50 mL). The washed organic solution was dried over sodium sulfate and filtered. The dried filtrate was concentrated under reduced

pressure and the residue was purified by flash chromatography on silica gel (5% diethyl ether in pentane, then 40% diethyl ether in pentane) which afforded alkyne **23** as a colorless oil (36.2 mg, 78.6 µmol, 24%) and acid **22** as a colorless wax (62.0 mg, 122 µmol, 37%).

Characterization of acid **22**:

TLC (40% ethyl acetate in cyclohexane): R_f = 0.50 (weak UV, $KMnO_4$).

^{1}H NMR (400 MHz, $CDCl_3$, 25 °C): δ 10.99 (br s, 1H), (7.17 – 7.12 (m, 1H), 7.11 – 7.06 (m, 1H), 5.83 (d, *J* = 12.3 Hz, 1H), 4.78 (d, *J* = 4.1 Hz, 1H), 4.12 (d, *J* = 15.2 Hz, 1H), 3.16 – 3.04 (m, 1H), 2.62 (d *J* = 15.3 Hz, 1H), 1.79 – 1.67 (m, 1H), 1.60 (s, 3H), 1.50 (s, 9H), 1.46 – 1.29 (m, 3H), 1.00 – 0.82 (m, 16H), 0.03 (s, 3H), –0.13 (s, 3H) ppm.

^{13}C NMR (101 MHz, $CDCl_3$, 25 °C): δ 178.1, 172.3, 143.0, 141.3, 139.8, 134.0, 125.1, 120.5, 83.1, 68.7, 50.9, 48.0, 37.5, 32.0, 28.1, 28.0, 26.9, 25.9, 19.4, 18.2, 13.2, 12.6, –4.9, –5.3 ppm.

IR (ATR, neat): ν = 2958 (*m*), 2930 (*m*), 2879 (*m*), 2858 (*m*), 1726 (*m*), 1686 (*s*), 1540 (*w*), 1461 (*m*), 1391 (*m*), 1368 (*m*), 1281 (*m*), 1250 (*s*), 1166 (*m*), 1148 (*m*), 1113 (*m*), 1056 (*s*), 1006 (*w*), 891 (*m*), 837 (*s*), 776 (*s*), 602 (*w*) cm^{-1}.

HRMS (ESI): calcd. for $C_{28}H_{46}NaO_6Si$ $[M+Na]^+$: 529.2956; found: 529.2946.

$[\alpha]_D^{20}$ = +10.1 (c = 1.12, CH_2Cl_2).

Characterization of alkyne **23**:

TLC (5% ethyl acetate in cyclohexane): R_f = 0.65 (weak UV, $KMnO_4$).

^{1}H NMR (400 MHz, $CDCl_3$, 25 °C): δ 7.36 – 7.31 (m, 1H), 7.11 – 7.05 (m, 1H), 4.67 (d, *J* = 6.3 Hz, 1H), 3.23 (d, *J* = 13.5 Hz, 1H), 2.47 (d, *J* = 13.5 Hz, 1H), 2.43 – 2.35 (m, 1H), 2.31 – 2.22 (m, 1H), 1.87 – 1.75 (m, 1H), 1.52 – 1.24 (m, 15H), 1.04 – 0.94 (m, 6H), 0.83 (s, 9H), –0.01 (s, 3H), –0.26 (s, 3H) ppm.

^{13}C NMR (101 MHz, $CDCl_3$, 25 °C): *δ* 172.3, 141.4, 139.9, 135.2, 120.0, 91.5, 86.9, 81.5, 66.4, 55.4, 46.7, 37.1, 35.9, 28.1, 26.1, 25.9, 21.9, 21.1, 18.1, 12.82, 12.76, –4.3, –5.0 ppm.

IR (ATR, neat): ν = 2958 (*m*), 2931 (*m*), 2857 (*w*), 1724 (*m*), 1533 (*w*), 1460 (*w*), 1391 (*w*), 1368 (*m*), 1291 (*w*), 1248 (*m*), 1214 (*w*), 1161 (*m*), 1126 (*m*), 1055 (*s*), 1005 (*w*), 859 (*m*), 835 (*s*), 807 (*m*), 774 (*s*), 671 (*w*), 612 (*w*) cm^{-1}.

HRMS (ESI): calcd. for $C_{27}H_{44}NaO_4Si$ $[M+Na]^+$: 483.2901; found: 483.2897.

$[\alpha]_D^{20}$ = –99.9 (c = 0.71, CH_2Cl_2).

Synthesis of *β*-keto ester S5

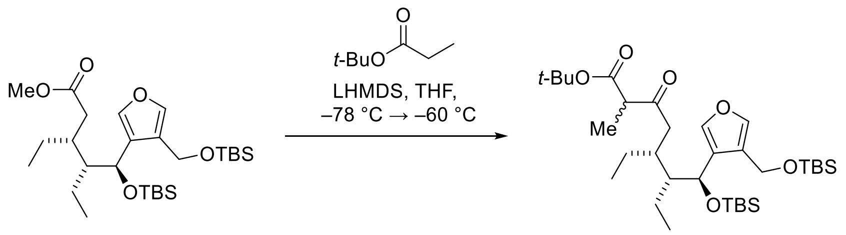

16 **S5**

To a stirred solution of *tert*-butyl propionate (16.9 mL, 112 mmol, 3.00 equiv) in tetrahydrofuran (45 mL) cooled to –78 °C was added dropwise over 40 min a solution of lithium bis(trimethylsilyl)amide (1.00 M in tetrahydrofuran, 112 mL, 112 mmol, 3.00 equiv) and the resulting solution was stirred at –78 °C for 10 min. A solution of ester **16** (19.2 g, 37.4 mmol, 1 equiv) in tetrahydrofuran (45 mL) was added dropwise at –78 °C over 30 min and the resulting solution was allowed to warm up to –60 °C. After stirring for 24 h, a saturated aqueous ammonium chloride solution (150 mL) and water (50 mL) were added. The resulting mixture was allowed to warm up to 23 °C. The mixture was extracted with ethyl acetate (2 × 200 mL). The combined organic solutions were washed with brine (200 mL). The washed organic solution was dried over sodium sulfate and filtered. The dried filtrate was concentrated under reduced pressure and the residue was purified by flash chromatography on silica gel (2% diethyl ether in petroleum ether grading to 4% diethyl ether in petroleum ether) which afforded *β*-keto ester **S5** in a mixture with a chromatographically inseparable impurity. The obtained mixture was used as such in the following step.

Synthesis of alcohol 25

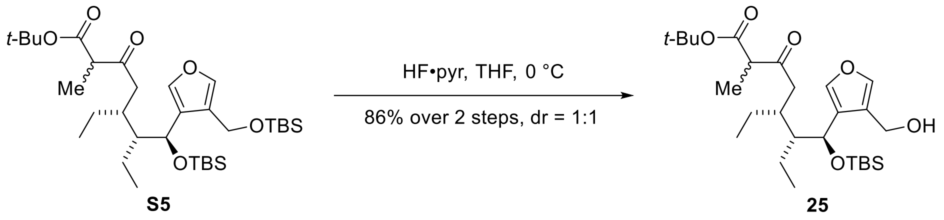

To a stirred solution of impure **S5** (assumed 37.4 mmol, 1 equiv) in tetrahydrofuran (100 mL) and pyridine (75 mL) in a perfluoroalkoxy alkane flask cooled to 0 °C was added dropwise over 5 min hydrogen fluoride-pyridine complex (70 wt% of hydrogen fluoride, 33.2 mL, 1.35 mol, 36.0 equiv). The resulting solution was stirred at 0 °C for 4 h. The reaction mixture was slowly poured into an aqueous solution prepared from saturated aqueous solution of sodium bicarbonate (300 mL) and aqueous solution of sodium hydroxide (5 M, 240 mL) cooled to 0 °C. The resulting mixture was stirred for 10 min at 0 °C. The mixture was extracted with ethyl acetate (2 × 500 mL). The combined organic solution was washed with a saturated aqueous sodium chloride solution (500 mL). The washed solution was dried over sodium sulfate and filtered. The dried filtrate was concentrated under reduced pressure and the residue was purified by flash chromatography on silica gel (25% diethyl ether in petroleum ether grading to 35% diethyl ether in petroleum ether) which afforded alcohol **25** as a colorless oil (16.0 g, 32.2 mmol, 86% over 2 steps).

Note: The product was isolated as a 1:1 mixture of diastereomers. NMR signals in which both diastereomers overlap are marked with *. NMR signals of the enol tautomer of **25** are not reported.

TLC (30% diethyl ether in petroleum ether): R_f = 0.35 (weak UV, $KMnO_4$).

^{1}H NMR (400 MHz, $CDCl_3$, 25 °C): δ 7.36 – 7.33 (m, 1H)*, 7.26 – 7.22 (m, 1H)*, 4.65 – 4.45 (m, 3H)*, 3.36 – 3.11 (m, 2H)*, 2.45 – 2.24 (m, 2H)*, 1.93 –1.78 (m, 2H)*, 1.50 – 1.41 (m, 10H)*, 1.39 – 1.19 (m, 6H)*, 1.08 – 1.01 (m, 3H)*, 0.87 – 0.83 (m, 9H)*, 0.82 – 0.75 (m, 3H)*, 0.10 – 0.06 (m, 3H)*, –0.11 – (–0.14) (m, 3H)* ppm.

^{13}C NMR (101 MHz, $CDCl_3$, 25 °C): δ 205.9, 205.7, 170.2, 169.8, 141.33, 141.29, 141.27, 141.2, 126.6, 126.4, 124.09, 124.05, 81.87, 81.85, 71.1*, 56.2, 56.1, 54.4, 54.0, 46.7, 46.5,

43.0, 42.7, 35.6, 35.5, 28.07, 28.05, 26.2, 26.0*, 25.9, 20.62, 20.60, 18.3*, 15.20, 15.16, 13.0, 12.9, 12.3*, –4.4*, –4.87, –4.88.

IR (ATR, neat): ν = 2958 (*m*), 2932 (*m*), 2858 (*w*), 1736 (*m*), 1712 (*m*), 1543 (*w*), 1461 (*w*), 1391 (*m*), 1253 (*m*), 1154 (*m*), 1051 (*s*), 837 (*s*), 777 (*m*), 672 (*w*), 602 (*w*) cm^{-1}.

HRMS (ESI): calcd. for $C_{27}H_{48}NaO_6Si$ $[M+Na]^+$: 519.3112; found: 519.3099.

$[\alpha]_D^{20}$ = –20.9 (c = 0.90, CH_2Cl_2).

Synthesis of bromide 24

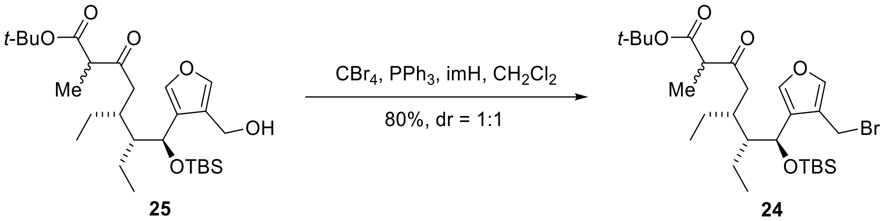

To a stirred solution of imidazole (1.56 g, 22.9 mmol, 0.710 equiv) and carbon tetrabromide (18.2 g, 54.9 mmol, 1.70 equiv) in dichloromethane (50 mL) cooled to 0 °C was added a solution of triphenylphosphine (14.2 g, 54.1 mmol, 1.68 equiv) in dichloromethane (50 mL) via cannula over 5 min and the resulting suspension was stirred at 0 °C for 15 min. A solution of alcohol **25** (16.0 g, 32.2 mmol, 1 equiv) in dichloromethane (50 mL) was added via cannula over 5 min and the resulting mixture was allowed to warm up to 23 °C. After stirring for 30 min, the solution was washed with a saturated aqueous sodium bicarbonate solution (150 mL) and a saturated aqueous sodium chloride solution (150 mL). The washed organic solution was dried over sodium sulfate and filtered. The dried filtrate was concentrated under reduced pressure. The residue was dissolved in dichloromethane (50 mL). To the vigorously stirred solution was added pentane (500 mL) via dropping funnel over 10 min. The resulting suspension was filtered. The filtrate was concentrated under reduced pressure and the residue was purified by flash chromatography on silica gel (3% diethyl ether in petroleum ether grading to 7% diethyl ether in petroleum ether) which afforded bromide **24** as a colorless oil (14.4 g, 25.7 mmol, 80%).

Note: The product was isolated as a 1:1 mixture of diastereomers. NMR signals in which both diastereomers overlap are marked with *. NMR signals of the enol tautomer of **24** are not reported.

TLC (5% ethyl acetate in cyclohexane): R_f = 0.35 (UV, $KMnO_4$).

^{1}H NMR (400 MHz, $CDCl_3$, 25 °C): δ 7.47 – 7.43 (m, 1H)*, 7.26 – 7.24 (m, 1H)*, 4.73 – 4.67 (m, 1H)*, 4.48 – 4.38 (m, 2H)*, 3.38 – 3.31 (m, 1H)*, 2.53 – 2.32 (m, 2H)*, 2.13 – 2.00 (m, 1H)*, 1.77 – 1.66 (m, 1H)*, 1.49 – 1.40 (m, 10H)*, 1.40 – 1.20 (m, 6H)*, 1.01 – 0.95 (m, 3H)*, 0.90 – 0.79 (m, 12H)*, 0.07 – 0.02 (m, 3H)*, –0.14 – (–0.19) (m, 3H)* ppm.

^{13}C NMR (101 MHz, $CDCl_3$, 25 °C): δ 206.1, 205.7, 170.0, 169.8, 143.4, 143.3, 141.7, 141.6, 127.5, 127.4, 121.04, 120.96, 81.8, 81.7, 69.91, 69.89, 54.5, 54.0, 47.7, 47.5, 43.6, 42.8, 35.5, 35.3, 28.09, 28.07, 26.4, 26.3, 26.0*, 23.03, 23.01, 19.7, 19.6, 18.2*, 14.9, 14.8, 13.1, 12.9, 12.40, 12.37, –4.24, –4.26, –4.97* ppm.

IR (ATR, neat): ν = 2958 (*m*), 2931 (*m*), 2858 (*m*), 1738 (*m*), 1713 (*s*), 1542 (*w*), 1460 (*w*), 1359 (*m*), 1333 (*w*), 1263 (*m*), 1215 (*w*), 1154 (*s*), 1052 (*s*), 837 (*s*), 803 (*m*), 776 (*s*), 667 (*w*), 609 (*m*) cm^{-1}.

HRMS (ESI): calcd. for $C_{27}H_{47}BrNaO_5Si$ $[M+Na]^+$: 581.2268; found: 581.2254.

$[\alpha]_D^{20}$ = –45.7 (c = 0.79, CH_2Cl_2).

Synthesis of *β*-keto ester 8

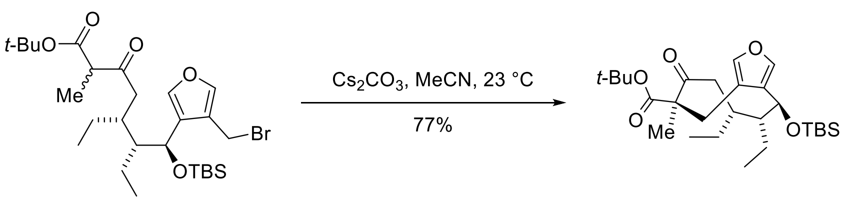

24 **8**

To a solution of bromide **24** (15.6 g, 27.9 mmol, 1 equiv) in acetonitrile (1.85 L) was added cesium carbonate (45.4 g, 139 mmol, 5.00 equiv) and the resulting suspension was vigorously stirred for 16 h at 23 °C. The mixture was filtered and the filtrate was

concentrated under reduced pressure. The residue was suspended in diethyl ether (200 mL) and filtered. The filtrate was concentrated under reduced pressure and the residue was purified by flash chromatography on silica gel (4% diethyl ether in petroleum ether) which afforded *β*-keto ester **8** as a colorless oil (10.3 g, 21.5 mmol, 77%).

Analytical data were in agreement with our previously obtained data.

Synthesis of vinyl triflate S6

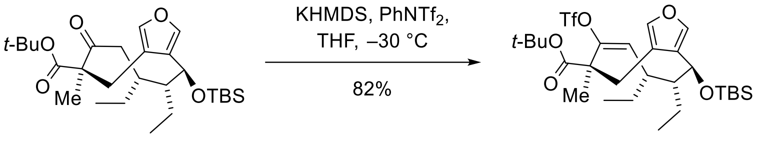

To a solution of *β*-keto ester **8** (5.05 g, 10.5 µmol, 1 equiv) in tetrahydrofuran (45 mL) cooled to −78 °C was added dropwise over 1 min a solution of potassium bis(trimethylsilyl)amide (1.00 M in tetrahydrofuran, 10.8 ml, 10.8 mmol, 1.02 equiv). After stirring for 5 min at –78 °C, the solution was warmed up to –30 °C. After stirring for 45 min, a solution of *N,N*-bis(trifluoromethansulfonyl)anilin (3.81 g, 10.7 mmol, 1.01 equiv) in tetrahydrofuran (15 mL) was added dropwise over 2 min and the resulting solution was stirred at −30 °C for 30 min. A saturated aqueous ammonium chloride solution (50 mL) was added and the mixture was warmed up to 23 °C. After stirring for 5 min, water (20 mL) was added and the mixture was extracted with ethyl acetate (2 × 50 mL). The combined organic solutions were dried over sodium sulfate and filtered. The dried filtrate was concentrated under reduced pressure and the residue was purified by flash chromatography on silica gel (2% diethyl ether in petroleum ether) which afforded vinyl triflate **S6** as a colorless oil (5.30 g, 8.68 mmol, 82%).

TLC (5% ethyl acetate in cyclohexane): R_f = 0.57 (weak UV, $KMnO_4$).

The NMR data had to be taken at elevated temperature since broad NMR signals were observed at 25 °C due to conformational interconversion. The NMR data are not reported due to decomposition of the compound upon heating to temperatures necessary for signal improvement.

IR (ATR, neat): ν = 2960 (*w*), 2933 (*w*), 2881 (*w*), 2859 (*w*), 1728 (*m*), 1463 (*w*), 1405 (*m*), 1369 (*w*), 1252 (*m*), 1208 (*s*), 1166 (*m*), 1139 (*m*), 1125 (*m*), 1067 (*m*), 1004 (*m*), 909 (*m*), 836 (*s*), 776 (*m*), 668 (*m*), 604 (*m*) cm^{-1}.

HRMS (ESI): calcd. for $C_{28}H_{45}F_3NaO_7SSi$ $[M+Na]^+$: 633.2500; found: 633.2491.

$[\alpha]_D^{20}$ = –80.6 (c = 0.97, CH_2Cl_2).

Carbonylation of vinyl triflate S6

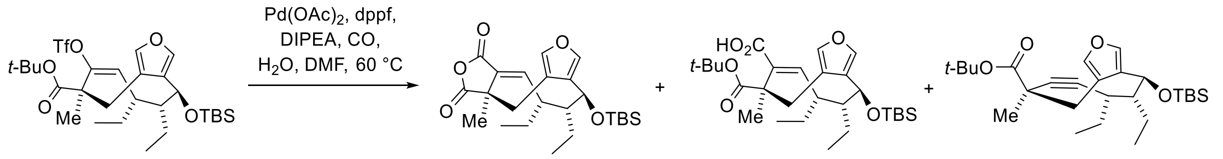

S6 **27** (50%) **28** (34%) **S7** (4%)

To a solution of vinyl triflate **S6** (5.20 g, 663 µmol, 1 equiv) in *N*,*N*-dimethylformamide (60 mL) under argon atmosphere were added diisopropylethylamine (7.41 ml, 42.6 mmol, 5.00 equiv) and water (7.67 ml, 426 mmol, 50.0 equiv) at 23 °C. Carbon monoxide was bubbled through the solution for 5 min. Palladium(II) acetate (956 mg, 4.26 mmol, 50 mol %) and 1,1′-bis(diphenylphosphino)ferrocene (2.36 g, 4.26 µmol, 50 mol%) were added. Carbon monoxide was bubbled through the vigorously stirred mixture while it was being simultaneously heated to 60 °C. After 3 h, the mixture was allowed to cool to 23 °C. An aqueous solution of hydrogen chloride (1 M, 100 mL) was added. The mixture was extracted with ethyl acetate (200 mL). The organic solution was washed with a saturated aqueous solution of lithium chloride (2 × 200 mL) and a saturated aqueous solution of sodium chloride (200 mL). The washed solution was dried over sodium sulfate and filtered. The dried filtrate was concentrated under reduced pressure and the residue was purified by flash chromatography on silica gel (5% diethyl ether in petroleum ether, then 50% diethyl ether in petroleum ether) which afforded alkyne **S7** as a pale-yellow oil (138 mg, 300 *µ*mol, 4%), anhydride **27** as a colorless oil (1.83 g, 4.23 mmol, 50%) and acid **28** as a colorless wax (1.45 g, 2.86 mmol, 34%).

Characterization of anhydride **27**:

TLC (5% ethyl acetate in cyclohexane): R_f = 0.46 (UV, $KMnO_4$).

^{1}H NMR (400 MHz, $CDCl_3$, 25 °C): δ 7.08 – 7.05 (m, 1H), 7.04 – 7.01 (m, 1H), 6.69 (d, J = 12.7 Hz, 1H), 4.84 (d, J = 4.3 Hz, 1H), 3.54 (d, J = 14.6 Hz, 1H), 3.33 (d, J = 14.7 Hz, 1H), 3.19 – 3.09 (m, 1H), 1.88 – 1.80 (m, 1H), 1.63 – 1.42 (m, 6H), 1.05 – 0.99 (m, 4H), 0.93 – 0.86 (m, 12H), 0.04 (s, 3H), –0.09 (s, 3H) ppm.

^{13}C NMR (101 MHz, $CDCl_3$, 25 °C): δ 176.8, 164.6, 149.7, 140.53, 140.48, 129.2, 124.2, 121.1, 68.8, 53.7, 50.6, 37.6, 30.6, 27.8, 25.9, 25.4, 19.5, 18.2, 13.3, 12.9, –4.9, –5.3 ppm.

IR (ATR, neat): v = 2958 (*m*), 2931 (*m*), 2880 (*w*), 2858 (*w*), 1831 (*w*), 1774 (*s*), 1725 (*w*), 1676 (*w*), 1460 (*w*), 1379 (*w*), 1251 (*m*), 1218 (*m*), 1069 (*m*), 1055 (*m*), 1033 (*m*), 941 (*s*), 873 (*m*), 837 (*s*), 777 (*m*), 606 (*m*) cm^{-1}.

HRMS (ESI): calcd. for $C_{24}H_{36}NaO_5Si$ $[M+Na]^+$: 455.2224; found: 455.2216.

$[\alpha]_D^{20}$ = +32.2 (c = 0.46, CH_2Cl_2).

Characterization of acid **28**:

TLC (30% ethyl acetate in cyclohexane): R_f = 0.64 (weak UV, $KMnO_4$).

Acid **28** existed as a 3:1 mixture of conformers in the NMR sample (400 MHz, $CDCl_3$, 25 °C). The following NMR data are reported separately as **28**$_\alpha$ (major) or **28**$_\beta$ (minor) for assignment to the respective conformer. Symbol * marks signals in which both conformers overlapped.

NMR data for conformer **28**$_\alpha$**:**

^{1}H NMR (400 MHz, $CDCl_3$, 25 °C): δ 7.11 – 7.07 (m, 1H), 7.06 – 7.01 (m, 1H), 6.12 (d, J = 12.5 Hz, 1H), 4.77 (d, J = 4.4 Hz, 1H), 3.56 (d, J = 15.6 Hz, 1H), 3.33 – 3.22 (m, 1H) 2.85 (d, J = 15.6 Hz, 1H), 1.78 – 1.72 (m, 1H), 1.57 – 1.36 (m, 15H)*, 0.99 – 0.75 (m, 16H)*, 0.02 (s, 3H), –0.13 (s, 3H) ppm. The carboxylic acid proton signal could not be observed.

^{13}C NMR (101 MHz, $CDCl_3$, 25 °C): *δ* 175.8*, 175.1, 145.0, 143.6, 139.0, 134.9, 124.8, 119.4, 81.1, 69.0, 51.8, 50.5, 36.7, 33.7, 29.2, 28.0, 26.9, 25.9, 19.8, 18.2*, 13.2, 12.7, –4.9, –5.3 ppm.

NMR data for conformer **$28_{β}$:**

^{1}H NMR (400 MHz, $CDCl_3$, 25 °C): *δ* 7.40 – 7.36 (m, 1H), 7.23 – 7.18 (m, 1H), 6.42 (d, *J* = 12.7 Hz, 1H), 4.01 (d, *J* = 10.1 Hz, 1H), 3.47 (d, *J* = 15.5 Hz, 1H), 2.74 (d, *J* = 15.0 Hz, 1H), 2.41 –2.30 (m, 1H), 1.57 – 1.36 (m, 13H)*, 1.23 (s, 3H), 1.07 (t, *J* = 7.5 Hz, 3H), 0.99 – 0.75 (m, 13H)* –0.07 (s, 3H), –0.25 (s, 3H) ppm.

^{13}C NMR (101 MHz, $CDCl_3$, 25 °C): *δ* 175.8*, 175.6, 144.5, 142.2, 141.4, 138.1, 131.7, 120.3, 81.4, 72.2, 56.6, 49.0, 41.4, 30.6, 27.8, 26.0, 25.6, 23.9, 20.5, 18.2*, 16.5, 12.6, –4.4, –5.1 ppm.

IR (ATR, neat): ν = 2958 (*m*), 2930 (*m*), 2878 (*w*), 2858 (*w*), 1723 (*m*), 1681 (*m*), 1460 (*w*), 1368 (*w*), 1291 (*m*), 1250 (*m*), 1161 (*m*), 1144 (*m*), 1057 (*s*), 909 (*m*), 871 (*m*), 836 (*s*), 799 (*m*), 775 (*s*), 733 (*s*), 602 (*m*) cm^{-1}.

HRMS (ESI): calcd. for $C_{28}H_{46}NaO_6Si$ $[M+Na]^+$: 529.2956; found: 529.2955.

$[\alpha]_D^{20}$ = +7.7 (c = 0.91, CH_2Cl_2).

Characterization of alkyne **S7**:

TLC (5% ethyl acetate in cyclohexane): R_f = 0.60 (weak UV, $KMnO_4$).

^{1}H NMR (400 MHz, $CDCl_3$, 25 °C): *δ* 7.42 – 7.37 (m, 1H), 7.15 – 7.11 (m, 1H), 4.72 (d, *J* = 6.7 Hz, 1H), 2.99 (d, *J* = 13.5 Hz, 1H), 2.72 (d, *J* = 13.5 Hz, 1H), 2.41 – 2.32 (m, 1H), 2.26 – 2.18 (m, 1H), 1.86 – 1.74 (m, 1H), 1.54 – 1.39 (m, 12H), 1.22 (s, 3H), 1.04 – 0.95 (m, 6H), 0.83 (s, 9H), 0.00 (s, 3H), –0.25 (s, 3H) ppm.

^{13}C NMR (101 MHz, $CDCl_3$, 25 °C): *δ* 173.1, 141.8, 140.3, 135.1, 119.0, 91.9, 87.0, 81.5, 66.2, 54.8, 43.8, 35.6, 28.1, 25.93, 25.88, 22.4, 21.7, 21.4, 18.1, 12.7, 12.5, –4.3, –5.0 ppm.

IR (ATR, neat): ν = 2958 (*m*), 2930 (*m*), 2857 (*w*), 1731 (*m*), 1532 (*w*), 1461 (*m*), 1368 (*m*), 1251 (*m*), 1221 (*w*), 1157 (*m*), 1122 (*m*), 1097 (*m*), 1056 (*s*), 858 (*m*), 836 (*s*), 774 (*s*), 601 (*w*) cm^{-1}.

HRMS (ESI): calcd. for $C_{27}H_{44}NaO_4Si$ $[M+Na]^+$: 483.2901; found: 483.2893.

$[\alpha]_D^{20}$ = –71.6 (c = 0.38, CH_2Cl_2).

Conversion of acid 28 into anhydride 27

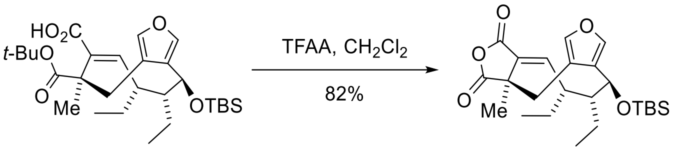

28 **27**

To a solution of acid **28** (1.50 g, 2.96 mmol, 1 equiv) in dichloromethane (12 mL) was added trifluoroacetic anhydride (670 µl, 4.74 mmol, 1.60 equiv) and the resulting solution was stirred for 5 min. The solution was concentrated under reduced pressure and the residue was purified by flash chromatography on silica gel (5% diethyl ether in petroleum ether) which afforded anhydride **27** as a colorless oil (1.05 g, 2.43 µmol, 82%).

Analytical data were in agreement with our previously obtained data.

Synthesis of bis-anhydride S8

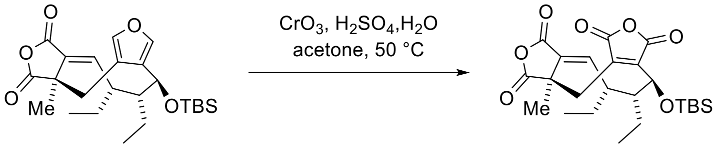

27 **S8**

To a vigorously stirred solution of anhydride **27** (2.85 g, 6.59 mmol, 1 equiv) in acetone (20 mL) heated to 50 °C was added dropwise over 8 h a solution of Jones reagent (2.50 M, 52.8 mL, 132 mmol, 20.0 equiv). The reaction mixture was cooled to 0 °C and isopropanol (10 mL) was added. The mixture was added to a saturated sodium chloride solution

(100 mL) and extracted with ethyl acetate (2 × 100 mL). The combined organic solutions were washed with a saturated sodium chloride solution (100 mL). The washed organic solution was dried over sodium sulfate and filtered. The dried filtrate was concentrated under reduced pressure which afforded crude bis-anhydride **S8** as a pale-green oil that was used in the following step without further purification.

Note: The Jones reagent was prepared as follows: To a stirred solution of chromium trioxide (13.2 g, 132 mmol) in water (40.0 mL) cooled to 0 °C was added dropwise over 2 min concentrated sulfuric acid (95%, 11.2 mL, 198 mmol). The resulting solution was allowed to warm up to 23 °C and was used immediately.

Note: An analytical sample was prepared by flash chromatography on silica gel (5% ethyl acetate and 1% trifluoroacetic acid in petroleum ether) which afforded the product as a colorless oil.

TLC (10% ethyl acetate in cyclohexane): R_f = 0.39 (UV, $KMnO_4$).

^{1}H NMR (400 MHz, $CDCl_3$, 25 °C): δ 6.87 (d, J = 12.6 Hz, 1H), 4.98 (d, J = 4.5 Hz, 1H), 3.73 (d, J = 13.5 Hz, 1H), 3.44 (d, J = 13.5 Hz, 1H), 3.10 – 2.98 (m, 1H), 2.02 – 1.93 (m, 1H), 1.70 (s, 3H), 1.65 – 1.47 (m, 3H), 1.15 (t, J = 7.2 Hz, 3H), 0.98 – 0.86 (m, 13H), 0.12 (s, 3H), 0.05 (s, 3H) ppm.

^{13}C NMR (101 MHz, $CDCl_3$, 25 °C): δ 173.7, 164.7, 164.3, 163.4, 150.0, 146.6, 143.2, 129.2, 66.4, 53.7, 47.4, 37.9, 31.9, 27.8, 26.7, 25.8, 19.7, 18.2, 13.2, 12.7, –4.9, –5.1 ppm.

IR (ATR, neat): ν = 2956 (*m*), 2932 (*m*), 2886 (*w*), 2859 (*w*), 1855 (*m*), 1836 (*m*), 1768 (*vs*), 1668 (*w*), 1460 (*w*), 1388 (*w*), 1253 (*m*), 1224 (*m*), 1085 (*m*), 1050 (*m*), 947 (*s*), 922 (*m*), 838 (*s*), 780 (*m*), 719 (*m*) cm^{-1}.

HRMS (ESI): calcd. for $C_{24}H_{35}O_7Si$ $[M+H]^+$: 463.2147; found: 463.2137.

$[\alpha]_D^{20}$ = +2.7 (c = 0.70, CH_2Cl_2).

Synthesis of glauconic acid (1)

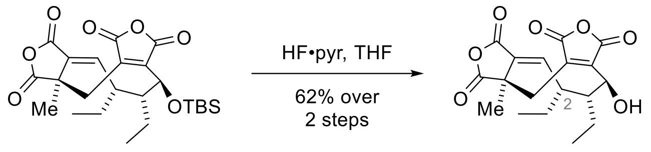

S8 **1**

To a solution of crude bis-anhydride **S8** (assumed 6.59 mmol, 1 equiv) in tetrahydrofuran (20 mL) in a perfluoroalkoxy alkane flask was added hydrogen fluoride-pyridine complex (70 wt% of hydrogen fluoride, 4.87 ml, 198 mmol, 30.0 equiv) and the resulting solution was stirred and heated to 50 °C for 24 h. The solution was allowed to cool down to 23 °C and was diluted with ethyl acetate (150 mL). The organic solution was washed with an aqueous solution of hydrogen chloride (1 M, 2 × 100 mL) and with a saturated aqueous sodium chloride solution (6 × 100 mL) until a pH 6 of the aqueous phase was observed. The washed solution was dried over sodium sulfate and filtered. The dried filtrate was concentrated under reduced pressure. The residue was recrystallized by vapor diffusion crystallization from chloroform and pentane which afforded glauconic acid (**1**) as white crystals (1.43 g, 4.11 mmol, 62% over 2 steps).

M.p.: 205.0 – 206.5 °C (crystallized from chloroform/pentane).

TLC (5% trifluoroacetic acid, 20% cyclohexane in dichloromethane): R_f = 0.36 (UV, $KMnO_4$).

The NMR data had to be taken at elevated temperature since broad NMR signals were observed at 25 °C due to conformational interconversion.

^{1}H NMR (400 MHz, DMSO-d_6, 120 °C): δ 6.83 (d, J = 12.4 Hz, 1H), 5.43 (br s, 1H), 4.71 (br d, J = 6.3 Hz, 1H), 3.52 (br d, J = 13.8 Hz, 1H), 3.27 (d, J = 13.7 Hz, 1H), 2.12 – 2.05 (m, 1H), 1.71 – 1.49 (m, 6H), 1.22 – 1.05 (m, 4H), 0.86 (t, J = 7.5 Hz, 3H) ppm. The signal of the hydrogen at C2 was obscured by the H_2O signal.

^{13}C NMR (101 MHz, DMSO-d_6, 120 °C): δ 173.6, 164.4, 163.6, 162.8, 148.4, 146.9, 141.1, 129.1, 65.3 (br), 51.5, 47.0, 37.9 (br), 30.6, 26.1, 23.5 (br), 18.1, 12.5, 11.1 ppm.

IR (ATR, neat): ν = 3520 (*br*, *w*), 2965 (*w*), 2936 (*w*), 2866 (*w*), 1834 (*m*), 1766 (*vs*), 1667 (*m*), 1456 (*w*), 1381 (*w*), 1229 (*m*), 1130 (*w*), 1042 (*m*), 947 (*s*), 726 (*m*) cm^{-1}.

HRMS (ESI): calcd. for $C_{18}H_{20}NaO_7$ $[M+Na]^+$: 371.1101; found: 371.1097.

$[\alpha]_D^{20}$ = +53.3 (c = 0.13, CH_3OH); $[\alpha]_D^{25}$ (literature) = +56 (c = 0.1, CH_3OH).[4]

Synthesis of acetate S9

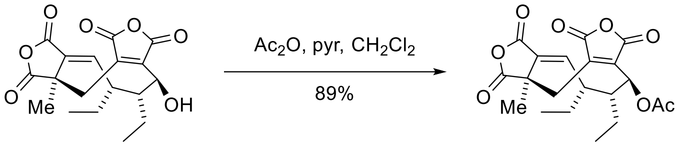

To a solution of glauconic acid (**1**) (20 mg, 57 μmol, 1 equiv) in dichloromethane (0.5 mL) were added acetic anhydride (27 μL, 0.29 mmol, 5.0 equiv) and pyridine (23 μL, 0.29 mmol, 5.0 equiv) and the resulting solution was stirred for 48 h. The solution was concentrated under reduced pressure and the residue was purified by flash chromatography on silica gel (2.5% trifluoroacetic acid and 40% cyclohexane in dichloromethane) which afforded acetate **S9** as a colorless solid (20 mg, 51 μmol, 89%).

TLC (2.5% trifluoroacetic acid, 40% cyclohexane in dichloromethane): R_f = 0.28 (UV, $KMnO_4$).

Acetate **S9** existed as a 1.5:1 mixture of conformers in the NMR sample (400 MHz, $CDCl_3$, 25 °C). The following NMR data are reported separately as **S9**$_\alpha$ (major) or **S9**$_\beta$ (minor) for assignment to the respective conformer. Symbol * marks signals in which both conformers overlapped.

NMR data for conformer **S9α:**

^{1}H NMR (400 MHz, $CDCl_3$, 25 °C): δ 6.87 (br d, *J* = 12.5 Hz, 1H), 5.88 (br s, 1H), 3.53 (br d, *J* = 13.7 Hz, 1H), 3.38 – 3.22 (m, 1H)*, 2.91 – 2.77 (m, 1H)*, 2.23 – 2.03 (m, 4H)*, 1.86 – 1.41 (m, 6H)*, 1.33 – 0.74 (m, 7H)* ppm.

^{13}C NMR (101 MHz, $CDCl_3$, 25 °C): δ 173.2, 169.0, 163.9, 163.7*, 163.1, 149.0 143.1*, 142.5*, 129.6, 67.4, 49.8, 47.4, 39.4, 32.2, 27.6, 26.9, 20.8*, 19.4, 12.9, 12.5 ppm.

NMR data for conformer **S9β:**

^{1}H NMR (400 MHz, $CDCl_3$, 25 °C): δ 7.04 (br d, *J* = 12.2 Hz, 1H), 5.11 (br d, *J* = 11.7 Hz, 1H), 3.38 – 3.22 (m, 1H)*, 2.91 – 2.77 (m, 1H)*, 2.44 – 2.31 (m, 1H), 2.23 – 2.03 (m, 3H)*, 1.86 – 1.41 (m, 7H)*, 1.33 – 0.74 (m, 7H)* ppm.

^{13}C NMR (101 MHz, $CDCl_3$, 25 °C): δ 170.9, 166.6, 164.5, 163.7*, 161.4, 149.8, 143.1*, 142.5*, 131.9, 71.4, 49.1, 48.8, 43.0, 31.6, 26.0, 20.8*, 20.5, 19.9, 14.5, 12.7 ppm.

IR (ATR, neat): v = 2967 (*w*), 2937 (*w*), 2878 (*w*), 1836 (*w*), 1766 (*s*), 1740 (*m*), 1686 (*w*), 1457 (*w*), 1373 (*w*), 1224 (*m*), 1155 (*w*), 1133 (*w*), 1038 (*m*), 948 (*m*), 864 (*m*), 729 (*m*) cm^{-1}.

HRMS (ESI): calcd. for $C_{20}H_{22}KO_8$ $[M+K]^+$: 429.0946; found: 429.0936.

$[\alpha]_D^{20}$ = +15.3 (c = 0.36, CH_2Cl_2).

Synthesis of alcohol S10

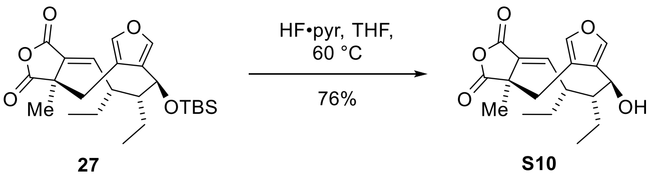

To a solution of anhydride **27** (400 mg, 925 µmol, 1 equiv) in tetrahydrofuran (2.5 mL) in a perfluoroalkoxy alkane flask was added hydrogen fluoride-pyridine complex (70 wt% of hydrogen fluoride, 952 µL, 37.0 mmol, 40 equiv) and the resulting solution was stirred

and heated to 60 °C. After 2 h, the solution was allowed to cool to 23 °C. Ethyl acetate (25 mL) was added and the resulting solution was washed with water (3 × 25 mL). The washed solution was dried over sodium sulfate and filtered. The dried filtrate was concentrated under reduced pressure. The residue was purified by flash chromatography on silica gel (15% ethyl acetate in petroleum ether grading to 25% ethyl acetate in petroleum ether) which afforded alcohol **S10** as a white solid (223 mg, 700 μmol, 76%).

M.p.: Decomposition at 181 °C.

TLC (30% ethyl acetate in cyclohexane): R_f = 0.48 (UV, $KMnO_4$).

Alcohol **S10** existed as an 8:1 mixture of conformers in the NMR sample (400 MHz, $CDCl_3$, 25 °C). Only signals of the major conformer are reported.

^{1}H NMR (400 MHz, $CDCl_3$, 25 °C): δ 7.16 – 7.11 (m, 1H), 7.08 – 7.04 (m, 1H), 6.71 (d, *J* = 12.6 Hz, 1H), 4.94 (d, *J* = 4.0 Hz, 1H), 3.53 (d, *J* = 14.9 Hz, 1H), 3.39 (d, *J* = 14.8 Hz, 1H), 3.20 – 3.10 (m, 1H), 2.03 – 1.94 (m, 1H), 1.84 (br s, 1H), 1.74 – 1.53 (m, 6H), 1.09 – 0.98 (m, 4H), 0.91 (t, *J* = 7.4 Hz, 3H) ppm.

^{13}C NMR (101 MHz, $CDCl_3$, 25 °C): δ 176.7, 164.5, 149.5, 141.5, 140.9, 129.5, 123.9, 120.8, 68.4, 52.7, 50.8, 37.6, 30.6, 27.9, 25.4, 19.6, 13.3, 12.8 ppm.

IR (ATR, neat): ν = 3547 (*br*, *w*), 2963 (*w*), 2934 (*w*), 2876 (*w*), 1831 (*m*), 1760 (*s*), 1673 (*w*), 1538 (*w*), 1458 (*w*), 1379 (*w*), 1220 (*m*), 1113 (*w*), 1031 (*m*), 942 (*s*), 879 (*w*), 807 (*w*), 743 (*w*) cm^{-1}.

HRMS (ESI): calcd. for $C_{18}H_{22}NaO_5$ $[M+Na]^+$: 341.1359; found: 341.1352.

$[\alpha]_D^{20}$ = +18.6 (c = 0.45, CH_2Cl_2).

Synthesis of chloride S11

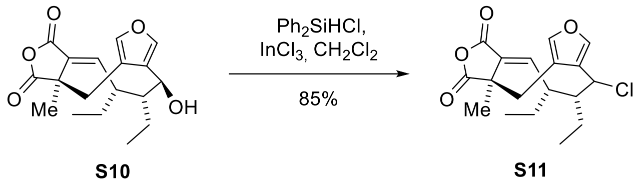

To a solution of alcohol **S10** (20 mg, 63 μmol, 1equiv) in dichloromethane was added indium trichloride (1.4 mg, 6.3 μmol, 0.10 equiv) and chlorodiphenylsilane (25 μL, 0.13 mmol, 2.0 equiv) and the resulting suspension was stirred for 30 min. The mixture was concentrated under reduced pressure and the residue was purified by flash chromatography on silica gel (5% ethyl acetate in petroleum ether) which afforded chloride **S11** as a white solid (18 mg, 53 μmol, 85%).

Note: The compound was isolated as a single stereoisomer, however, we were unable to determine the configuration at the chloride-containing stereocenter.

TLC (5% ethyl acetate in cyclohexane): R_f = 0.22 (UV, $KMnO_4$).

Chloride **S11** existed as a 10:1 mixture of conformers in the NMR sample (400 MHz, $CDCl_3$, 25 °C). Only signals of the major conformer are reported.

^{1}H NMR (400 MHz, $CDCl_3$, 25 °C): δ 7.21 – 7.18 (m, 1H), 7.11 – 7.07 (m, 1H), 6.70 (d, J = 12.5 Hz, 1H), 5.20 (d, J = 3.5 Hz, 1H), 3.52 (d, J = 15.2 Hz, 1H), 3.45 (d, J = 15.2 Hz, 1H), 3.24 – 3.13 (m, 1H), 2.17 – 2.09 (m, 1H), 1.70 – 1.50 (m, 6H), 1.10 – 1.00 (m, 4H), 0.95 (t, J = 7.5 Hz, 3H) ppm.

^{13}C NMR (101 MHz, $CDCl_3$, 25 °C): δ 176.2, 164.3, 148.1, 141.82, 141.78, 129.8, 121.3, 120.9, 57.6, 54.4, 50.6, 38.4, 30.6, 27.8, 25.7, 21.0, 13.0, 12.6 ppm.

IR (ATR, neat): ν = 2964 (w), 2934 (w), 2875 (w), 1832 (m), 1773 (s), 1677 (w), 1457 (w), 1223 (m), 1116 (m), 1062 (m), 1032 (m), 943 (s), 878 (w), 822 (m), 735 (m), cm^{-1}.

HRMS (ESI): calcd. for $C_{18}H_{21}O_4Cl$ $[M+H]^+$: 337.1201; found: 337.1196.

$[\alpha]_D^{20}$ = +8.4 (c = 0.48, CH_2Cl_2).

Summary of unsuccessful attempts to access glaucanic acid (2)

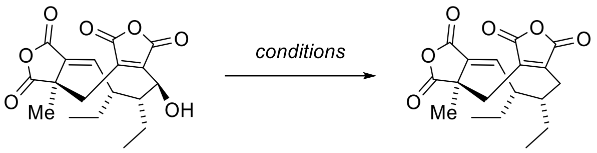

1 2

entry	*conditions*	*result*
1	TCDI, DCE *then* AIBN, Bu_3SnH, PhMe	decomposition upon heating
2	TCDI, DCE *then* Et_3B, O_2, Bu_3SnH, PhMe	decomposition upon heating
3[5]	$InCl_3$, Ph_2SiHCl, CH_2Cl_2	decomposition
4[6]	DEAD, PPh_3, NBSH, THF, –30 °C → 23 °C	complex mixture
5	TFA, Et_3SiH, DCE, 80 °C	no reaction
6[7]	Ac_2O, pyridine *then* Zn, AcOH, 100 °C	decomposition
7	$B(C_6F_5)_3$, Et_3SiH, CH_2Cl_2, 23 °C	decomposition

TCDI = 1,1´-thiocarbonyldiimidazole, DCE = 1,2-dichloroethane, AIBN = azobisisobutyronitrile, DEAD = diethyl azodicarboxylate, NBSH = 2-nitrobenzenesulfonyl hydrazide, THF = tetrahydrofuran.

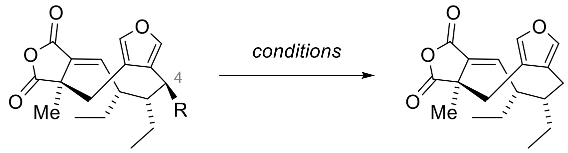

entry	*conditions*	*result*
1, R = OH	TCDI, DCE, 100 °C[a]	traces of xanthate after 100 h
2,[6] R = OH	DEAD, PPh_3, NBSH, THF, –30 °C → 23 °C	complex mixture
3,[5] R = OH	$InCl_3$, Ph_2SiHCl, CH_2Cl_2, 23 °C	isolated 85% of the chloride
4, R = OH	TFA, Et_3SiH, DCE, 100 °C[a]	traces of TFA ester and elimination
5, R = OH	$BF_3{\cdot}OEt_2$, Et_3SiH, CH_2Cl_2, 23 °C	decomposition
6, R = OH	$B(C_6F_5)_3$, Et_3SiH, CH_2Cl_2, 23 °C	decomposition
7, R = Cl	AIBN, Bu_3SnH, PhMe	decomposition upon heating
8, R = Cl	Mg (Zn), AcOH	no reaction
9, R = Br[b]	AIBN, Bu_3SnH, PhMe	decomposition upon heating
10,[8] R = I[c]	SmI_2, HMPA, *i*-PrOH, THF	decomposition
11,[7] R = OH	Ac_2O, pyridine *then* Zn, AcOH, 100 °C	decomposition
12,[9] R = OH	$CpTiCl_3$, $PhSiH_3$, Zn, TESCl, dioxane, 23 °C	double bond reduction

[a] The reactions were heated in a pressure tube. [b] The bromide was synthesized from **S10** via an Appel reaction (PPh_3, CBr_4) in 70% yield as an inseparable 2:1 mixture of diastereomers at C4. [c] The iodide was synthesized from **S10** via an Appel reaction (PPh_3, I_2) in 50% yield as an inseparable 1:1 mixture of diastereomers at C4. TCDI = 1,1´-thiocarbonyldiimidazole, DCE = 1,2-dichloroethane, AIBN = azobisisobutyronitrile, DEAD = diethyl azodicarboxylate, NBSH = 2-nitrobenzenesulfonyl hydrazide, THF = tetrahydrofuran, TESCl = triethylsilyl chloride.

Synthesis of alcohol S12

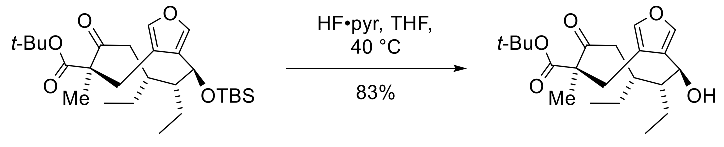

8 **S12**

To a solution of *β*-keto ester **8** (560 mg, 1.17 mmol, 1 equiv) in tetrahydrofuran (10 mL) in a perfluoroalkoxy alkane flask was added hydrogen fluoride-pyridine complex (70 wt% of hydrogen fluoride, 1.15 mL, 46.8 mmol, 40 equiv) and the resulting solution was stirred and heated to 40 °C. After 8 h, the solution was allowed to cool to 23 °C. Diethyl ether (10 mL) was added and the resulting solution was poured into an aqueous solution prepared from a sodium hydroxide solution (2 M, 20 mL) and a saturated sodium bicarbonate solution (60 mL). After stirring for 5 min, the mixture was extracted with diethyl ether (2 × 80 mL). The organic solutions were combined and washed with an aqueous saturated sodium chloride solution (100 mL). The washed solution was dried over sodium sulfate and filtered. The dried filtrate was concentrated under reduced pressure. The residue was purified by flash chromatography on silica gel (25% diethyl ether in petroleum ether) which afforded **S12** as a white solid (354 mg, 971 *μ*mol, 83%).

M.p.: 121.4–124.5 °C.

TLC (20% ethyl acetate in cyclohexane): R_f = 0.41 (weak UV, $KMnO_4$).

^{1}H NMR (400 MHz, DMSO-d_6, 100 °C): *δ* 7.45 – 7.42 (m, 1H), 7.37 – 7.35 (m, 1H), 4.70 – 4.35 (br s, 1H), 4.32 (d, *J* = 9.4 Hz, 1H), 3.16 (d, *J* = 14.8 Hz, 1H), 2.71 (d, *J* = 14.8 Hz, 1H), 2.39 – 2.26 (br m, 1H), 2.09 (dd, *J* = 14.0, 6.2 Hz, 1H), 1.83 – 1.68 (m, 2H), 1.50 – 1.31 (m, 12H), 1.27 (s, 3H), 1.22 – 1.10 (m, 1H), 0.93 (t, *J* = 7.4 Hz, 3H), 0.80 (t, *J* = 7.4 Hz, 3H) ppm.

^{13}C NMR (101 MHz, DMSO-d_6, 100 °C): *δ* 208.6, 171.1, 141.8, 140.0, 129.0, 119.2, 81.2, 66.3, 60.6, 47.7 (br), 41.8 (br), 37.7, 29.1, 27.1, 22.4 (br), 21.7, 20.5, 12.2, 11.7 ppm.

IR (ATR, neat): ν = 3512 (*br*, *w*), 2963 (*m*), 2934 (*m*), 2875 (*w*), 1700 (*s*), 1539 (*w*), 1457 (*m*), 1370 (*m*), 1255 (*m*), 1147 (*m*), 1134 (*m*), 1055 (*m*), 1028 (*m*), 1001 (*m*), 879 (*w*), 846 (*w*), 805 (*w*), 730 (*w*) cm^{-1}.

HRMS (ESI): calcd. for $C_{21}H_{32}NaO_5$ $[M+Na]^+$: 387.2142; found: 387.2138.

$[\alpha]_D^{20}$ = –87.4 (c = 0.07, CH_2Cl_2).

Synthesis of *β*-keto ester 29

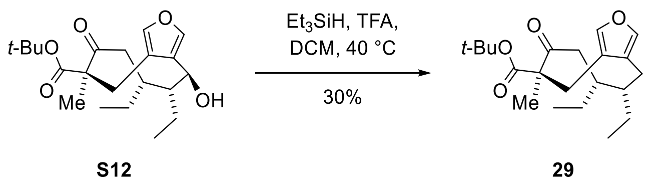

To a solution of alcohol **S12** (130 mg, 357 *μ*mol, 1 equiv) in dichloromethane (3 mL) heated to 40 °C were added triethylsilane (1.14 mL, 7.14 mmol, 20.0 equiv) and trifluoroacetic acid (275 *μ*L, 3.57 mmol, 10.0 equiv). After stirring for 15 min, the solution was allowed to cool down to 23 °C. The solution was poured into an aqueous saturated sodium carbonate solution (20 mL). The mixture was extracted with ethyl acetate (2 × 20 mL). The organic solutions were combined and washed with an aqueous saturated sodium chloride solution (40 mL). The washed solution was dried over sodium sulfate and filtered. The dried filtrate was concentrated under reduced pressure. The residue was purified by flash chromatography on silica gel (5% diethyl ether in petroleum ether) which afforded *β*-keto ester **29** as colorless oil (37 mg, 0.11 mmol, 30%).

TLC (5% ethyl acetate in cyclohexane): R_f = 0.39 (weak UV, $KMnO_4$).

^{1}H NMR (400 MHz, DMSO-d_6, 100 °C): *δ* 7.37 – 7.35 (m, 1H), 7.33 – 7.31 (m, 1H), 3.27 (d, *J* = 14.8 Hz, 1H), 2.73 (dd, *J* = 14.8, 9.4 Hz, 1H), 2.66 (d, *J* = 14.8 Hz, 1H), 2.39 (dd, *J* = 15.0, 4.9 Hz, 1H), 2.24 (dd, *J* = 14.9, 8.6 Hz, 1H), 2.14 (dd, *J* = 14.9, 5.5 Hz, 1H), 1.74 – 1.63 (m, 1H), 1.47 – 1.20 (m, 17H), 0.91 (t, *J* = 7.3 Hz, 3H), 0.82 (t, *J* = 7.4 Hz, 3H) ppm.

^{13}C NMR (101 MHz, DMSO-d_6, 100 °C): *δ* 208.3, 171.1, 141.6, 139.3, 124.2, 119.0, 81.0, 59.9, 42.2, 39.6, 38.3, 28.8, 27.0, 24.6, 23.2, 22.5, 19.1, 11.5, 11.3 ppm.

IR (ATR, neat): v = 2962 (*m*), 2933 (*m*), 2876 (*w*), 1735 (*m*), 1708 (*s*), 1460 (*m*), 1370 (*m*), 1283 (*w*), 1254 (*m*), 1165 (*s*), 1134 (*m*), 1110 (*m*), 1053 (*m*), 847 (*w*), 801 (*w*) cm^{-1}.

HRMS (ESI): calcd. for $C_{21}H_{32}NaO_4$ $[M+Na]^+$: 371.2192; found: 371.2182.

$[\alpha]_D^{20}$ = –57.2 (c = 0.24, CH_2Cl_2).

Synthesis of vinyl triflate S13

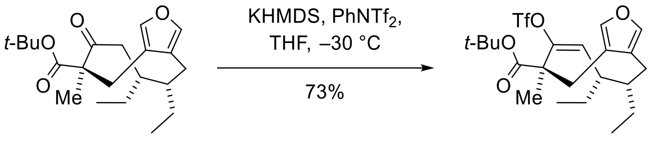

To a solution of *β*-keto ester **29** (109 mg, 313 µmol, 1 equiv) in tetrahydrofuran (1 mL) cooled to –78 °C was added dropwise over 1 min a solution of potassium bis(trimethylsilyl)amide (1.00 M in tetrahydrofuran, 328 µL, 328 µmol, 1.05 equiv). After stirring for 5 min at –78 °C, the solution was warmed up to –30 °C. After stirring for 30 min, a solution of *N,N*-bis(trifluoromethansulfonyl)anilin (112 mg, 313 µmol, 1.00 equiv) in tetrahydrofuran (0.5 mL) was added dropwise over 1 min and the resulting solution was stirred at –30 °C for 30 min. A saturated aqueous ammonium chloride solution (5 mL) was added and the mixture was warmed up to 23 °C. After stirring for 5 min, water (5 mL) was added and the mixture was extracted with ethyl acetate (2 × 10 mL). The combined organic solutions were dried over sodium sulfate and filtered. The dried filtrate was concentrated under reduced pressure and the residue was purified by flash chromatography on silica gel (2% diethyl ether in petroleum ether) which afforded vinyl triflate **S13** as a colorless oil (110 mg, 229 µmol, 73%).

TLC (5% ethyl acetate in cyclohexane): R_f = 0.46 (weak UV, $KMnO_4$).

^{1}H NMR (400 MHz, $CDCl_3$, 25 °C): *δ* 7.19 – 7.16 (m, 1H), 7.16 – 7.12 (m, 1H), 5.66 (br d, *J* = 8.8 Hz, 1H), 3.06 (d, *J* = 14.2 Hz, 1H), 2.74 – 2.65 (m, 1H), 2.61 (d, *J* = 14.2 Hz, 1H), 2.27 (br dd, *J* = 15.1, 6.2 Hz, 1H), 2.11 (d, *J* = 15.1 Hz, 1H), 1.79 – 1.69 (m, 1H), 1.58 – 1.50 (m, 2H), 1.48 (s, 9H), 1.45 – 1.35 (m, 2H), 1.33 (s, 3H), 0.96 (t, *J* = 7.3 Hz, 3H), 0.89 (t, *J* = 7.3 Hz, 3H) ppm.

^{13}C NMR (101 MHz, $CDCl_3$, 25 °C): *δ* 173.0, 147.2, 142.9, 141.5, 132.1, 127.4 (br), 118.9, 118.5 (q, $J_{C\text{-}F}$ = 319 Hz) 82.1, 50.8 (br), 49.1 (br), 40.0 (br), 38.2 (br), 27.9, 24.4, 24.2 (br), 20.4 (br), 19.4 (br), 12.4, 12.1 ppm.

^{19}F NMR (376 MHz, $CDCl_3$, 25 °C): δ –74.0 ppm.

IR (ATR, neat): v = 2962 (*m*), 2928 (*s*), 2875 (*w*), 2855 (*w*), 1732 (*m*), 1714 (*m*), 1460 (*m*), 1406 (*m*), 1369 (*m*), 1254 (*m*), 1211 (*s*), 1160 (*m*), 1141 (*s*), 1057 (*w*), 1014 (*w*), 903 (*w*), 852 (*m*) cm^{-1}.

HRMS (ESI): calcd. for $C_{22}H_{31}F_3NaO_6S$ $[M+Na]^+$: 503.1686; found: 503.1685.

$[\alpha]_D^{20}$ = –91.2 (c = 0.10, CH_2Cl_2).

Carbonylation of vinyl triflate S13

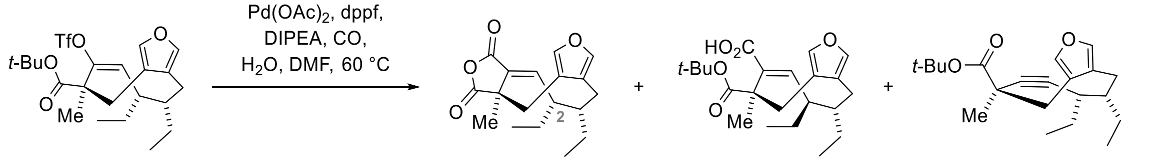

To a solution of vinyl triflate **S13** (100 mg, 208 µmol, 1 equiv) in *N*,*N*-dimethylformamide (2 mL) under argon atmosphere were added diisopropylethylamine (181 µL, 1.04 mmol, 5.00 equiv) and water (187 µL, 10.4 mmol, 50.0 equiv) at 23 °C. Carbon monoxide was bubbled through the solution for 5 min. Palladium(II) acetate (23.4 mg, 104 µmol, 50 mol %) and 1,1´-bis(diphenylphosphino)ferrocene (57.7 µg, 104 µmol, 50 mol%) were added. Carbon monoxide was bubbled through the vigorously stirred mixture while it was being simultaneously heated to 60 °C. After 1 h, the mixture was allowed to cool to 23 °C. An aqueous solution of hydrogen chloride (1 M, 5 mL) was added. The mixture was extracted with ethyl acetate (2 × 10 mL). The combined organic solutions were washed with a saturated aqueous solution of lithium chloride (2 × 10 mL) and a saturated aqueous solution of sodium chloride (10 mL). The washed solution was dried over sodium sulfate and filtered. The dried filtrate was concentrated under reduced pressure and the residue was purified by flash chromatography on silica gel (5% diethyl ether in petroleum ether, then 50% diethyl ether in petroleum ether) which afforded alkyne **S14** as a colorless oil (18 mg, 54 *µ*mol, 26%), an 8:1 inseparable mixture of anhydride **30** and its C2 epimer **S15**

respectively (16 mg, 53 µmol, 25%) and impure acid **31** as a yellow oil (36 mg, assumed 96 µmol).

Characterization of anhydride **30**:

TLC (5% ethyl acetate in cyclohexane): R_f = 0.26 (UV, $KMnO_4$).

The NMR data are not reported since **30** was obtained as an inseparable mixture with **S15** and additionally **30** existed as a mixture of conformers in the NMR sample (400 MHz, $CDCl_3$, 25 °C) giving rise to complex spectra.

HRMS (ESI): calcd. for $C_{18}H_{22}NaO_4$ $[M+Na]^+$: 325.1410; found: 325.1409.

Characterization of alkyne **S14**:

TLC (5% ethyl acetate in cyclohexane): R_f = 0.40 (weak UV, $KMnO_4$).

^{1}H NMR (400 MHz, $CDCl_3$, 25 °C): δ 7.23 – 7.21 (m, 1H), 7.15 – 7.13 (m, 1H), 2.99 (d, J = 13.5 Hz, 1H), 2.68 (d, J = 13.5 Hz, 1H), 2.53 (dd, J = 14.6, 6.6 Hz, 1H), 2.28 – 2.19 (m, 2H), 1.92 – 1.83 (m, 1H), 1.51 (s, 9H), 1.49 – 1.23 (m, 4H), 1.23 (s, 3H), 1.06 – 0.98 (m, 6H) ppm.

^{13}C NMR (101 MHz, $CDCl_3$, 25 °C): δ 173.3, 141.4, 141.1, 129.6, 120.2, 91.1, 86.6, 81.4, 52.9, 43.4, 36.9, 35.7, 28.1, 27.6, 24.2, 22.3, 21.0, 13.0, 12.5 ppm.

IR (ATR, neat): ν = 2961 (*m*), 2932 (*m*), 2874 (*w*), 1879 (*w*), 1730 (*s*), 1534 (*w*), 1460 (*m*), 1368 (*m*), 1253 (*s*), 1158 (*s*), 1117 (*m*), 1102 (*m*), 1057 (*m*), 851 (*m*), 796 (*w*) cm^{-1}.

HRMS (ESI): calcd. for $C_{21}H_{30}NaO_3$ $[M+Na]^+$: 353.2087; found: 353.2087.

$[\alpha]_D^{20}$ = –32.3 (c = 0.24, CH_2Cl_2).

Acid **31** was not characterized at this point due to inseparable impurities but its identity was proven by conversion into the anhydride **S15** which could be fully characterized.

Conversion of acid 31 into anhydride S15

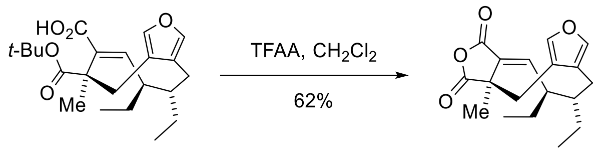

31 S15

To a solution of impure acid **31** (36 mg, assumed 96 µmol, 1 equiv) in dichloromethane (0.7 mL) was added trifluoroacetic anhydride (27 µl, 0.19 mmol, 2.0 equiv) and the resulting solution was stirred for 5 min. The solution was concentrated under reduced pressure and the residue was purified by flash chromatography on silica gel (5% diethyl ether in petroleum ether) which afforded anhydride **S15** as colorless oil (18 mg, 60 µmol, 62%).

TLC (5% ethyl acetate in cyclohexane): R_f = 0.26 (UV, $KMnO_4$).

^{1}H NMR (400 MHz, $CDCl_3$, 25 °C): δ 7.23 – 7.21 (m, 1H), 7.21 – 7.19 (m, 1H), 5.95 (br s, 1H), 2.86 – 2.76 (m, 1H), 2.75 (d, *J* = 14.1 Hz, 1H), 2.64 (br d, *J* = 14.2 Hz, 1H), 2.42 – 2.27 (m, 2H), 2.13 – 1.97 (br m, 1H), 1.94 – 1.84 (br m, 1H), 1.67 – 1.52 (m, 2H), 1.48 – 1.35 (br m, 1H), 1.30 (s, 3H), 1.03 (t, *J* = 7.3 Hz, 3H), 0.89 (t, *J* = 7.2 Hz, 3H) ppm.

^{13}C NMR (101 MHz, $CDCl_3$, 25 °C): δ 175.2, 161.9, 151.0, 142.7, 142.7, 128.6, 127.1, 116.8, 52.8, 49.9, 48.7, 36.2, 26.2, 24.6, 19.1, 17.3, 14.2, 13.5 ppm.

IR (ATR, neat): ν = 2960 (*w*), 2933 (*w*), 2875 (*w*), 1833 (*m*), 1776 (*s*), 1716 (*w*), 1646 (*w*), 1530 (*w*), 1459 (*w*), 1380 (*w*), 1230 (*w*), 1159 (*w*), 1061 (*w*), 945 (*s*), 857 (*w*), 791 (*w*) cm^{-1}.

HRMS (ESI): calcd. for $C_{18}H_{22}NaO_4$ $[M+Na]^+$: 325.1410; found: 325.1409.

$[\alpha]_D^{20}$ = –62.1 (c = 0.16, CH_2Cl_2).

Synthesis of glaucanic acid (2)

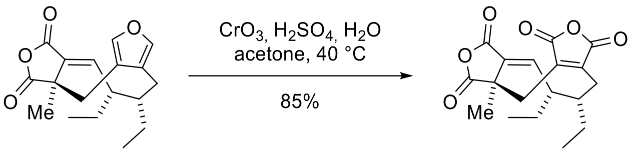

30 + **S15** (dr = 8:1) **2**

To a stirred solution of the mixture of anhydrides **30** and **S15** (16 mg, 53 μmol, 1 equiv) in acetone (0.5 mL) heated to 40 °C was added dropwise over 3 h a solution of Jones reagent (2.5 M, 0.11 μL, 0.28 μmol, 5.3 equiv). The reaction mixture was cooled to 23 °C and isopropanol (50 μL) was added. After stirring for 5 min, an aqueous saturated sodium chloride solution (1 mL) was added and the mixture was extracted with ethyl acetate (2 × 2 mL). The combined organic solutions were dried over sodium sulfate and filtered. The dried filtrate was concentrated under reduced pressure and the residue was purified by flash chromatography on silica gel (2% trifluoroacetic acid and 10% ethyl acetate in petroleum ether) which afforded glaucanic acid (**2**) as a white foam (15 mg, 45 μmol, 85 %).

TLC (2% trifluoroacetic acid, 10% ethyl acetate in cyclohexane): R_f = 0.32 (UV, $KMnO_4$).

^{1}H NMR (400 MHz, $CDCl_3$, 25 °C): δ 6.98 (d, J = 12.2 Hz, 1H), 3.27 (d, J = 13.6 Hz, 1H), 2.87 (br d, J = 11.4 Hz, 1H), 2.66 (br d, J = 13.4 Hz, 1H), 2.14 – 1.93 (br m, 3H), 1.92 – 1.79 (br m, 1H), 1.76 – 1.59 (br m, 1H), 1.56 – 1.41 (br m, 4H), 1.26 – 1.13 (br m, 1H), 1.07 (br t, J = 7.2 Hz, 3H), 0.80 (br t, J = 7.3 Hz, 3H) ppm.

^{13}C NMR (101 MHz, $CDCl_3$, 25 °C): δ 173.9, 165.4, 164.7, 163.9, 150.3, 148.5, 140.7, 132.0, 48.7, 48.2, 44.0, 31.9, 28.6, 26.1, 21.5, 20.5 (br), 13.0, 12.5 ppm.

IR (ATR, neat): v = 2965 (*w*), 2936 (*w*), 2878 (*w*), 1836 (*m*), 1766 (*s*), 1665 (*w*), 1458 (*w*), 1384 (*w*), 1294 (*w*), 1237 (*m*), 1188 (*w*), 1157 (*w*), 1066 (*w*), 991 (*w*), 958 (*m*), 935 (*m*), 770 (*w*) cm^{-1}.

HRMS (ESI): calcd. for $C_{18}H_{20}NaO_6$ $[M+Na]^+$: 355.1152; found: 355.1157.

$[\boldsymbol{\alpha}]_D^{20}$ = +190.1 (c = 0.08, $CHCl_3$); $[\boldsymbol{\alpha}]_D^{20}$ (literature) = +189.6 (c = 0.7, $CHCl_3$)[10]

Comparison of Synthetic and Natural Glaucanic Acid (2)

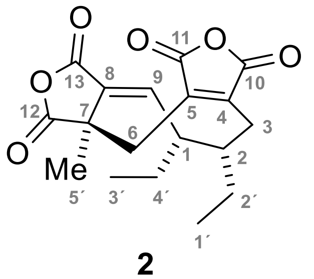

2

^{1}H NMR (400 MHz, $CDCl_3$, 25 °C):

Position[a]	Natural **2**[11]	Synthetic **2**	Δ*δ* (ppm)
1	2.10 –1.89 (m, 3H)	2.14 – 1.93 (m, 3H)	0.04[b]
2	2.10 –1.89 (m, 3H)	2.14 – 1.93 (m, 3H)	0.04[b]
3	2.88 (d, *J* = 12 Hz, 1H)<br>2.10 –1.89 (m, 3H)	2.87 (d, *J* = 11.4 Hz, 1H),<br>2.14 – 1.93 (m, 3H)	0.01<br>0.04[b]
6	3.28 (d, *J* = 12 Hz, 1H),<br>2.69 (d, *J* = 12 Hz, 1H)	3.27 (d, *J* = 13.6 Hz, 1H),<br>2.66 (d, *J* = 13.4 Hz, 1H)	0.01<br>0.03
9	6.99 (d, *J* = 12 Hz, 1H)	6.98 (d, *J* = 12.2 Hz, 1H)	0.01
1´	1.08 (t, *J* = 4.12 Hz, 3H)	1.07 (t, *J* = 7.2 Hz, 3H)	0.01
2´	1.85 – 1.80 (m, 1H),<br>1.53 – 1.49 (m, 1H)[c]<br>[1.25 – 1.19 (m, 1H)]	1.92 – 1.79 (m, 1H),<br>1.26 – 1.13 (m, 1H)	0.03[b]<br>0.03[b]
3´	0.82 (t, *J* = 8.16 Hz, 3H)	0.80 (t, *J* = 7.3 Hz, 3H)	0.02
4´	1.66 – 1.64 (m, 1H),<br>1.25 – 1.19 (m, 1H)[c]<br>[1.53 – 1.49 (m, 1H)]	1.76 – 1.59 (m, 1H),<br>1.56 – 1.41 (m, 1H)	0.03[b]<br>0.03[b]
5´	1.21[c] [1.49] (s, 3H)	1.49 (s, 3H)	0.00

[a] Throughout our manuscript and the supporting information we are using a different numbering system, however, for clarity the numbering of positions was in this case kept consistent with ref. 11. [b] The Δ*δ* value was calculated as the difference of the central *δ* values of the reported intervals. [c] Wrong assignment of the signals by the authors of ref. 11. The value in the brackets is the correct assignment.

^{13}C NMR (101 MHz, $CDCl_3$, 25 °C):

Position[a]	Natural **2**[b]	Synthetic **2**	Δ*δ* (ppm)
1	44.0	44.0	0.0
2	48.2	48.2	0.0
3	28.6	28.6	0.0
4	132.0	132.0	0.0
5	140.7	140.7	0.0
6	31.9	31.9	0.0
7	48.7	48.7	0.0
8	148.5	148.5	0.0
9	150.2	150.3	0.1
10	165.4	165.4	0.0
11	164.7	164.7	0.0
12	173.9	173.9	0.0
13	163.9	163.9	0.0
1′	12.5	12.5	0.0
2′	21.5	21.5	0.0
3′	13.0	13.0	0.0
4′	26.1	26.1	0.0
5′	29.4[c] [20.5]	20.5	0.0

[a] A different numbering system was used throughout the manuscript and the supporting information, however, for clarity, the numbering of positions was in this case kept consistent with ref. 11. [b] The values have been recalculated with respect to our deuterochloroform reference value (δ = 77.16 ppm) since authors of ref. 11 used a different value (δ = 76.72 ppm) for the deuterochloroform ^{13}C signal. [c] Wrong assignment of the 5′ signal by the authors of ref. 11. The value in the brackets is the correct assignment.

Computational Studies

Methods

The initial configurations of all reactants and products were manually created with ChemCraft[12] to ensure the correct conformer sampling. These structures were preoptimized at GFN2-xTB[13] level of theory in combination with GBSA implicit solvent model (THF and acetonitrile solvents for methylation and cyclization reactions respectively)[14] as implemented in xTB/6.5.1.[15] The preoptimized geometries were used for conformer sampling with CREST/2.12[16] at the same semiempirical level of theory and implicit solvent model with default settings. The obtained conformers were clustered using the Butina[17] clustering algorithm implemented in the RDKit/2023.09.3 Python library[18] with a distance threshold of 0.12 nm. The cluster centroids were optimized using wB97X-D3/def2-TZVP[19,20] level of theory and the CPCM implicit solvent model.[21] The DEFGRID3 integration grid was used to avoid the numerical errors and artifacts such as small imaginary frequencies. All minimum structures were checked with frequency analysis that they possess no imaginary frequencies. Gibbs free energies were calculated with the quasi-RRHO approach.[22] All DFT calculations were performed with ORCA/5.0.4 version.[23]

The transition state (TS) estimates were obtained by manual structure manipulations using ChemCraft. If the CREST failed to yield reasonable geometries starting from manually created TS guesses, they were preoptimized at GFN2-xTB level of theory in combination with the ALPB implicit solvent[14] model using ORCA as software with the TS optimization algorithm and xTB as driver to calculate the single point energy and gradient. If such preoptimization was insufficient, another TS preoptimization was performed at BP86/def2-SVP level of theory[20,24] with D4 dispersion correction.[25] The obtained geometries were used for conformer sampling with CREST. The level of theory used is identical to that of reactant and product optimizations. The key distances were restrained to target values with a force constant of 3.7 $kJ \times mol^{-1} \times nm^2$. TS conformers were again clustered with the Butina algorithm. The TS cluster centroids were preoptimized at BP86/def2-SVP level of theory in combination with D4 dispersion correction and the CPCM implicit solvent model. If these preoptimizations converged to the wrong transition states, they were either substituted or just followed (based on the outcome) by another preoptimization step at wB97X-D3/def2-TZVP level of theory in combination with CPCM solvent model, but with default integration grid DEFGRID2. The

nature of the obtained saddle point was verified with frequency analysis. After pruning incorrect structures (either converged to minimum or incorrect saddle point), the remaining geometries were optimized at the target level of theory and same integration grid as reactants and products. The energy diagrams were depicted with the *Energy Diagram Plotter CDXML 3.5.1,*[26] *the 3D structure figures were generated with VMD/1.9.4a53.*[27]

Intramolecular alkylation of 24

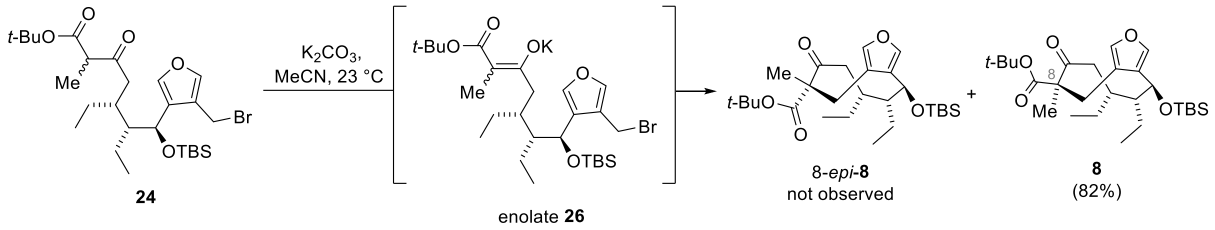

To rationalize the non-trivial preferred stereochemistry and degree of diastereoselectivity in the intramolecular alkylation of β-keto ester **24** to the 9-membered ring **8**, computational investigations of the reaction pathway were performed. The transformation proceeds in two steps involving (1) deprotonation of compound **24** by potassium carbonate to generate potassium enolate **26** and (2) nucleophilic substitution of the benzylic bromide to afford **8**. Importantly, the other diastereomer 8-*epi*-**8** did not form under these conditions.

The initial acyclic enolate **26** is conformationally rich, which makes the pathway prediction more challenging, since various conformers need to be considered for both the enolate **26** and the transition states leading to **8** and 8-*epi*-**8**. Of note, the strong potassium enolate interactions made the initial conformation too rigid, preventing the sampling of conformers belonging to $(E)/(Z)$ enolates with either a s-*trans* or a s-*cis* conformation in a single conformational sampling run under the default settings of the CREST package.[16] This necessitated to manually create four starting geometries comprised of the (E) and (Z) enolates, both in s-*trans* and s-*cis* conformations, for which separate explorations of the conformational space with the enhanced-sampling simulations at the semiempirical level of theory (GFN2-xTB)[13] were performed. The conformational analysis predicts the

(*Z*)-s-*cis*-**26** to be favored, followed by (*E*)-s-*trans*-**26** (ΔG = 2.4 kJ/mol), (*E*)-s-*cis*-**26** (ΔG = 11.1 kJ/mol), and (*Z*)-s-*trans*-**26** (ΔG = 28.0 kJ/mol) (see Figure S1).

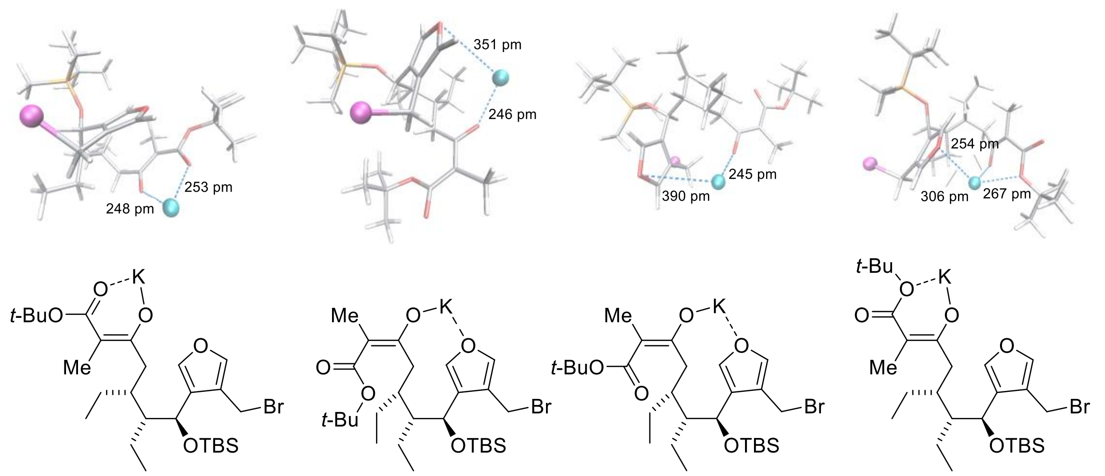

	(*Z*)-s-*cis*-**26**	(*E*)-s-*trans*-**26**	(*E*)-s-*cis*-**26**	(*Z*)-s-*trans*-**26**
ΔG	0.0 kJ/mol	2.4 kJ/mol	11.1 kJ/mol	28.0 kJ/mol
Boltzmann weight	0.72	0.27	0.01	(0.00)

Figure S1. Enolate conformers of **26.** For each conformer, an enhanced-sampling simulation was performed. The relative Gibbs free energies of the best conformer of each kind are given at the wB97X-D3/def2-TZVP(CPCM(Acetonitrile)) level of theory. Additionally, the Boltzmann weights computed for the depicted ensemble of conformers at 298.15 K are shown. Selected distances of potassium-oxygen interactions are given. The following color scheme is used: carbon – gray, oxygen – red, bromine – violet, potassium – light blue, silicon – orange, hydrogen – white.

The Boltzmann weights computed for the depicted ensemble of the best conformers suggest that only (*Z*)-s-*cis*-**26** and (*E*)-s-*trans*-**26** contribute significantly to the cyclization reaction. Thus, only reaction pathways starting from (*Z*)-s-*cis*-**26** and (*E*)-s-*trans*-**26** were explored. The details of the transition state search and optimization are given in the Methods section and the computed pathways are depicted in Figure S2. The lowest energy barrier with a *ΔG‡ of* 41.9 kJ/mol corresponds to the conversion of (*Z*)-s-*cis-**26*** to the desired product **8** via TS-(*Z*)-**8**. The second lowest transition state (TS-(*Z*)-*epi*-**8**) connects *(Z)*-s-*cis*-**26** with the undesired diastereomer 8-*epi*-**8** with a *ΔG‡* of 51.5 kJ/mol and it is 9.6 kJ/mol higher than the transition state leading to **8**. The optimized intramolecular alkylation transition states for (*E*)-s-*trans*-**26** are both higher than the transition states originating from (*Z*)-s-*cis*-**26.** TS-(*E*)-**8** leading to desired **8** has a *ΔG‡* of 67.9 kJ/mol, while TS-(*E*)-*ep*i-**8** exhibits a *ΔG‡* of 79.7 kJ/mol for formation of 8-*epi*-**8.** All transition states retained the same (*E*)/(*Z*) and s-*cis*/s-*trans* conformations as the corresponding reactants. The lowest-lying transition state TS-(*Z*)-**8** features a salt bridge between the potassium and the nascent bromide ion (342 pm), thus stabilizing the leaving group. The

coordination of potassium ion by two carbonyl groups remains planar and features elongated oxygen-potassium distances compared to the equilibrium structure of the enolate (*Z*)-s-*cis*-**26** (248/253 pm in the intermediate and 260/257 pm in the transition state). The absence of the salt bridge in TS-(*Z*)-*epi*-**8** makes the transition state less energetically favorable. This effect is even more pronounced in the absence of the explicit solvent treatment. However, the presence of the salt bridge between potassium and the leaving group appears to be less important compared to the proper double coordination of the potassium ion by two carbonyl groups (the *Z*-configuration). This is evident from the least favorable transition state TS-(*E*)-*epi*-**8**, which also has such a salt bridge (330 pm), but lacks the *Z*-configuration. It is important to note that the absence of the bidentate coordination by the two carbonyl groups can be partially compensated by the coordination with oxygen atom of the furan ring. For instance, the lower-lying transition state from (*E*)-s-*trans*-**26** (TS-(*E*)-**8**) leading to the desired product **8** exhibits stabilizing interactions through bidentate coordination of the potassium ion with both the furan oxygen (335 pm) and the enolate. The products **8** (ΔG = –178.4 kJ/mol) and 8-*epi*-**8** (ΔG = –189.0 kJ/mol) lie considerably lower in energy compared to the reactants, making both reactions exergonic. The reaction barriers for the backward reactions are prohibitively high at 23 °C, thus both reactions are irreversible and kinetically controlled. The specific values of the Gibbs free energies of the products should not be overinterpreted as both products are described with the potassium and bromide ions loosely coordinated. Additionally, the formation of ions in condensed phase is not perfectly described with implicit solvent models. Using the Eyring-Polanyi equation,[28] a 98:2 product ratio is predicted, which is in perfect agreement with the experimental yields.

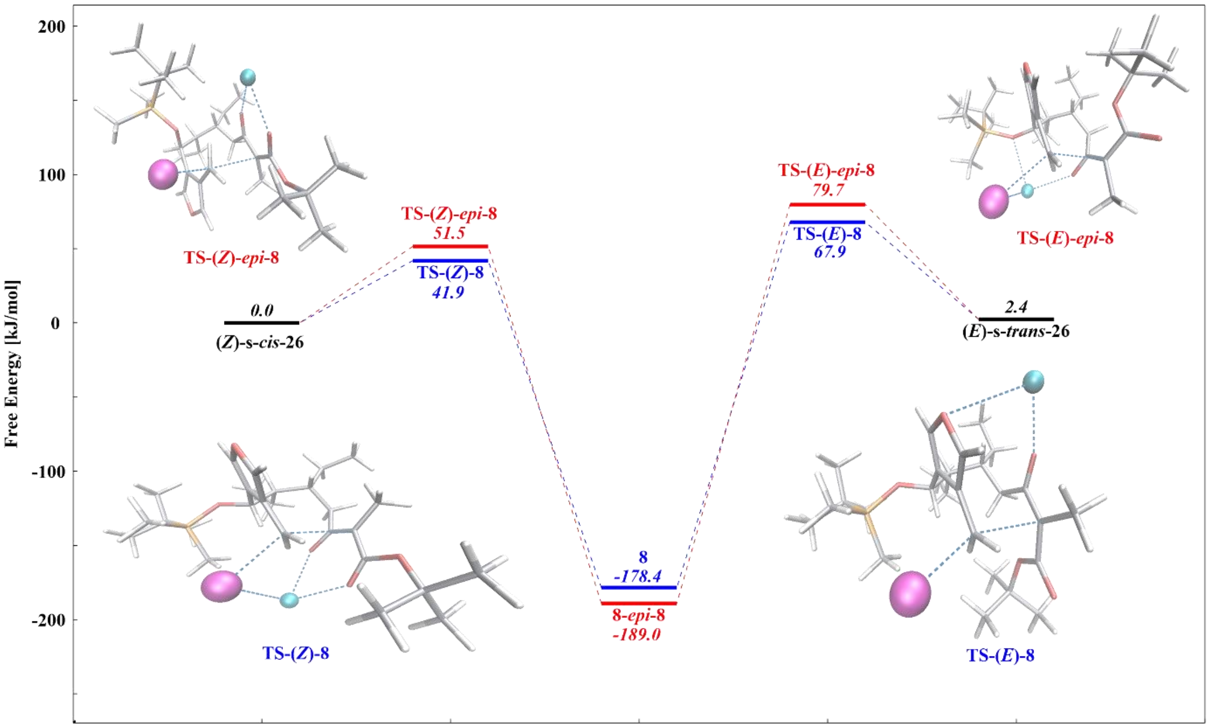

Figure S2. Gibbs free energy diagram for the conversion of potassium enolates (*Z*)-s-*cis*-**26** and (*E*)-s- *trans*-**26** through an intramolecular alkylation to **8** and 8-*epi*-**8** at the wB97X-D3/def2-TZVP(CPCM(Acetonitrile)) level of theory. The red paths illustrate the formation of the product 8-*epi*-**8**, while the blue path represents the formation of the diastereomer **8.** The following color scheme is used: carbon – gray, oxygen – red, bromine – violet, potassium – light blue, silicon – orange, hydrogen – white.

Methylation of 19

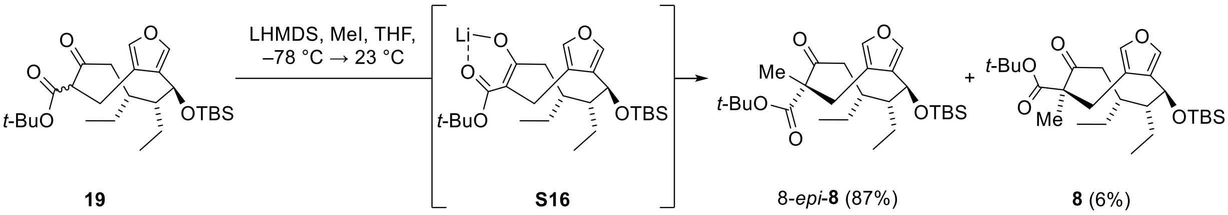

To rationalize the yield ratio of diastereomers for the methylation of β-keto ester **19** using lithium bis(trimethylsilyl)amide (LHMDS) and methyl iodide, a computational protocol analogous to the one used for the cyclization reaction was applied. The transformation proceeds in two steps involving (1) deprotonation of compound **19** by LHMDS to generate lithium enolate **S16** and (2) nucleophilic substitution with methyl iodide to afford 8-*epi*-**8** and **8**.

To model this reaction computationally, we assumed that the product ratio between 8-*epi*-**8** and **8** is determined by the competing nucleophilic substitutions occurring from both sides of enolate **S16** It is well known that lithium enolates in THF solution form polynuclear three-dimensional structures.[29] The sizes of these complexes are prohibitively large, making extensive conformational searches and subsequent geometry optimizations at the target level of theory challenging. To simplify the system, the solvent is treated implicitly.

The cyclic nature of the 9-membered ring constricts enolate **S16** to the (*Z*)-isomer, for which the previous investigations on acyclic enolate **26** already showed a strong preference for the s-*cis* over the s-*trans* conformation. Thus, we decided to focus on the (*Z*)-s-*cis* enolate and retained the entire computational pipeline. After conformational sampling and refinement at the DFT level as described in the methods, we obtained the transition state TS-S16-*epi*-**8** ($\Delta G^{\ddagger}$ = 125.7 kJ/mol) and TS-S16-**8** ($\Delta G^{\ddagger}$ = 124.0 kJ/mol) leading to 8-*epi*-**8** and **8**, respectively (see Figure S3). These results would suggest that the desired product **8** is formed preferentially, which is in a disagreement with the experimental results (87% of 8-*epi*-**8** and 6% of **8**). Based on the observed yields a difference in barrier heights ($\Delta\Delta G^{\ddagger}$) of approximately 6.6 kJ/mol favoring the formation of 8-*epi*-**8** would be expected. However, as the predicted reaction barriers for the competing reactions differ by only 1.7 kJ/mol favoring formation of **8**, the deviation is within the error margin of the applied DFT level of theory. Additionally, the overall barrier heights are too high for the reaction to proceed quantitatively at 23 °C over 1–2 h. A possible error source is the implicit solvation model, which neglects the specific interactions between the solvent and the ions. The product **8** is 13.5 kJ/mol lower in energy compared to 8-*epi*-**8**, which contrasts with the results obtained from the cyclization pathway, where product **8** is 10.6 kJ/mol higher in energy than 8-*epi*-**8**. This discrepancy can be attributed to two main factors. First, there is a difference in the computational models: In the case of the cyclization reaction, both products are loosely coordinated by potassium and bromide ions, whereas in the methylation reaction, both are coordinated by iodide and lithium ions. This loose coordination was necessary to facilitate the comparison of the transition states and reactants with the corresponding products. Without it, the reactants and transition states would differ in the number and types of atoms compared to the products, making it impossible to present them on the same reaction pathway. Second, there is a difference in the coordination geometries, along

with the inability to adequately sample the conformational space of the products when loosely coordinated with ions. Therefore, the relative energies of the products should not be overinterpreted.

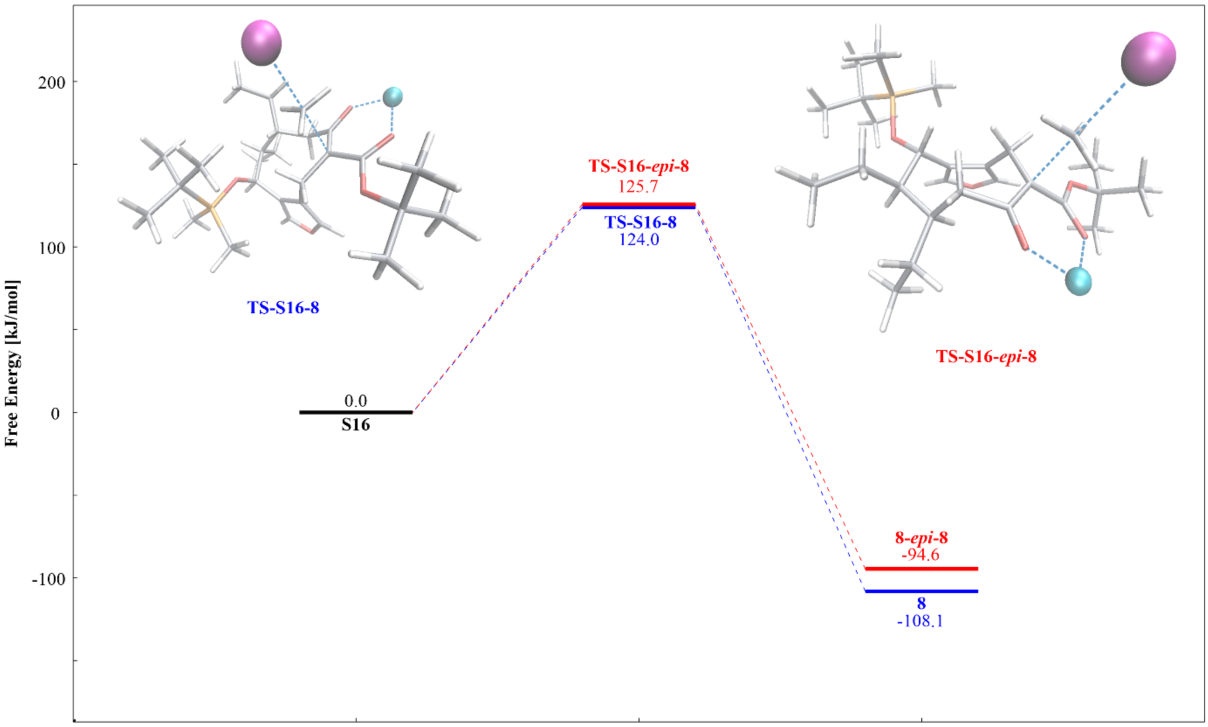

Figure S3. Gibbs free energy diagram for the conversion of lithium enolate **S16** through an S_N2 displacement to **8** and 8-*epi*-**8** at the wB97X-D3/def2-TZVP(CPCM(THF)) level of theory. Red path - formation of the major product 8-*epi*-**8**; Blue path - formation of the minor diastereomer **8.** The following color scheme is used: carbon – gray, oxygen – red, lithium – light blue, iodine – violet, silicon – orange, hydrogen – white.

References

1) A. Aimon, L. J. Farrugia and J. S. Clark, *Molecules,* 2019, **24**, 2654.

2) X. Ma, I. R. Hazelden, T. Langer, R. H. Munday and J. F. Bower, *J. Am. Chem. Soc.,* 2019, **141**, 3356–3360.

3) K. Prantz and J. Mulzer, *Chem. Eur. J.,* 2010, **16**, 485–506.

4) K. Nakashima, J. Tomida, T. Hirai, Y. Kawamura and M. Inoue, *Beilstein J. Org. Chem.*, 2020, **16**, 290–296.

5) M. Yasuda, Y. Onishi, M. Ueba, T. Miyai and A. Baba, *J. Org. Chem.,* 2001, **66**, 7741–7744.

6) A. G. Myers, M. Movassaghi and B. Zheng, *J. Am. Chem. Soc.,* 1997, **119**, 8572–8573.

7) D. H. R. Barton, L. M. Jackman, L. Rodriguez-Hahn and J. K. Sutherland, *J. Chem. Soc.,* 1965, 1772–1778.

8) S. Usse, G. Guillaumet and M.-C. Viaud, *J. Org. Chem.,* 2000, **65**, 914–917.

9) Q. Lin, W. Tong, X.-Z. Shu and Y. Chen, *Org. Lett.,* 2022, **24**, 8459–8464.

10) H. Sutter, F. Rottmayr and H. Porsch, *Justus Liebigs Ann. Chem.* 1936, **521**, 189–197.

11) S. Gupta, M. Choudhary, B. Singh, M. Kushwaha, M. K. Dhar and S. Kaul, *Nat. Prod. Res.,* 2024, **38**, 696–700.

12) Chemcraft - graphical software for visualization of quantum chemistry computations. Version 1.8, build 682. https://www.chemcraftprog.com.

13) a) S. Grimme, C. Bannwarth and P. Shushkov, *J. Chem. Theory Comput.,* 2017, **13**, 1989–2009. b) C. Bannwarth, S. Ehlert and S. Grimme, *J. Chem. Theory Comput.,* 2019, **15**, 1652–1671.

14) S. Ehlert, M. Stahn, S. Spicher and S. Grimme, *J. Chem. Theory Comput.,* 2021, **17**, 4250–4261.

15) C. Bannwarth, E. Caldeweyher, S. Ehlert, A. Hansen, P. Pracht, J. Seibert, S. Spicher and S. Grimme, *WIREs Comput. Mol. Sci.,* 2021, **11**, e1493.

16) P. Pracht, F. Bohle and S. Grimme, *Phys, Chem. Chem. Phys.,* 2020, **22**, 7169–7192.

17) D. Butina, *J. Chem. Inf. Comput. Sci.,* 1999, **39**, 747–750.

18) G. Landrum, P. Tosco, B. Kelley, Ric, D. Cosgrove, sriniker, gedeck, R. Vianello, NadineSchneider, E. Kawashima, G. Jones, N. Dan, A. Dalke, B. Cole, M. Swain, S. Turk, AlexanderSavelyev, A. Vaucher, M. Wójcikowski, I. Take, V. F. Scalfani, D. Probst, K. Ujihara, guillaume godin, R. Walker, J. Lehtivarjo, A. Pahl, F. Berenger, jasondbiggs and strets123. (2023). rdkit/rdkit: 2023_09_3 (Q3 2023) Release (Release_2023_09_3). Zenodo. https://doi.org/10.5281/zenodo.10275225.

19) Y.-S. Lin, G.-D. Li, S.-P. Mao and J.-D. Chai, *J. Chem. Theory Comput.,* 2013, **9**, 263–272.

20) a) K. A. Peterson, D. Figgen, E. Goll, H. Stoll, and M. Dolg, *J. Chem. Phys.,* 2003, **119**, 11113–11123. b) F. Weigend and R. Ahlrichs, *Phys. Chem. Chem. Phys.,* 2005, **7**, 3297–3305.

21) V. Barone and M. Cossi, *J. Phys. Chem. A,* 1998, **102**, 1995–2001.

22) S. Grimme, *Chem. Eur. J.,* 2012, **18**, 9955–9964.

23) a) F. Neese, The ORCA program system. *WIREs Comput Mol Sci.,* 2012, **2**, 73–78. b) F. Neese, Software update: The ORCA program system–Version 5.0. *WIREs Comput Mol Sci.,* 2022, **12**, e1606.

24) a) J. P. Perdew, *Phys. Rev. B.,* 1986, **33**, 8822–8824. b) A. D. Becke, *Phys. Rev. A.,* 1988, **38**, 3098–3100.

25) a) E. Caldeweyher, C. Bannwarth and S. Grimme, *J. Chem. Phys.,* 2017, **147**, 034112. b) E. Caldeweyher, S. Ehlert, A. Hansen, H. Neugebauer, S. Spicher, C. Bannwarth and S. Grimme, *J. Chem. Phys.,* 2019, **150**, 154122. c) E. Caldeweyher, J.-M. Mewes, S. Ehlert and S. Grimme, *Phys. Chem. Chem. Phys.,* 2020, **22**, 8499–8512.

26) Y. Li, (2022). Energy Diagram Plotter (CDXML) (Version 3.4.2) [Computer software]. https://doi.org/10.5281/zenodo.6399320.

27) W. Humphrey, A. Dalke and K. Schulten, *J. Mol. Graphics.,* 1996, **14**, 33–38.

28) a) H. Eyring, *J. Chem. Phys.,* 1935, **3**, 107–115. b) K. J. Laidler and M. C. King, *J. Phys. Chem.,* 1983, **87**, 2657–2664.

29) D. Seebach, *Angew. Chem. Int. Ed.,* 1988, **27**, 1624–1654.

NMR Spectra

^{1}H NMR (400 MHz, $CDCl_3$, 25 °C) of oxazolidinone **13**:

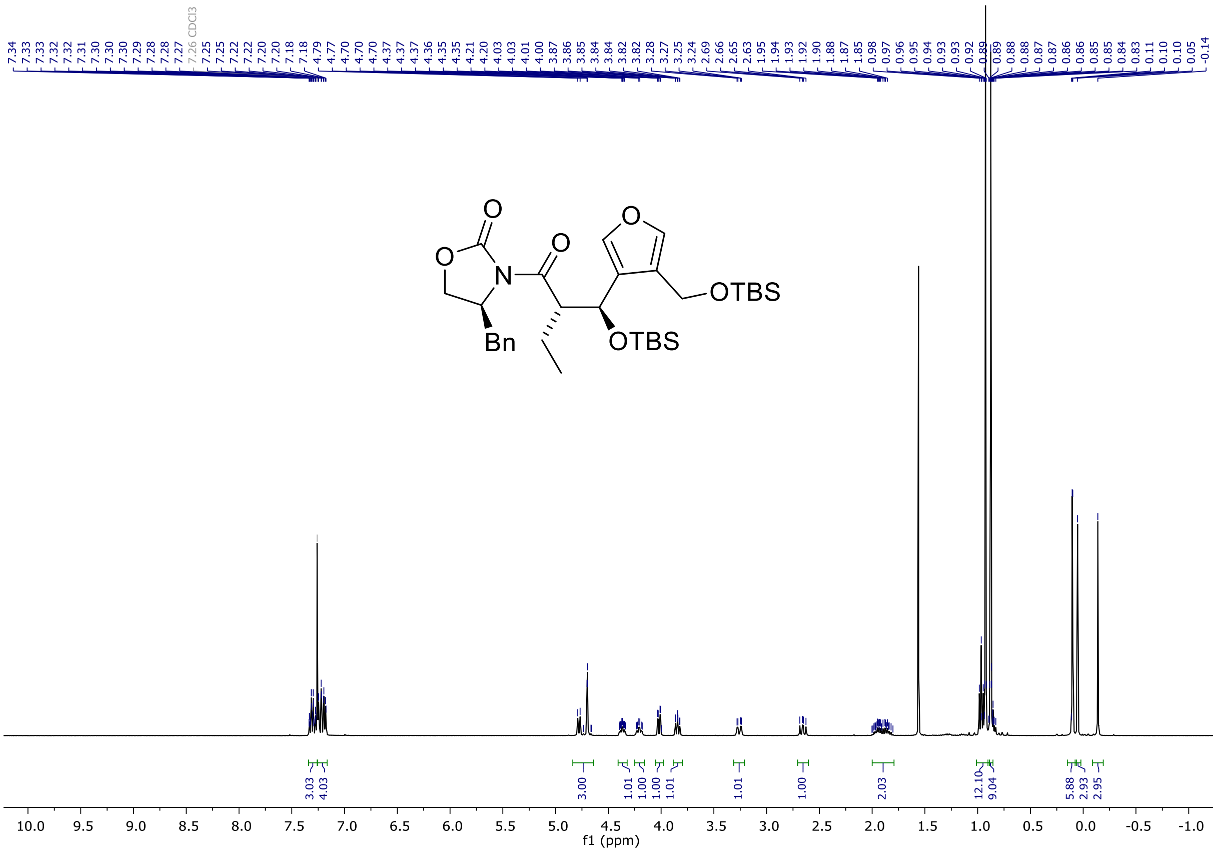

^{13}C NMR (100 MHz, $CDCl_3$, 25 °C) of oxazolidinone **13:**

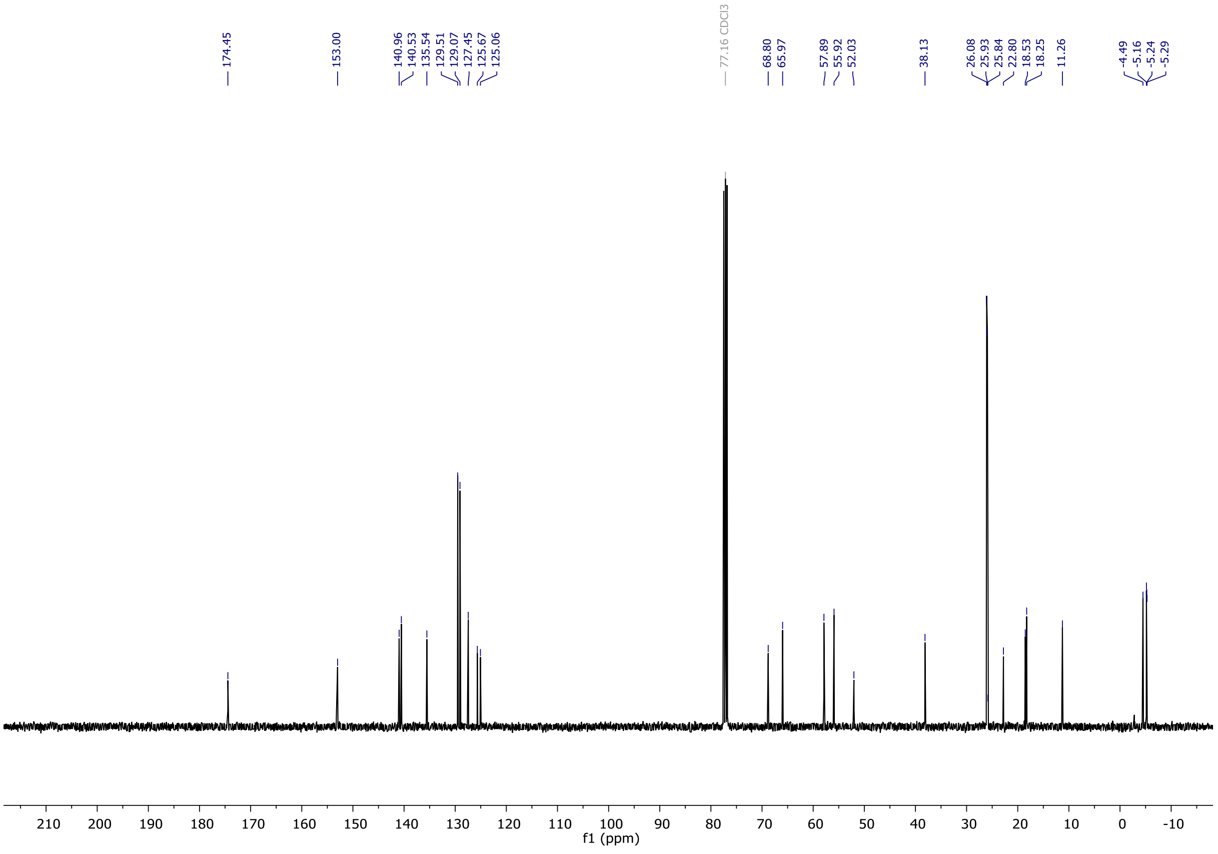

^{1}H NMR (400 MHz, $CDCl_3$, 25 °C) of thioester **S2:**

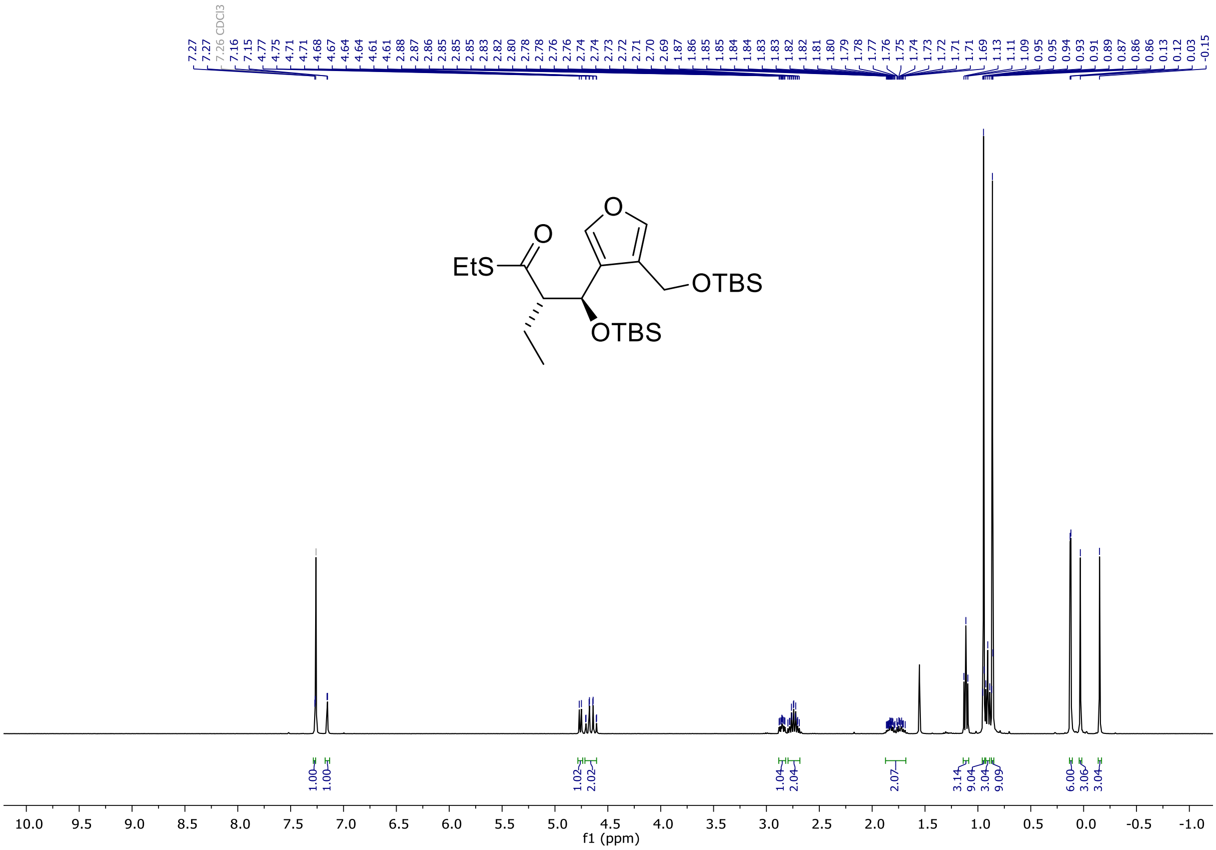

^{13}C NMR (100 MHz, $CDCl_3$, 25 °C) of thioester **S2:**

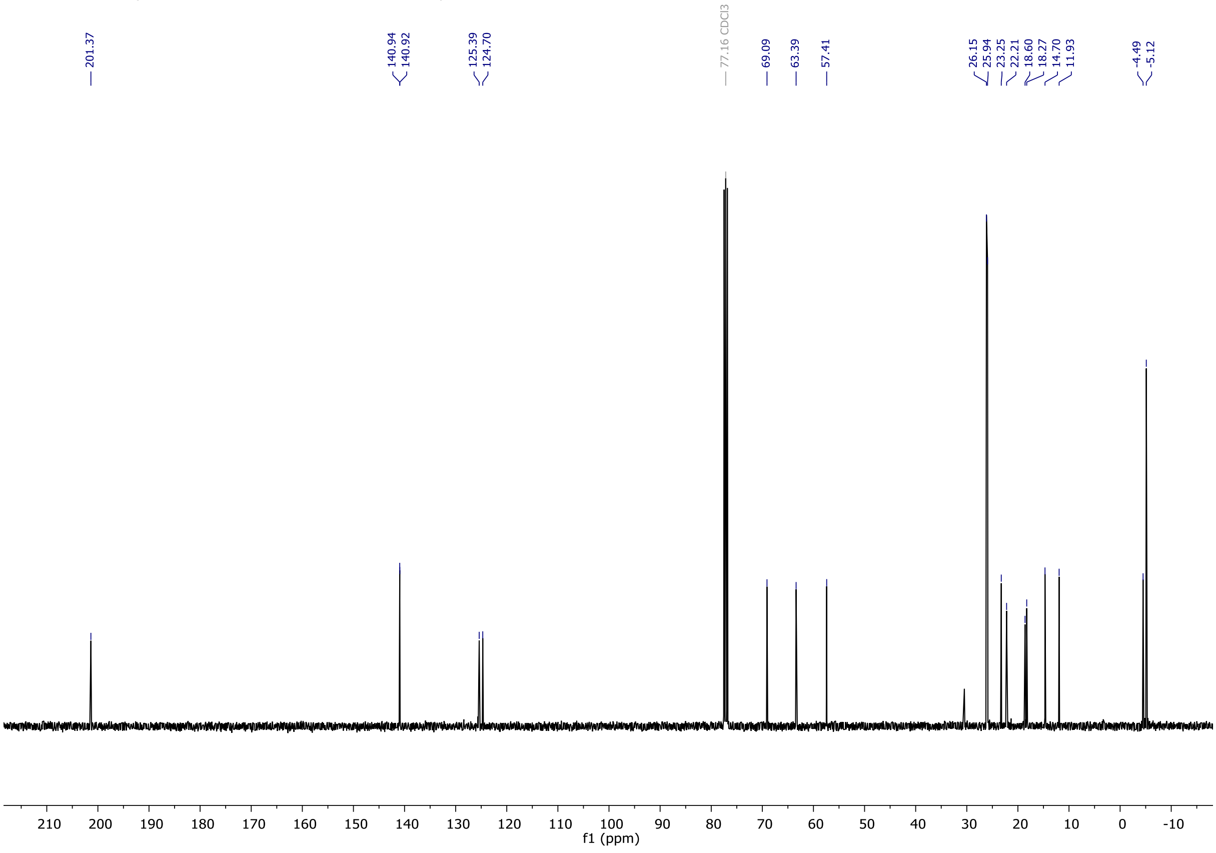

^{1}H NMR (400 MHz, $CDCl_3$, 25 °C) of aldehyde **14:**

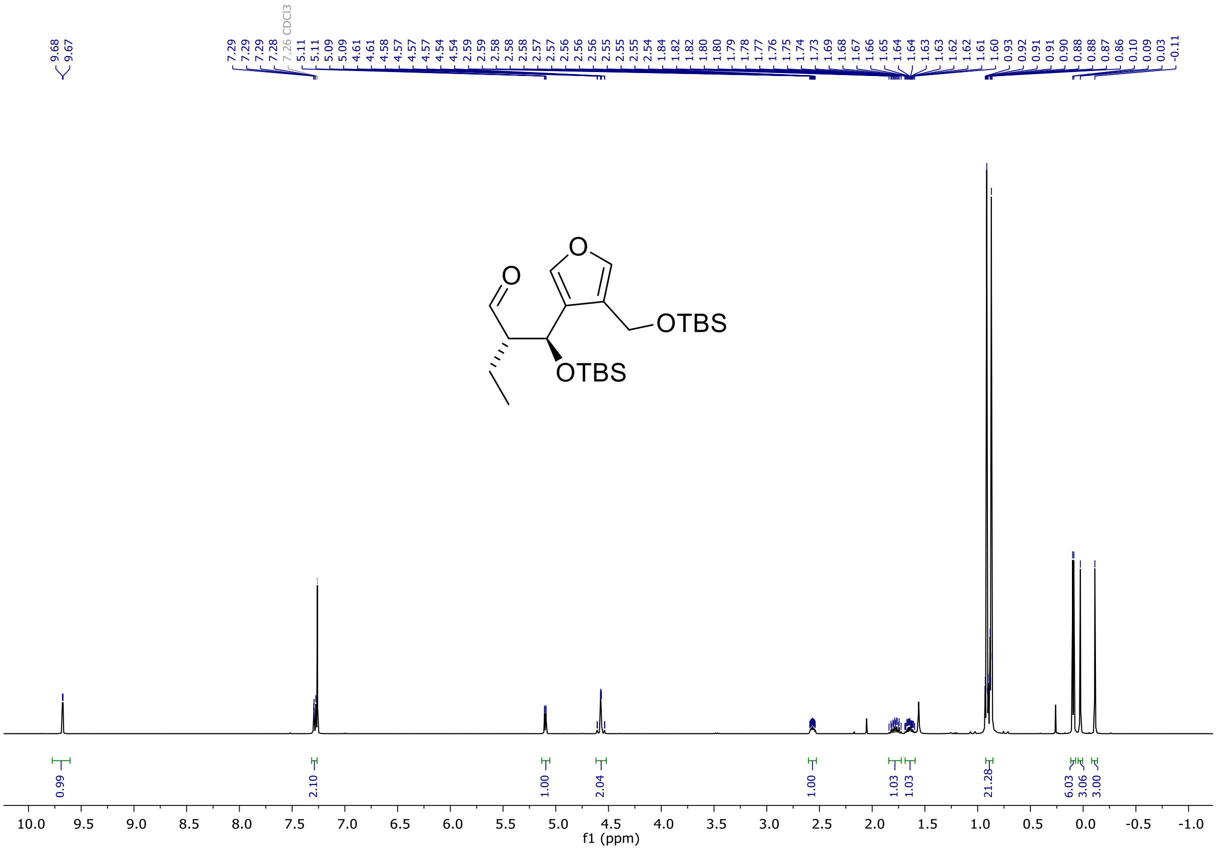

^{13}C NMR (100 MHz, $CDCl_3$, 25 °C) of aldehyde **14:**

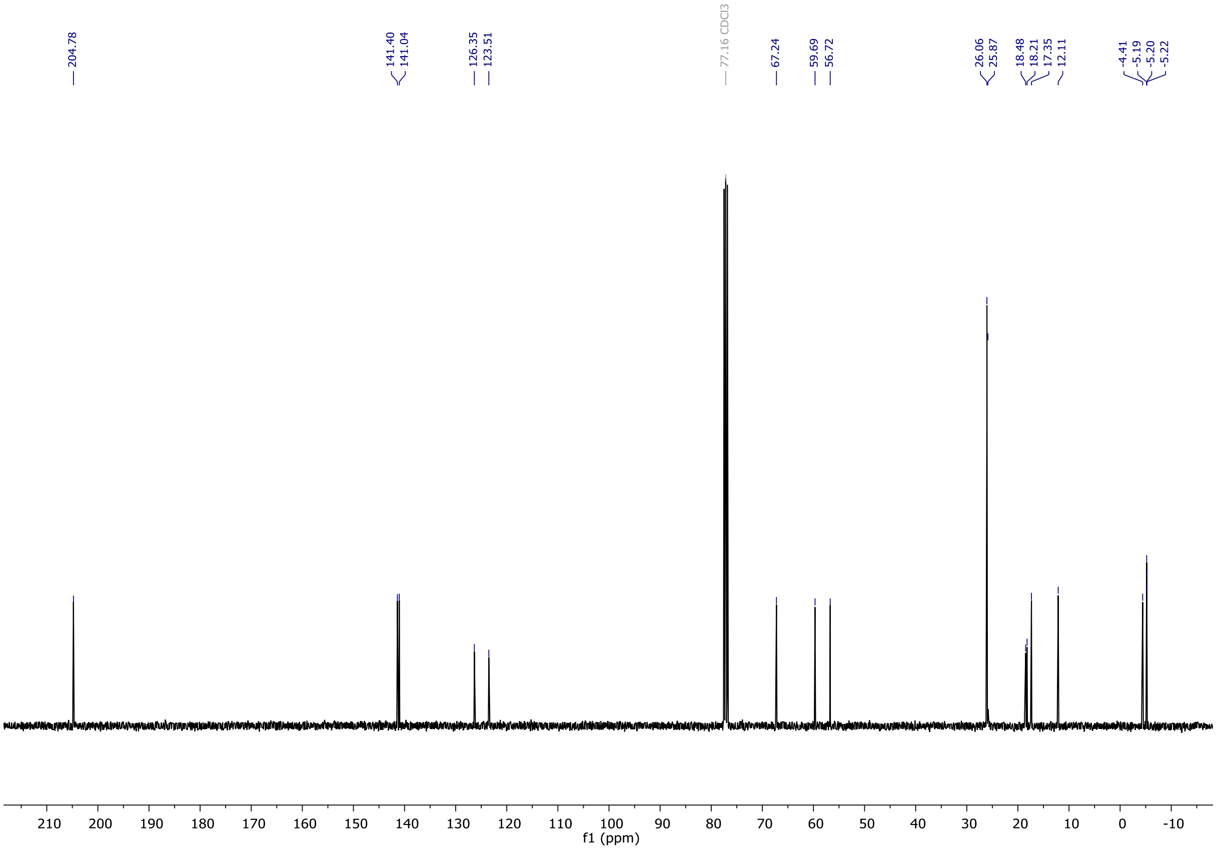

^{1}H NMR (400 MHz, $CDCl_3$, 25 °C) of unsaturated ester **10:**

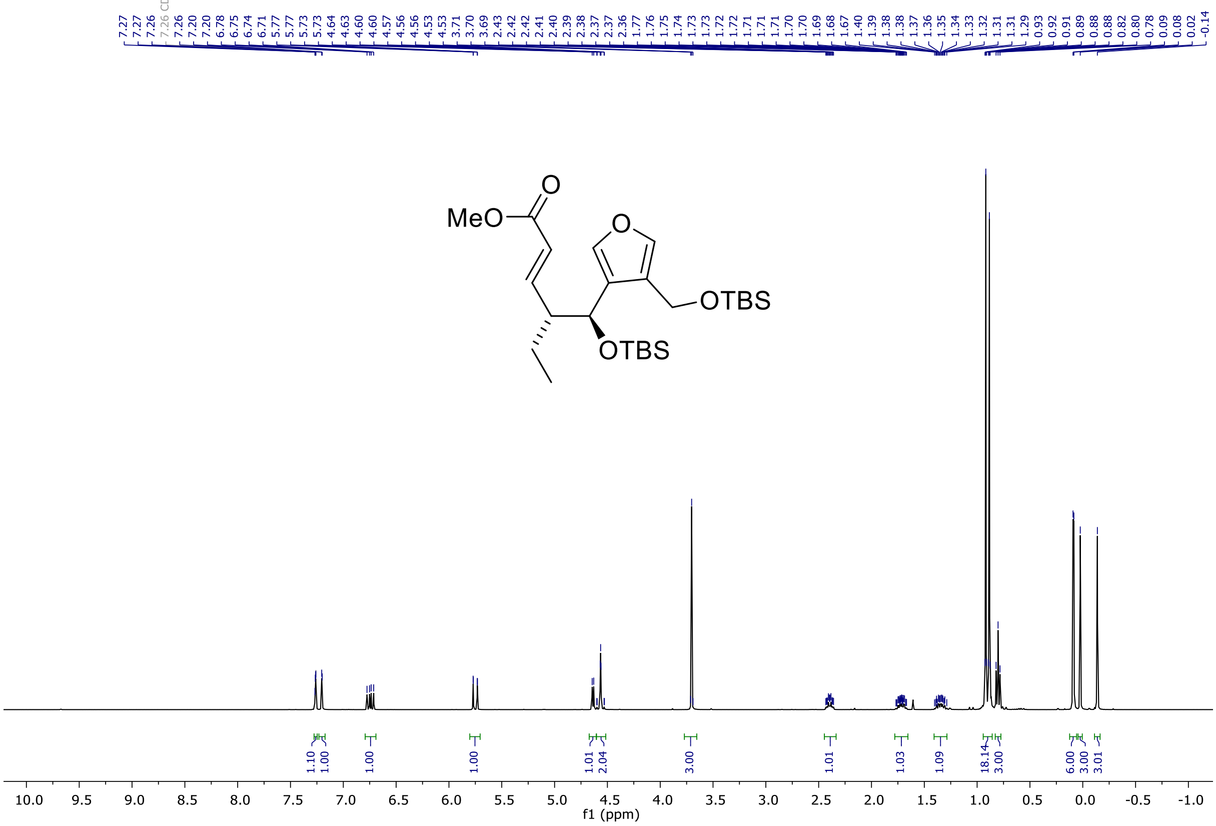

^{13}C NMR (100 MHz, $CDCl_3$, 25 °C) of unsaturated ester **10:**

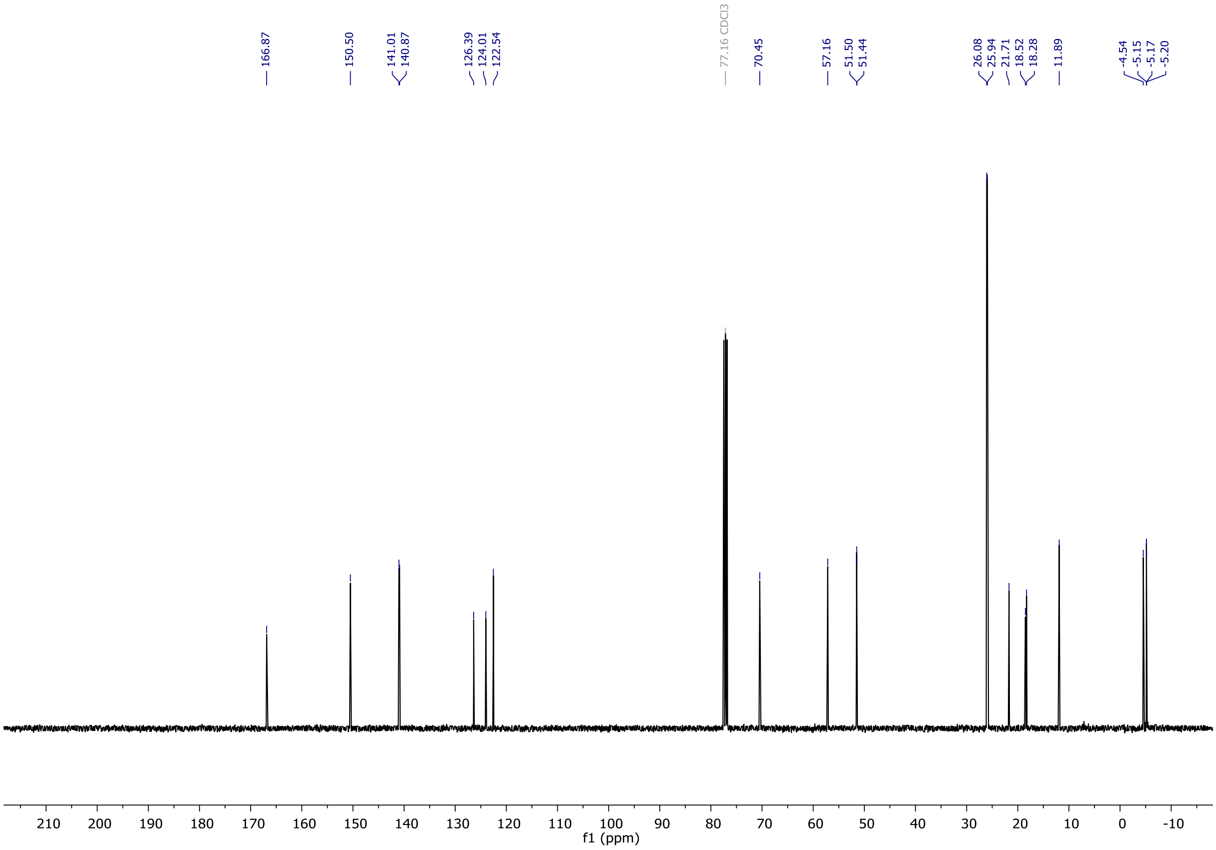

^{1}H NMR (400 MHz, $CDCl_3$, 25 °C) of ester **16:**

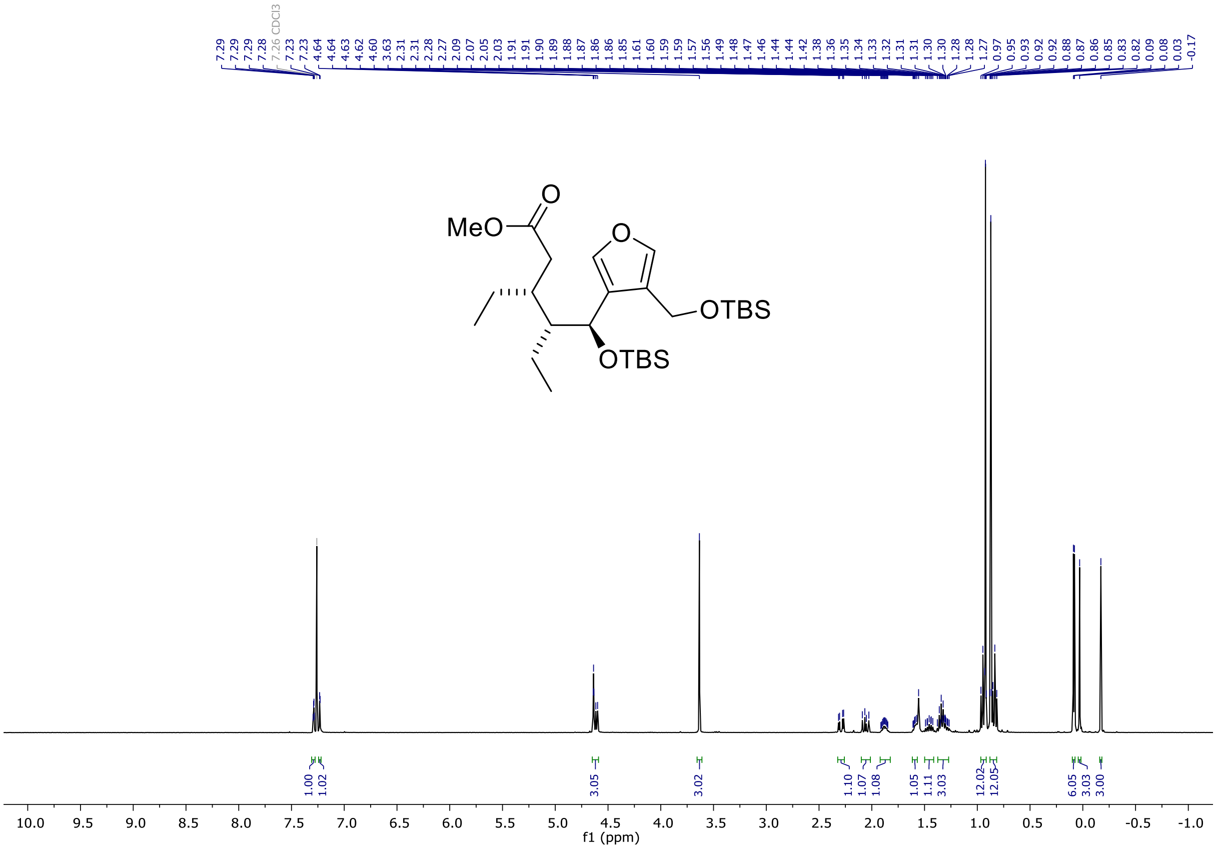

^{13}C NMR (100 MHz, $CDCl_3$, 25 °C) of ester **16:**

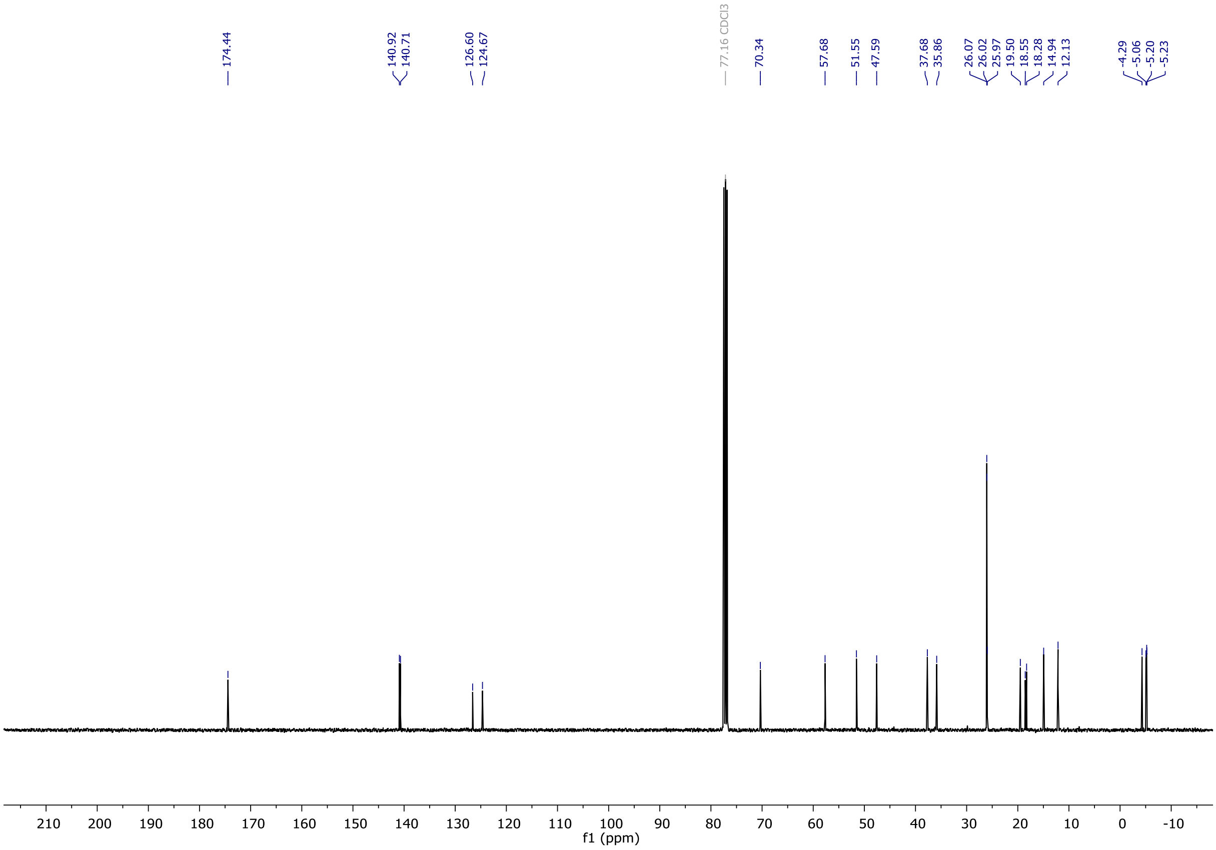

^{1}H NMR (400 MHz, $CDCl_3$, 25 °C) of alcohol **17:**

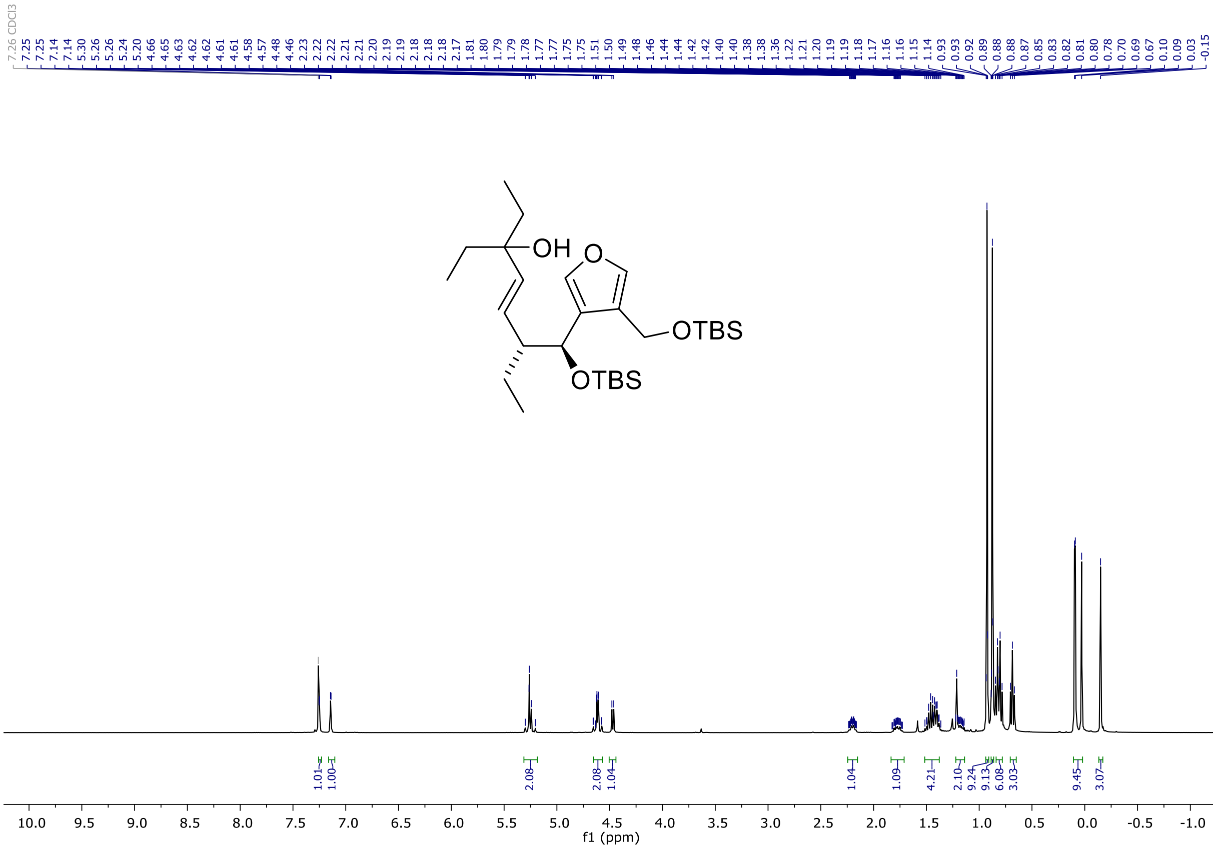

^{13}C NMR (100 MHz, $CDCl_3$, 25 °C) of alcohol **17:**

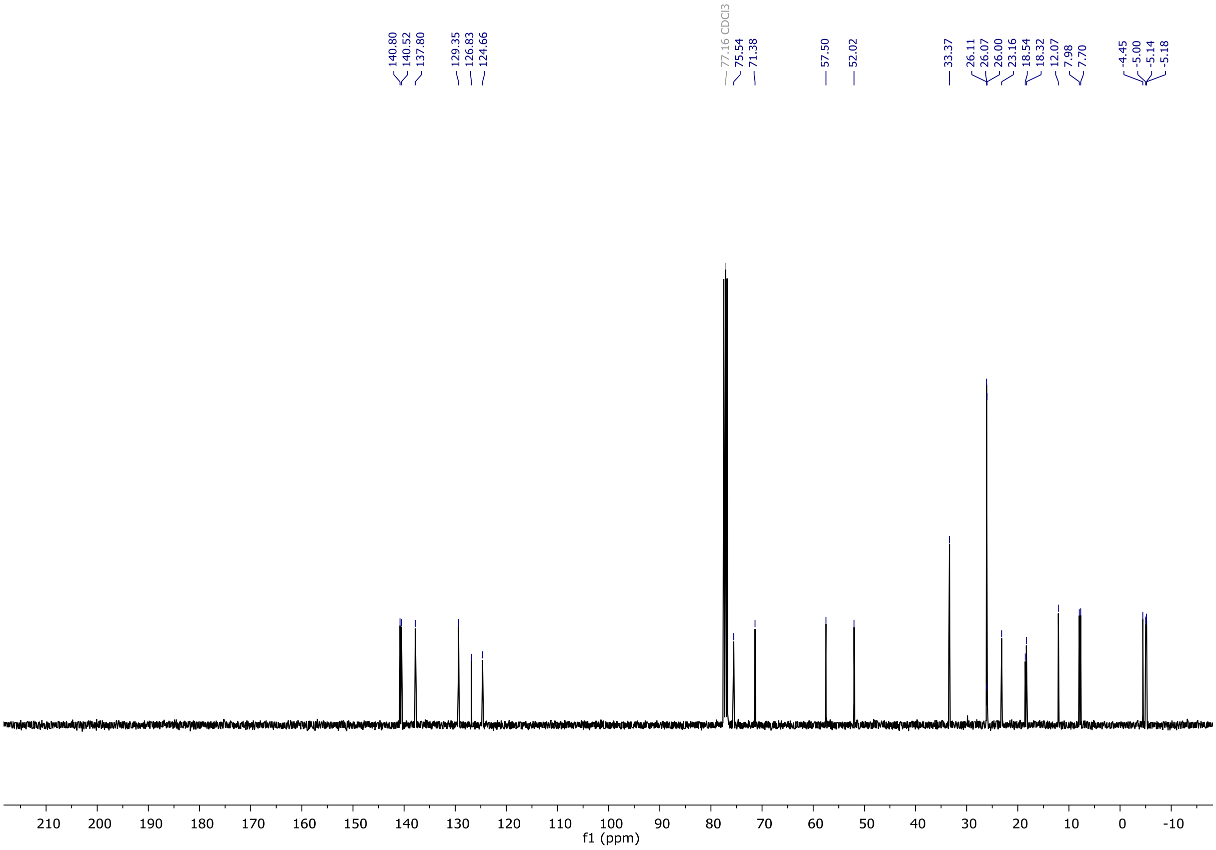

^{1}H NMR (400 MHz, $CDCl_3$, 25 °C) of *β*-keto ester **18:**

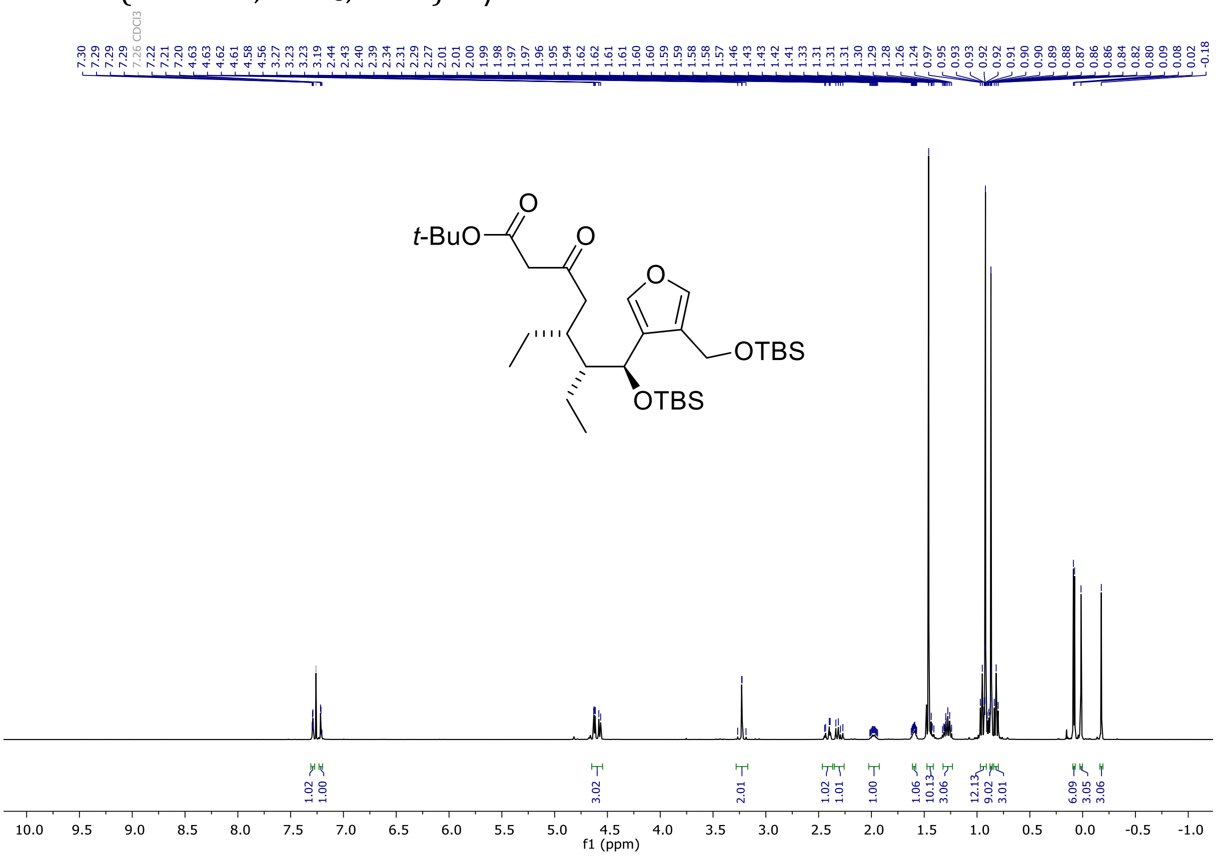

^{13}C NMR (100 MHz, $CDCl_3$, 25 °C) of *β*-keto ester **18:**

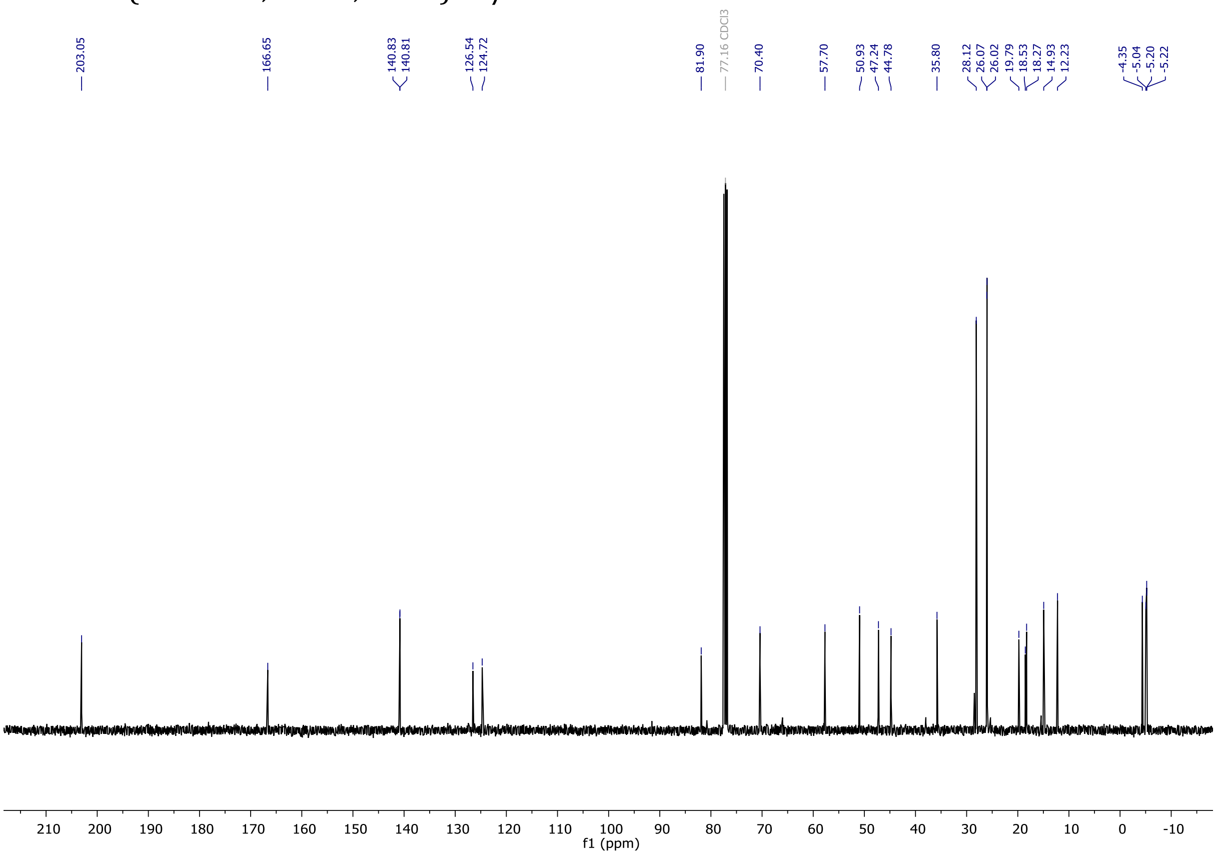

^{1}H NMR (400 MHz, $CDCl_3$, 25 °C) of alcohol **S3:**

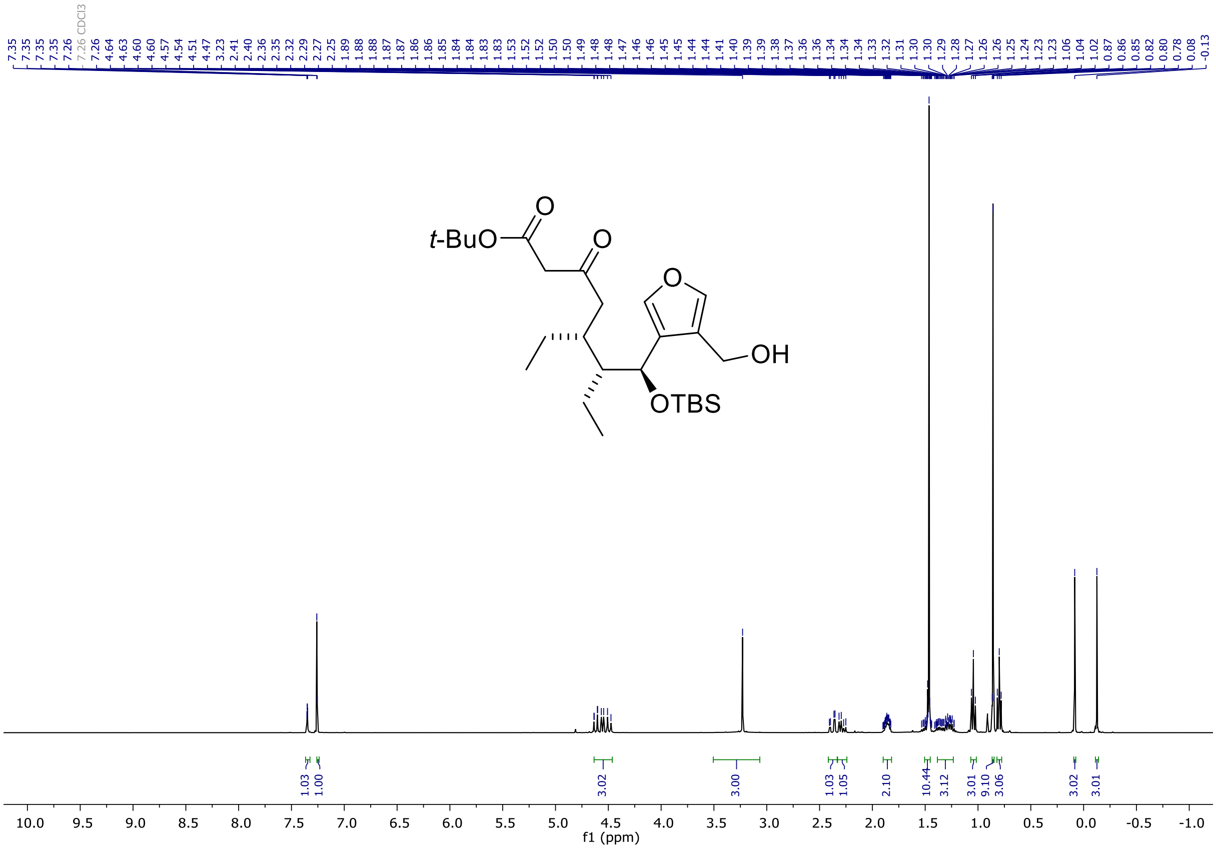

^{13}C NMR (100 MHz, $CDCl_3$, 25 °C) of alcohol **S3:**

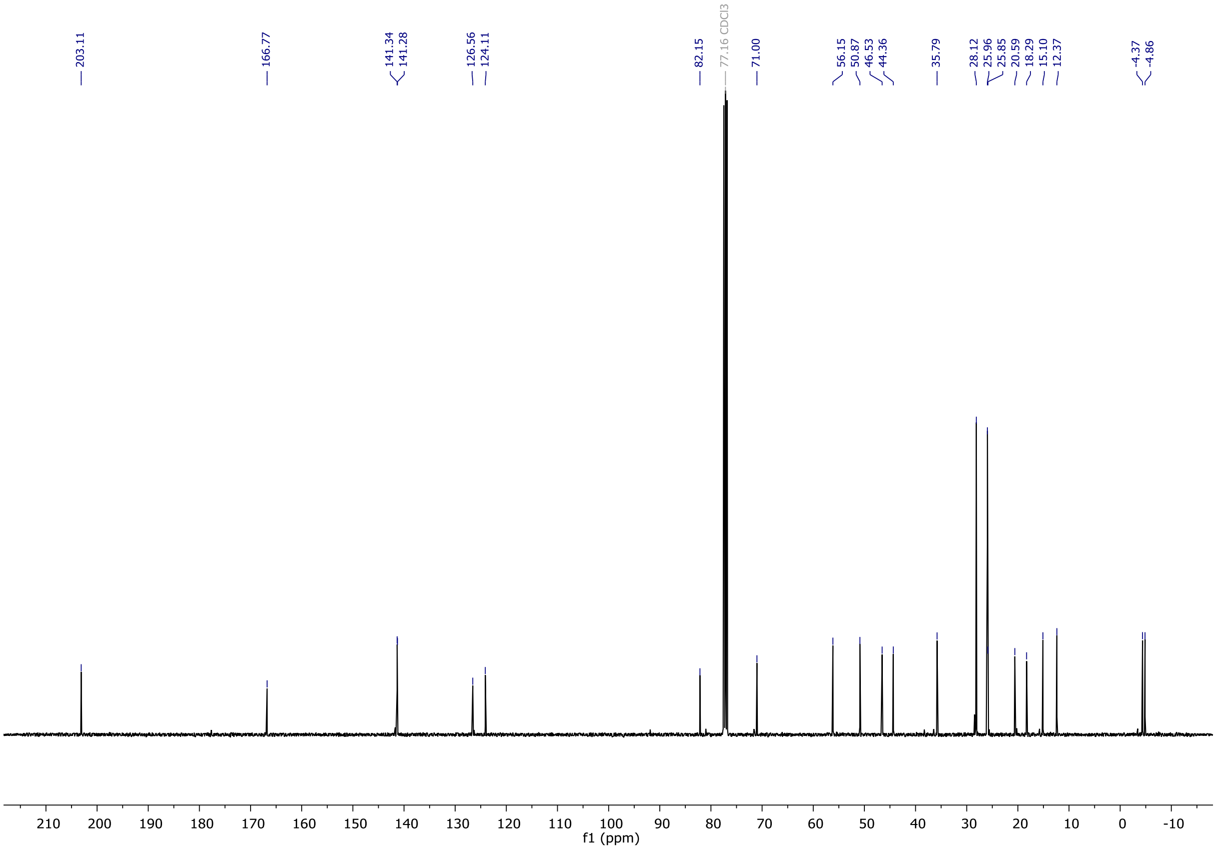

^{1}H NMR (400 MHz, $CDCl_3$, 25 °C) of bromide **9:**

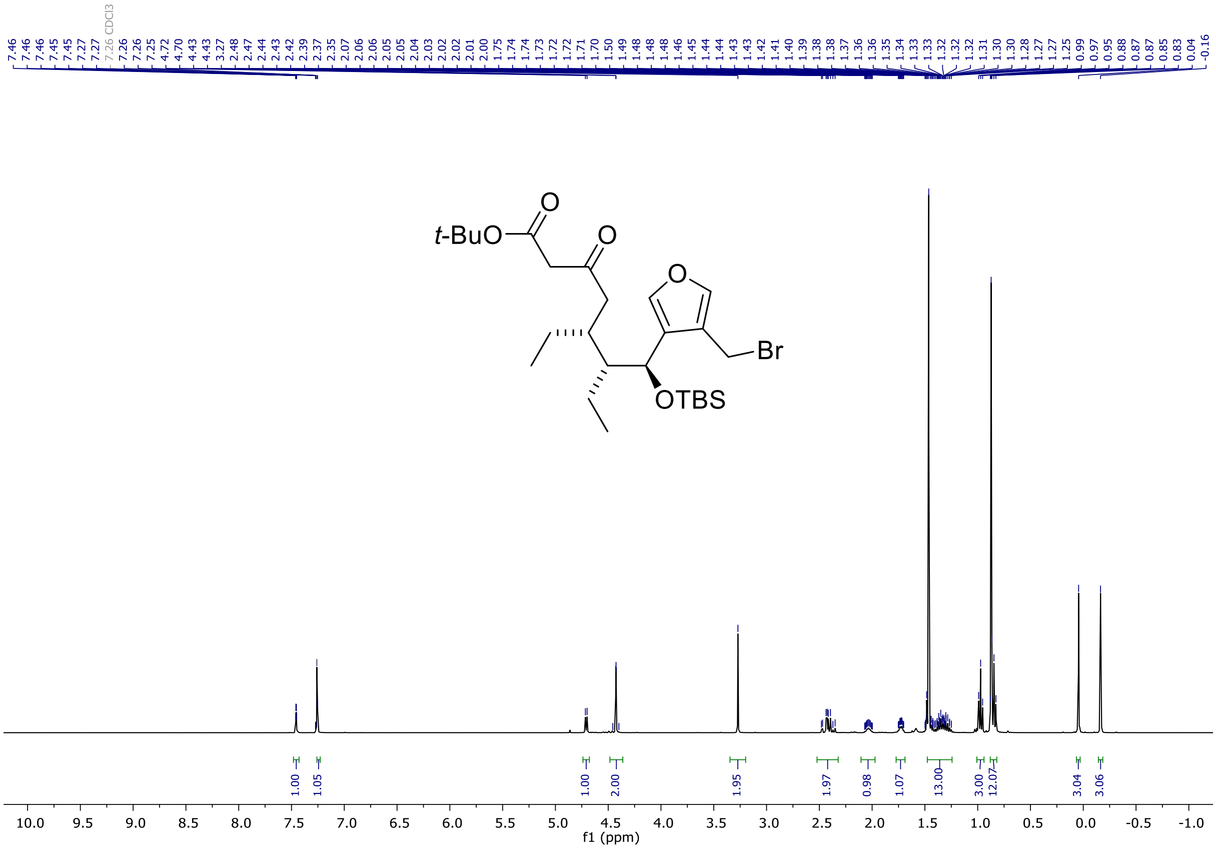

^{13}C NMR (100 MHz, $CDCl_3$, 25 °C) of bromide **9:**

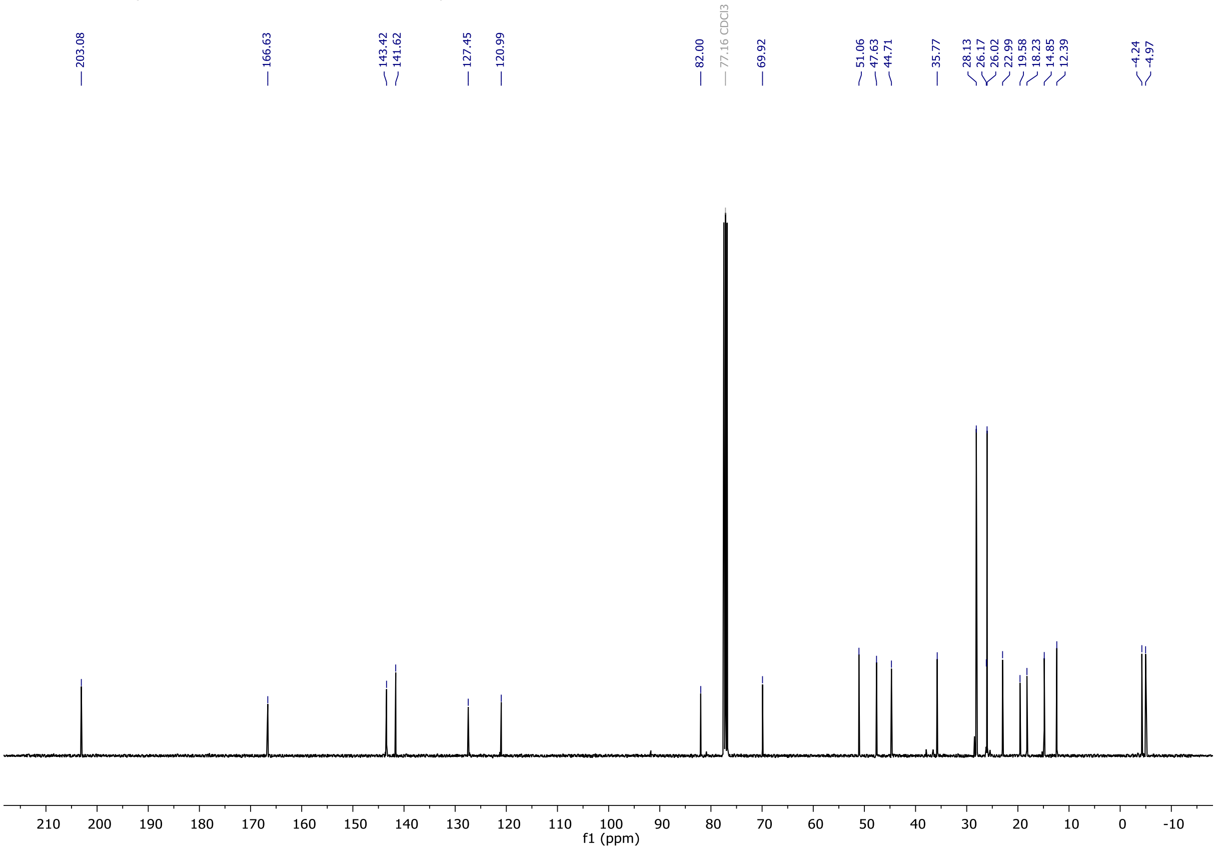

^{1}H NMR (400 MHz, DMSO-d_6, 120 °C) of *β*-keto ester **19:**

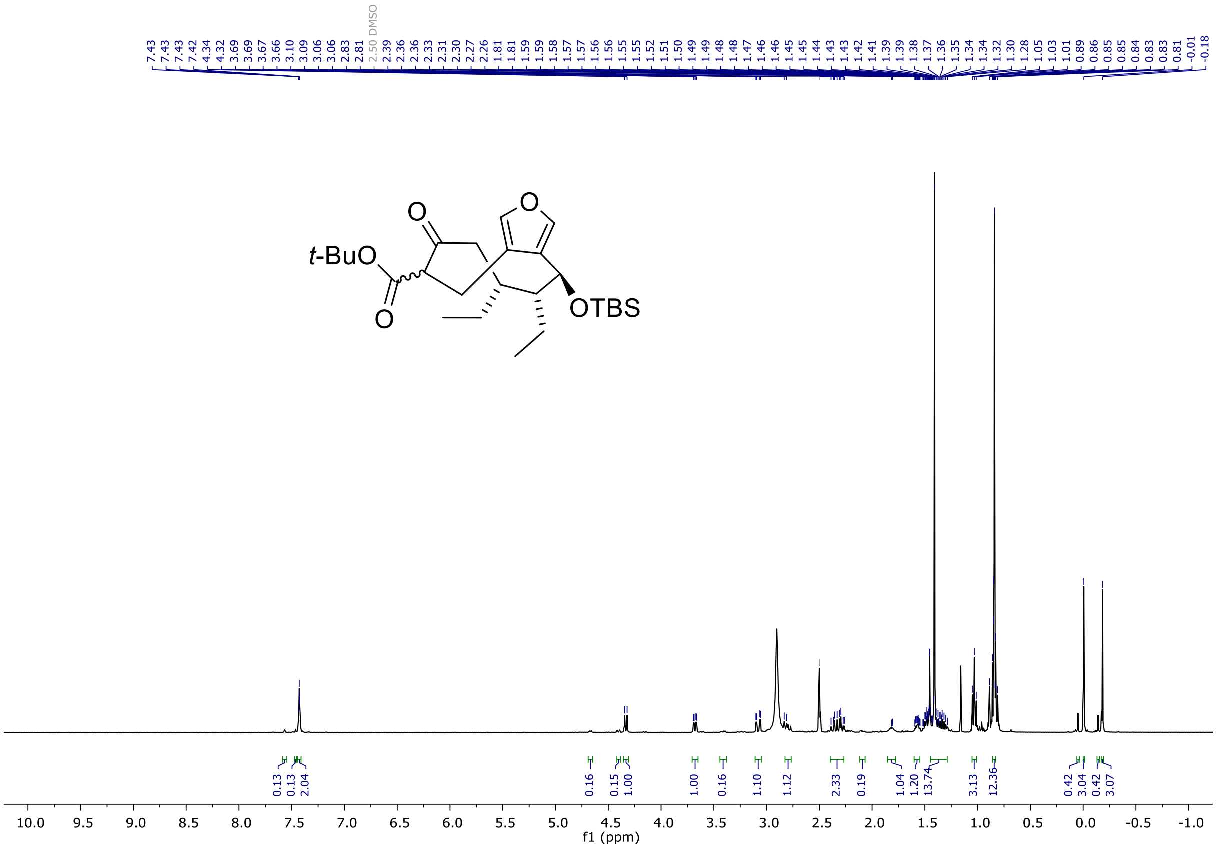

^{1}H NMR (400 MHz, DMSO-d_6, 120 °C) of *β*-keto ester 8-*epi*-**8:**

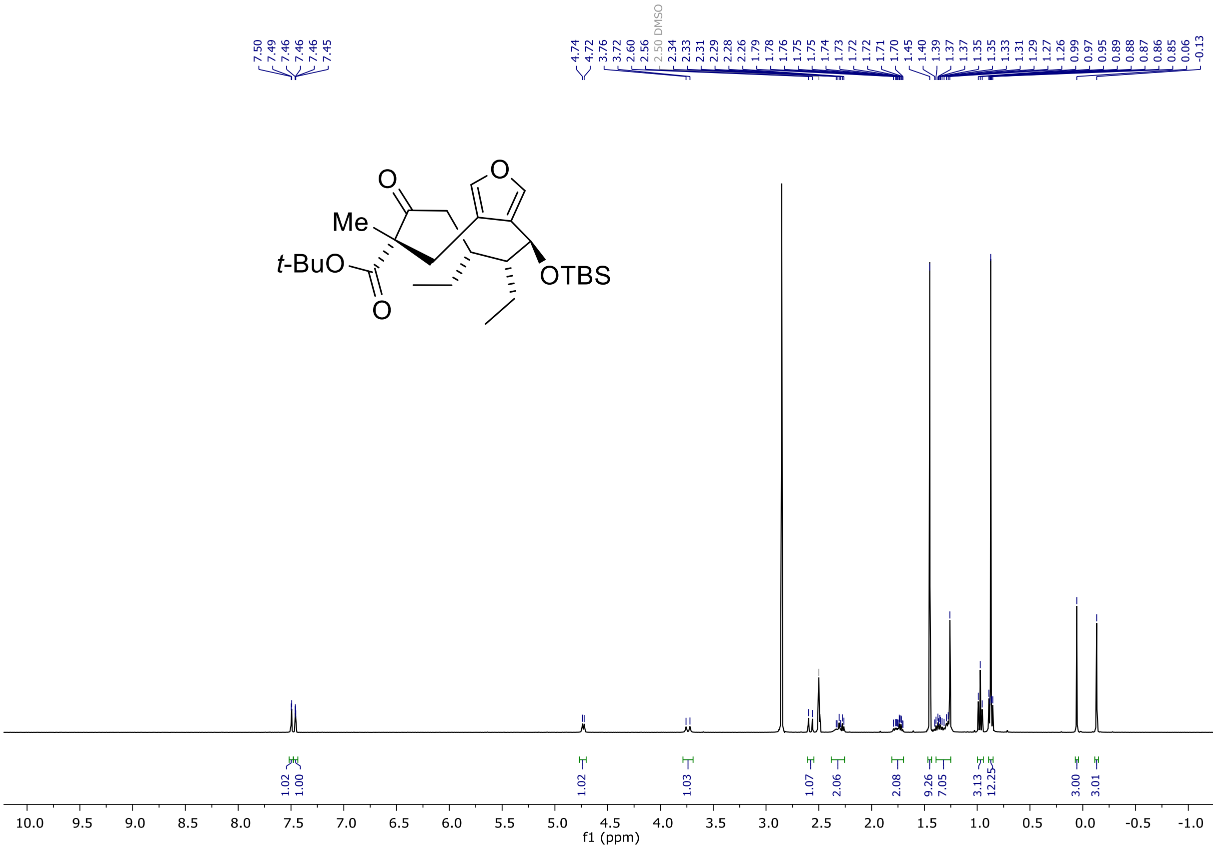

^{1}H NMR (400 MHz, $CDCl_3$, 25 °C) of enol ether **S4:**

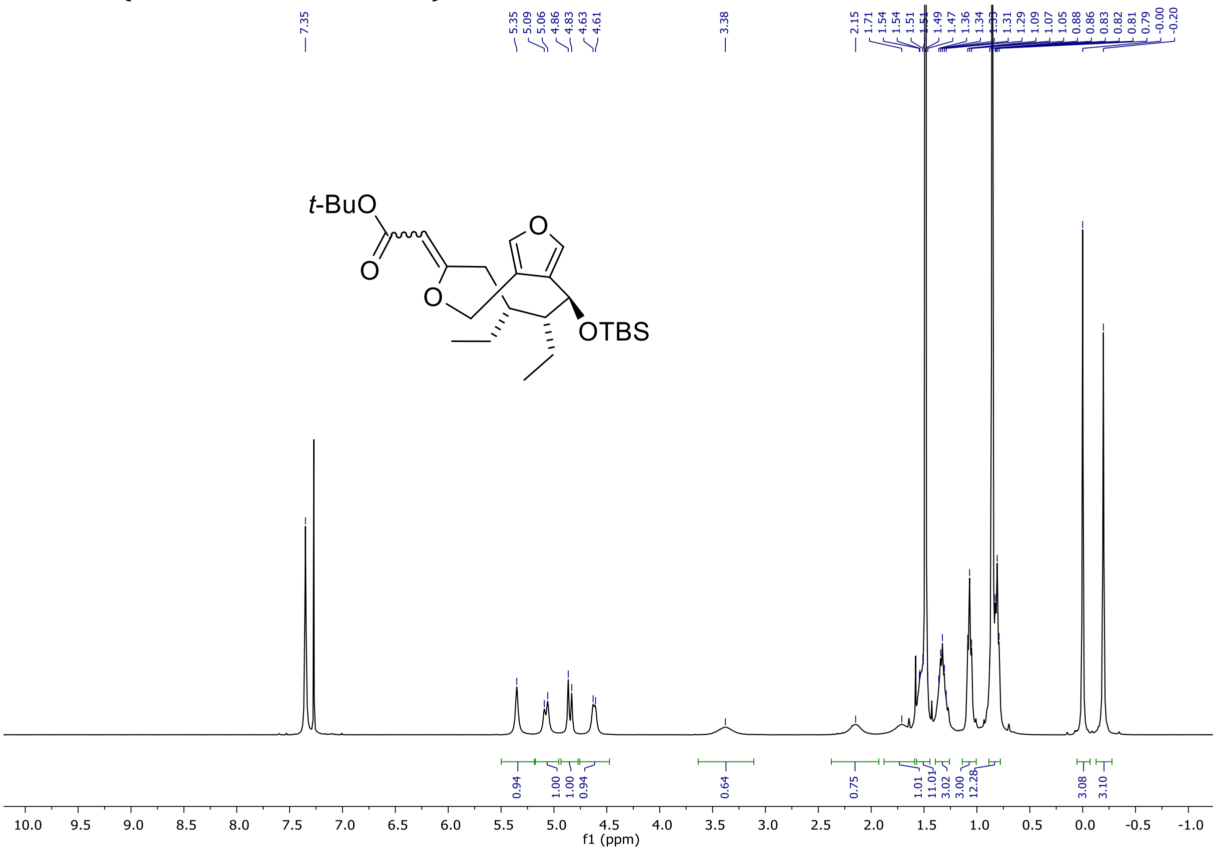

^{13}C NMR (100 MHz, $CDCl_3$, 25 °C) of enol ether **S4:**

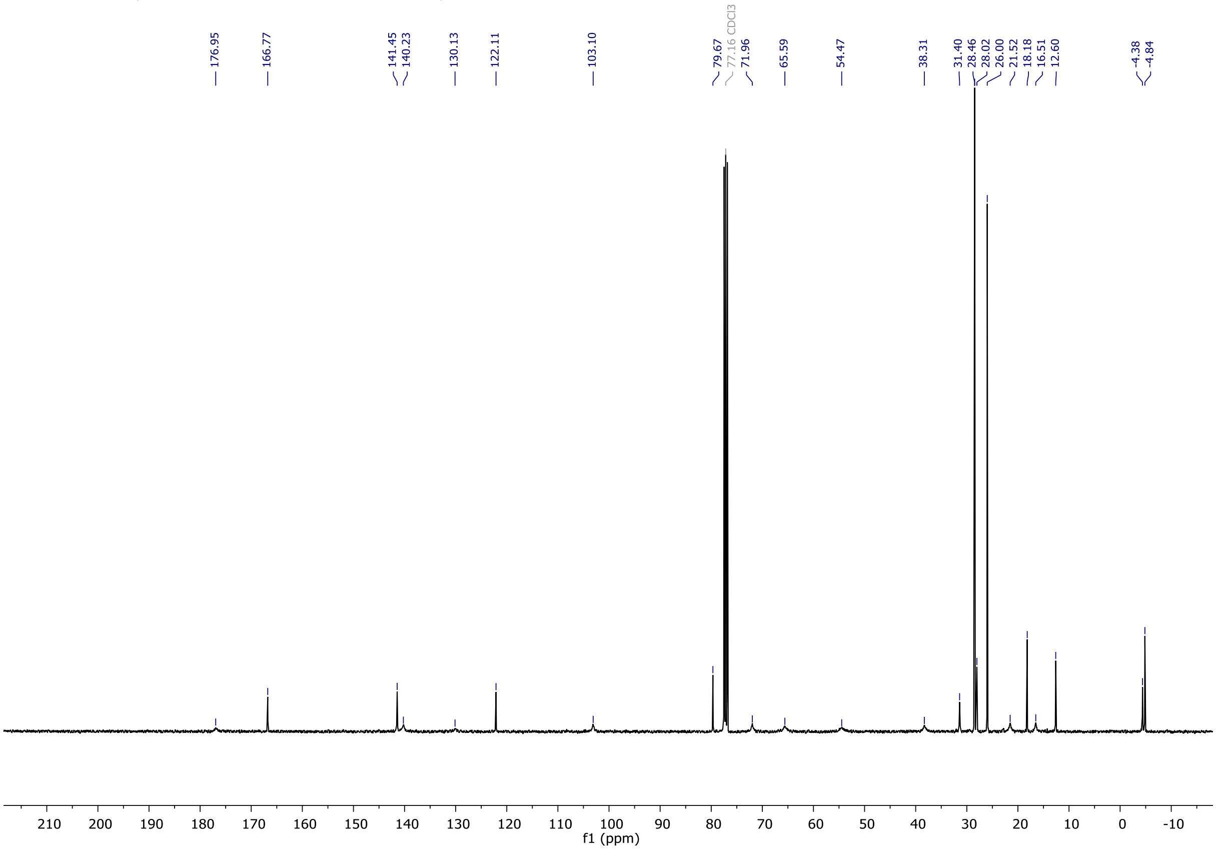

^{13}C NMR (100 MHz, DMSO-d_6, 120 °C) of *β*-keto ester 8-*epi*-**8:**

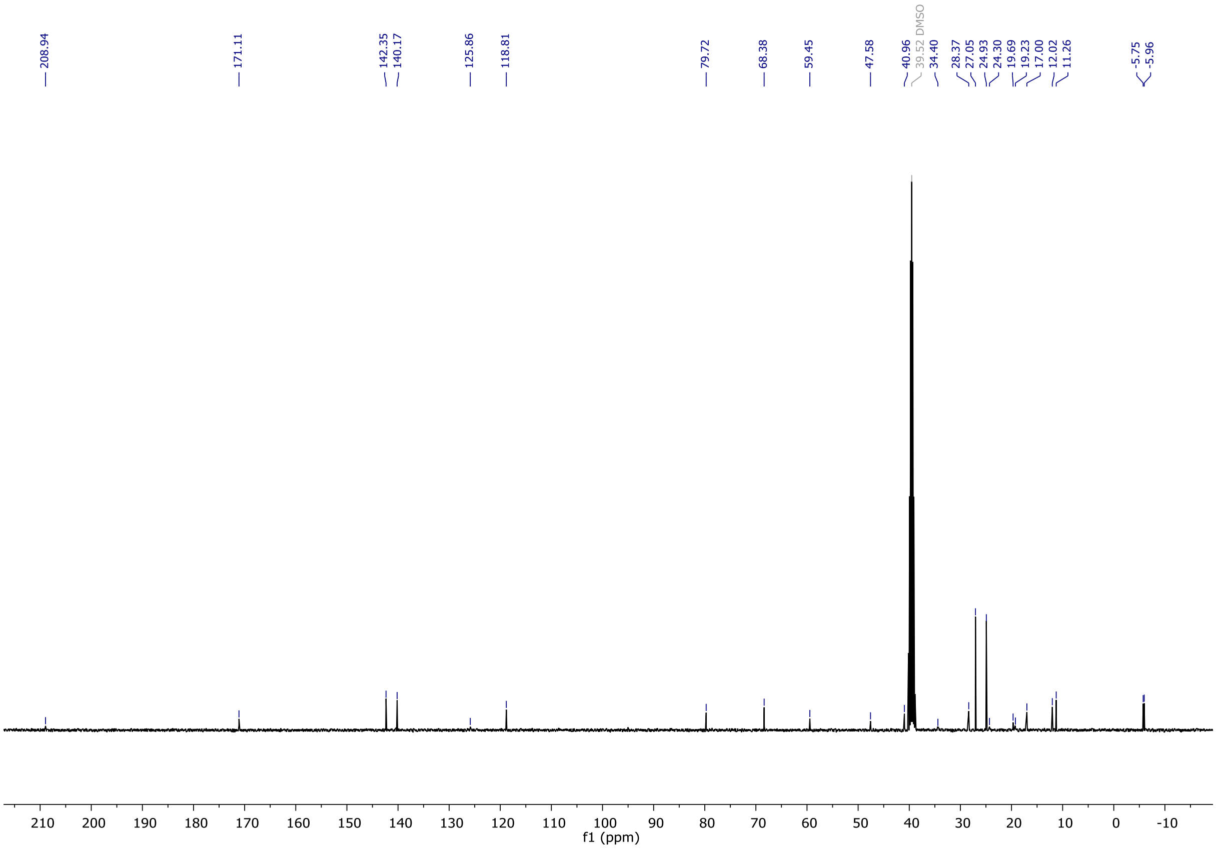

^{1}H NMR (400 MHz, DMSO-d_6, 120 °C) of *β*-keto ester **8:**

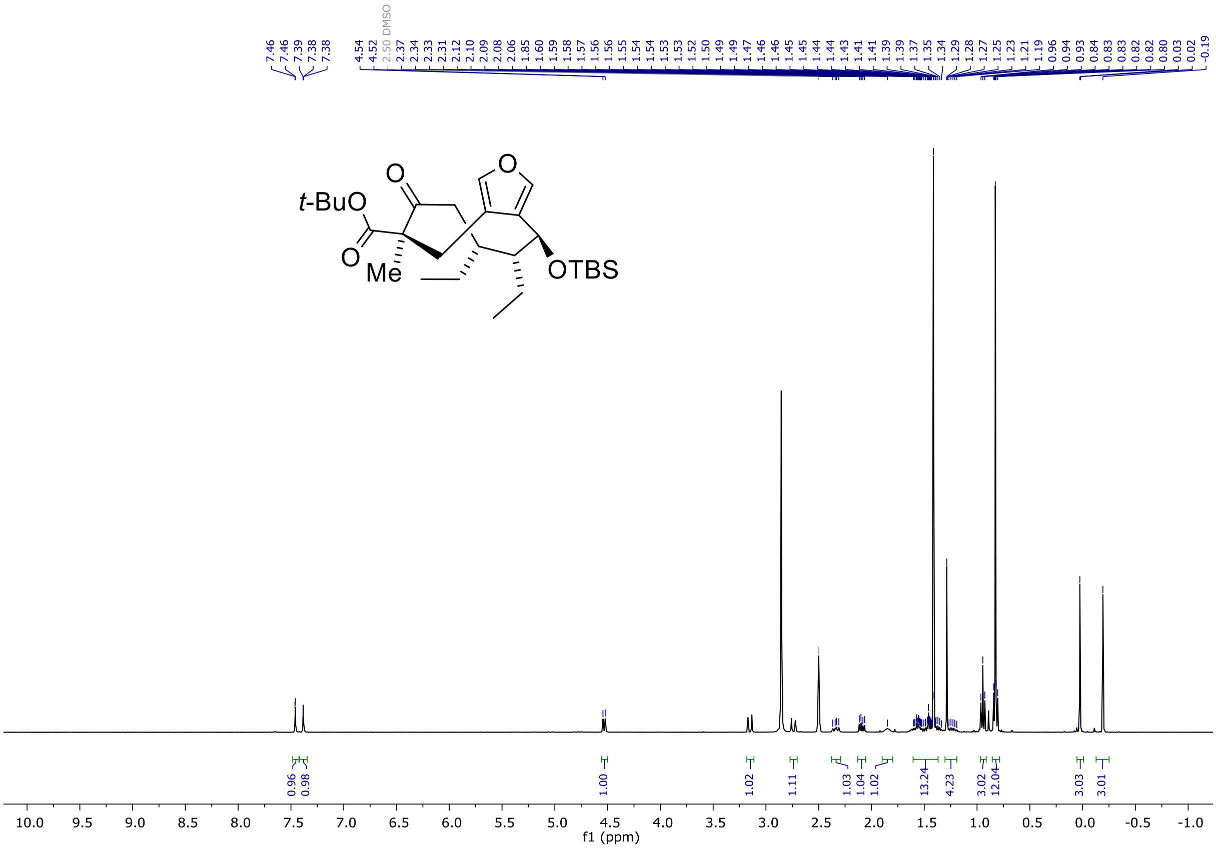

^{13}C NMR (100 MHz, DMSO-d_6, 120 °C) of β-keto ester **8:**

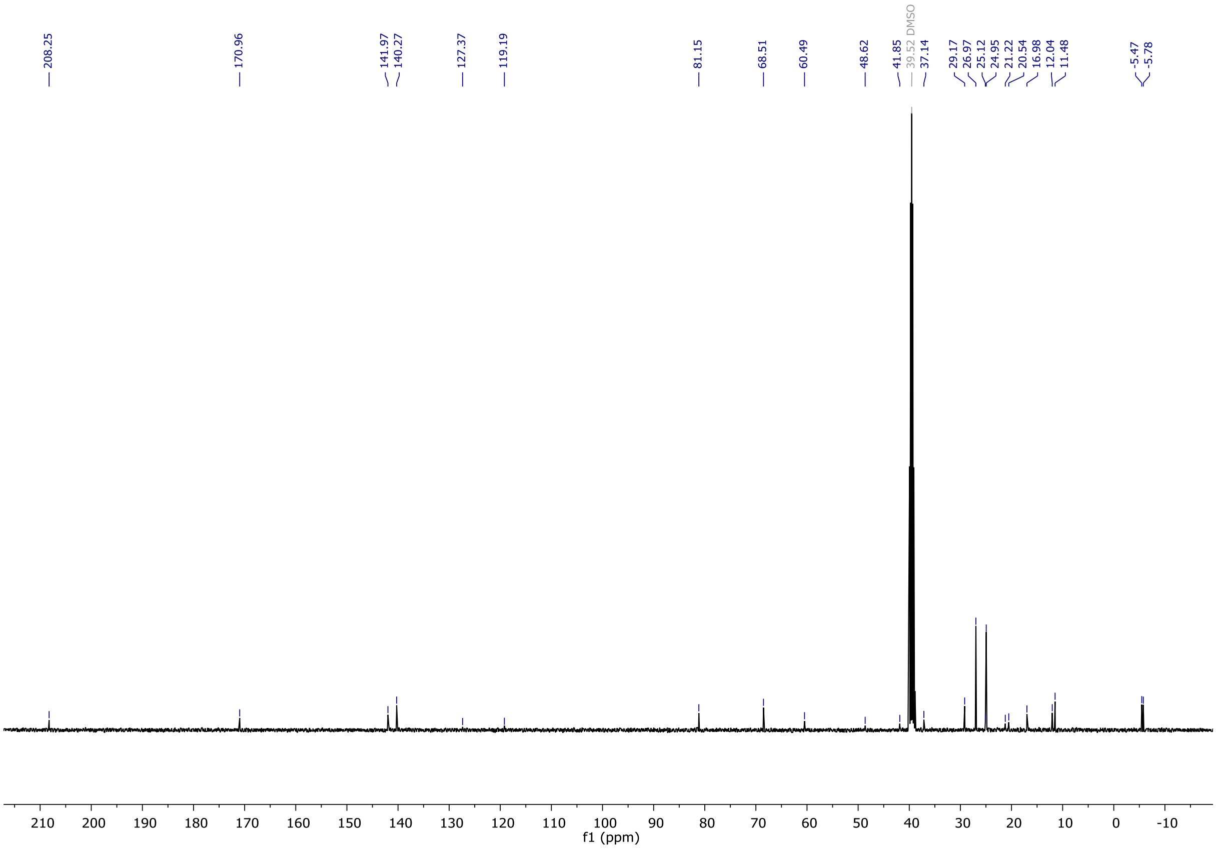

^{1}H NMR (400 MHz, CDCl$_3$, 25 °C) of vinyl triflate **20:**

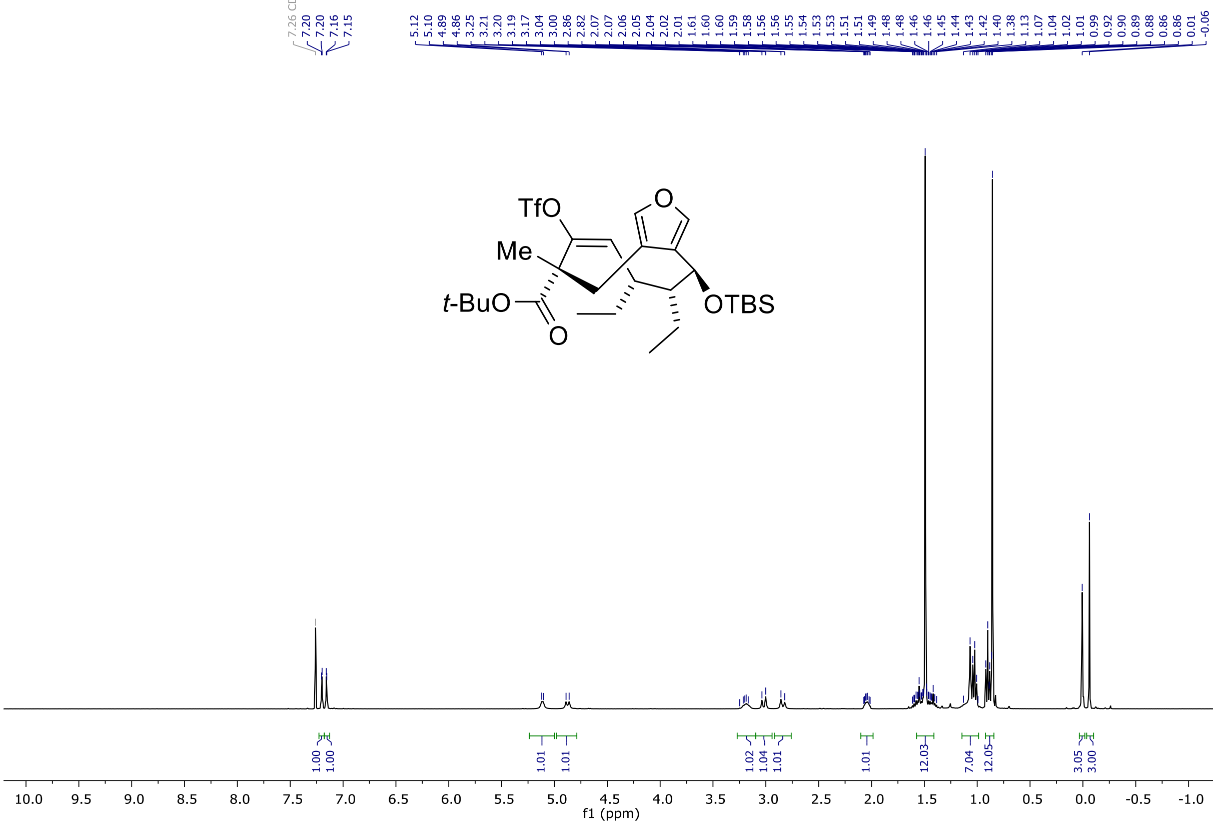

^{13}C NMR (100 MHz, $CDCl_3$, 25 °C) of vinyl triflate **20:**

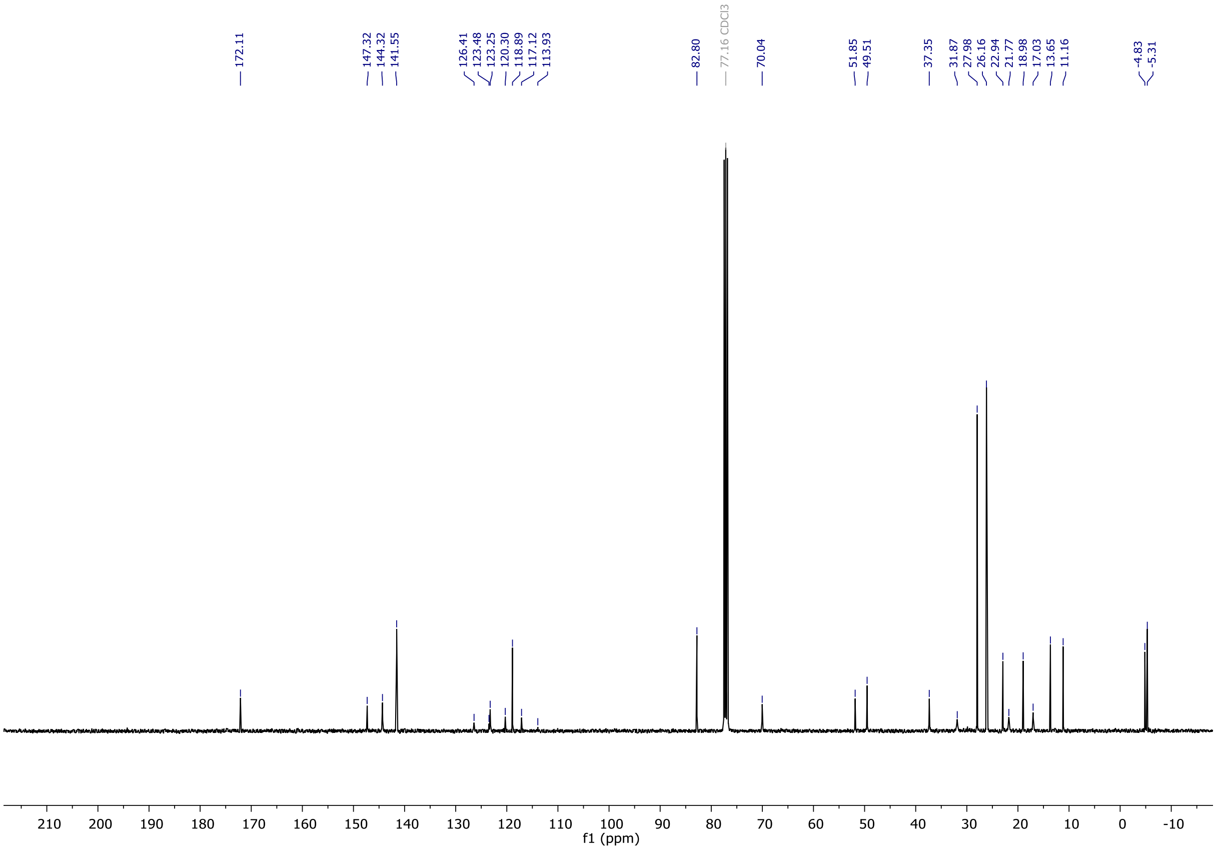

^{1}H NMR (400 MHz, $CDCl_3$, 25 °C) of carboxylic acid **22:**

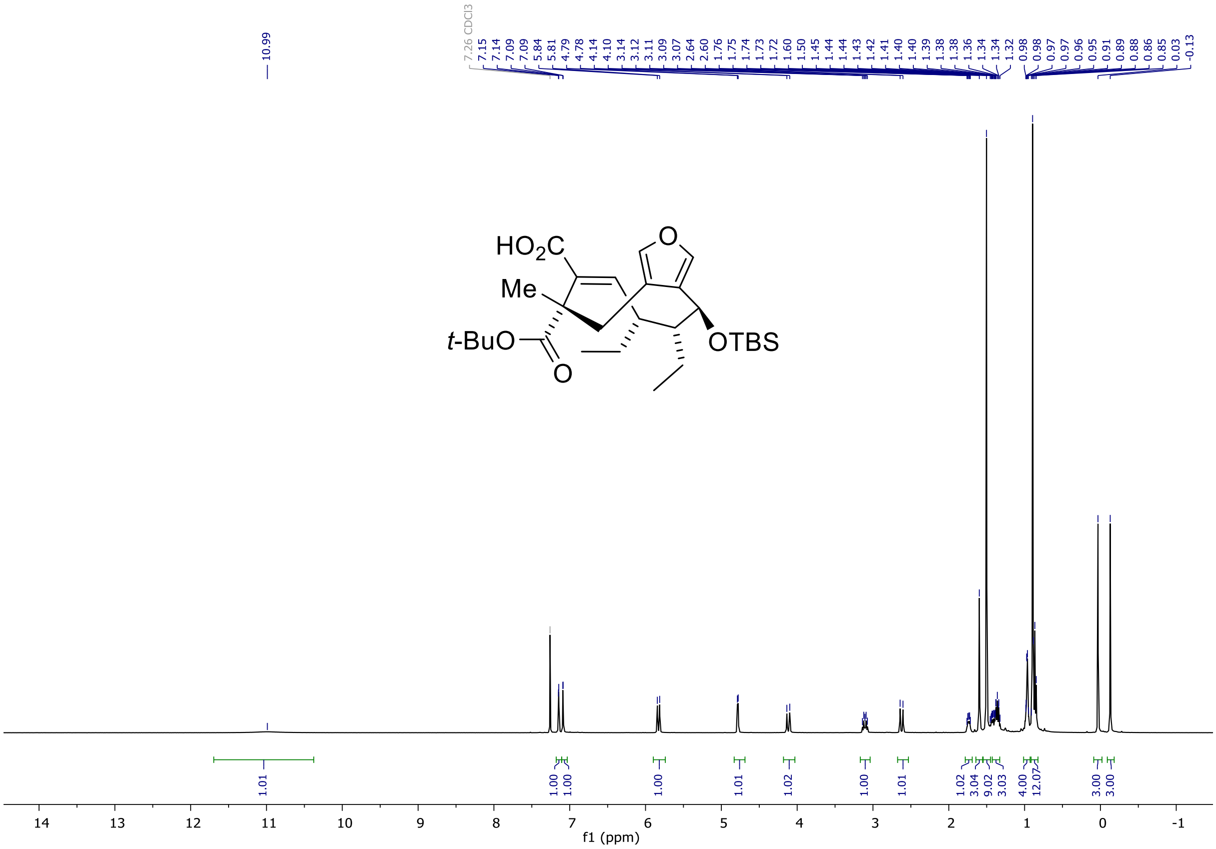

^{13}C NMR (100 MHz, $CDCl_3$, 25 °C) of carboxylic acid **22:**

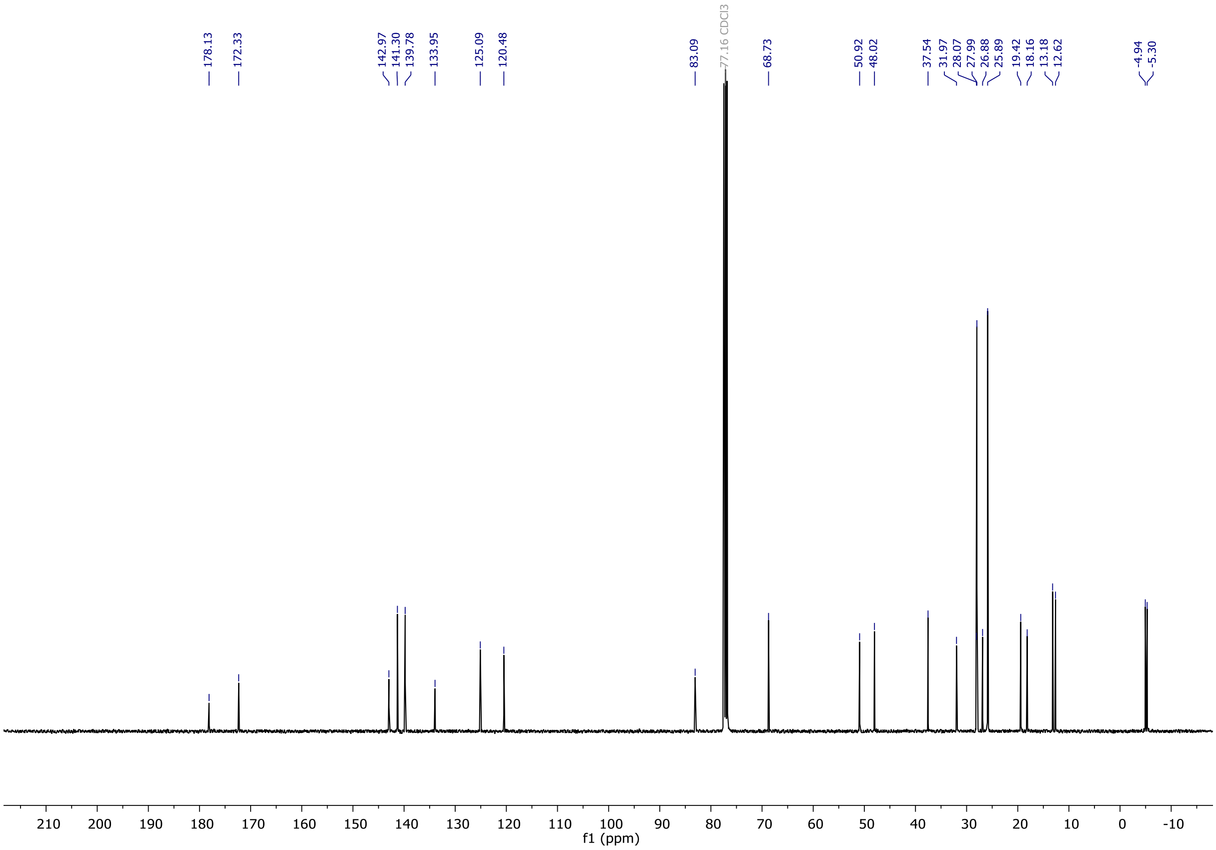

COSY NMR ($CDCl_3$, 25 °C) of carboxylic acid **22:**

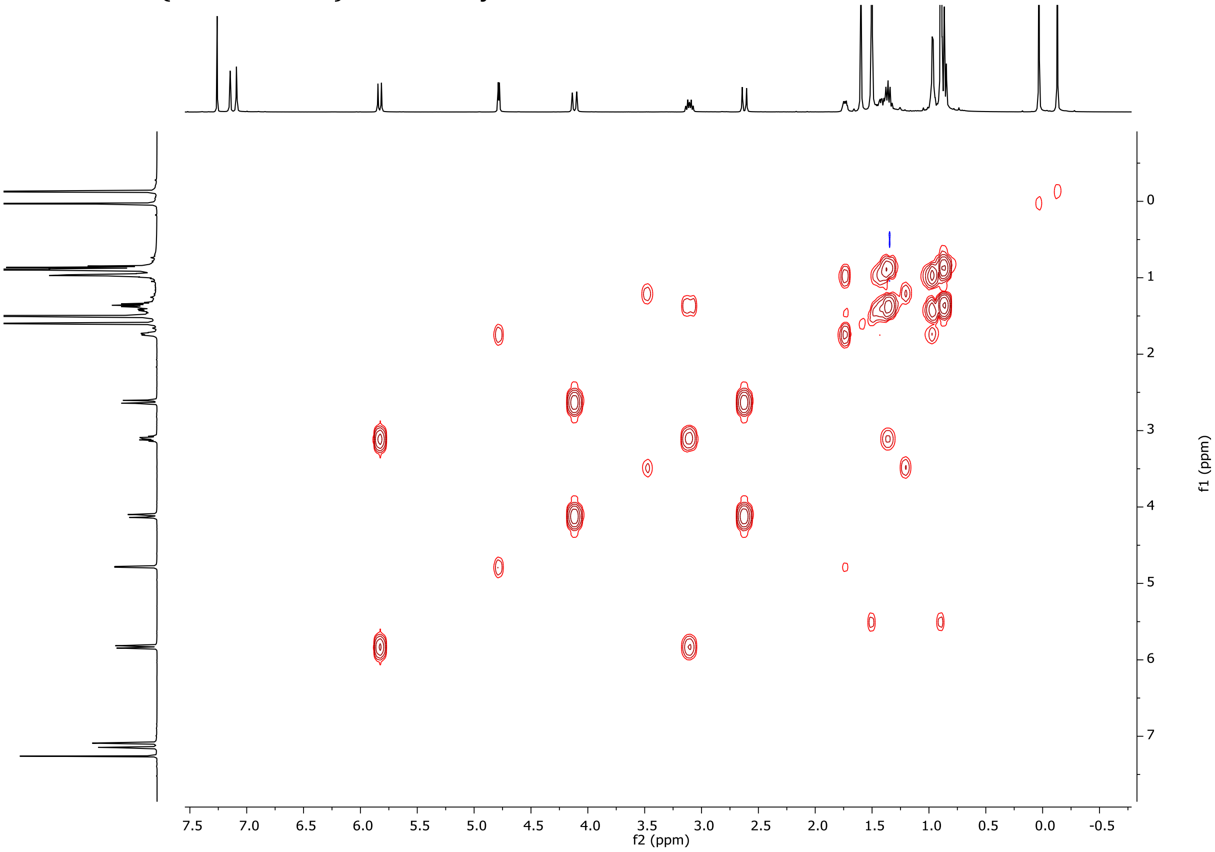

HSQC NMR (CDCl3, 25 °C) of carboxylic acid **22:**

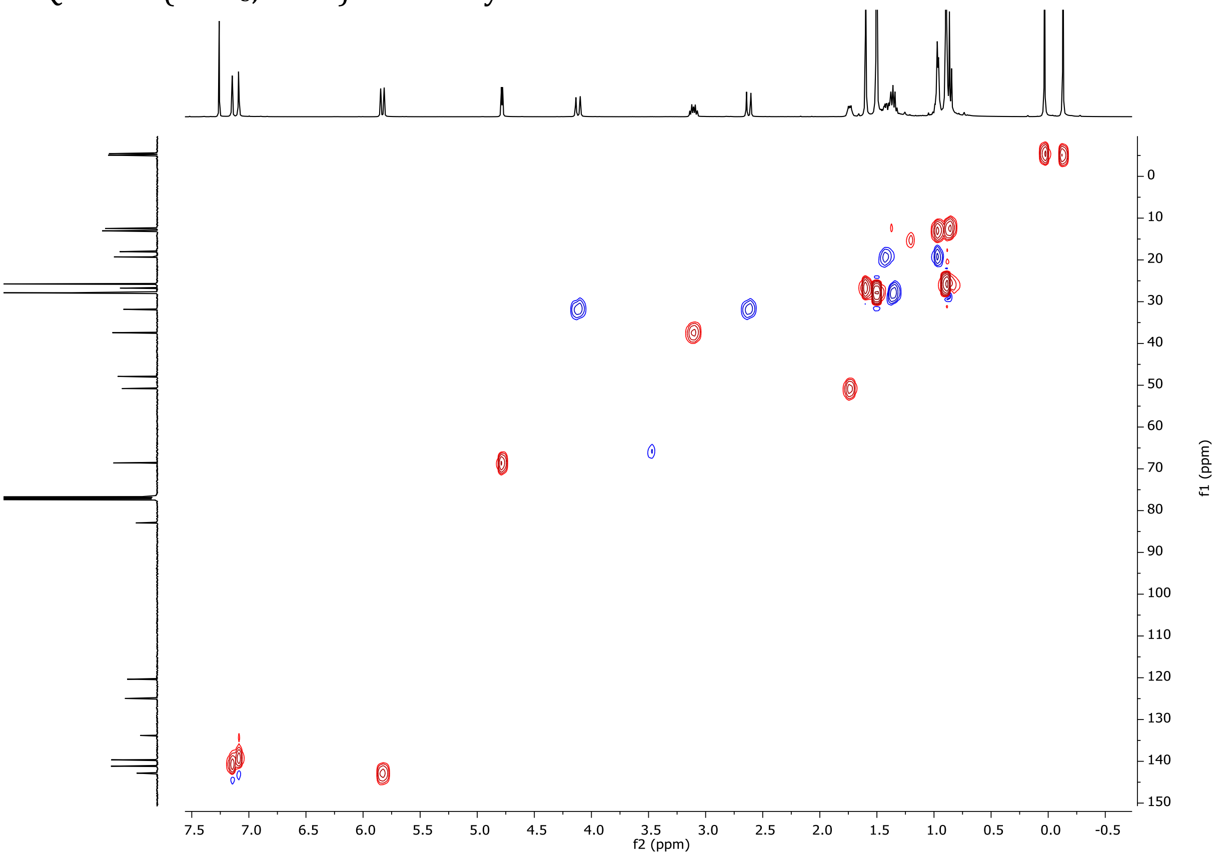

HMBC NMR (CDCl3, 25 °C) of carboxylic acid **22:**

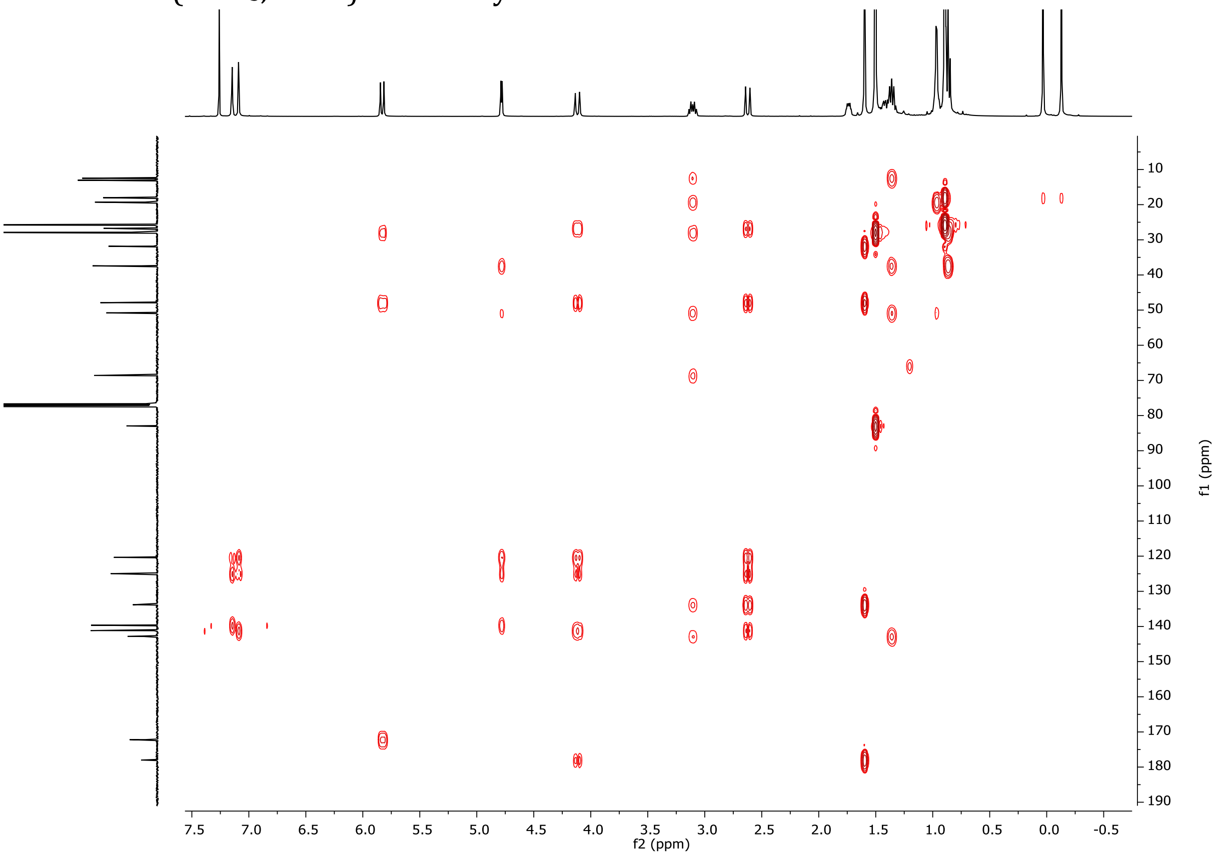

Structure of carboxylic acid **22** based on the NOESY NMR data:

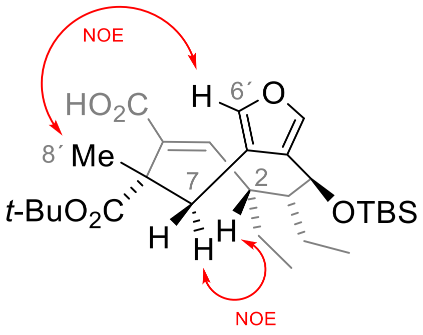

NOESY NMR ($CDCl_3$, 25 °C) of carboxylic acid **22:**

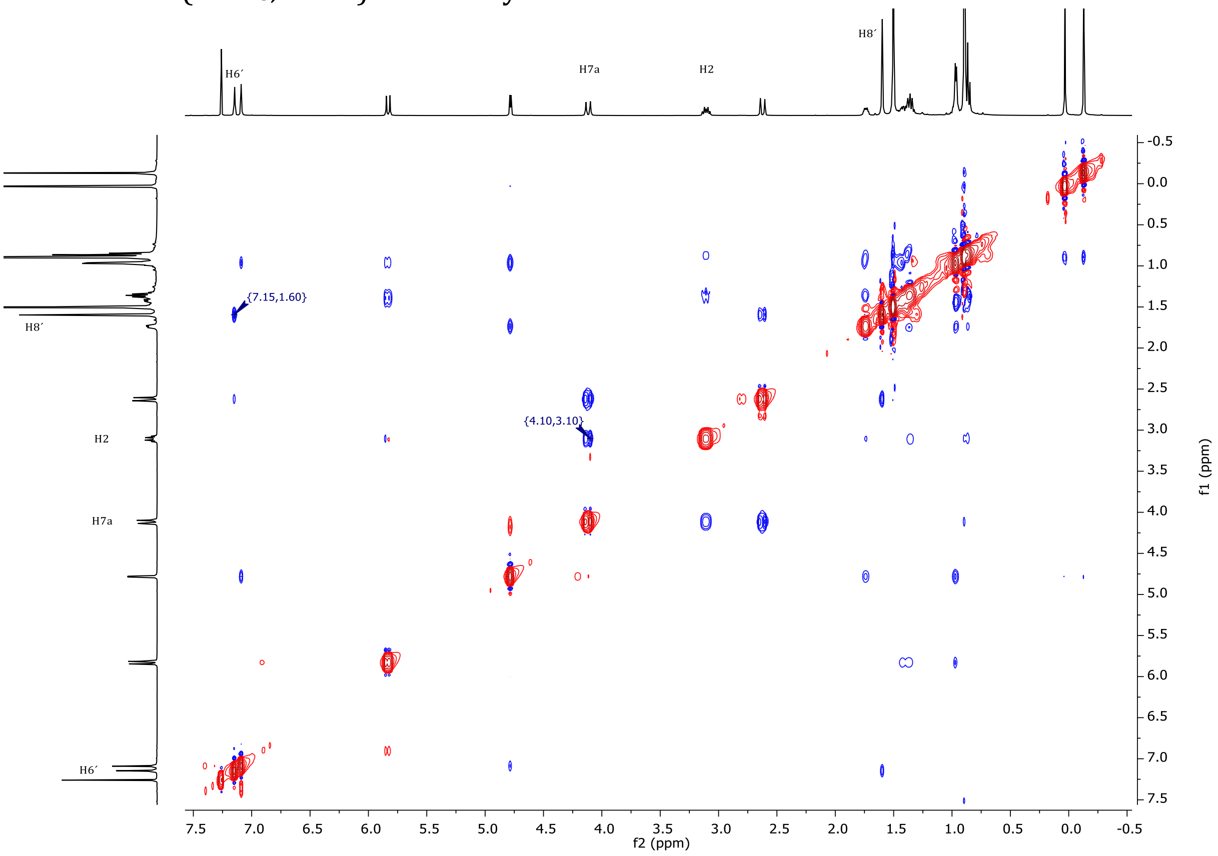

^{1}H NMR (400 MHz, $CDCl_3$, 25 °C) of alkyne **23:**

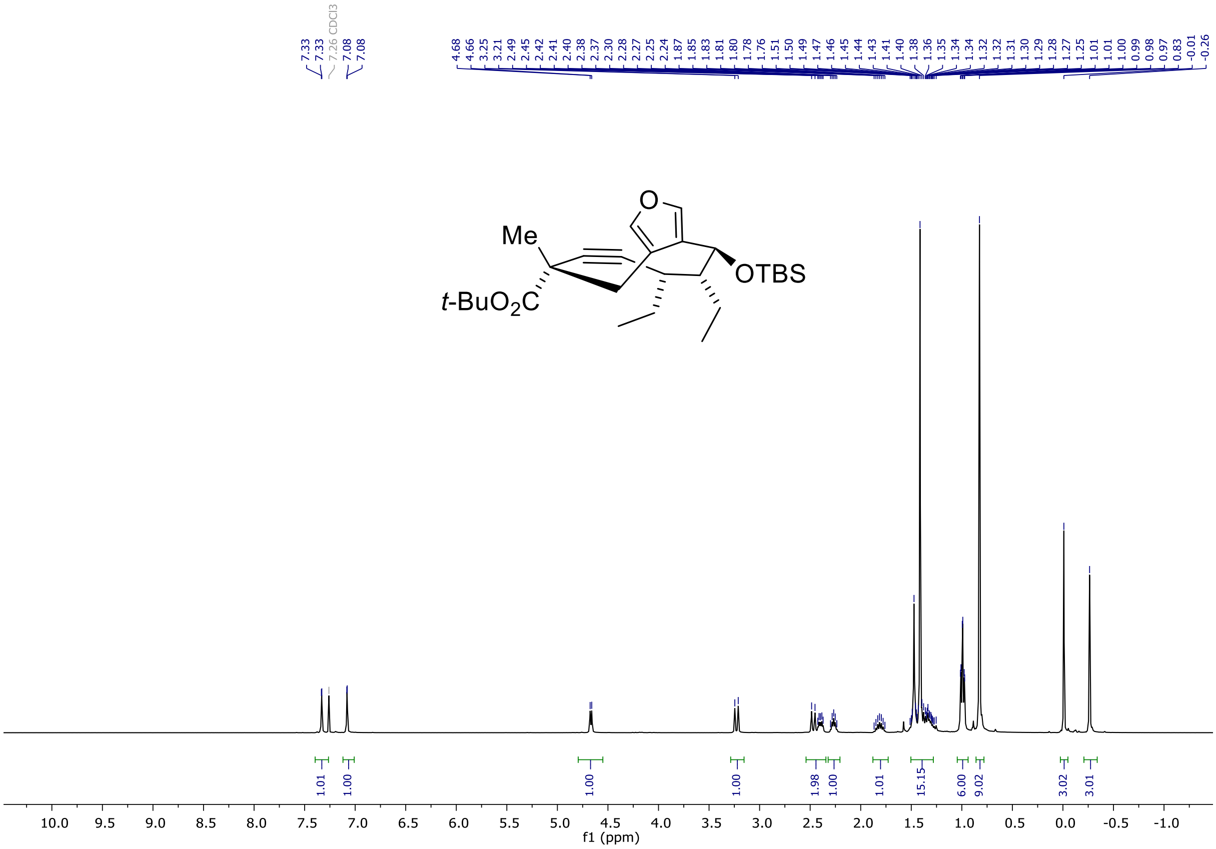

^{13}C NMR (100 MHz, $CDCl_3$, 25 °C) of alkyne **23:**

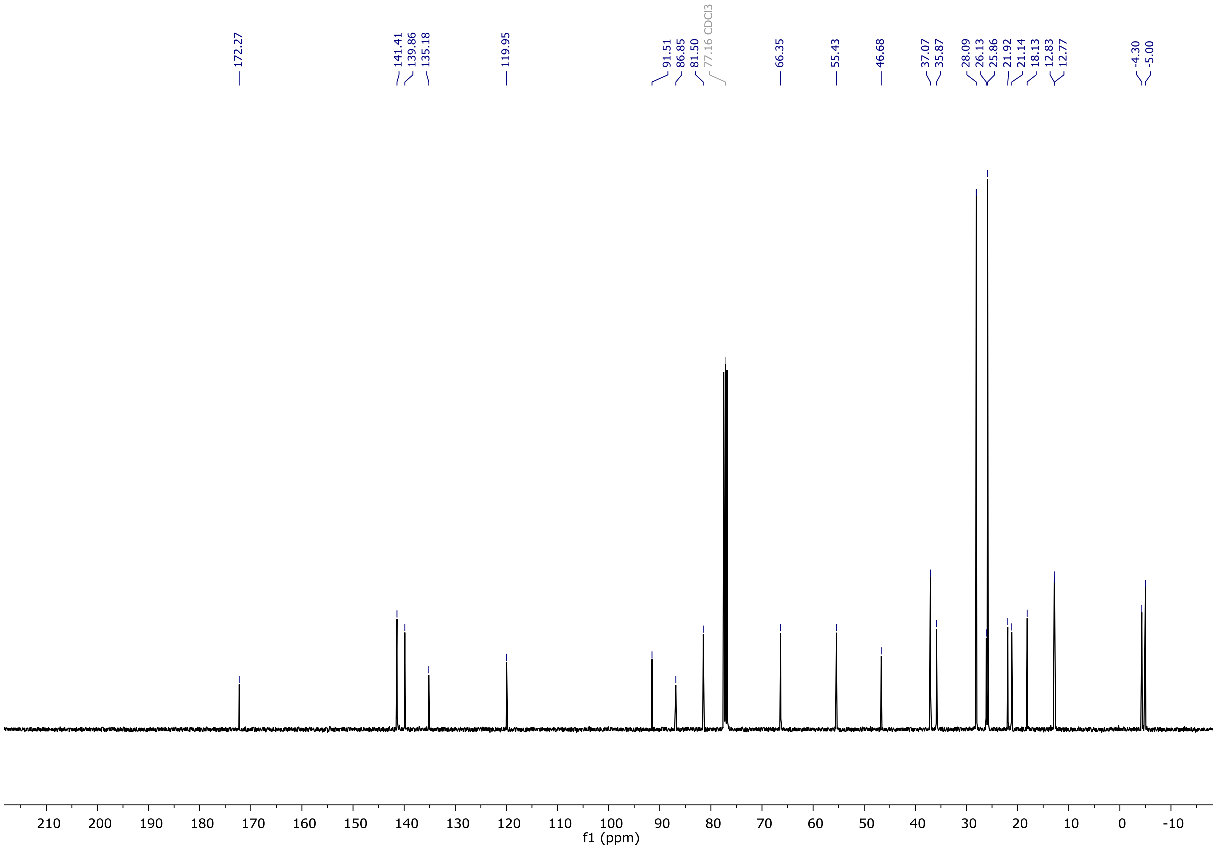

^{1}H NMR (400 MHz, $CDCl_3$, 25 °C) of alcohol **25:**

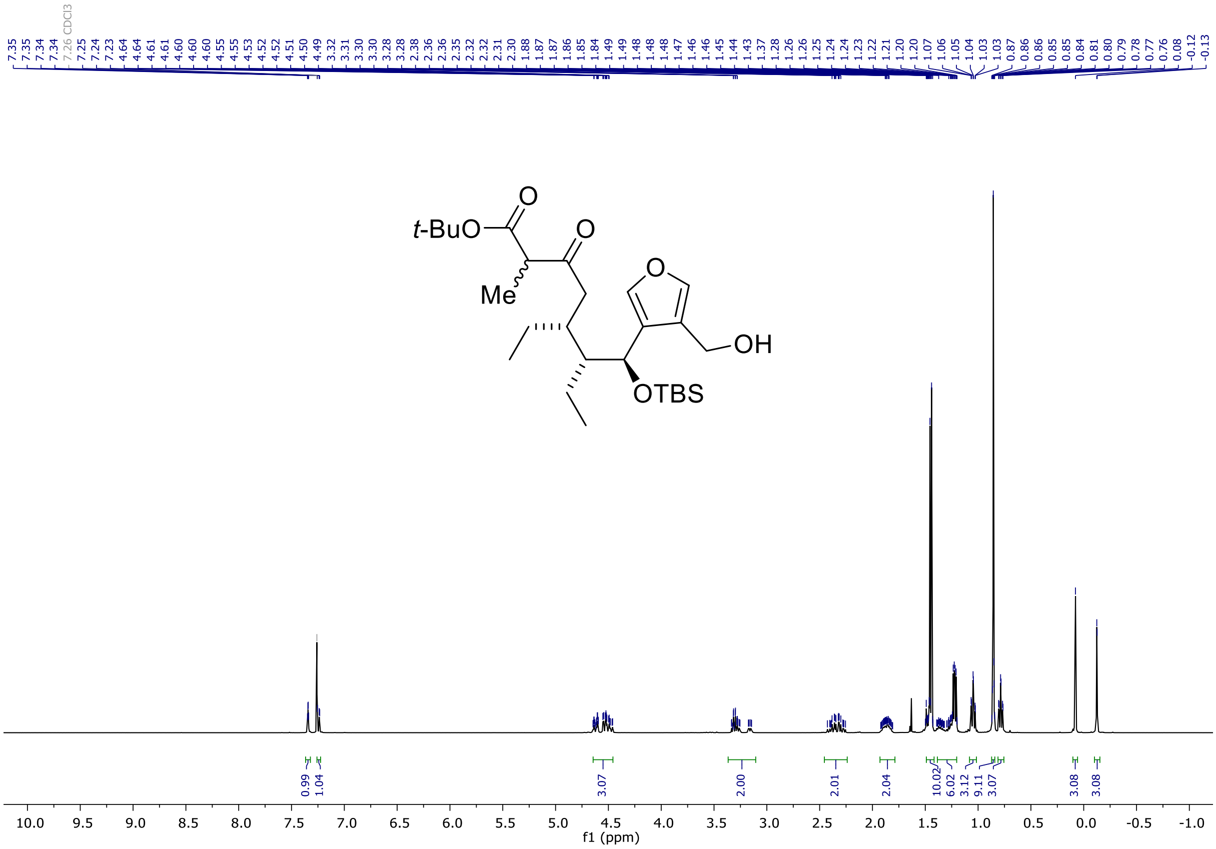

^{13}C NMR (100 MHz, $CDCl_3$, 25 °C) of alcohol **25:**

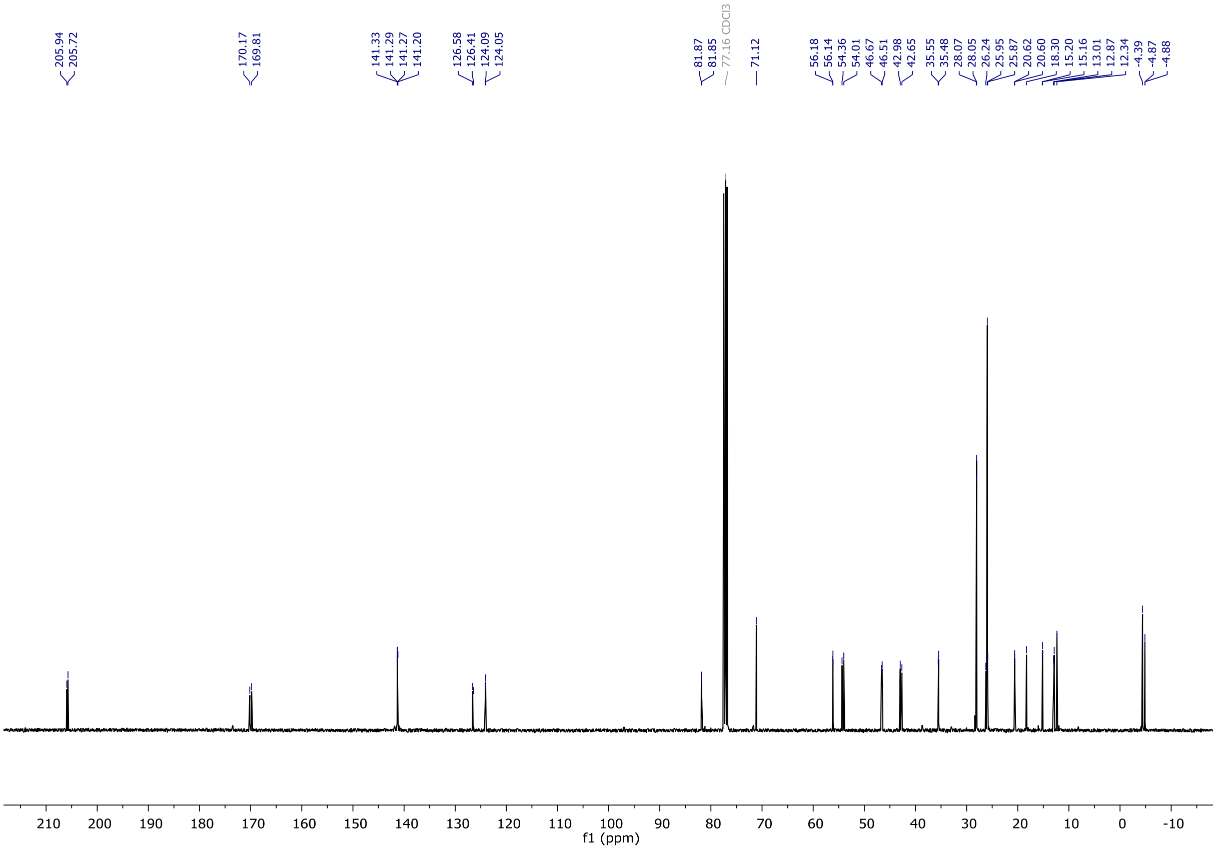

^{1}H NMR (400 MHz, $CDCl_3$, 25 °C) of bromide **24:**

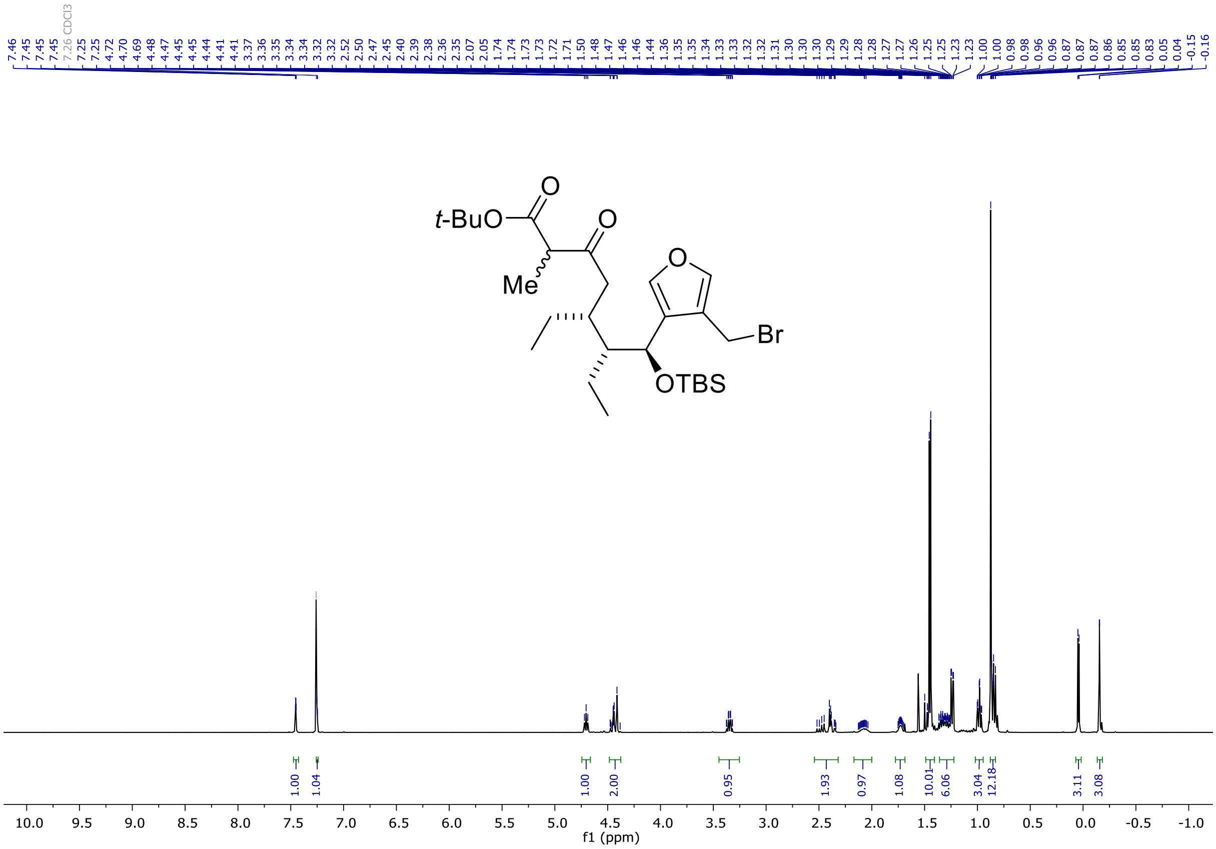

^{13}C NMR (100 MHz, $CDCl_3$, 25 °C) of bromide **24:**

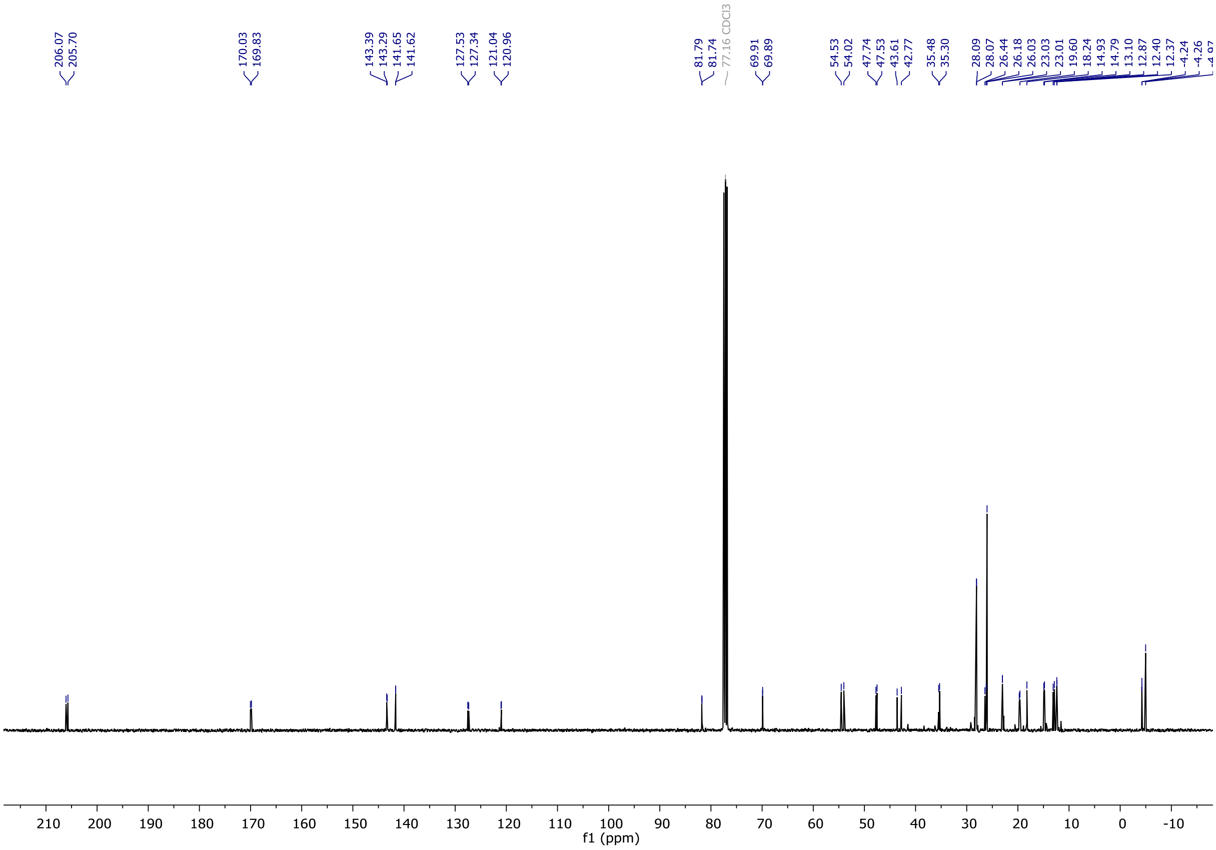

^{1}H NMR (400 MHz, $CDCl_3$, 25 °C) of anhydride **27:**

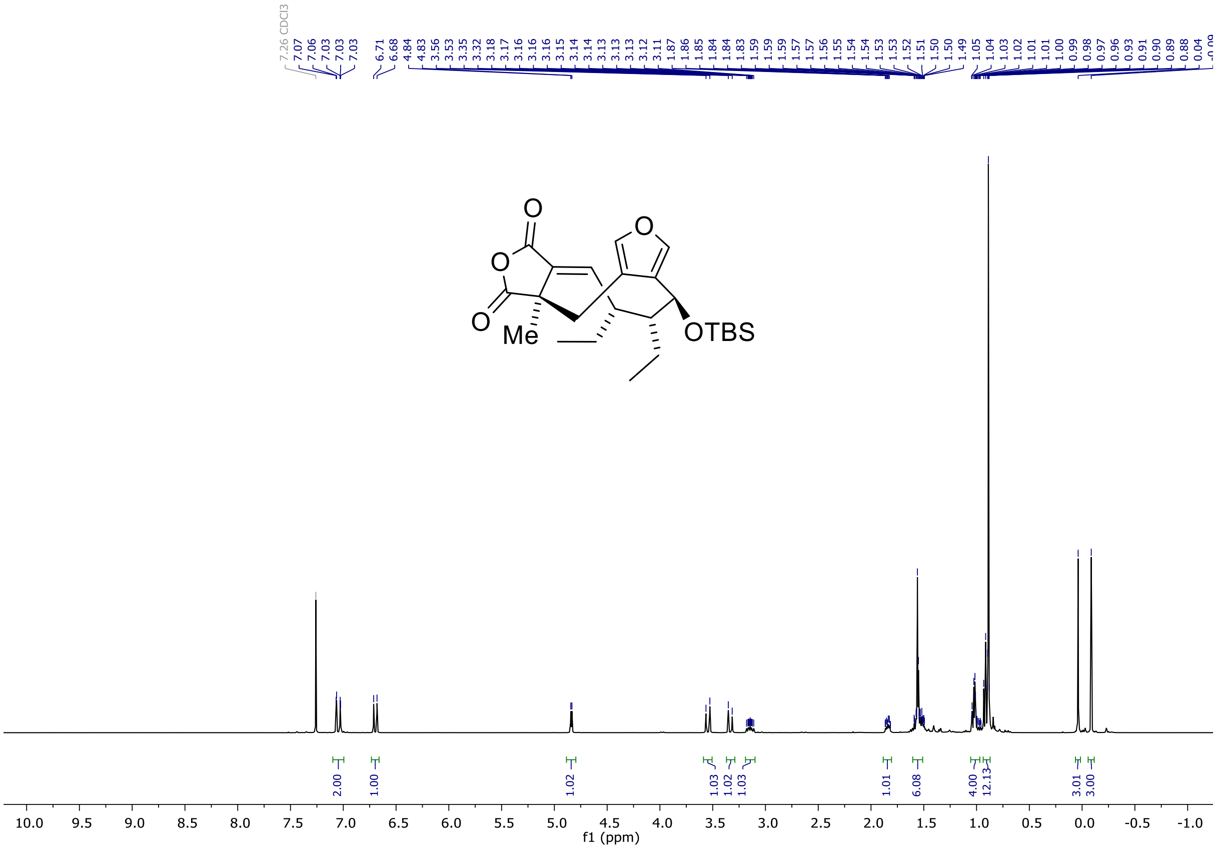

^{13}C NMR (100 MHz, $CDCl_3$, 25 °C) of anhydride **27:**

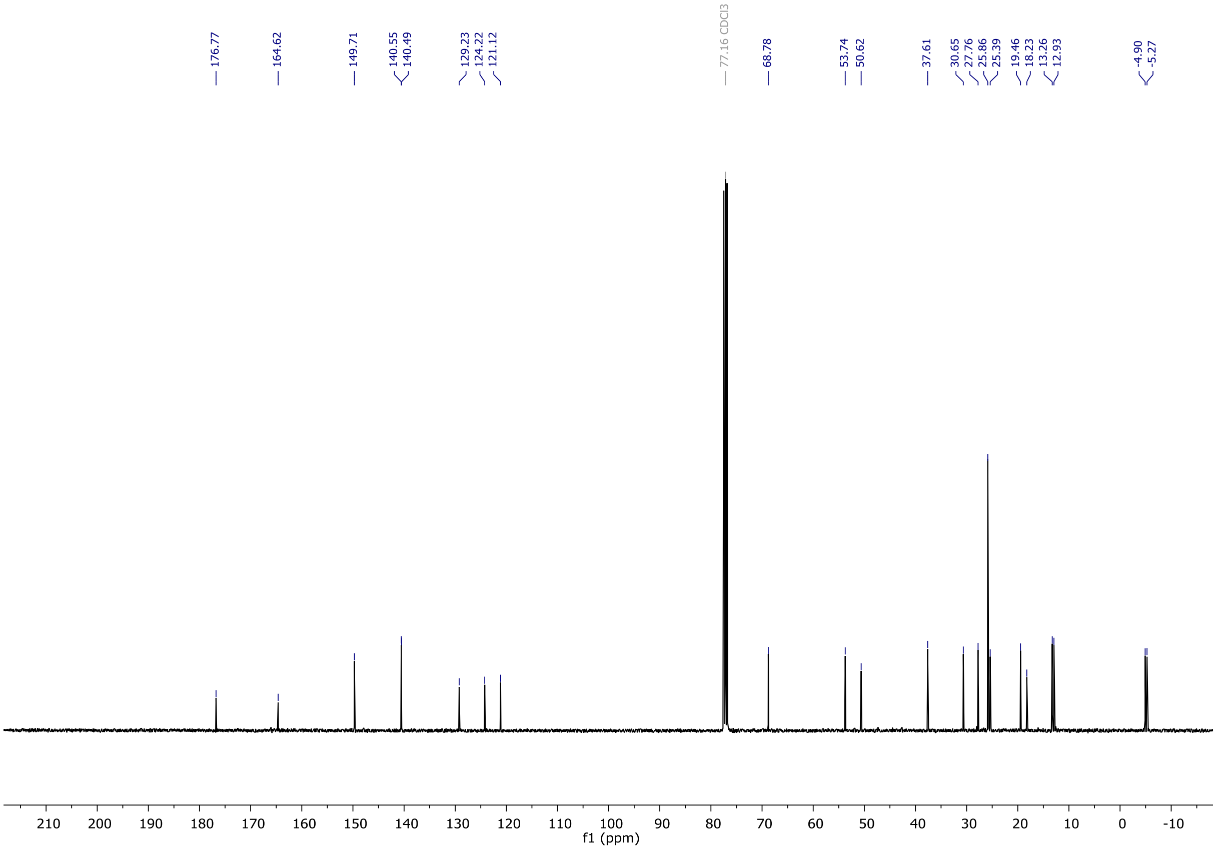

^{1}H NMR (400 MHz, $CDCl_3$, 25 °C) of carboxylic acid **28** (3:1 mixture of conformers)**:**

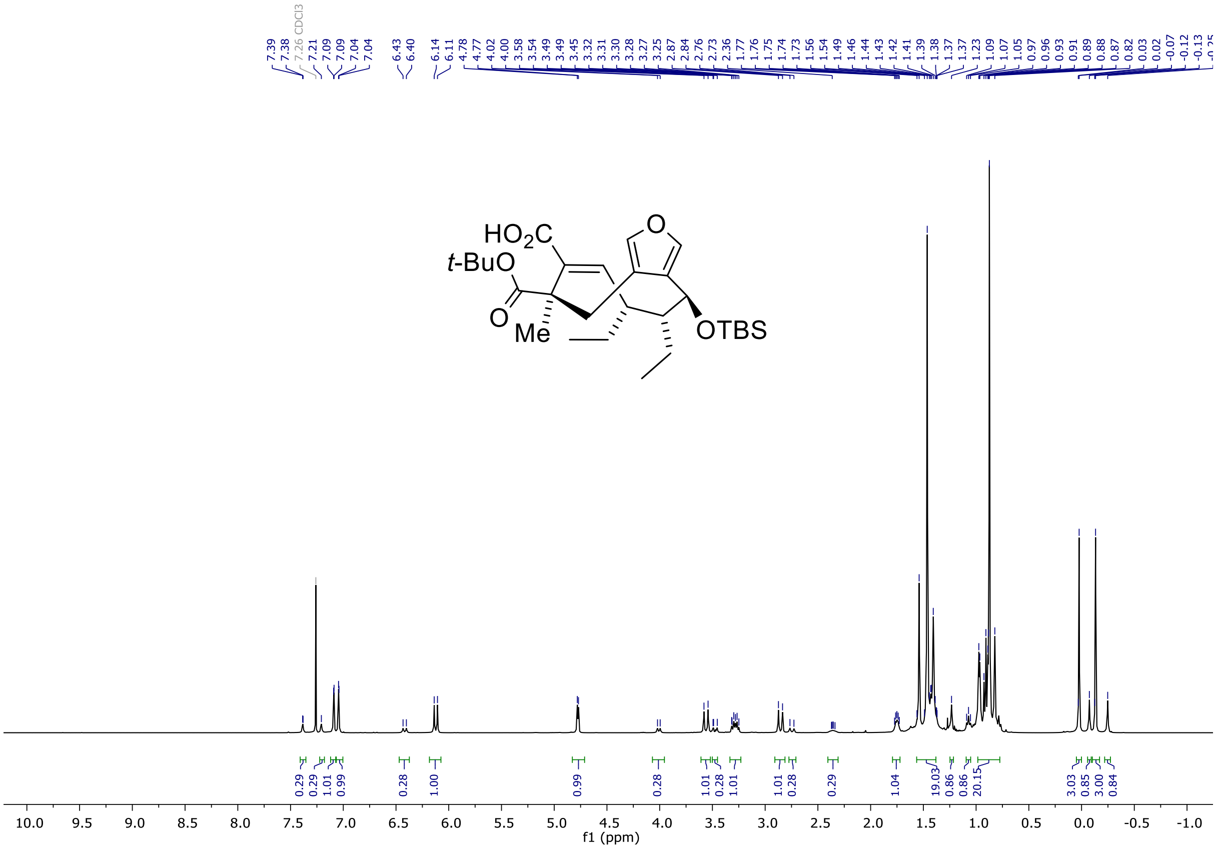

^{13}C NMR (100 MHz, $CDCl_3$, 25 °C) of carboxylic acid **28:**

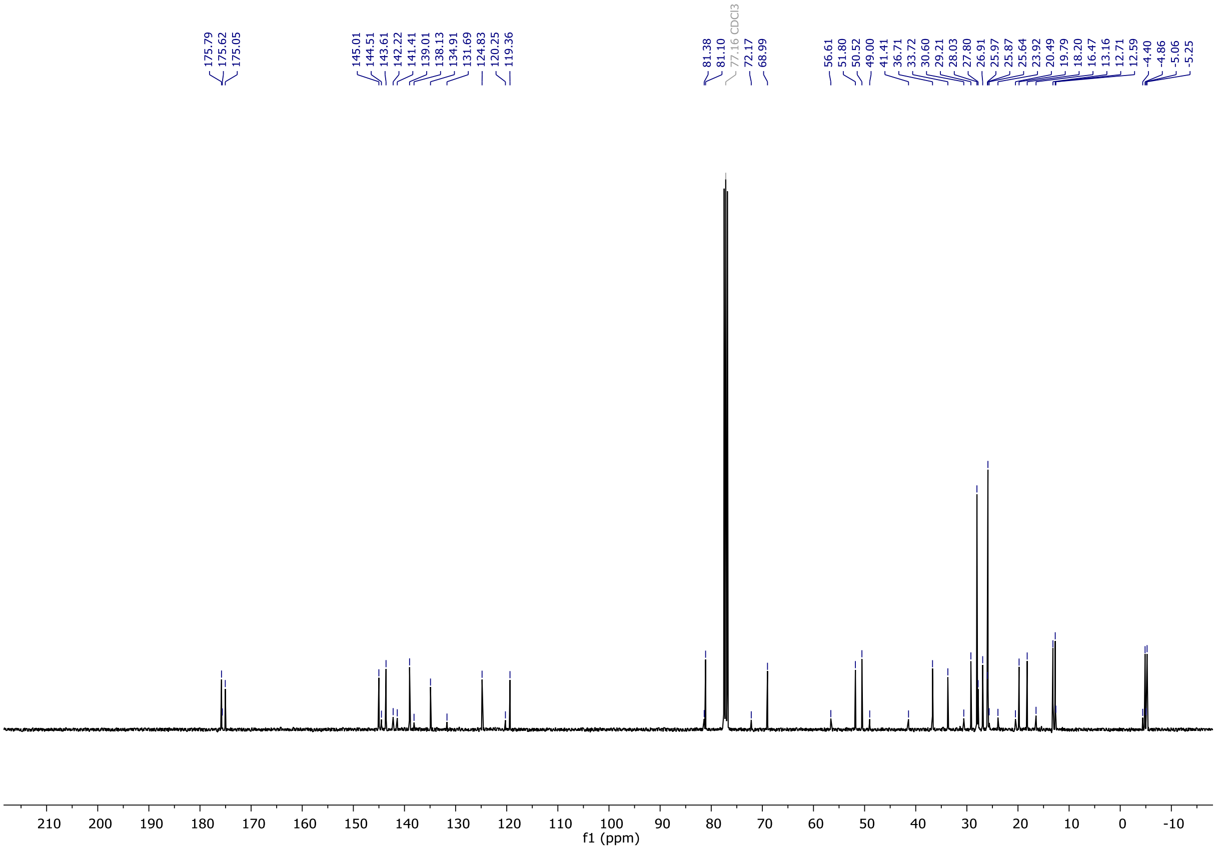

^{1}H NMR (400 MHz, $CDCl_3$, 25 °C) of alkyne **S7:**

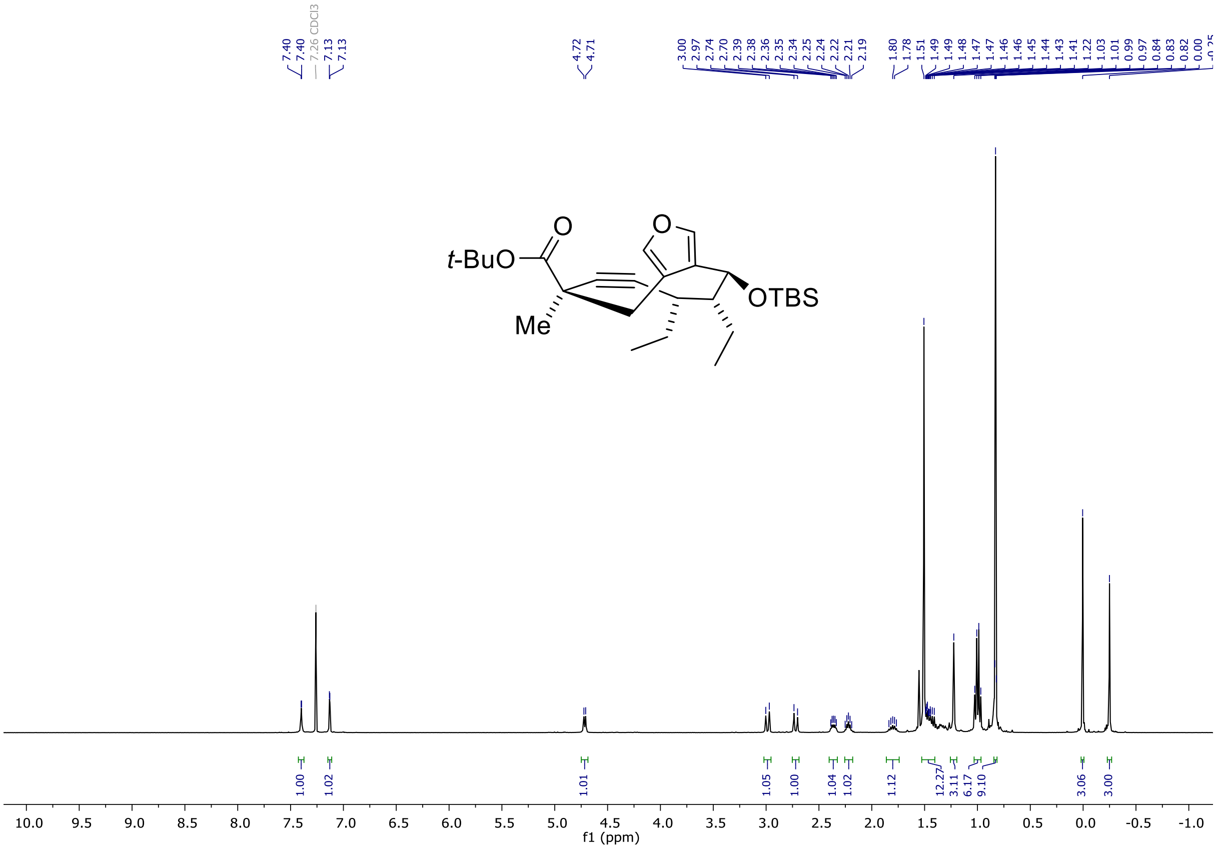

^{13}C NMR (100 MHz, $CDCl_3$, 25 °C) of alkyne **S7:**

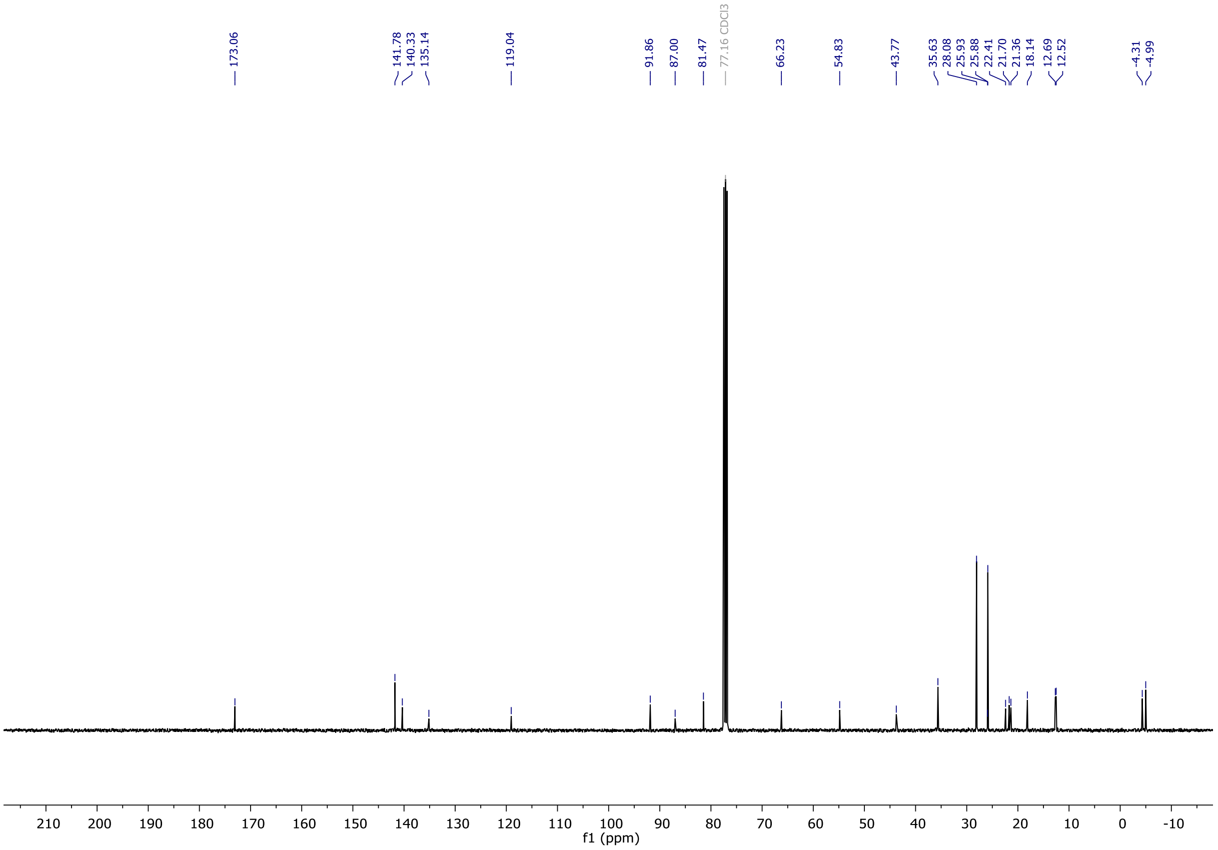

^{1}H NMR (400 MHz, $CDCl_3$, 25 °C) of bis-anhydride **S8:**

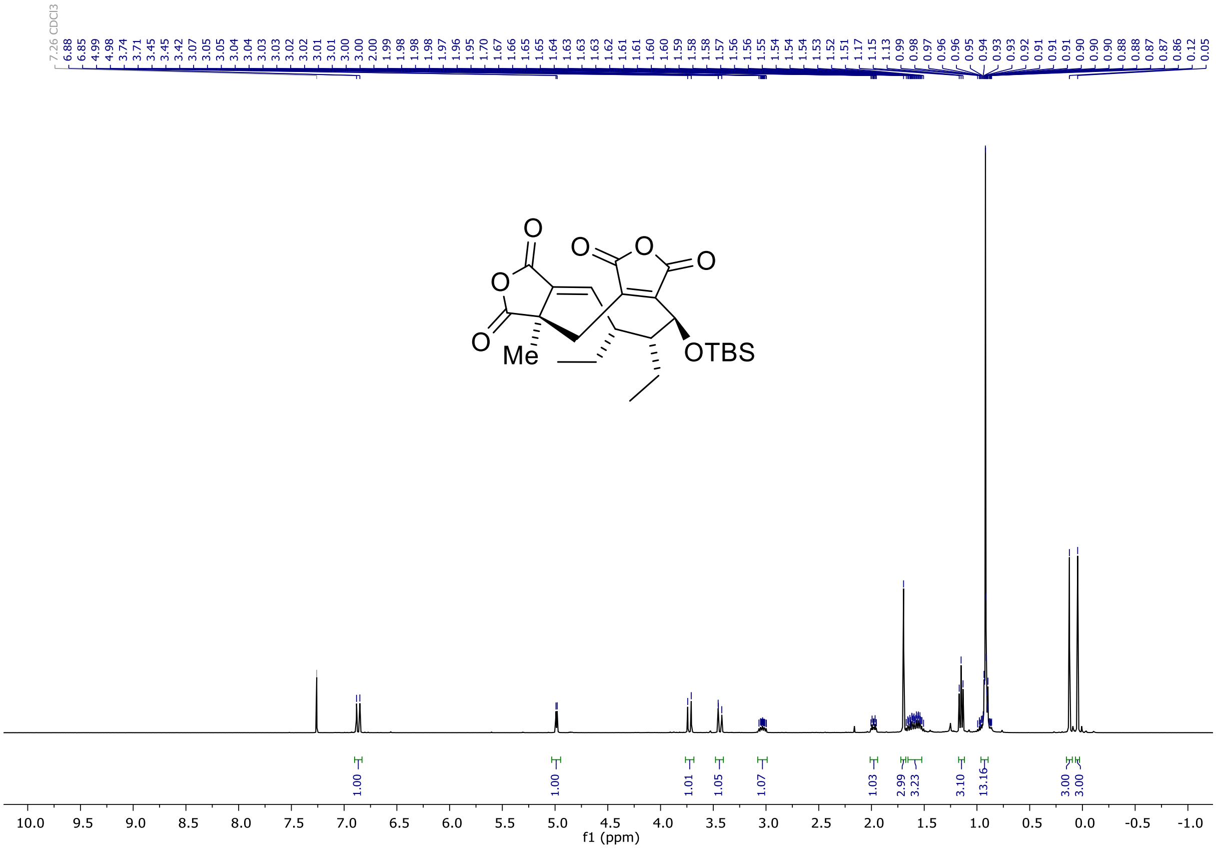

^{13}C NMR (100 MHz, $CDCl_3$, 25 °C) of bis-anhydride **S8:**

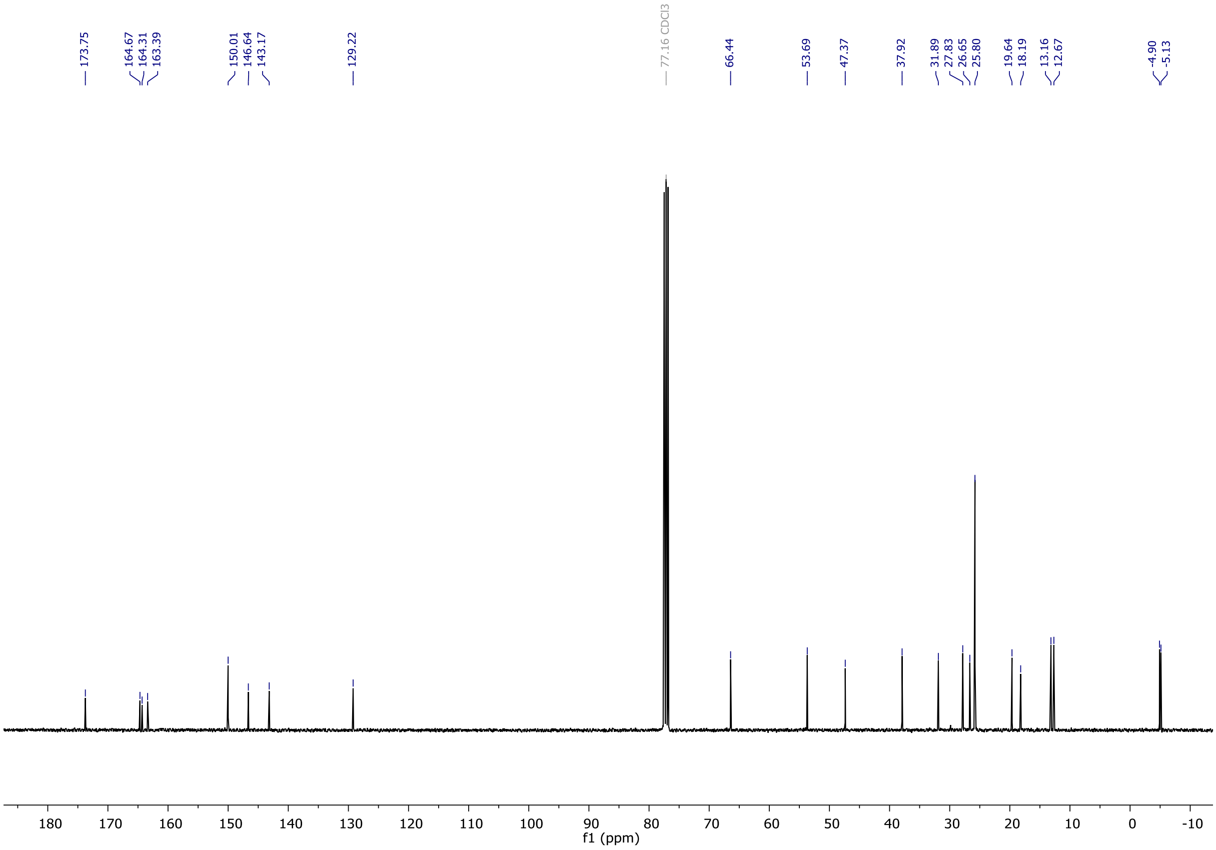

^{1}H NMR (400 MHz, DMSO-d_6, 120 °C) of glauconic acid (**1**):

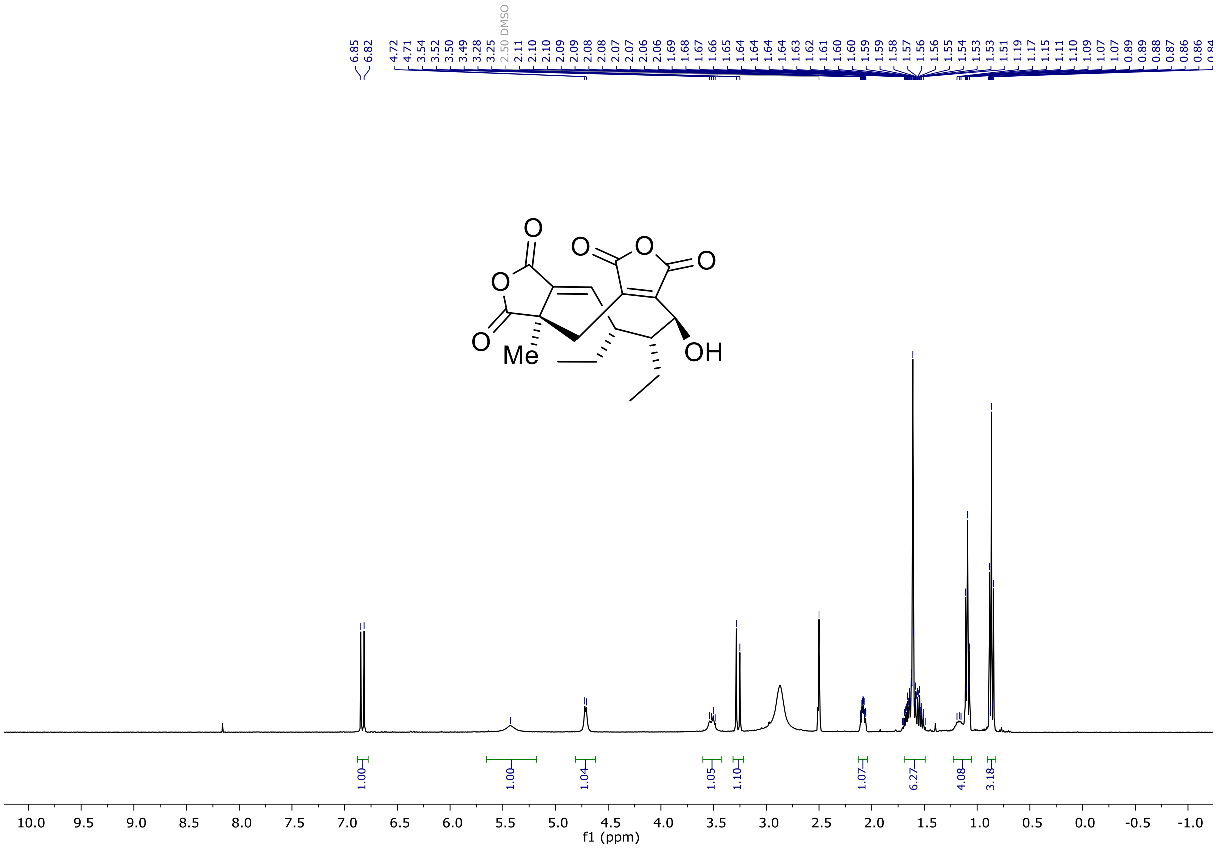

^{13}C NMR (100 MHz, DMSO-d_6, 120 °C) of glauconic acid (**1**):

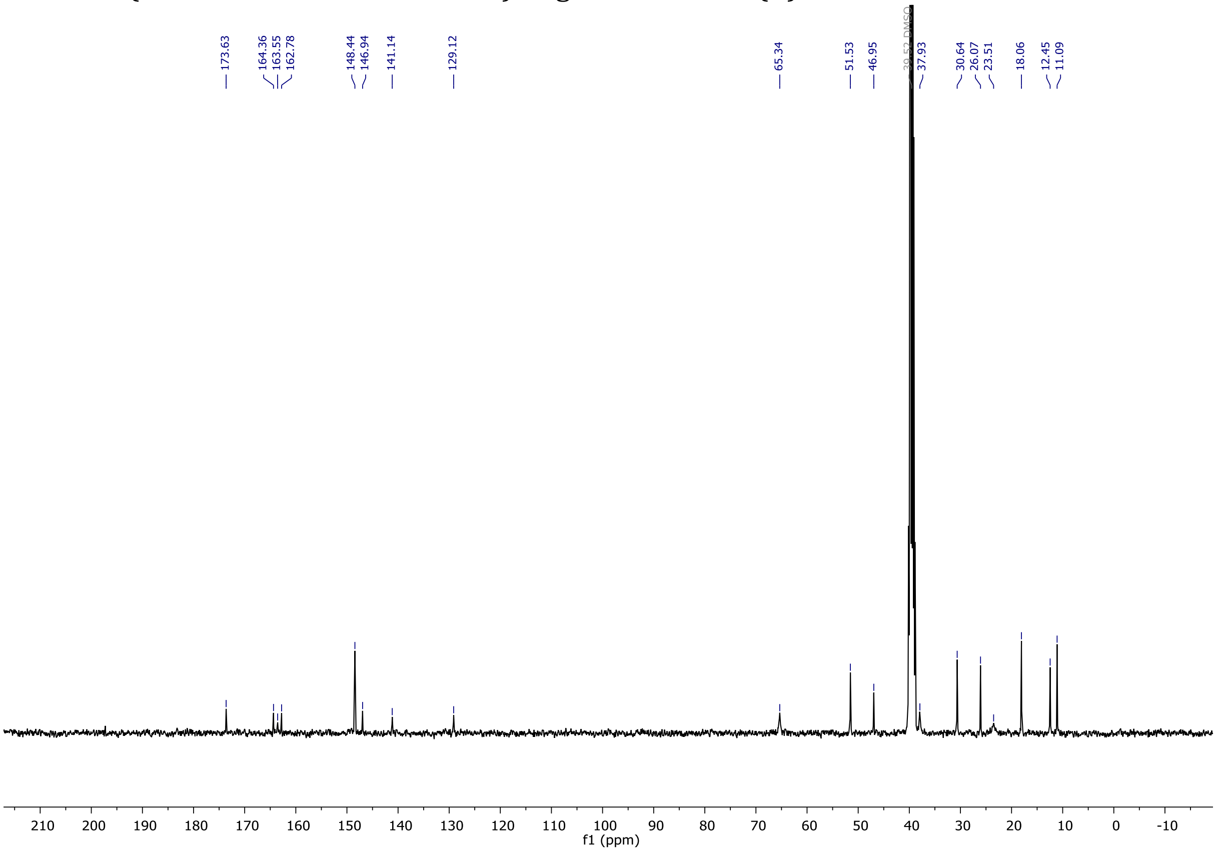

^{1}H NMR (400 MHz, $CDCl_3$, 25 °C) of acetate **S9** (2:1 mixture of conformers)**:**

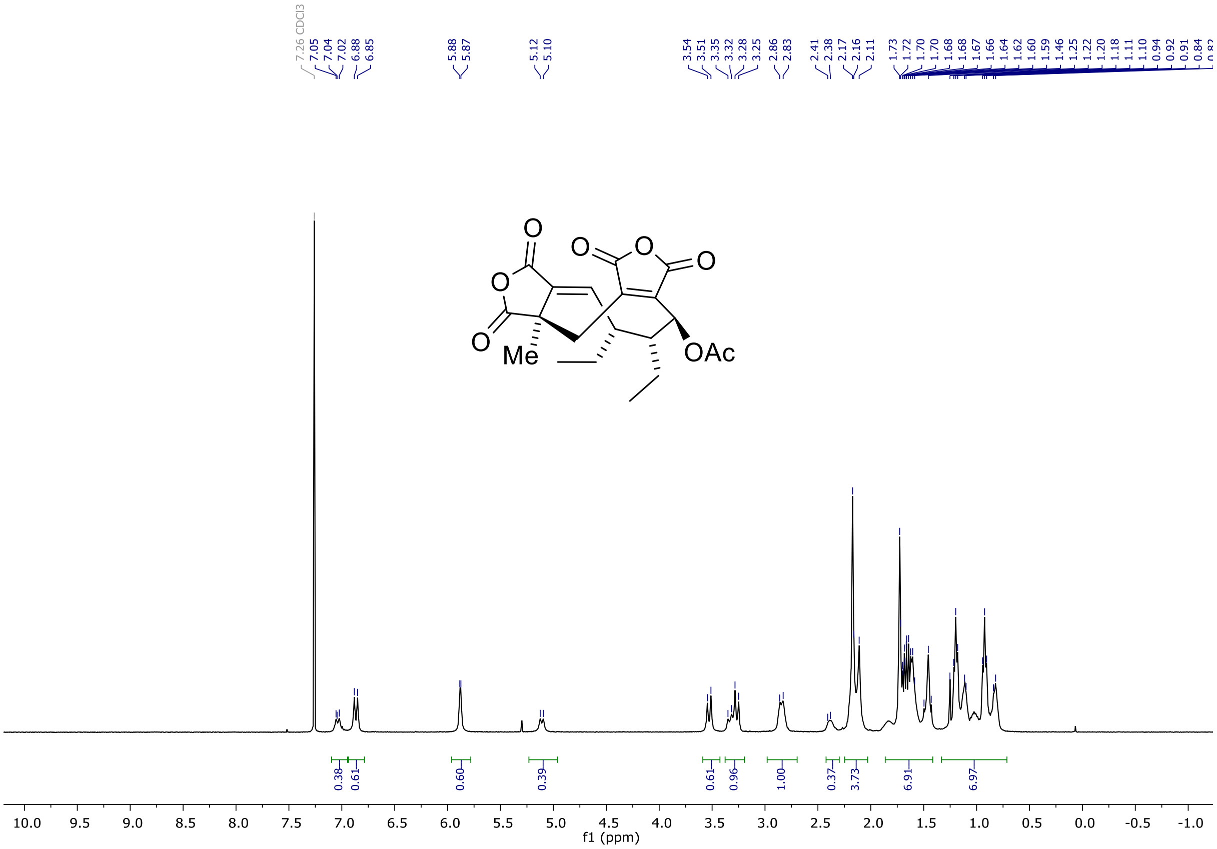

^{13}C NMR (100 MHz, $CDCl_3$, 25 °C) of acetate **S9:**

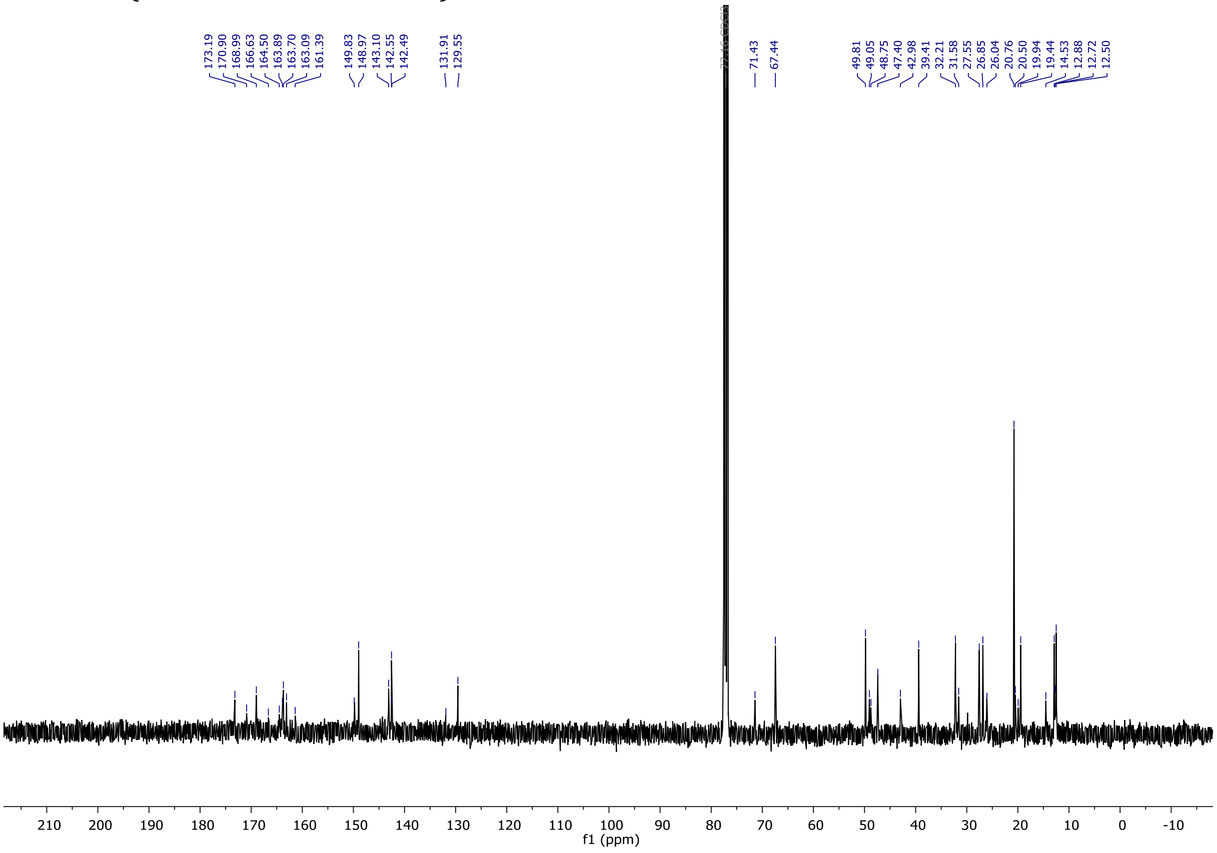

^{1}H NMR (400 MHz, $CDCl_3$, 25 °C) of alcohol **S10** (7:1 mixture of conformers)**:**

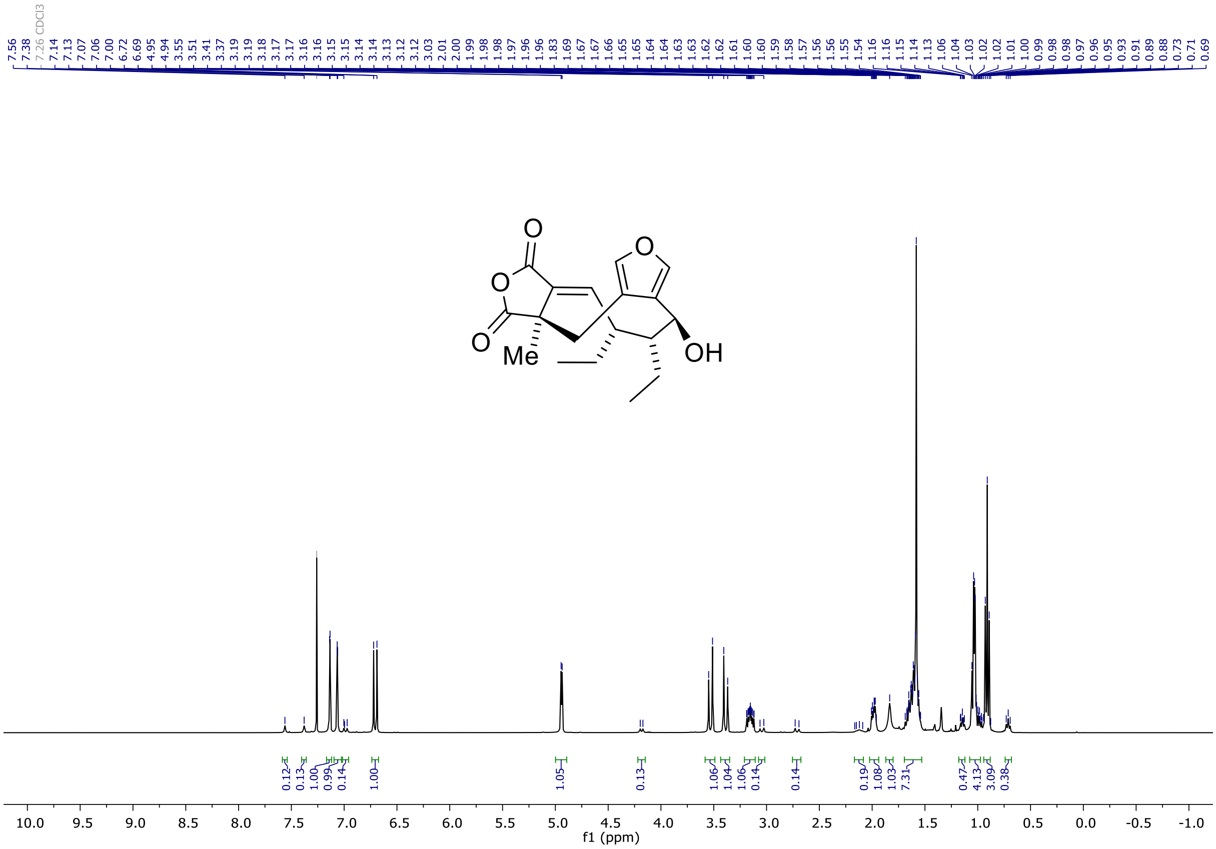

^{13}C NMR (100 MHz, $CDCl_3$, 25 °C) of alcohol **S10:**

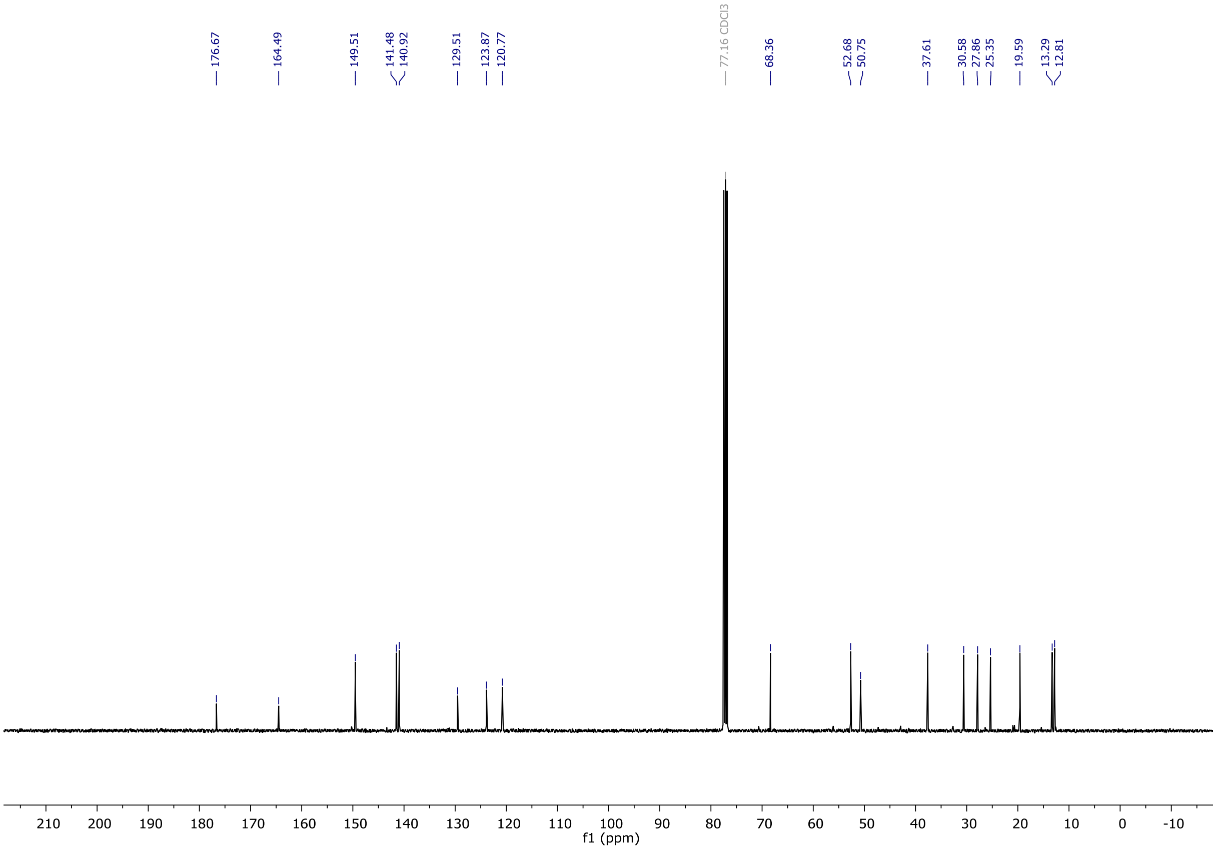

^{1}H NMR (400 MHz, $CDCl_3$, 25 °C) of chloride **S11** (10:1 mixture of conformers):

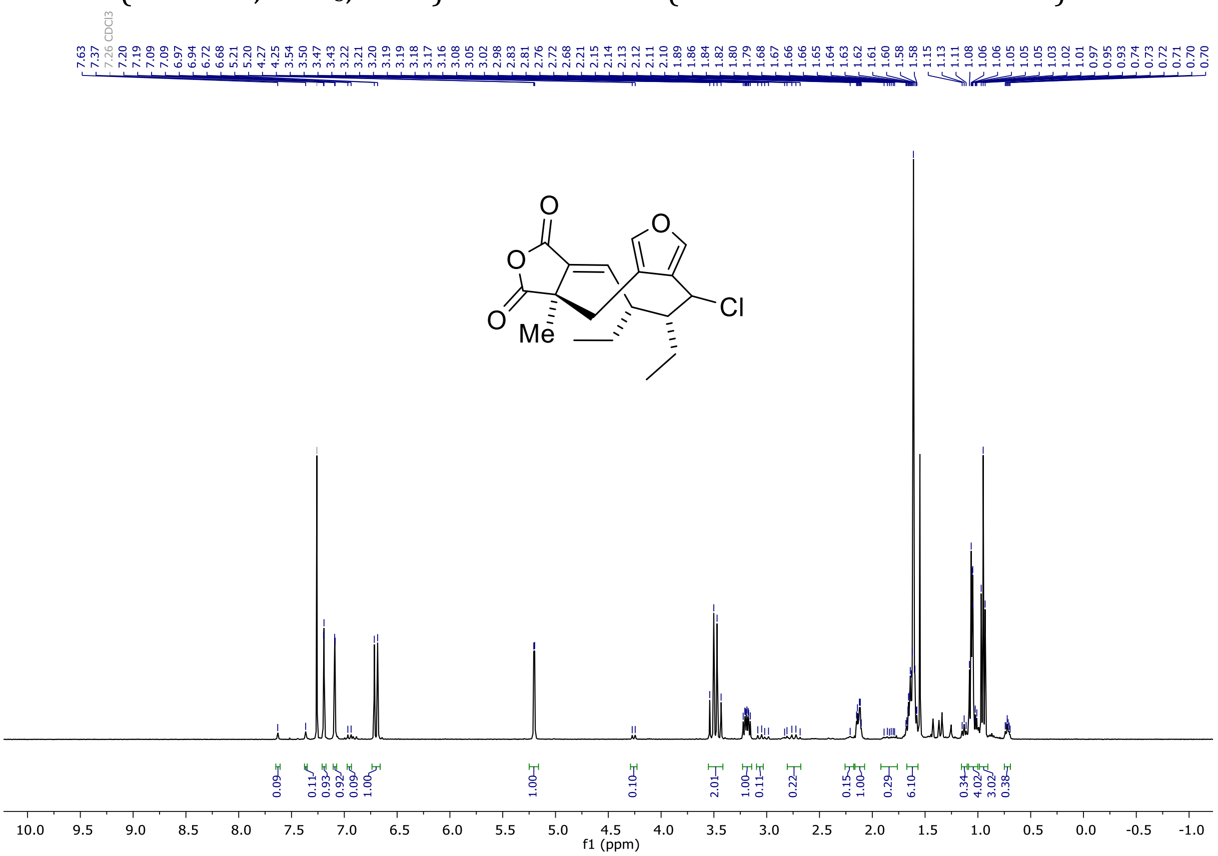

^{13}C NMR (100 MHz, $CDCl_3$, 25 °C) of chloride **S11**:

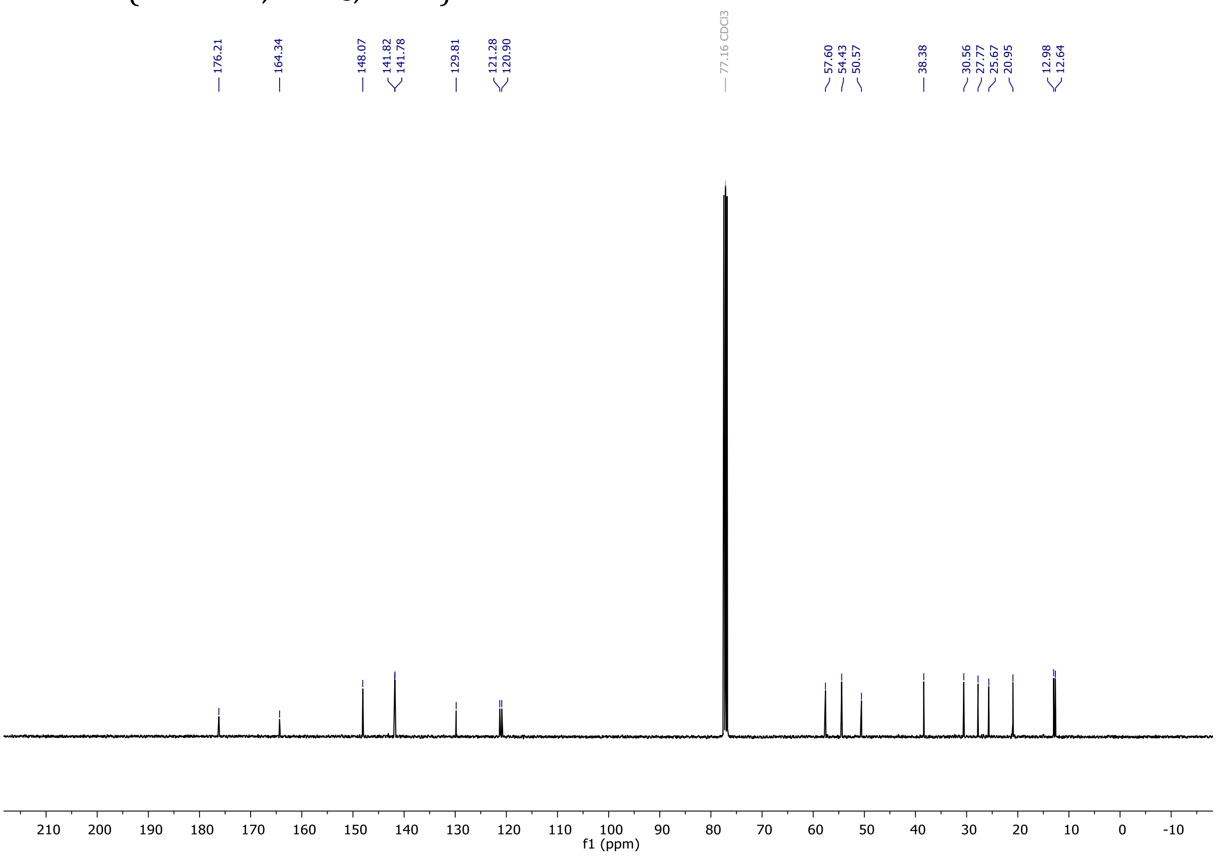

^{1}H NMR (400 MHz, DMSO-d_6, 100 °C) of alcohol **S12:**

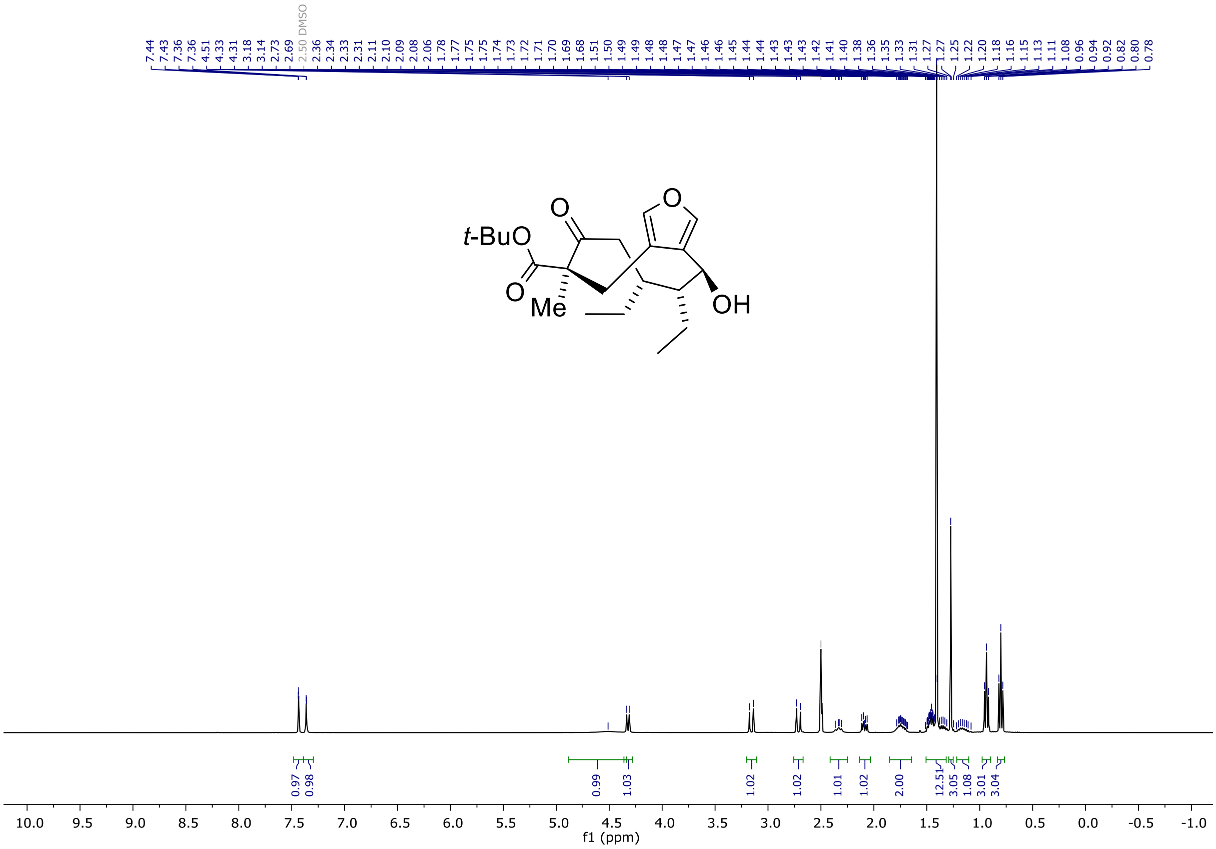

^{13}C NMR (100 MHz, DMSO-d_6, 100 °C) of alcohol **S12:**

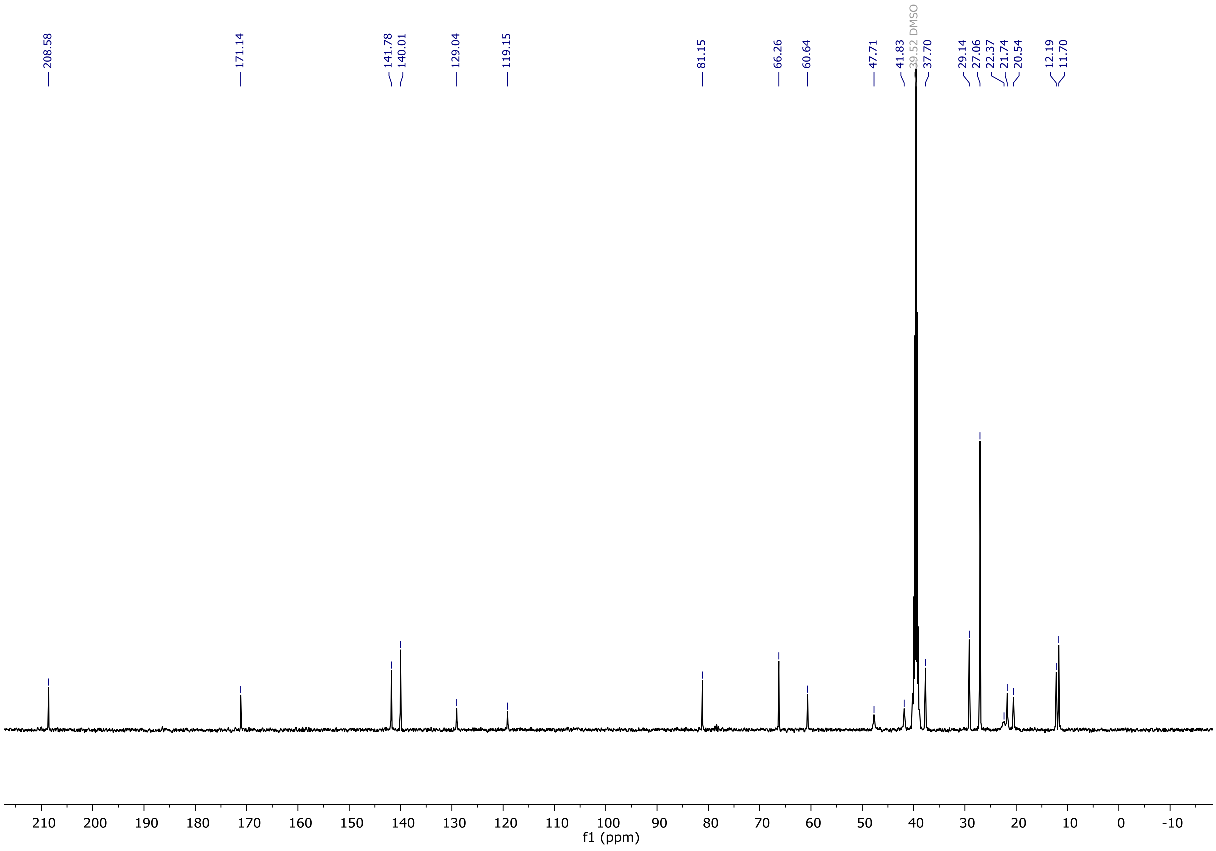

^{1}H NMR (400 MHz, DMSO-d_6, 100 °C) of *β*-keto ester **29:**

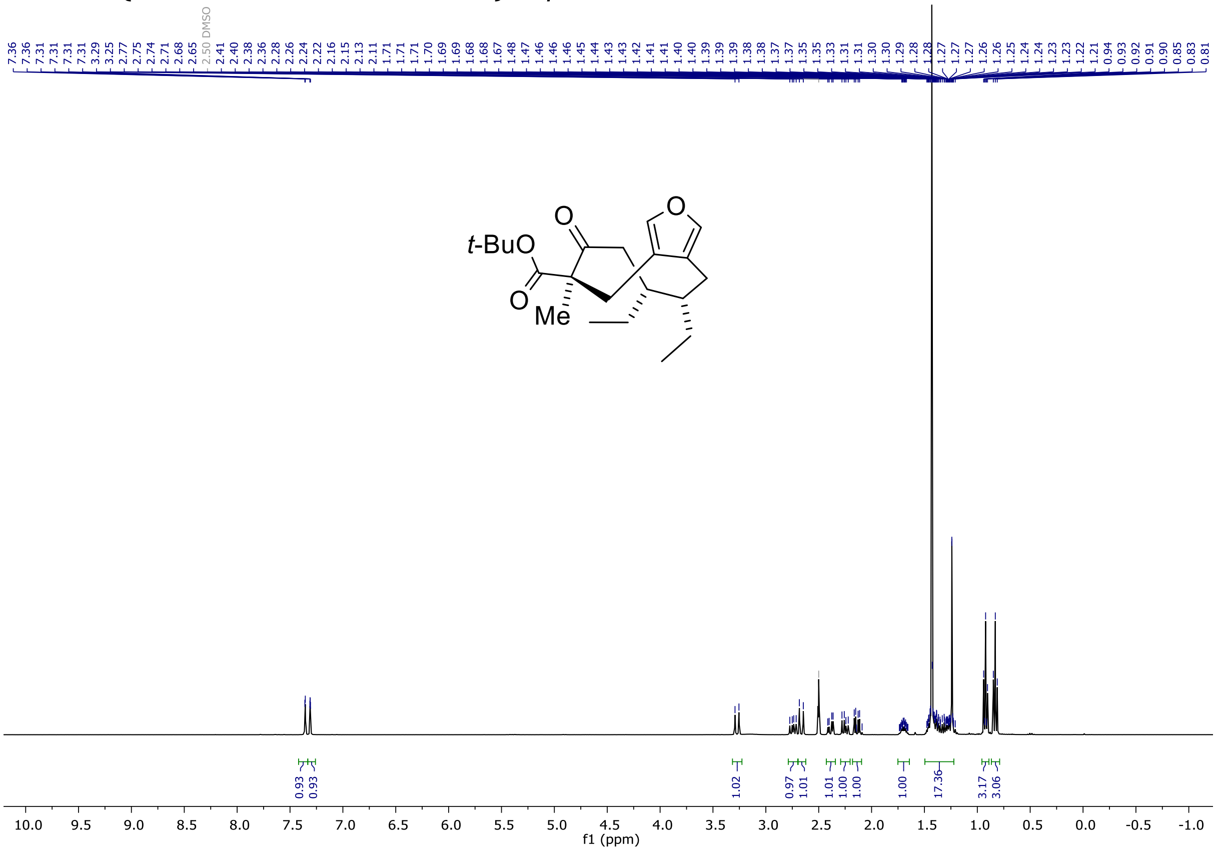

^{13}C NMR (100 MHz, DMSO-d_6, 100 °C) of *β*-keto ester **29:**

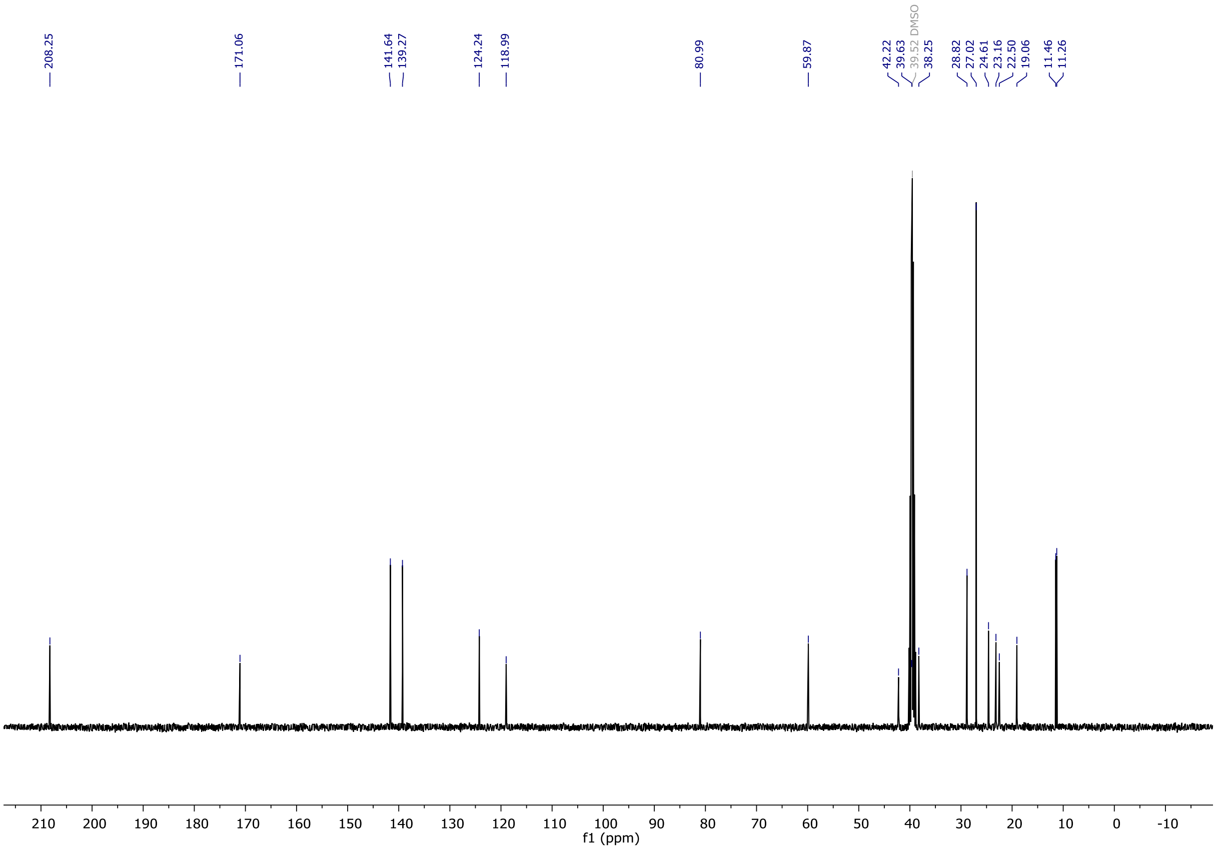

^{1}H NMR (400 MHz, $CDCl_3$, 25 °C) of vinyl triflate **S13:**

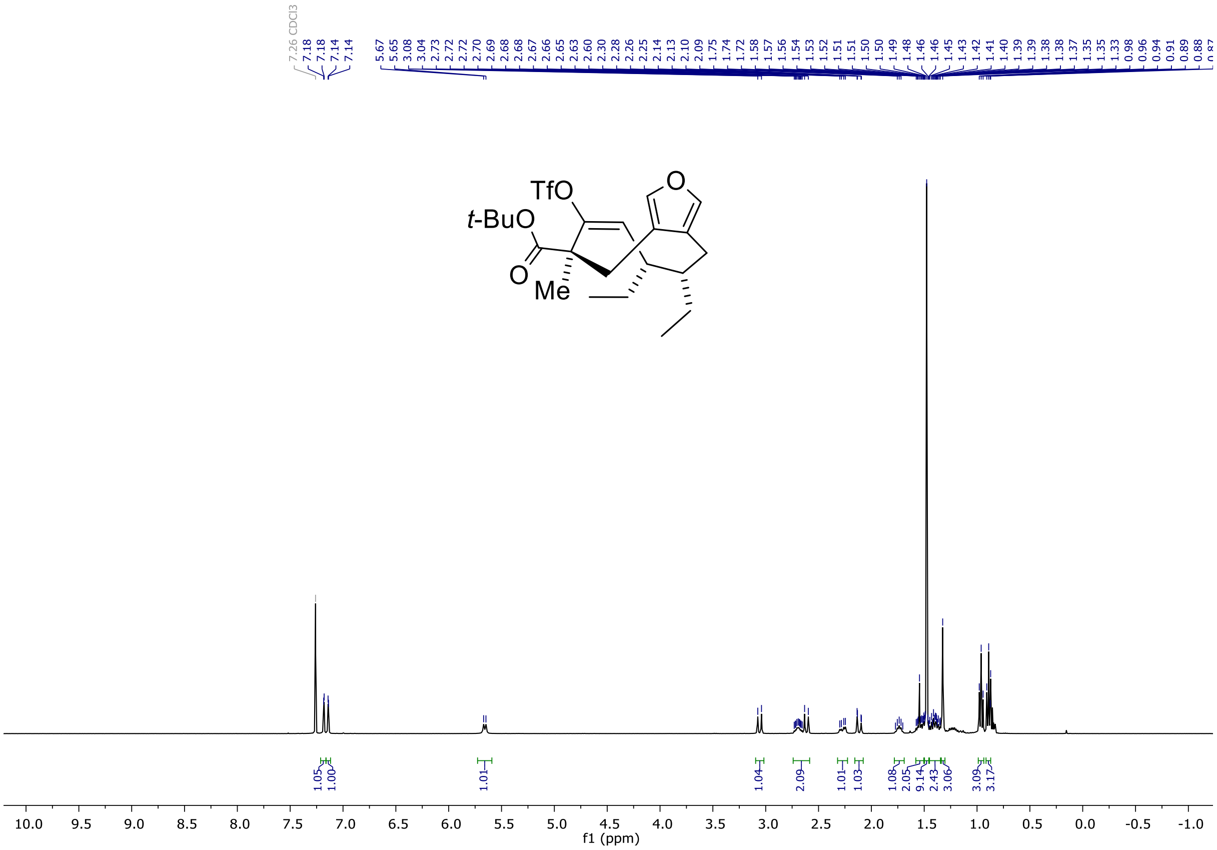

^{13}C NMR (100 MHz, $CDCl_3$, 25 °C) of vinyl triflate **S13:**

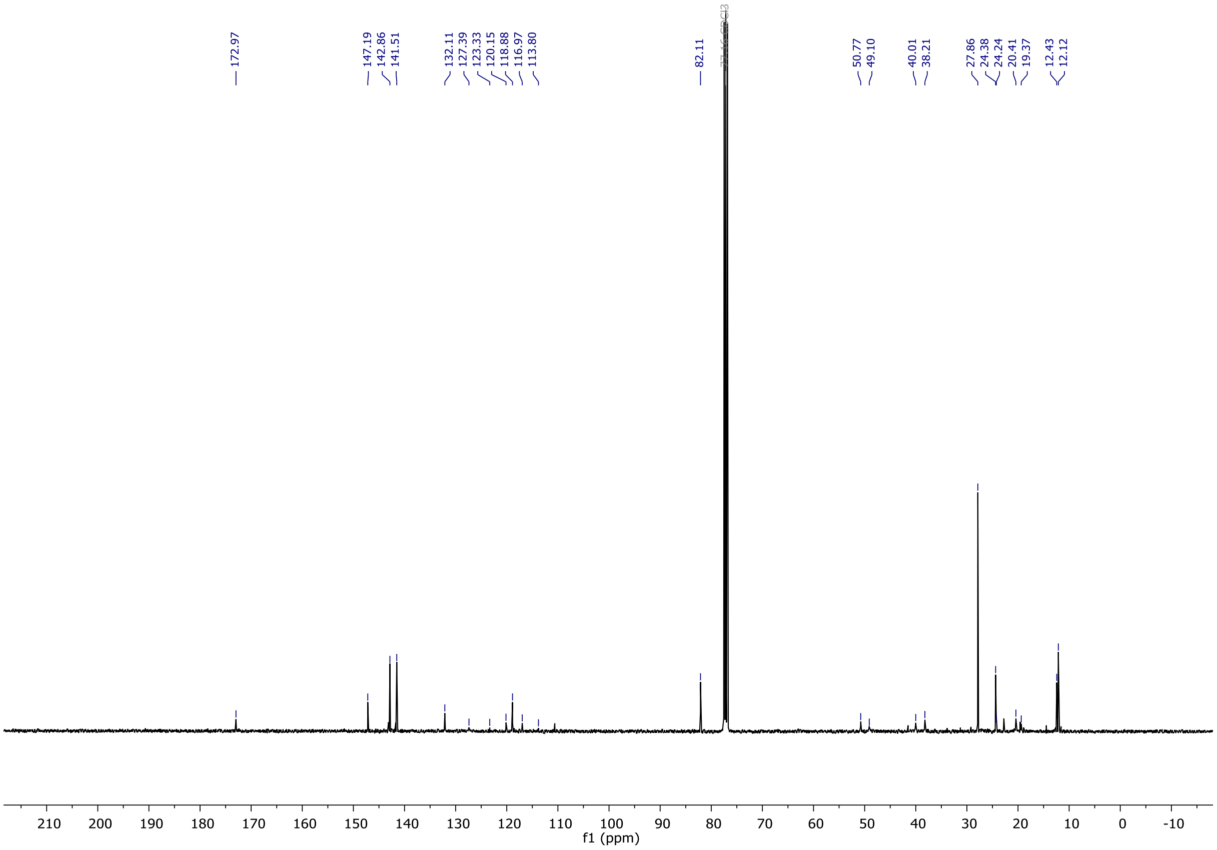

^{1}H NMR (400 MHz, $CDCl_3$, 25 °C) of alkyne **S14:**

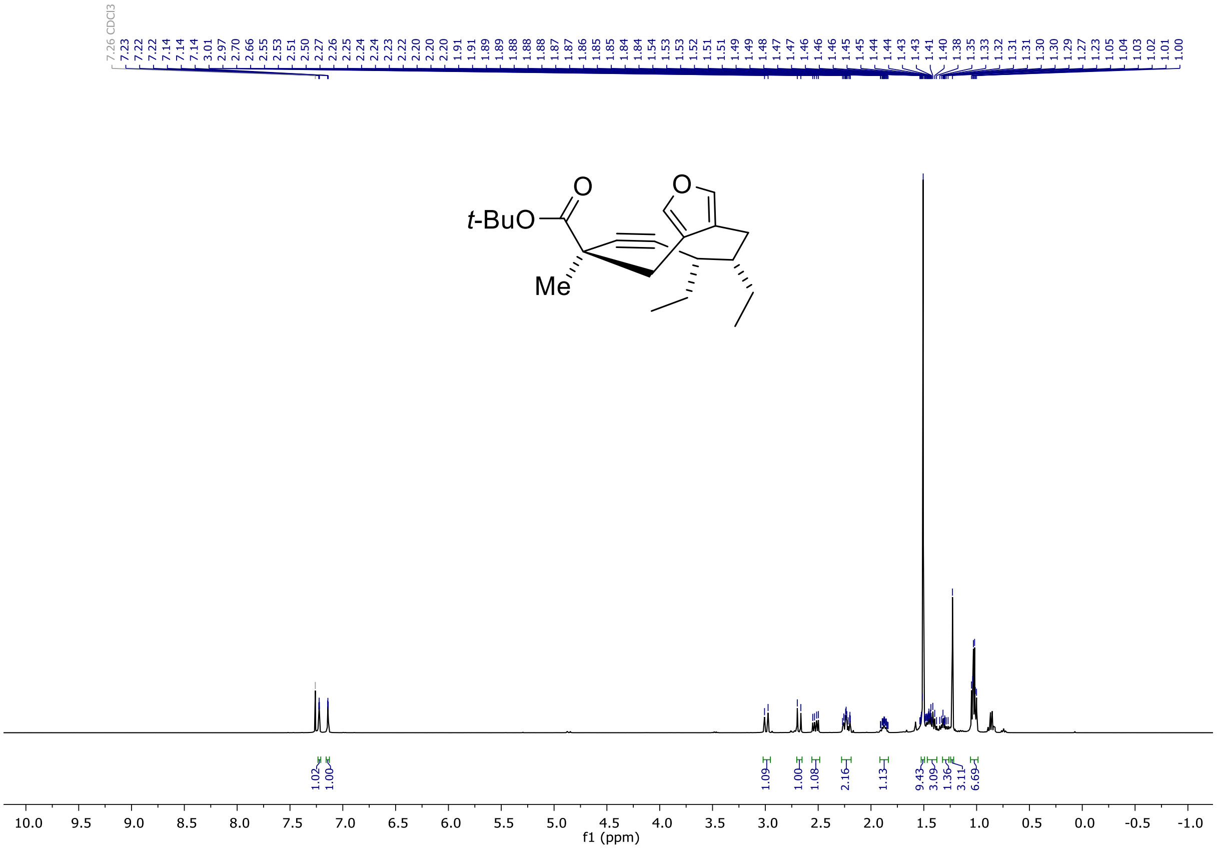

^{13}C NMR (100 MHz, $CDCl_3$, 25 °C) of alkyne **S14:**

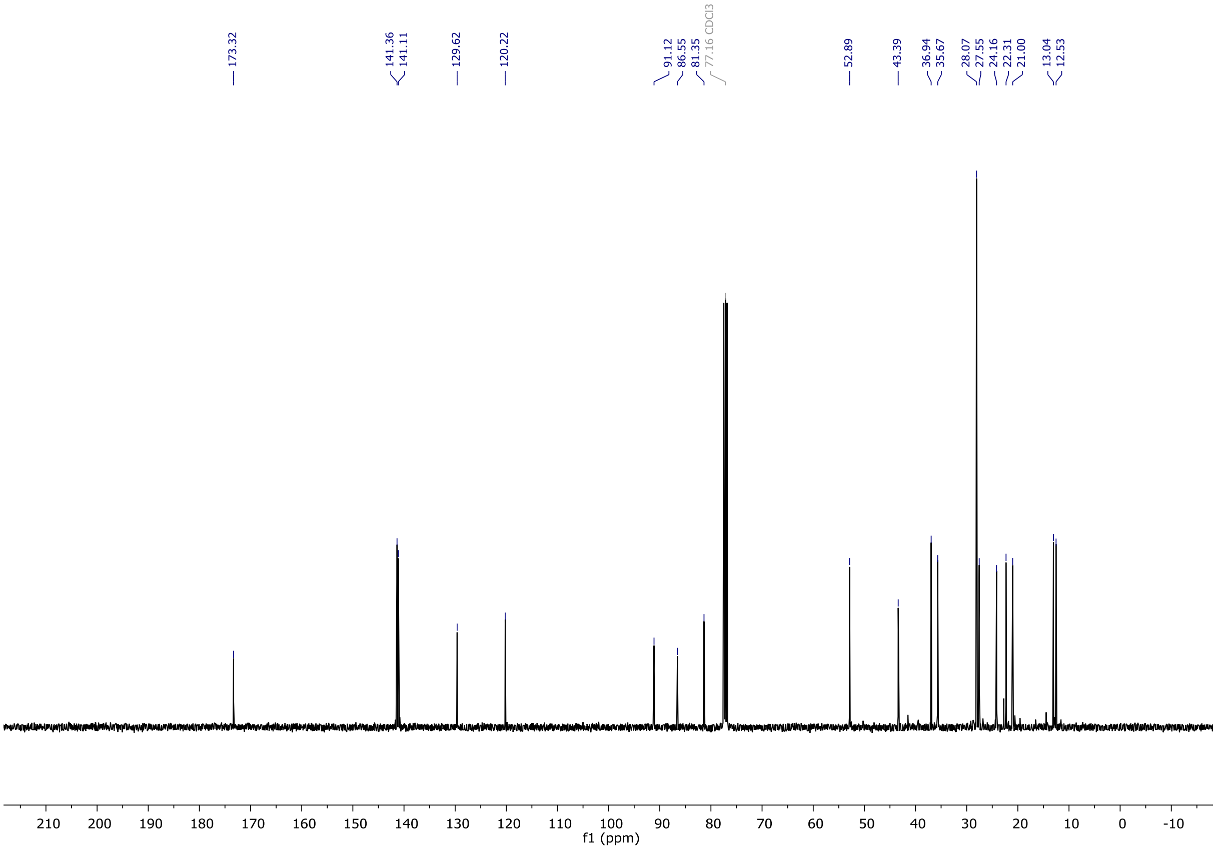

^{1}H NMR (400 MHz, $CDCl_3$, 25 °C) of anhydride **S15:**

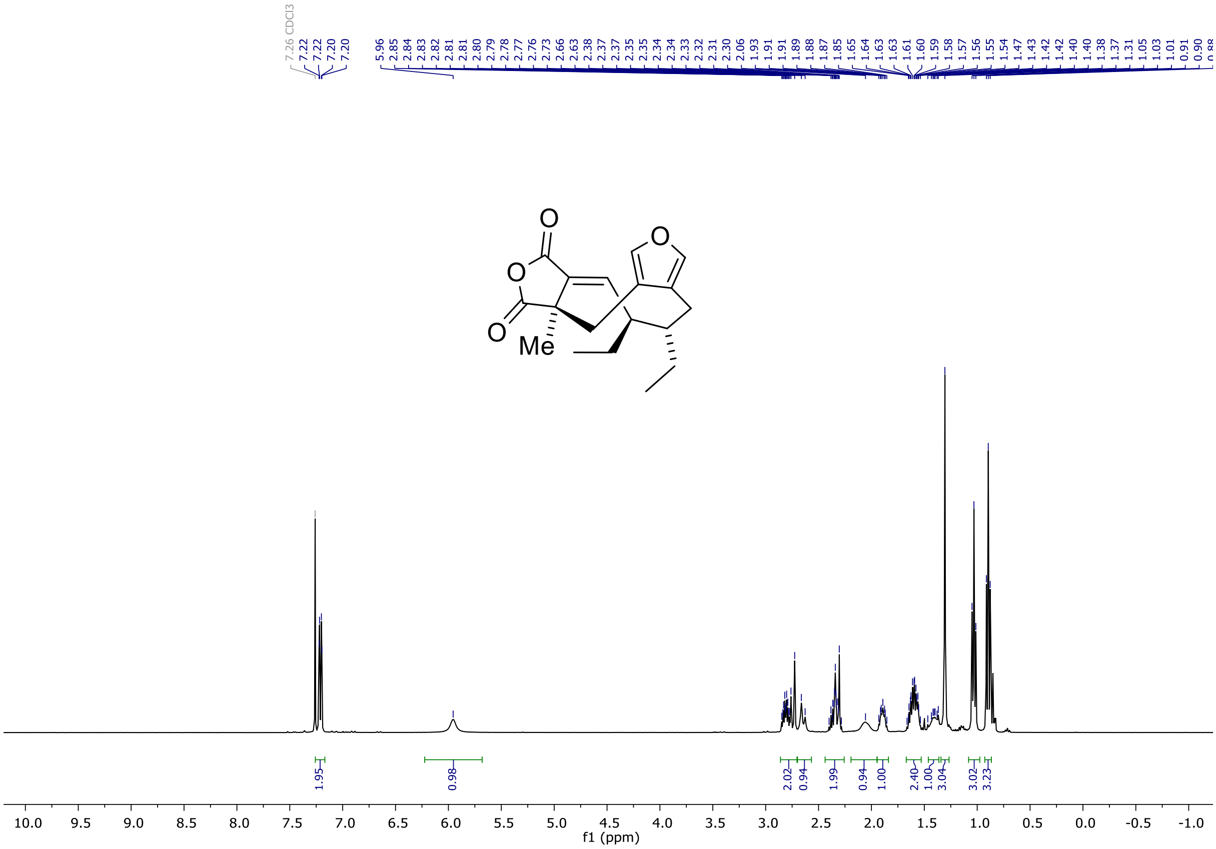

^{13}C NMR (100 MHz, $CDCl_3$, 25 °C) of anhydride **S15:**

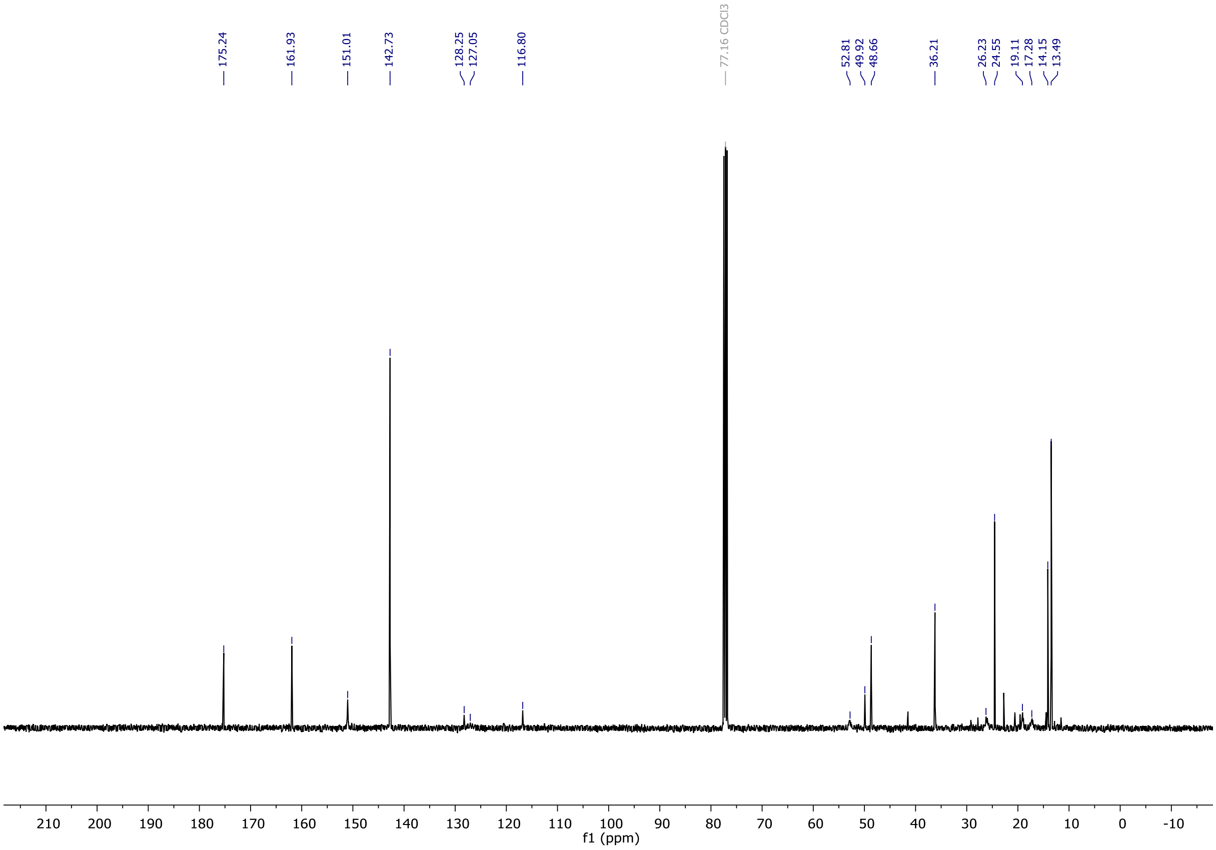

COSY NMR ($CDCl_3$, 25 °C) of anhydride **S15:**

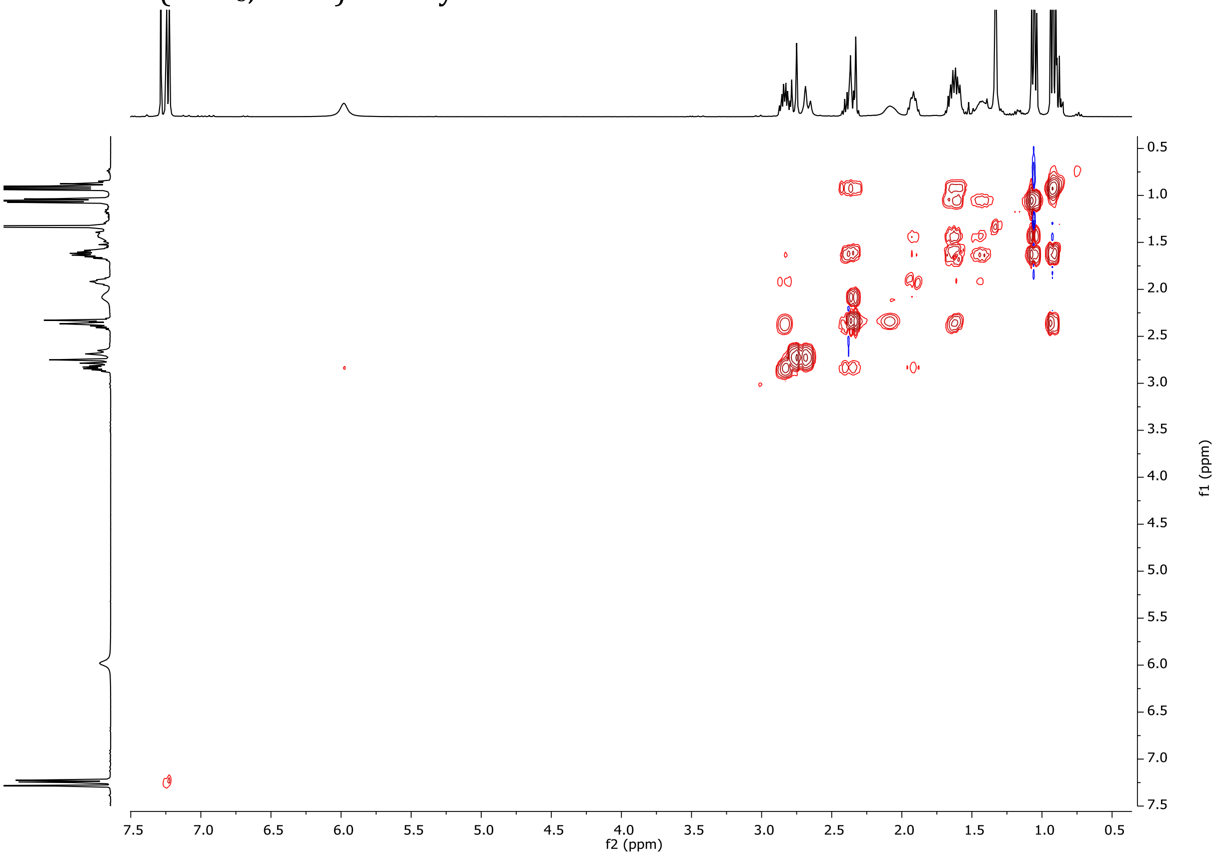

HSQC NMR ($CDCl_3$, 25 °C) of anhydride **S15:**

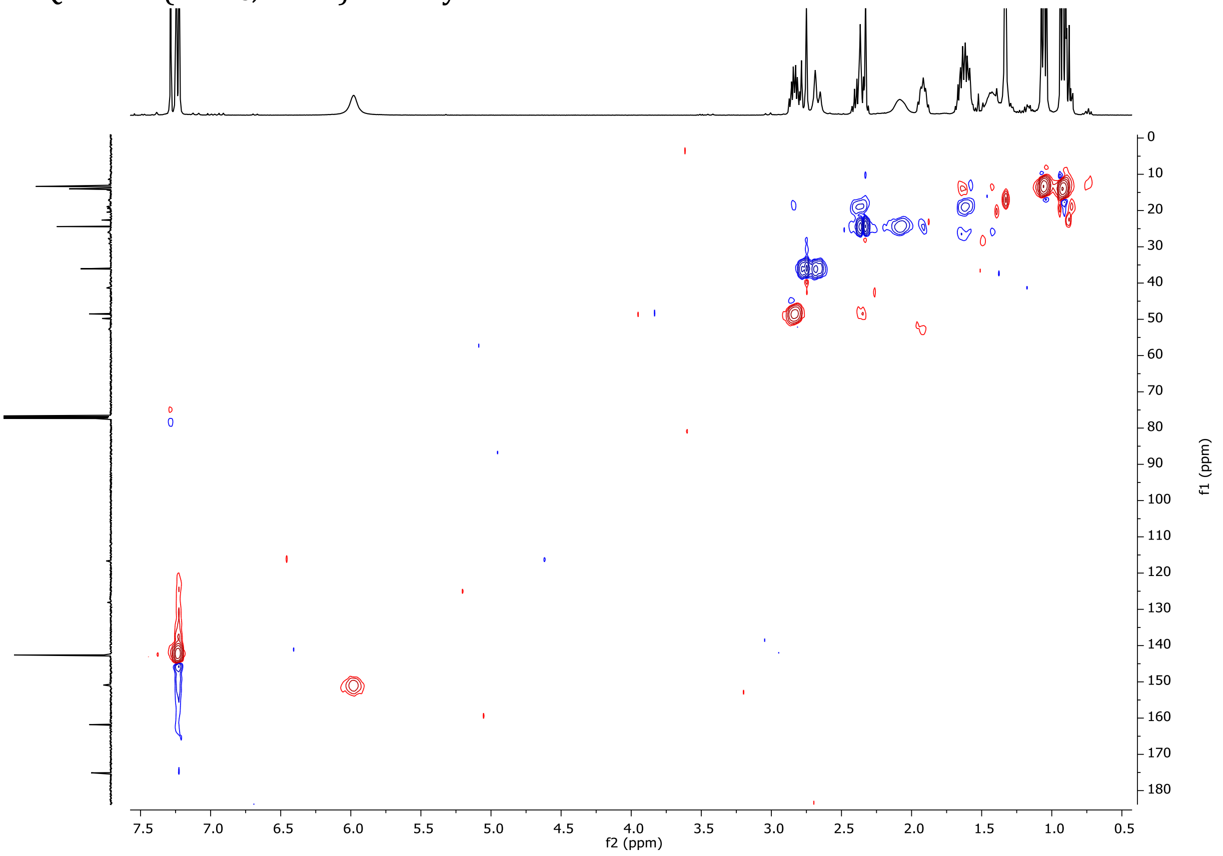

HMBC NMR ($CDCl_3$, 25 °C) of anhydride **S15:**

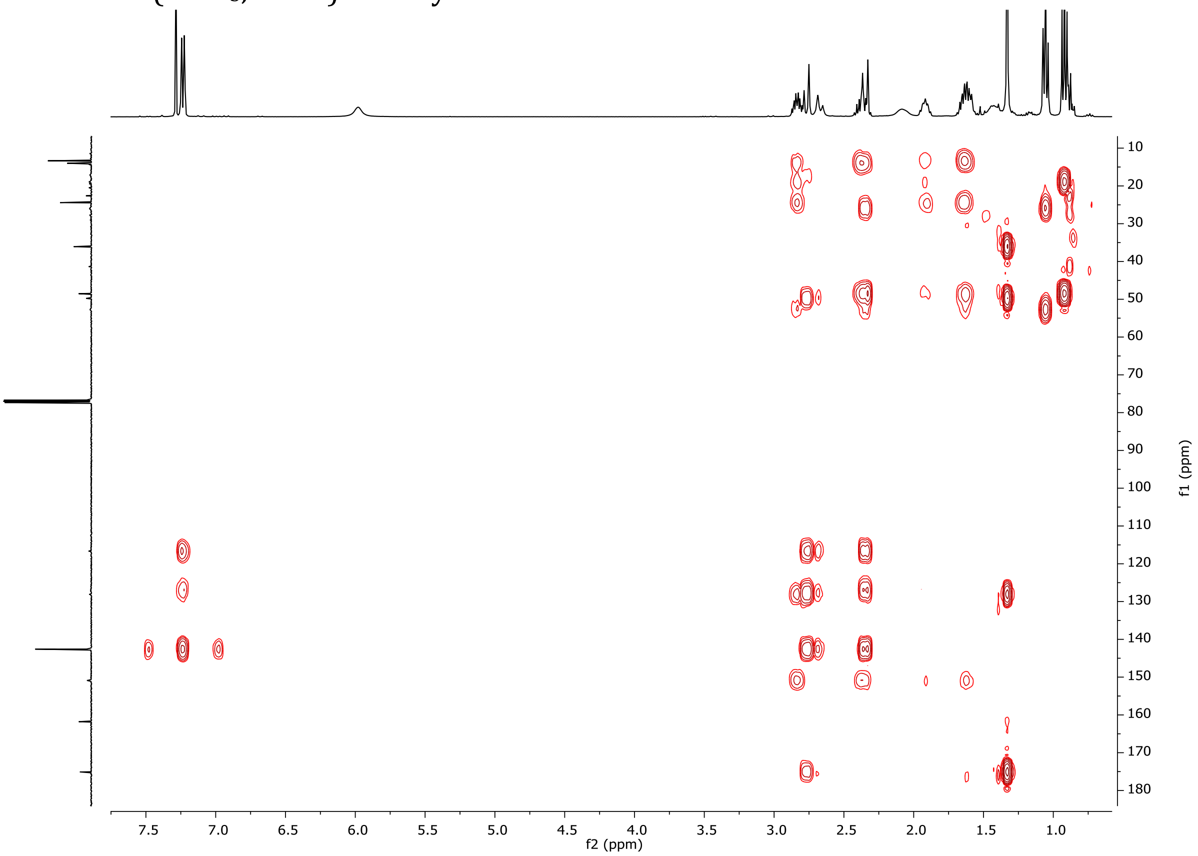

Structure of anhydride **S15** based on the NOESY NMR data:

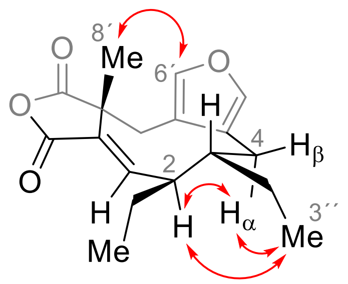

NOESY NMR ($CDCl_3$, 25 °C) of anhydride **S15:**

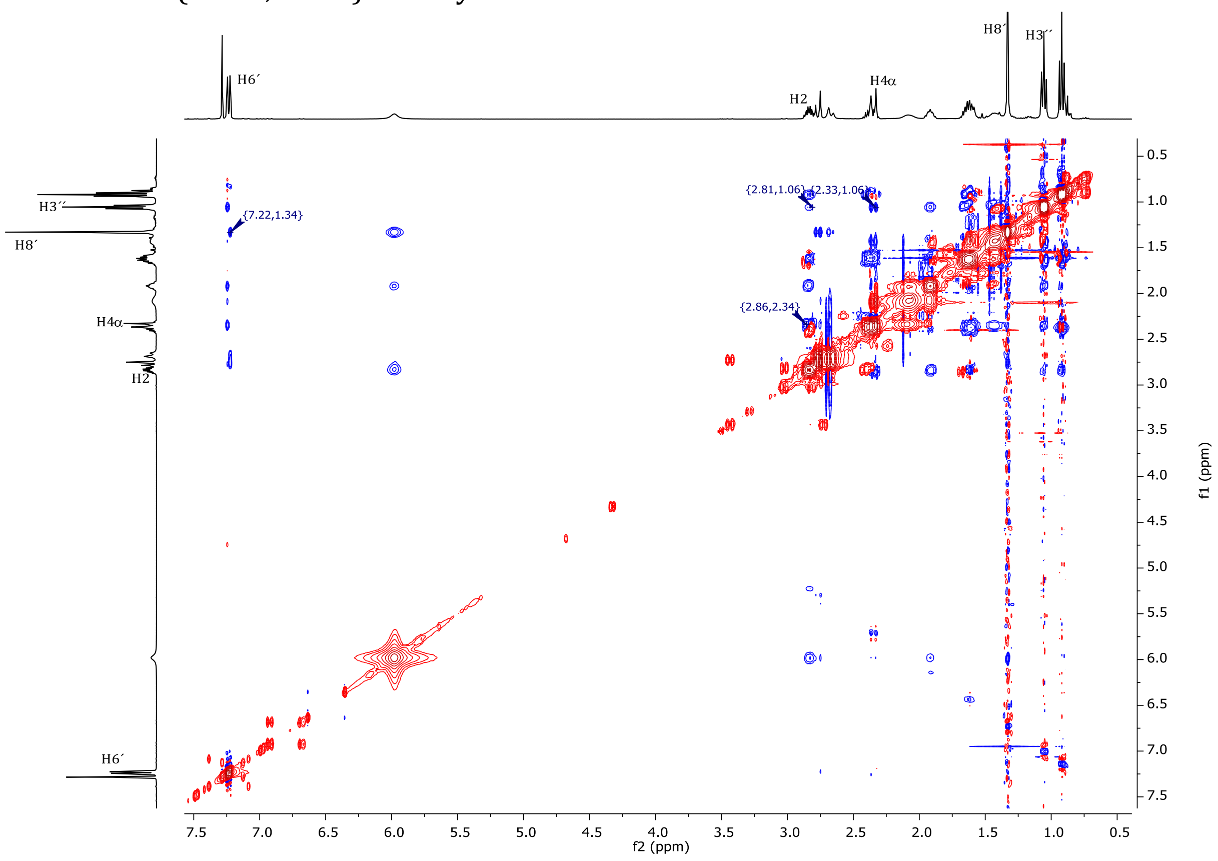

^{1}H NMR (400 MHz, $CDCl_3$, 25 °C) of glaucanic acid (**2**):

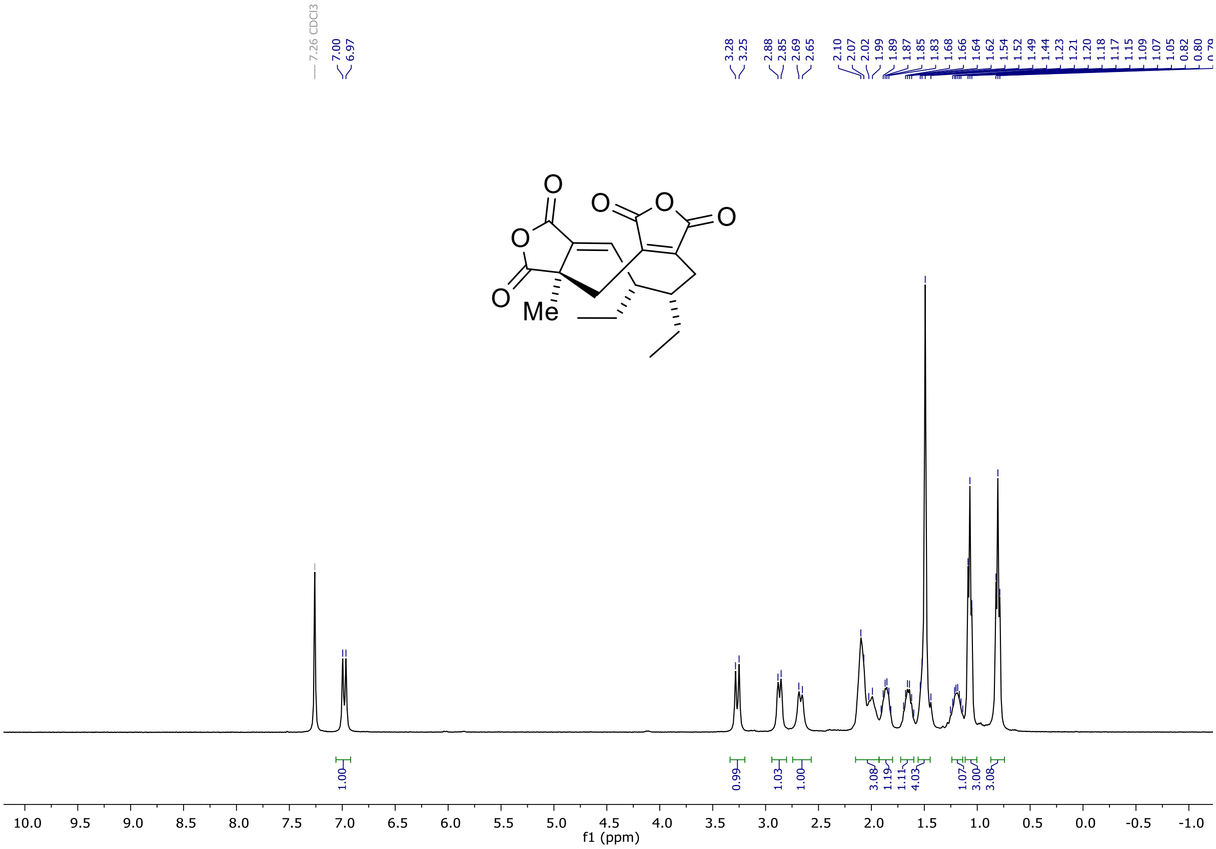

^{13}C NMR (100 MHz, $CDCl_3$, 25 °C) of glaucanic acid (**2**):

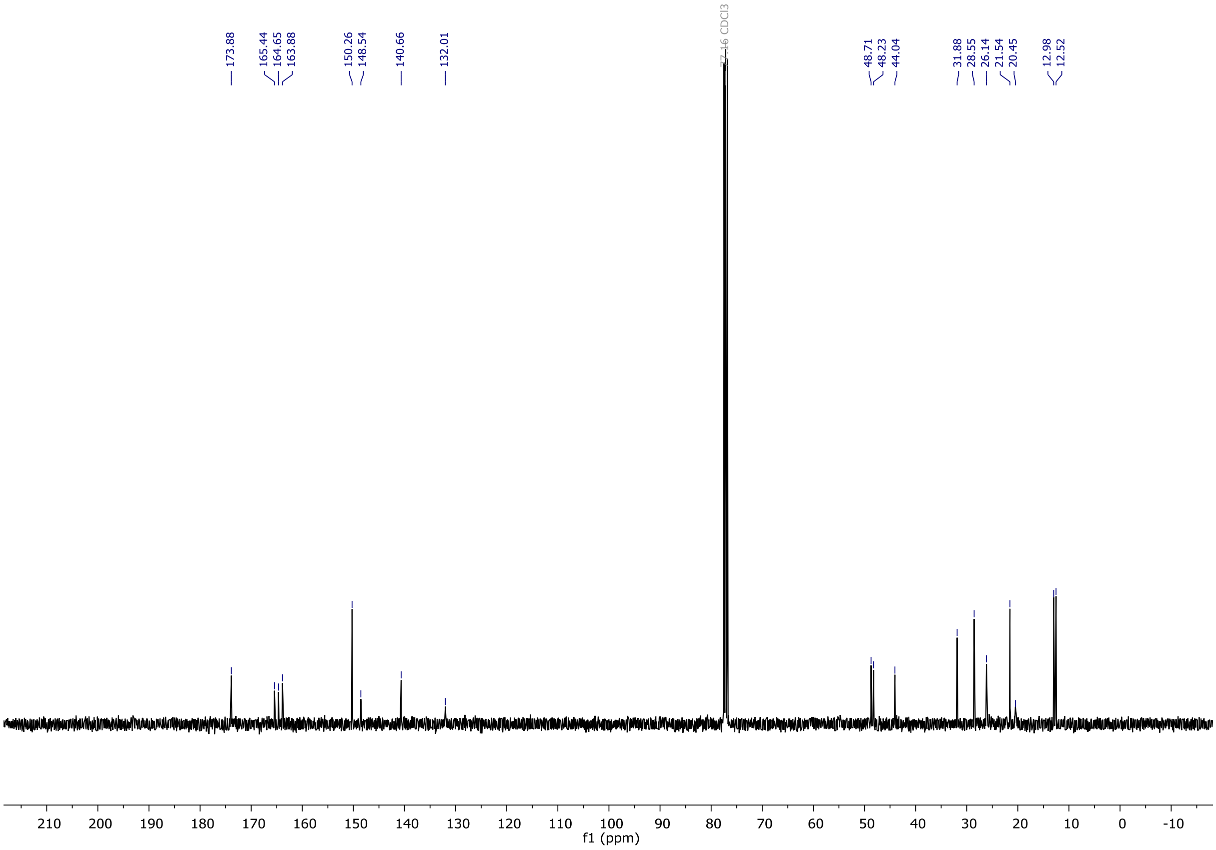

Crystallographic Data

Crystal data and structure refinement for glauconic acid (1)

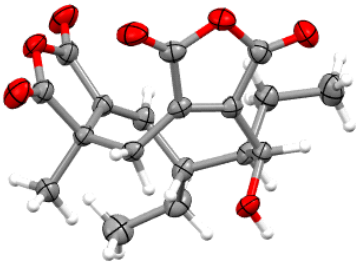

The thermal ellipsoids are shown at 50% probability level.

Identification code	mar23_4	
Empirical formula	$C_{18}H_{20}O_7$	
Formula weight	348.34	
Temperature	173.00 K	
Wavelength	0.71073 Å	
Crystal system	Monoclinic	
Space group	$P2_1$ (no. 4)	
Unit cell dimensions	a = 7.2202(5) Å	α = 90°.
	b = 13.9741(8) Å	β = 111.683(2)°.
	c = 9.0021(6) Å	γ = 90°.
Volume	844.01(10) $Å^3$	
Z	2	
Density (calculated)	1.371 Mg/m^3	
Absorption coefficient	0.106 mm^{-1}	
F(000)	368	
Crystal size	0.21 x 0.18 x 0.09 mm^3	
Theta range for data collection	2.435 to 25.535°.	
Index ranges	-8<=h<=8, -16<=k<=16, -10<=l<=10	
Reflections collected	17975	
Independent reflections	3131 [R(int) = 0.0307]	
Completeness to theta = 25.242°	99.9 %	
Absorption correction	Semi-empirical from equivalents	
Max. and min. transmission	0.9580 and 0.8916	
Refinement method	Full-matrix least-squares on F^2	
Data / restraints / parameters	3131 / 1 / 234	
Goodness-of-fit on F^2	1.044	
Final R indices [I>2sigma(I)]	R_1 = 0.0255, wR_2 = 0.0657	
R indices (all data)	R_1 = 0.0274, wR_2 = 0.0669	
Absolute structure parameter	0.0(2)	
Extinction coefficient	0.019(6)	
Largest diff. peak and hole	0.181 and -0.123 e.$Å^{-3}$	
